# Gene expression and anticancer evaluation of Kigelia africana (Lam.) Benth. Extracts using MDA-MB-231 and MCF-7 cell lines

**DOI:** 10.1371/journal.pone.0303134

**Published:** 2024-06-05

**Authors:** Aasia Kalsoom, Awais Altaf, Huma Sattar, Tahir Maqbool, Muhammad Sajjad, Muhammad Idrees Jilani, Ghulam Shabbir, Saira Aftab

**Affiliations:** 1 Institute of Molecular Biology (IMBB), Center for Research in Molecular Medicine (CRiMM), The University of Lahore, Lahore, Pakistan; 2 School of Biological Sciences, Punjab University, Lahore, Pakistan; 3 Department of Chemistry, The University of Lahore, Lahore, Pakistan; 4 Pakistan Council of Scientific and Industrial Research (PCSIR), Islamabad, Pakistan; 5 Department of Biochemistry and Biophysics, Stockholm University, Stockholm, Sweden; Monash University Malaysia, MALAYSIA

## Abstract

In recent years, a cancer research trend has shifted towards identifying novel therapeutic compounds from natural assets for the management of cancer. In this study, we aimed to assess the cytotoxic activity of *Kigelia Africana* (KA) extracts on breast cancer (MDA-MB-231 and MCF-7) and noncancerous kidney cells (HEK-293T) to develop an efficient anticancer medication. We used gas chromatography mass spectrometry (GC-MS to analyze the constituents of EKA and HKA extracts meanwhile the crystal violet and the MTT (3-(4,5-Dimethylthiazol-2-yl)-2,5-Diphenyltetrazolium Bromide) assays were used to examine the possible cytotoxic effects of plant extracts on our cancer cell lines along with non-cancerous control. The quantitative real-time PCR (RT-PCR) was run on cell samples to evaluate the differential expression of cell proliferative markers of cancer (BCL-2 and TP53). These phytochemicals have been reported to have binding affinity for some other growth factors and receptors as well which was evaluated by the *in-silico* molecular docking against Bcl2, EGFR, HER2, and TP53. Our Morphological observation showed a significant difference in the cell morphology and proliferation potential which was decreased under the effect of plant extracts treatment as compared to the control samples. The ethanol extract exhibited a marked antiproliferative activity towards MDA-MB-231 and MCF-7 cell lines with IC_**50**_ = 20 and 32 μg/mL, respectively. Quantitative RT-PCR gene expression investigation revealed that the IC_**50**_ concentration of ethanolic extract regulated the levels of mRNA expression of apoptotic genes. With the target and active binding site amino acids discovered in the molecular docking investigation, TP53/Propanoic acid, 3-(2, 3, 6-trimethyl-1, 4-dioxaspiro [4.4] non-7-yl)-, methyl ester (-7.1 kcal/mol) is the best-docked ligand. The use of this plant in folk remedies justifies its high *in vitro* anti-cancer capabilities. This work highlights the role of phytochemicals in the inhibition of cancer proliferation. Based on all these findings, it can be concluded that EKA extract has promising anti-proliferative effect on cancerous cells but more study is required in future to further narrow down the active ingredients of total crude extract with specific targets in cancer cells.

## Introduction

Breast cancer is one of the major non-communicable diseases (NCDs) that has become a significant threat to the public and claims thousands of lives every year [1]. The development of novel and precise methods for the early detection of breast cancer and treatment monitoring is imperative in low- and middle-income countries where healthcare facilities are often scarce. GLOBOCAN data estimated 19.3 million new cancer cases in 2020, resulting in around 10 million cancer deaths [2]. According to WHO data, malignant neoplasms may cause 107.8 million disability-adjusted life years (DALYs) for females, from which breast cancer demonstrates 19.6 million DALYs [3] with 2.26 million newly diagnosed cases in 2020 [4]. The early stage of cancer development is associated with inflammation while later stages have metastasis, progression, and relapse. Among many factors, genetic mutation is the dominant cause but among other reasons, alcohol consumption, obesity, and smoking also accelerate the progression of cancerous tumours to the advanced stages which become untreatable eventually [5]. Numerous mutated genes play a role in breast cancer proliferation, of which TP53 (Tumour Protein 53), is the most well-known mutant gene implicated in several cellular signals that include blood vessel formation, cell cycle regulation, metabolic reactions, and nucleic acid repair. Depending on the genetic subtypes of breast tumours, TP53 gene mutations are presumed to be present in approximately 30% of breast cancers [6]. The ER-positive (ER+) subtype of breast cancer accounts for 75% of all cases, while the other subtypes are characterized by their dependence on the estrogen receptor (ER), progesterone receptor (PR), and/or human epithelial receptor2 (HER2) [7]. The *BCL-2* (B-cell Lymphoma 2) is primarily responsible for regulating the permeability of the mitochondrial membrane and has an anti-apoptotic function. The gene halts apoptosis and causes the uncontrollable division of cells which exhibit homologous behaviour as they increase [8]. Bcl-2 has been identified as a new category of oncogenes and a target protein that encourages carcinogenesis by inhibiting apoptosis but has no effect on cell proliferation. Abnormal expression of Bcl-2 was observed in several human malignancies, including liver, colon, lung, stomach, prostate, breast cancer, and neuroblastoma [9]. Therefore, targeting these genes involved in carcinogenesis is one of the important therapeutic approaches to improve anti-breast cancer therapies.

Medicinal plants are regarded as necessary in all the elements of human life including culture, environment, nutrition, religion as well as society. Among these facts, the consumption of medicinal plants as substitutes for different treatments began approximately 60,000 years ago [10]. Phytomedicine is the application of phytoconstituents in treating diseases and enhancing the quality of life. The current focus of nanomedicine and phytomedicine approaches stems from the primary problems with the currently available chemotherapeutic options along with radiotherapy results in nonspecific targeting and damage even the normal cells around the tumour which results in wide spectrum side effects for patients and they suffer through the treatment period with very low-quality lifestyle. Phytonutrients (phytomedicines) are compounds derived from plants and can enhance the effectiveness of treatment and reduce the side effects of anti-cancer therapy in cancer patients [9]. Many phytochemicals with anti-cancer potential are naturally abundant like berberine, camptothecin derivatives, cephalotaxus, colchicine, combretastatins, ellipticine, taxanes, triterpenoid acids, and vinca alkaloids have well reported anti-cancer potential. The derivatives of vinca alkaloids including vinblastine, vincristine, vindesine, vinflunine, and vinorelbine induce cell cycle arrest and have demonstrated anti-proliferative and anti-mitotic potential [11]. Vinblastine and vincristine were the first two drugs to be isolated and Paclitaxel was the first medication that had a significant impact in treating cancer [11]. Among other phytocompounds that demonstrated potential anticancer activity against breast cancer cell lines (MCF-7 and MDA-MB-231) are actein, citral, curcumin, epigallocatechin, p-coumaric acid, ricinoleic acid, and ricinine [12].

*Kigelia africana* (Lam.) Benth is a natural flora of tropical Africa especially of South and West Africa, but it is also found in other parts of the world like countries in South Asia [13]. The plant comprises several phyto-compounds such as coumarins, flavonoids, iridoids, naphthoquinones, terpenes, and terpenoids [14]. One study reports *in vitro* cytotoxic activities of dichloromethane and methanolic extracts of the KA plant showed potent anticancer activity against breast cancer cells [15]. Ethyl acetate, hexane, and methanolic extracts of *K. pinnata* plant were fractionated and tested for their cytotoxicity on rhabdomyosarcoma human cancer cells and demonstrated strong anticancer and antioxidant activities. Fruit and stem bark extracts have also been screened for their cytotoxicity and have exhibited favourable outcomes against renal carcinomas as well as melanomas. Additionally, the plant extracts also demonstrated anti-diarrheal, anti-inflammatory, anti-malarial and even anti-leprotic properties [16]. This is the first comprehensive report to identify the possible active constituents for the establishment of anti-breast cancer medicines. The BCL-2, EGFR, HER-2, and TP53, proteins/receptors were targeted using a long-timescale docking procedure. The results of this study provide a scientific justification for using herbal therapeutic options against breast cancer. However, further studies are needed to narrow down the most effective constituents in the crude extract having anti-cancer potential.

## Materials and methods

### Sample collection and preparation of extracts

The KA leaves used in this study were collected from the natural reserve Lal Suhanra National Park which is situated in Bahawalpur District in Punjab Province, Pakistan. The collection was conducted during the summer season, to ensure the active growth season. Only healthy plant specimens were collected and identified (GC. Herb. Bot. 3909) by a qualified taxonomist Dr. Zaheer-ud-Din Khan. For the extraction procedure, an appropriate solvent system (ethanol-90%, and hexane-95%) was chosen depending on the ability to extract compounds of interest. After collection, the leaves were washed with distilled water, dried in the dark at 37°C and then ground into powder. Optimal extraction (maceration) efficiency was ensured by maintaining a constant ratio of plant material to solvent (3:10 w/v) and left at room temperature in this solvent ratio for up to 7 days before the extraction procedure to ensure complete maceration. The extraction procedure was done at a controlled room temperature of around 20–25 ^**°**^C to avoid the degradation of heat-sensitive compounds. When higher yields were required, the extraction process was repeated with fresh solvent each time. The mixture was filtered and a rotary evaporator (Heidolph Hei-Vap, Germany) removed the residual solvents from extracts at 35–40 ^**°**^C and stored them in the dark at -20 ^**°**^C. The dried extracts were obtained by lyophilization (Freeze drying) and stored at 4 ^**°**^C [17], and to calculate the percentage yield of the extracts, the following equation was applied [18].


$$\%\;\text{Yield}\;\text{of}\;\text{extract}\;(\text{g})=\frac{\text{Weight}\;\text{of}\;\text{dried}\;\text{extracts}}{\text{Weight}\;\text{of}\;\text{powdered}\;\text{material}}\times 100$$ (1)

### Characterization of plant extracts

The lyophilized ethanol and n-hexane extracts of KA leaves were sent to the International Center of Chemical and Biological Sciences (ICCBS) HEJ, Research Institute of Chemistry, University of Karachi, Karachi, Pakistan, for the gas chromatography-mass spectrometric (GC-MS) analysis to characterize the bioactive phytocompounds. The Agilent Technologies (7890A) GC-MS triple quad system with EI and CI ion source was used for GC-MS analysis of plant extracts. The instrument contains DB-5 MS capillary standard and a non-polar column having a 30 mm **×** 250 μm dimension with ID**×**0.25 μm film. The injector was run at 250 ^**°**^C, while the oven operated at a holding time of 50 ^**°**^C for 3 min, followed by 7–10°C per minute to 180°C for 25 minutes. The initial pressure was 9 psi with a flow rate of 1 mL/min, and the average velocity was 38 cm/sec. The compound identification was based on the HEJ GC-MS library along with their retention indices comparison. Helium 99.999% was utilized as the exporter gas during the GC-MS experiment, with a 1 mL/min column flow rate. The NIST library contains over 62000 patterns and was used to examine the mass spectrum. Understanding various characteristics of known and unknown compounds was made easier by comparing the spectra with the NIST library. The peak area expression of the "TIC" (total ionic chromatogram) was used to measure the relative percentage levels of each component, with calculations carried out automatically.

### Cancer cell culture and maintenance

The breast cancer (MCF-7, and MDA-MB-231), and non-cancer (HEK-293T) cell lines were provided by the University of Lahore’s cell BioBank (IMBB/CRiMM). The cancer cell lines were freshly cultured as monolayer in Dulbecco’s Modified Eagle’s Medium (DMEM) (Caisson Lot#02160032), supplemented with 10% of fetal bovine serum (FBS) (Sigma-Aldrich Lot#BCBS3184V), and 100 U/mL of Pen-Strep, a combination of Penicillin and Streptomycin antibiotics (Caisson Lot#10201011). On the other hand, the human kidney cells (HEK-293T) were cultured with 15% FBS in Minimum Essential Media (MEM) (Gibco) at 37°C and 5% CO_2_. To carry out trypsinization, 3 mL of trypsin (Gibco Lot#1297823) was added until the cells detached from the flask’s surface. Complete media (5 mL) was added to stop the reaction, and the cells were subjected to centrifuge at 1500 rpm for 5 minutes before the supernatant was aspirated. As a part of the passage procedure, around 5mL of suspension culture was seeded into a T75 cm^2^ flask [19].

### Determination of *in vitro* activity by cell viability and cytotoxicity assays

#### Colorimetric MTT assay

Human breast cancer cell lines were used for the determination of the cytotoxic activity, it is not possible, using the assay, to determine the mechanism of action of the extracts, compounds, DNA damaging agents, antimitotics, and so on are all measured as general cytotoxic agents in the assay. Although it is not possible to determine the mechanisms involved with this assay, it is possible to evaluate a wide range of extract concentrations using this method. KA leaf extracts were examined for their potential cytotoxic effects by colourimetric MTT [3-(4,5-Dimethylthiazol-2-yl)-2,5-Diphenyltetrazolium Bromide] test. The activity of extracts was examined against breast cancer (MDA-MB-231, and MCF-7), and non-cancer (HEK-293T) cell lines with 1**×**10^4^ cells/well grown in 96 flat-bottom well plates [20]. Crude ethanol and n-hexane extract stock solutions were prepared in dimethyl sulfoxide (DMSO) (Invitrogen Inc., USA) at 160 mg/mL concentration. The cells were further supplemented with various extract concentrations (400, 200, 100, 50, and 10 μg/mL) and incubated for 72 hours in a 5% CO_2_. The same procedure was conducted for HEK-293T cells in MEM medium with 15% FBS. Cisplatin (10 μg/mL) was procured from INMOL cancer hospital (Receipt No. 651278) and considered as a positive control, while the DMSO (0.1%) was considered as vehicle control. Control (UT) cells were cultivated in DMEM media with 2% FBS to compare before and after treatment effects. After 72 hrs., phosphate buffer saline (PBA) was used to clear the cells and dispensed with MTT (20 μL) (Invitrogen Inc., USA), and kept in an incubator for another two hours. After two hours, the formazan crystals were dissolved by adding 150 μL of DMSO in each well. The absorbance at 570 nm was measured using a microplate reader (BIO-RAD). Each sample was evaluated in triplicate. IC_**50**_, the half-maximal inhibitory concentration, was assessed by applying the linear regression method [21].

#### Cell viability assay

A crystal violet (0.1%, WV) viability assay was performed to evaluate the cellular viability. In a 96-well microtiter plate, MDA-MB-231, MCF-7, and HEK-293T cells were grown in 200 μL of complete DMEM media and treated with different concentrations. After incubation at 37°C and 5% CO_2_ for 72 hrs., the plate-cultivated adherent cells were fixed with 70% ethanol for 10 min at 20–22°C. After that, the CV solution (100 μL) was used to stain the cells and allowed to stay for 25 minutes. The cells were cleared using PBS to remove the debris, furthermore, 200 μL of 10% triton X-100 (Sigma-Aldrich CAS#9036195) was used to clear the cells from debris for 25 minutes. The sample absorbance at 570 nm was calculated using a spectrophotometer (BIO-RAD) [22]. The percentage of viable adherent cells was measured as a percentage [23].


$$\%\;\text{of}\;\text{cell}\;\text{viability}=\frac{\text{Absorbance}\;\text{mean}\;\text{of}\;\text{treated}\;\text{samples}}{\text{Absorbance}\;\text{mean}\;\text{of}\;\text{control}\;\text{samples}}\times 100$$ (2)

#### Observation of cytomorphological changes

Plant extracts of varying concentrations (10, 50, 100, 200, and 400 μg/mL) were used to treat fully grown cancer (MDA-MB-231 and MCF-7) and non-cancer (HEK-293T) cells, followed by incubation for 72 hours. Floid™ Imaging Station was used to analyze the overall morphological changes in both the cell lines after treatment with the plant extracts and the alterations were compared with the untreated (UT) group that served as control of the study.

### Relative expression analysis of selected oncogenes

#### RNA extraction and cDNA synthesis

The data obtained were analyzed using a linear regression model to get the IC_**50**_ value of each plant extract. To assess the gene expression profile and the effect of ethanol extracts, the MCF-7, and MDA-MB-231 cell lines were used. The cell lines obtained were grown in a T-25 culture flask. After 24 hrs, the concentration doses were given and incubated for 72 hrs. Total RNA was extracted through Thermo Scientific’s Pure Link RNA micro kit (CAT: 12183018A) following the manufacturer’s protocol. The concentrations used in this test were based on previously calculated IC_**50**_ (MDA = 20 μg/mL: MCF-7 = 32 μg/mL) values [24]. To estimate extracted RNA concentration from cancer cell samples (MDA-MB-231, and MCF-7), 1 μL of the sample was used by Nanodrop (Thermo scientific ND 2000/2000c Spectrophotometers) at 260/280 nm, and the concentrations were obtained as ng/μL. The cDNA was synthesized using 1 μg of total RNA isolated using ABScript II cDNA first-strand synthesis kit (Lot: 962100J09W09).

#### Real-time PCR analysis

The region to amplify *TP53*, *BCL2*, and *Hprt1* genes encompasses a partial 5´ upstream region. NCBI was used to retrieve the primer sequences and the primers were designed with the use of the Primer3 online software (https://primer3.org/) (Table 1) [25]. To investigate the relative expression of genes, real-time PCR (Qiagen Rotor-Gene Q 5plex HRM) was used along with SYBR Green (Thermo Scientific, USA). The expression of each gene (*BCL2*, *Hprt 1*, and *TP53)* was compared quantitatively using a comparative threshold cycle (CT) approach. For each gene of interest, *Hprt1* was used as a reference gene, and each Ct value was established to the Ct value of *Hprt1* RNA. Each reaction was carried out three times. Table 1 contains the sequences of the primers used.

**Table 1:** Details of primers designed and ‘AZENTA’ IDs.

Sr. No	Gene names	Primer sequence/ID
**1.**	*TP53*-F	5´TTCGACATAGTGTGGTGGTG 3´ **ID:** S2052409355K
	*TP53*-R	5´CCCTTTTTGGACTTCAGGTG 3´ **ID:** S2052409356K
**2.**	*BCL2*-F	5´GGGATTCCTGCGGATTGACA 3´ **ID:** S2052409351K
	*BCL2*-R	5´TCCCGGTTATCGTACCCTGT 3´ **ID:** S2052409352K
**3.**	*Hprt1*-F	5´CGAACCTCTCGGCTTTCC 3´ **ID:** S2052409353K
	*Hprt1*-R	5´TCCCCTGTTGACTGGTCATT 3´ **ID:** S2052409354K

Reagents for a single RT-PCR (Qiagen Rotor-Gene Q 5plex HRM) reaction include (2 μL or 200 ng) cDNA, endonuclease-free water (variable), primer forward + reverse (5 μM), and SYBR Green Master Mix (12.5 μL). Table 2 contains the RT-PCR cycling conditions for *TP53*, *BCL2*, and *Hprt1*. The Ct value of the endogenous *Hprt1* gene was subtracted from the mean Ct values of the target genes *TP53* and *BCL2* to determine ΔCt. The ΔΔCt value was calculated using the formula: ΔCt of sample- ΔCt of calibrator, while the fold change was calculated by the 2^-ΔΔCt^ method. Real-time PCR was used to assess the expression of the *BCL2* and *TP53* genes in treated and untreated cancer samples. Each reaction was carried out in triplicate [25].

**Table 2:** Optimized thermal profile for qPCR.

qPCR steps	Temperature	Duration	Repeat
Pre-denaturation	94°C for	10 min	1
Denaturation	94°C for	15 seconds	35
Annealing	63°C for	15 seconds	
Elongation	72°C for	15 seconds	
Final Elongation	72°C for	10 min	1

### Molecular docking

#### Protein target selection and preparation

The 3-dimensional (3D) X-ray crystallographic structures of Bcl-2, EGFR, HER2, and TP53 proteins/receptors with PDB IDs: (2OCJ, 2W3L, 3poz, and 1n8z) were obtained from the Research Collaboratory for Structural Bioinformatics (RCSB) protein (URL: https://www.rcsb.org) data library. To prepare the proteins for molecular docking, hydrogen was added, water ions, heteroatoms, and inhibitors were removed, and the proteins were then saved in PDBQT format [26]. PyMOL (version 2.5.4, 2010) was used to identify the docked complexes and possible residues at the active site. For docking the grid box was positioned at the co-crystallized ligand, and the data was saved in a config.txt file using the Autodock 4.2.6 software. The automated Protein-ligand interaction profiler (PLIP) website was used to analyze the various interacting residues along with their bond lengths involved in creating stable complexes [27].

#### Ligand selection and preparation

PubChem (https://pubchem.ncbi.nlm.nih.gov) databank was used to obtain the structures of the phytochemicals with potential anticancer activity, which were then saved in an SDF format and subsequently converted in PDB format using BIOVIA Discovery Studio Visualizer (Client 2021). For docking, each of the small molecules was uploaded independently and arranged by the Autodock® vina tool and was saved in PDBQT format [28].

#### Screening of compounds for drug-likeness

The assessment of the drug-like chemicals using Lipinski’s RO5 is a crucial stage in the drug development process to establish whether a small molecule is orally active or not. SwissADME (http://www.swissadme.ch/index.php) was used to evaluate the pharmacokinetic parameters of the ligands. The primary objective was to obtain any information that would support the development and research process. The admetSAR (http://lmmd.ecust.edu.cn/admetsar2) and pkCSM (https://biosig.lab.uq.edu.au/pkcsm) websites were used to upload the structures of the chosen phytochemicals in canonical SMILES format to investigate the pharmacokinetic properties such as BBB diffusion, carcinogenicity, cytochrome P450 enzyme inhibition, (HIA) human intestinal absorption of compounds, level of solubility, mutagenicity, and toxicity [26].

#### Molecular docking analysis

After preparing the proteins and ligands for Autodock® 4.2.6, the desired docking conformations were achieved by fixing the x, y, and z dimensions with a resolution of 0.500 Å. Docking calculations with a decreasing number of energy evaluations were carried out. The structures showing the interactions between the proteins and ligands were studied using Discovery Studio client 2021 [29]. The three-dimensional protein-ligand postures were used to understand the binding mechanisms of complexes. A variety of interactions including hydrogen bonding and hydrophobic interactions were examined using the 2D illustrations of complexes. Each protein’s grid center was selected so that it matched the active sites of the protein (Table 3).

**Table 3:** Grid box dimensions for target proteins.

Targets	Grid Dimensions			Size			Spacing	Exhaustiveness
	X	Y	Z	X	Y	Z		
TP53	20.681	19.971	14.415	40	40	40	0.775	8
Bcl-2	40.059	30.580	-5.437	40	40	40	0.481	8
EGFR	20.341	30.686	9.128	40	40	40	0.481	8
HER2	-1.078	86.773	129.24	40	40	40	0.853	8

### Statistical analysis

Each experiment was conducted three times, and the mean and standard deviation were used to express the findings. To ascertain how the three variables interacted, a one-way analysis of variance (ANOVA) and Tukey’s test using Graph Pad Prism 8.0 was used [30]. The proportion of variance in the result variable is represented by the R-squared statistics, which were obtained from analyses based on the general linear model (e.g., regression, ANOVA). The data were analyzed using a linear regression approach to generate the IC_**50**_ values; the statistical significance was assessed through a *p*-value of 0.05 or less.

### Ethical statement

The current study was conducted *in vitro* and *silico* and did not include any participants that mean patients (human) or animals. The submitted study protocols were approved by the Research Ethic Committee of the Institute of Molecular Biology and Biotechnology, The University of Lahore, Lahore, Pakistan following the institutional, national, and international ethical standards and legislation.

## Results

### Total yield of crude extracts

It has been found that the EKA (Ethanolic extract of *K. africana*) extract had a yield percentage of 18.23%. In comparison, the HKA (n-hexane extract of *K. africana*) extract yielded an extraction percentage of 8.15%.

### Profiling of phytocompounds by gas chromatography-mass spectroscopy (GC-MS)

**Fig 1:**
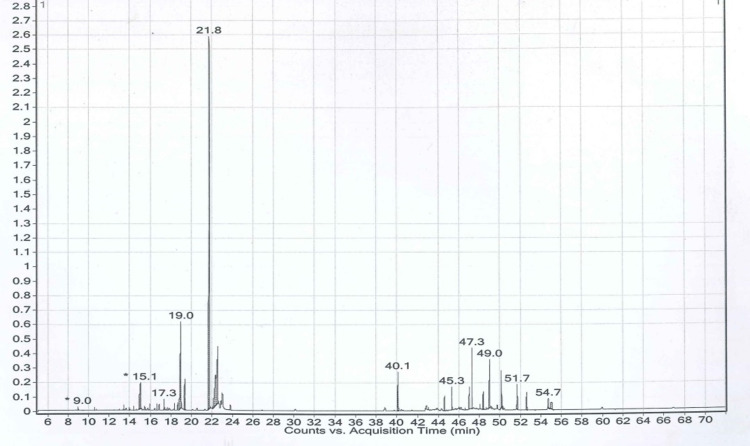
GC-MS analysis of *K. africana* ethanol (EKA) extract.

**Fig 2:**
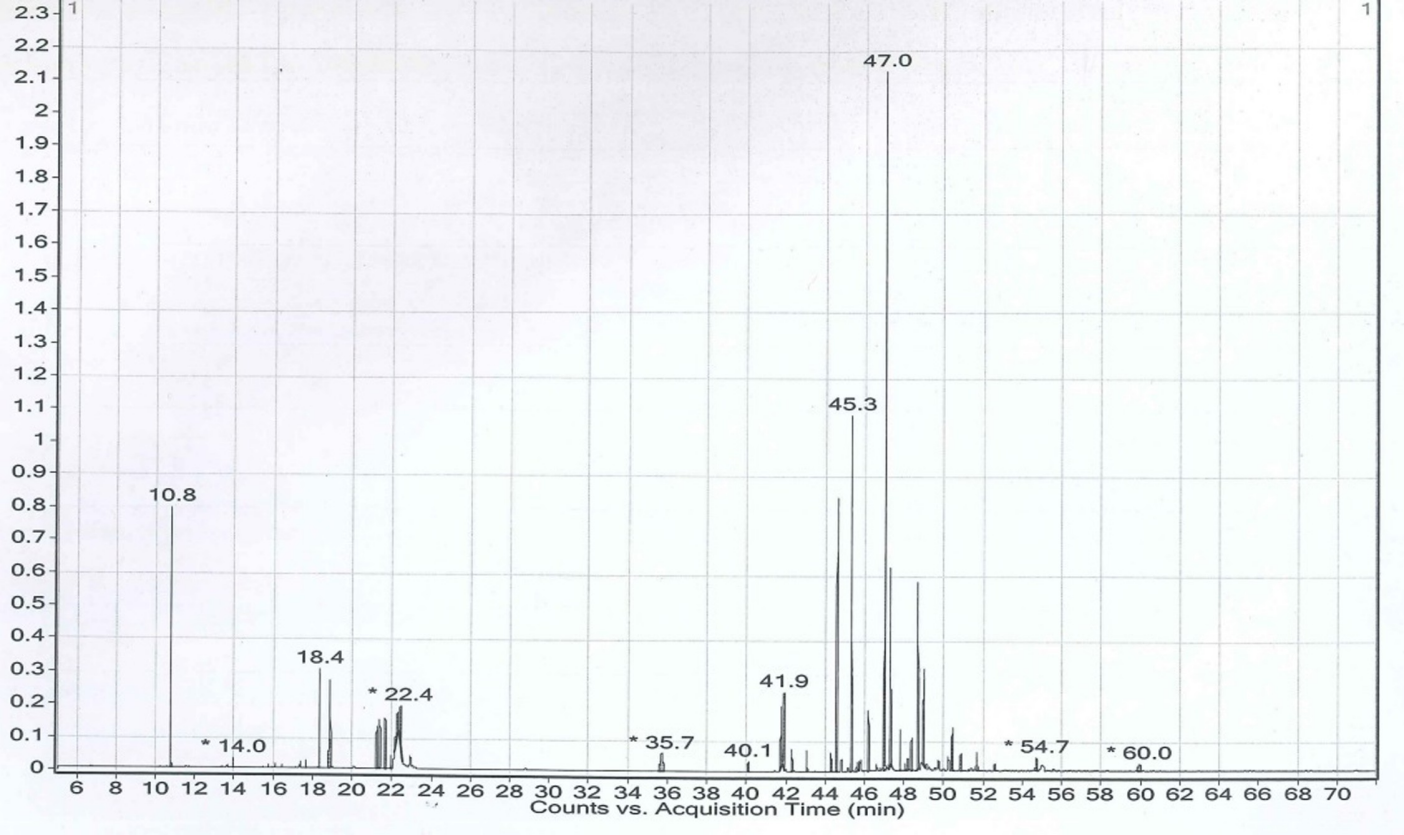
GC-MS analysis of *K. africana* n-hexane (HKA) extract.

The spectrum obtained from GC-MS analysis was interpreted and compared with the constituents kept in the NIST reference library. The criteria for identifying phytocompounds are based on their chemical name, retention time (RT), peak value, and molecular formula (MF). The percentage peak area is proportional to the number of components measured by relating the standard peak area to the total area and analyzing the compounds separated at different retention times according to their nature. These mass spectra are patterns of compounds that can be recognized from the data library. The phytochemical spectrum of ethanol and n-hexane extracts of the leaves of *K. africana* is illustrated in Figs 1 and 2. The GC-MS spectrum analysis of the ethanol and n-hexane extracts showed the existence of phytocompounds that strongly contribute to the medicinal properties of *K. africana*.

### Identification of phytoconstituents from *K. africana* extracts by GC-MS

GC-MS analysis identified the extracted components from EKA and HKA. The study reported the 24 and 40 chemicals that were found in both extracts (Figs 1 and 2). The extracts contain bioactive compounds including phenolic compounds, such as 2,4-di-tert-butylphenol. Terpenoids (2,6,10-trimethyl tetradecane, Propanoic acid, 3-(2,3,6-tri methyl-1,4-dioxaspiro[4.4]non-7-yl)-, methyl ester), terpenes (Dihydroactinidiolide), fatty acid esters (palmitic acid, 3,7,11,15-Tetramethyl-2-hexadecen-1-OL, Ethyl palmitate), alkenes (Linolenic acid), alkanes (Hexatriacontane, Nonacosane), volatile steroidal derivative (Ethyl iso-allocholate) and phytol were among the terpenoid derivatives that we obtained in this investigation. Volatile terpene (dihydroactinidiolide), and phytosterols such as stigmasterol, campesterol, clionasterol, and stigmast-4-en-3-one. While stigmasterol and clionasterol are thought to be anti-carcinogenic. This investigation also identified fatty acid methyl esters (Methyl linolenate) and phthalate esters (Dioctyl phthalate). The phytocompounds of *K. africana* were discovered together with their retention time (RT), molecular formula, molecular weight, and percentage area.

### *In vitro* analysis of plant extracts on cancer cell lines

#### Cytotoxic effect of *K. africana* extracts on MDA-MB-231 and HEK-293T cell lines

**Fig 3:**
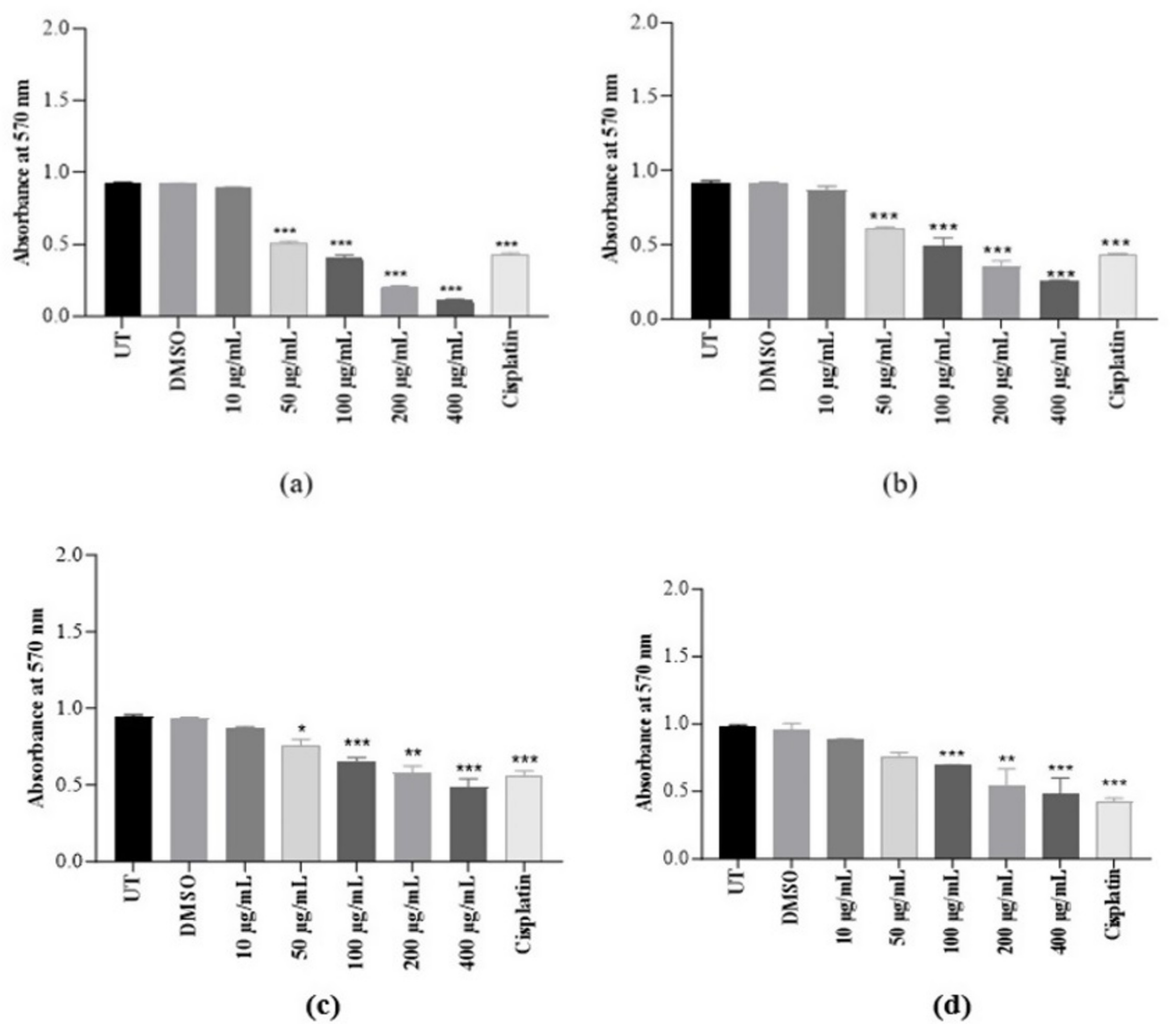
Cytotoxicity of KA extracts on MDA-MB-231 and HEK-293T cells. (A), EKA concentrations on MDA-MB-231 cells showed cellular inhibition at 72 hrs. (B), HKA concentrations on the MDA-MB-231 cells showed inhibitory effects. (C), The activity of EKA on the HEK-293T cell line showed mild effects only at higher concentrations. (D), HEK-293T cells showed minimal activity of HKA extract. Cisplatin had strong potential against both cell lines. All the experiments were compared to the study’s control (UT) and represented as mean and SD (*n* = 3). *p*-values, *p ˂ 0.05, **p ˂ 0.001, ***p ˂ 0.0001 denoted as statistically significant data. UT: Untreated; MTT: (3-(4,5-Dimethylthiazol-2-yl)-2,5-Diphenyltetrazolium Bromide); DMSO: Dimethyl sulfoxide; EKA: Ethanol extract of *K. africana*; HKA: n-hexane extract of *K. africana*.

The activity of EKA on breast cancer (MDA-MB-231) and non-cancer (HEK-293T) cell lines was determined through MTT assay after 72 hrs. of incubation (Fig 3). Different EKA and HKA extract concentrations (10, 50, 100, 200, and 400 μg/mL) were used to determine the cytotoxic effect.

The IC_**50**_ value of EKA extract on the MDA cell line obtained from the MTT assay was 20 μg/mL in comparison to HKA extract which is 48 μg/mL. We found a marked reduction in cell growth was analyzed in malignant cells (MDA-MB-231). However, the normal cells showed IC_**50**_ = 115 μg/mL which is marked as less toxicity against HEK-293T. On the other hand, HKA extract concentrations also exhibited cytotoxicity on breast cancer cells with decreased cell count and demonstrated enough potential against epithelial-like breast cancer with IC_**50**_ = 48 μg/mL but is less toxic against HEK-293T cells with IC_**50**_ = 158 μg/mL. In the case of conventional chemotherapeutic drugs, cisplatin has already been reported high cytotoxic effect in literature. The absorbance of the drug at 10 μg/mL exhibited strong antiproliferative activity on both cell lines and also proved to have more cytotoxicity at low doses toward healthy cells. Considering the DMSO (0.1%) cytotoxicity that was also evaluated through MTT assay indicated no influence on cells. However, we compared the mean absorbance values of all the experiments to the study’s control (UT) value which has no observable difference in MTT evaluation results, and represented them as mean and standard deviation. Our data showed significant results with *p-*value ≤ 0.05 (***).

#### Cell viability analysis of *K. africana* extracts on MDA-MB-231 and HEK-293T cell lines

**Fig 4:**
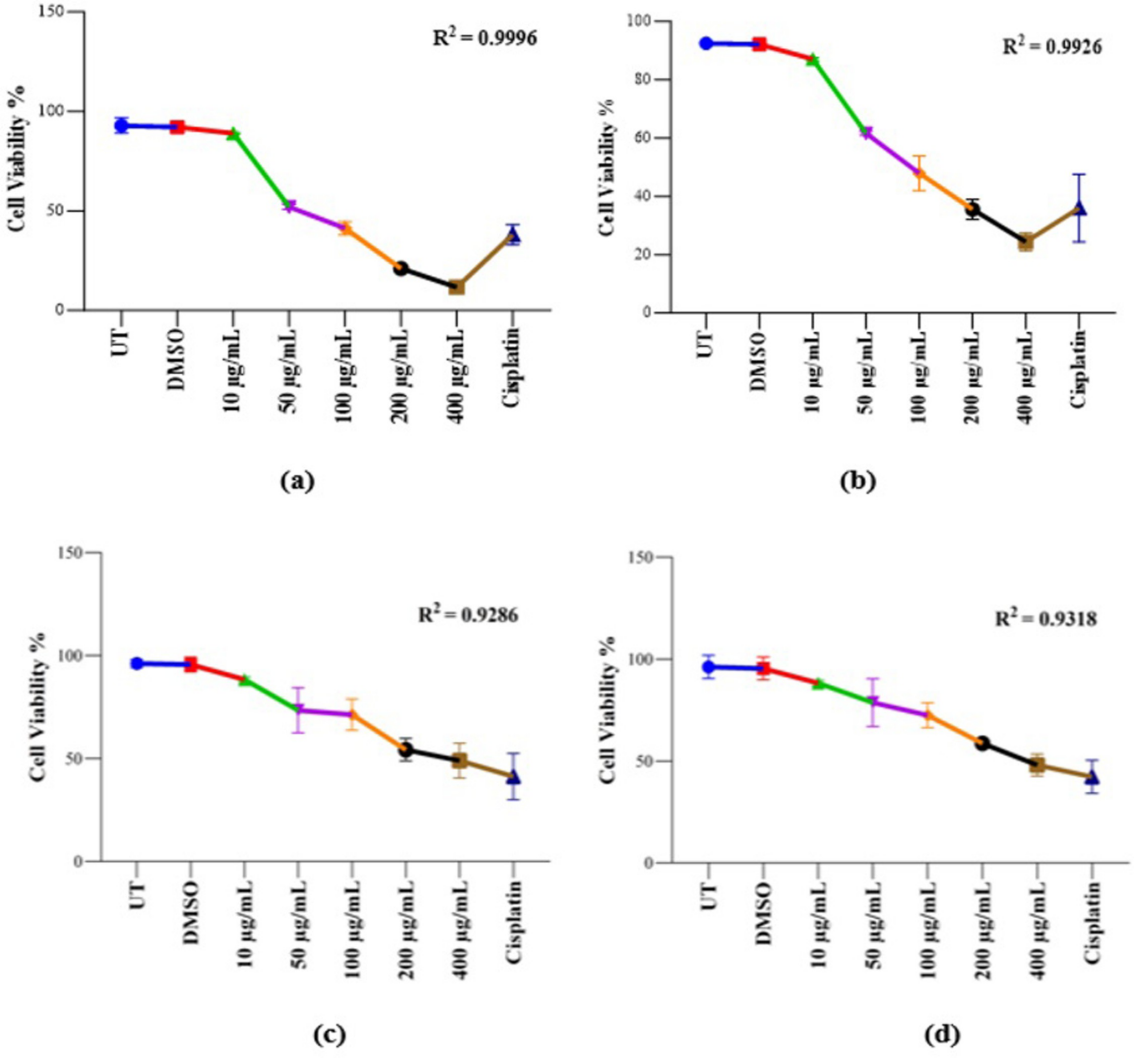
Cell viability analysis of KA extracts on MDA-MB-231 and HEK-293T cells. Cell viability was assessed by crystal violet (CV) assay. (A), EKA concentrations on MDA-MB-231 cells showed cellular inhibition at 72 hrs. (B), HKA concentrations on the MDA-MB-231 cells showed inhibitory effects. (C), The activity of EKA on the HEK-293T cell line showed mild effects only at higher concentrations. (D), HEK-293T cells showed minimal activity of HKA extract. Cisplatin had strong potential against both cell lines. All the experiments were compared to the study’s control (UT) and all the data values were represented as percentages (n = 3). R2 values fall between 0–1. p-values, *p ˂ 0.05, **p ˂ 0.001, ***p ˂ 0.0001 denoted as statistically significant data. UT: Untreated; DMSO: Dimethyl sulfoxide; EKA: Ethanol extract of *K. africana*; HKA: n-hexane extract of *K. africana*.

To investigate the possible cytotoxic effect on cancer (MDA-MB-231) and non-cancer (HEK-293T) cell lines and present them in percentage values, we conducted a dye-based cell viability assay (Fig 4). We found that crude extracts (EKA and HKA) exhibited a marked decrease in percentage proliferation in a dose-dependent manner. When comparing the calculated values of EKA and HKA extracts absorbance, both showed potent inhibition at all concentrations (10, 50, 100, 200, and 400 μg/mL) calculated as 96%, 56%, 44%, 22%, and 12% in MDA-MB-231 cells and HKA extract showed reduced viability significantly calculated as 94%, 66%, 51%, 38%, and 26%, while, a noticeable reduction was observed at 100, 200, and 400 μg/mL concentrations in the viability of MDA-MB-231. However, the observed percentage values of EKA extract concentrations in HEK-293T cells were recorded as 93%, 77%, 75%, 57%, and 52%, and HKA extract showed negligible effects on it as 92%, 83%, 76%, 62%, and 51%. As for the cisplatin anticancer drug, an observable percentage reduction in MDA-MB-231 was calculated as ˂ 35% and ˂ 45% in normal (HEK-293T) cells. Minimal inhibitory differences were noticeable at all concentrations of both extracts in the HEK-293T cell line. For the control (UT) of this study, the percentage values were calculated as 100% and its comparison suggested the strong interference of extract concentrations with the treated groups during the MTT assay. Therefore, the percentage values of DMSO (100%) showed no particular differences in the percentage of alive cells as in the untreated group. We also analyze the coefficient of determination (R^**2**^) in a regression model and verify differences among experimental data and standards. R^**2**^ values range between 0–1 and are stated as percentages, the standard R square values that are acceptable in science research must be 0.9 or above, so in this study, we calculated R^**2**^ as (a) = 0.9996, (b) = 0.9926, (c) = 0.9286, and (d) = 0.9318, and *p*-value = < 0.05 showed the significant results (Fig 4).

### Morphological assessment

#### Morphological changes in MDA-MB-231 and HEK-293T cells on exposure to *K. africana* ethanol extract

**Fig 5:**
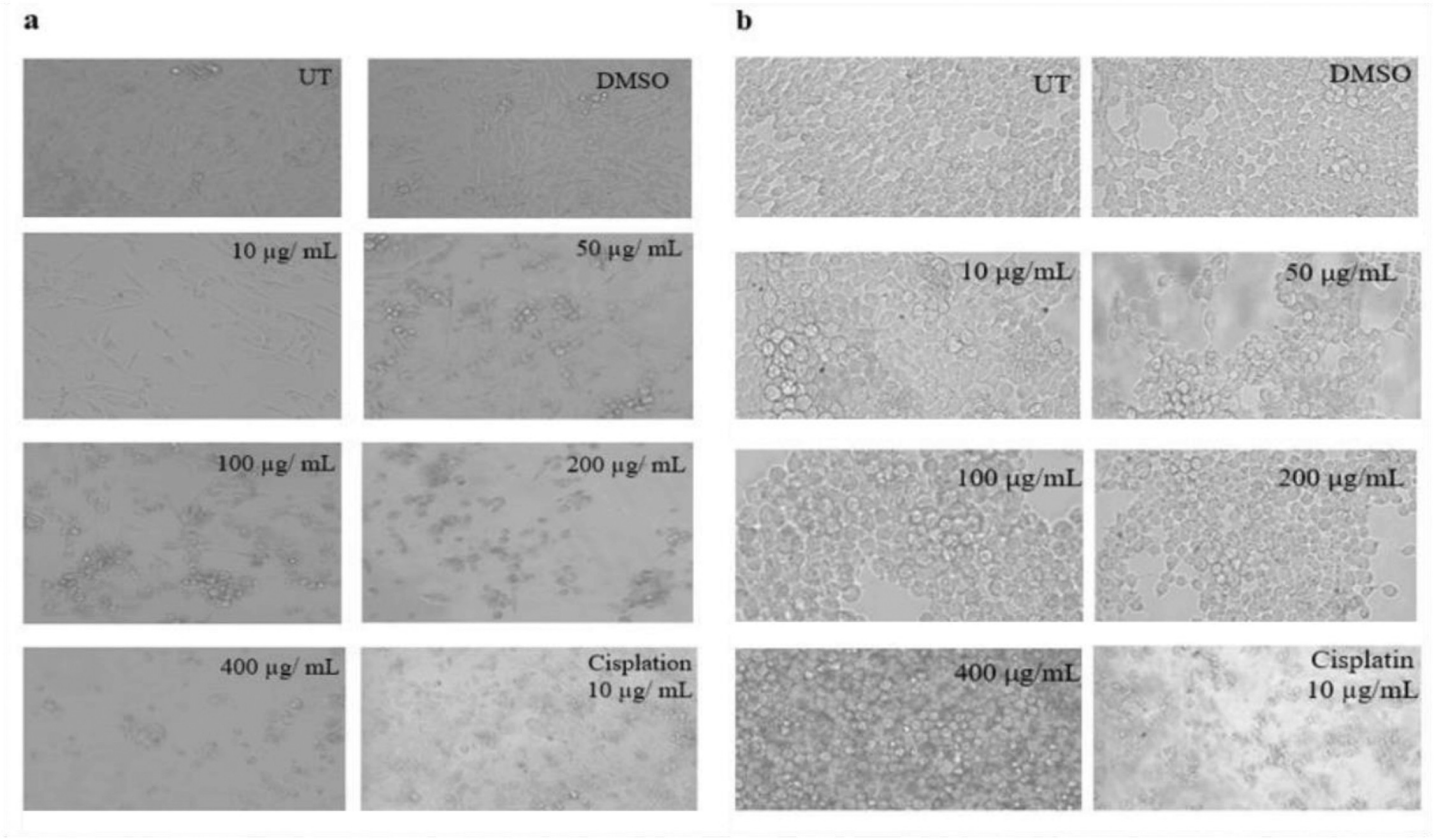
Morphological representation of MDA-MB-231 and HEK-293T cells exposed to various concentrations of EKA. (A), Alterations in MDA-MB-231 cancer cells were noticeable at concentrations ≥ 50 μg/mL at 72hrs. (B), Mild changes in HEK-293T cells were observed at 200, and 400 μg/mL. Positive control (Cisplatin) response revealed transformed cellular morphology in both cell lines. The scale bar was adjusted at 20x. UT: Untreated; DMSO: Dimethyl sulfoxide; EKA: Ethanol extract of *K. Africana*.

The morphological changes after treatment of EKA extract on breast cancer MDA-MB-231 and normal HEK-293T cell lines are shown in Fig 5. Noticeable changes have been observed, which include shrinkage of cells and reduction in their cell-to-matrix adhesion capacity, loss of shape, and reduced cell number in a dose-dependent way. Except for Untreated (UT) and DMSO-treated groups, over 12% of alive cells were calculated at 400 μg/mL. We noted a progressive decrease in morphological alterations, specifically at 100, 200, and 400 μg/mL. However, mild changes have been observed in the morphology of normal HEK-293T cells. Cisplatin at 10 μg/mL exhibited severe effects on both cell lines. MTT assay did not detect DNA changes as it is the basic cytotoxicity assay to evaluate the effect of the drug. Any drug/substance that causes cell shrinkage and loss of shape indicative of apoptosis as demonstrated in cancer cells only. Cisplatin shows the same pattern of cell death as our extract, might they both operate through similar mechanisms?

#### Morphological changes in MDA-MB-231 and HEK-293T cells on exposure to *K. africana* n-hexane extract

**Fig 6:**
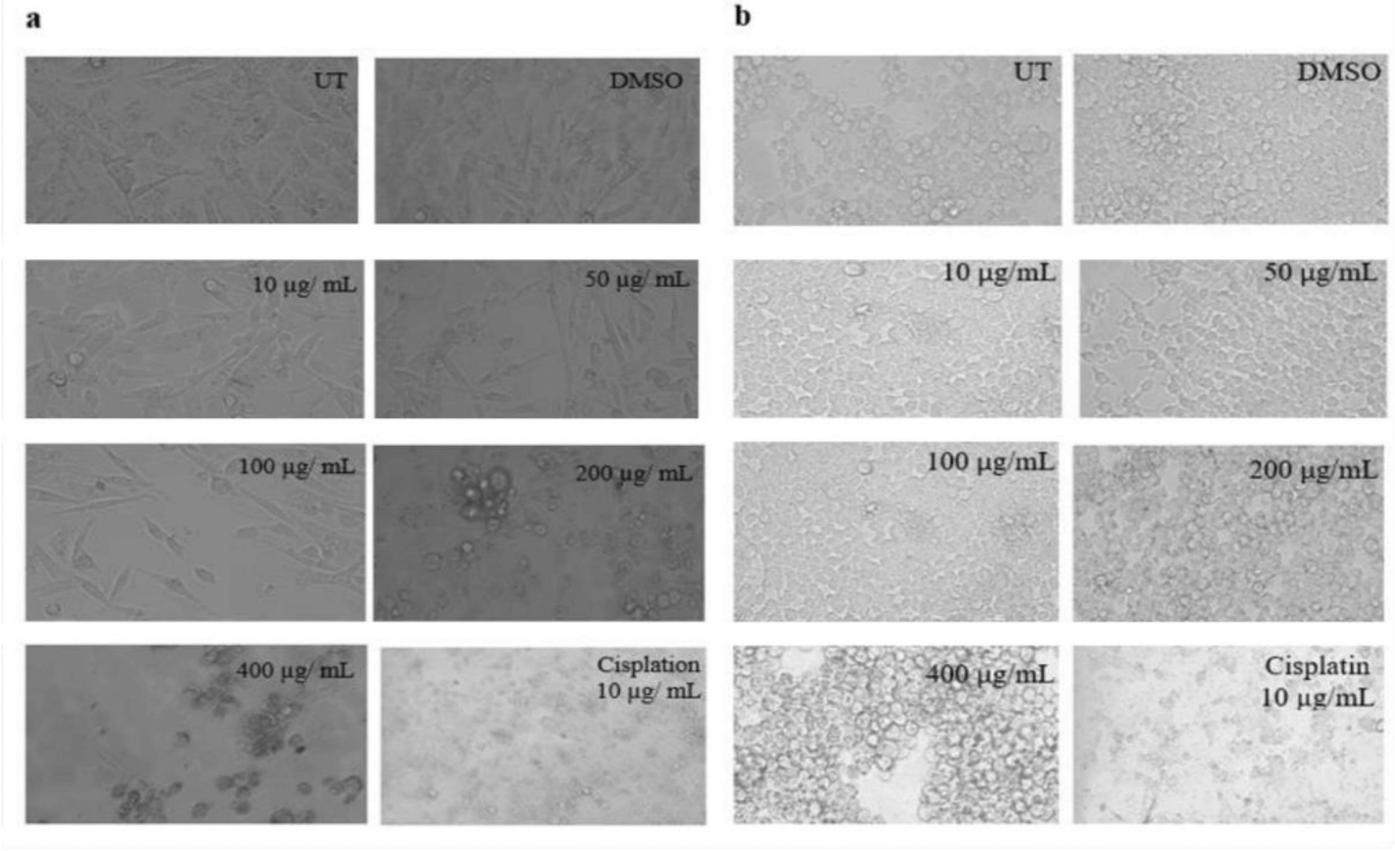
Morphological representation of MDA-MB-231 and HEK-293T cells exposed to various concentrations of HKA. (A), Alterations in MDA-MB-231 cancer cells were noticeable at concentrations ≥ 100 μg/mL at 72hrs. (B), Mild changes in HEK-293T cells were observed at 200, and 400 μg/mL. Positive control (Cisplatin) response revealed transformed cellular morphology in both cell lines. Cisplatin strongly affected both cell lines as growth arrested and detached cells. The scale bar was adjusted at 20x. UT: Untreated; DMSO: Dimethyl sulfoxide; HKA: n-hexane extract of *K. africana*.

The cancer cell cultures were inspected after 72 hrs. of HKA extract treatment and showed acceptable cellular changes in MDA-MB-231 cells. A decrease in cellular proliferation, formation of irregular cell aggregates, and transforming cells into clusters was observed at all concentrations when compared with the control (UT) cells. At low concentrations (10, 50, and 100 μg/mL), the morphology showed a reduced cell number when compared to the control group had healthy and confluent cells. Small clusters have been observed at 200 and 400 μg/mL with smaller cell body. No marked alterations in the morphology of normal HEK-293T cells were observed except at very high concentrations. However, the cisplatin (10 μg/mL) response exhibited a mutual decrease in the viability of cancerous and non-cancerous cells (Fig 6).

#### Cytotoxic effects of *K. africana* extracts on MCF-7 and HEK-293T cell lines

**Fig 7:**
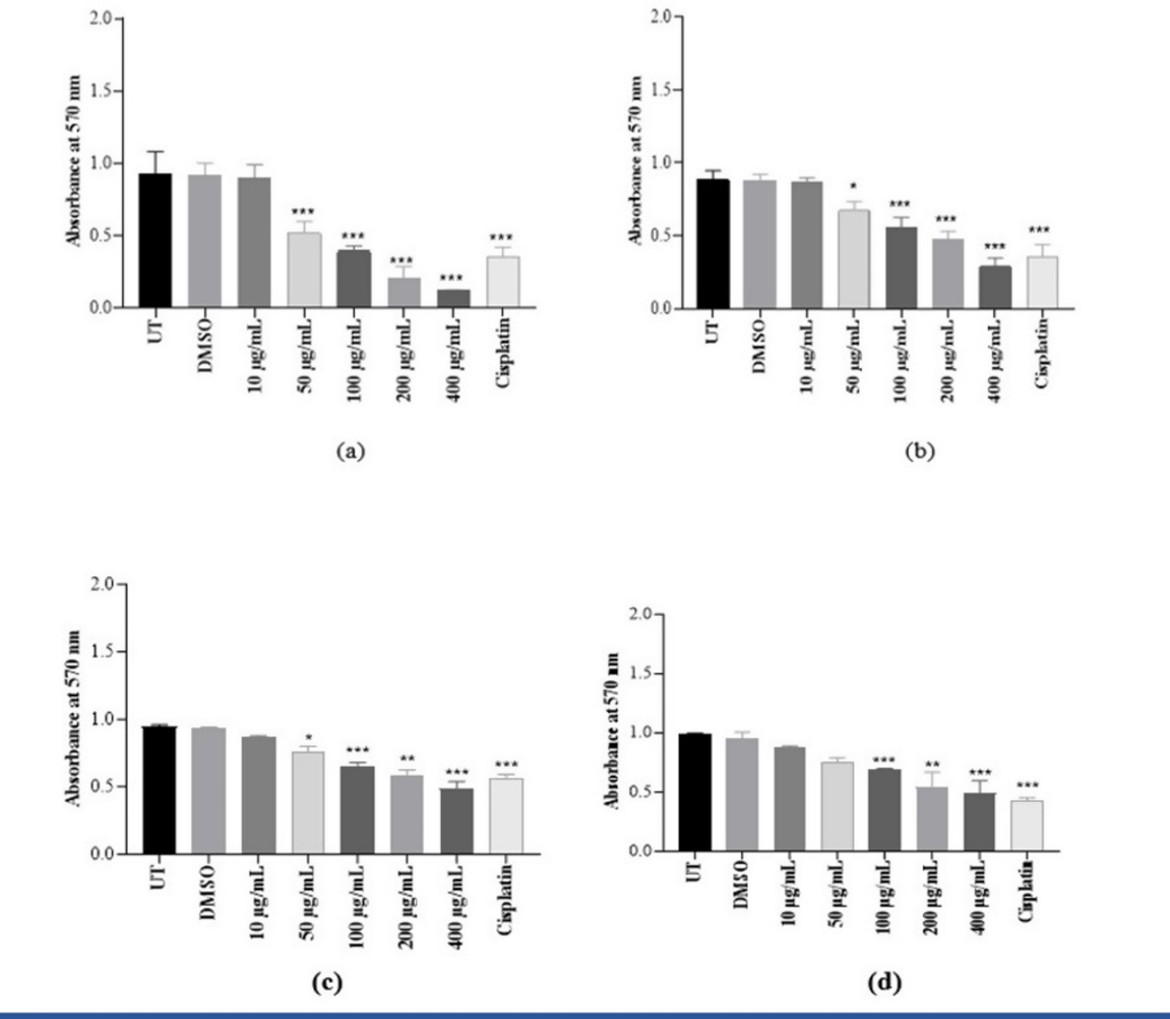
Cytotoxicity of KA extracts on MCF-7 and HEK-293T cells. (A), EKA concentrations on MCF-7 cells showed cellular inhibition at 72 hrs. (B), HKA concentrations on the MCF-7 cells showed inhibitory effects. (C), The activity of EKA on the HEK-293T cell line showed mild effects only at higher concentrations. (D), HEK-293T cells showed minimal activity of HKA extract. Cisplatin had strong potential against both cell lines. All the experiments were compared to the study’s control (UT) and represented as mean and SD (n = 3). p-values, *p ˂ 0.05, **p ˂ 0.001, ***p ˂ 0.0001 denoted as statistically significant data. UT: Untreated; DMSO: Dimethyl sulfoxide; EKA: Ethanol extract of *K. africana*; HKA: n-hexane extract of *K. Africana*.

MTT results for EKA and HKA extract (10, 50, 100, 200, and 400 μg/mL) doses revealed a dramatic decrease in MCF-7 cell proliferation at 72 hrs (Fig 7). The concentrations of bioactive plant extracts decreased the cell population depending on time and dose. The IC_**50**_ values of both extracts, EKA (IC_**50**_ = 32 μg/mL) and HKA (IC_**50**_ = 57 μg/mL) showed significant activity at 72 hrs. The actual decrease has been observed at 100, 200, and 400 μg/mL of EKA and HKA extracts. HEK-293T cells (EKA: IC_**50**_ = 115 μg/mL and HKA: IC_**50**_ = 158 μg/mL) represented a healthy control for comparing plant anticancer activity in cancer and noncancer cells. Both EKA and HKA extracts showed strong antiproliferative effects on cancer cells. Still, it also has a negligible effect on the HEK-293T cell line at 200 and 400 μg/mL concentrations as explained graphically by comparing it with the control (UT) group. The absorbance of the cisplatin drug at 10 μg/mL exhibited strong antiproliferative activity on both cell lines and also proved to have more cytotoxicity at low dose (10 μg/mL) toward healthy cells. Considering the DMSO (0.1%) cytotoxicity that was also evaluated through MTT assay indicated no influence on cells. However, we compared the mean absorbance values of all the experiments to the study’s control (UT) value which has no observable difference in MTT evaluation results, and represented them as mean and standard deviation. Our data showed significant results with *p-*value ≤ 0.05 (***). Therefore, we concluded that the assay may be suitable to confirm the general cytotoxicity of any drug.

#### Cell viability analysis of *K. africana* extracts on MCF-7 and HEK-293T cell lines

**Fig 8:**
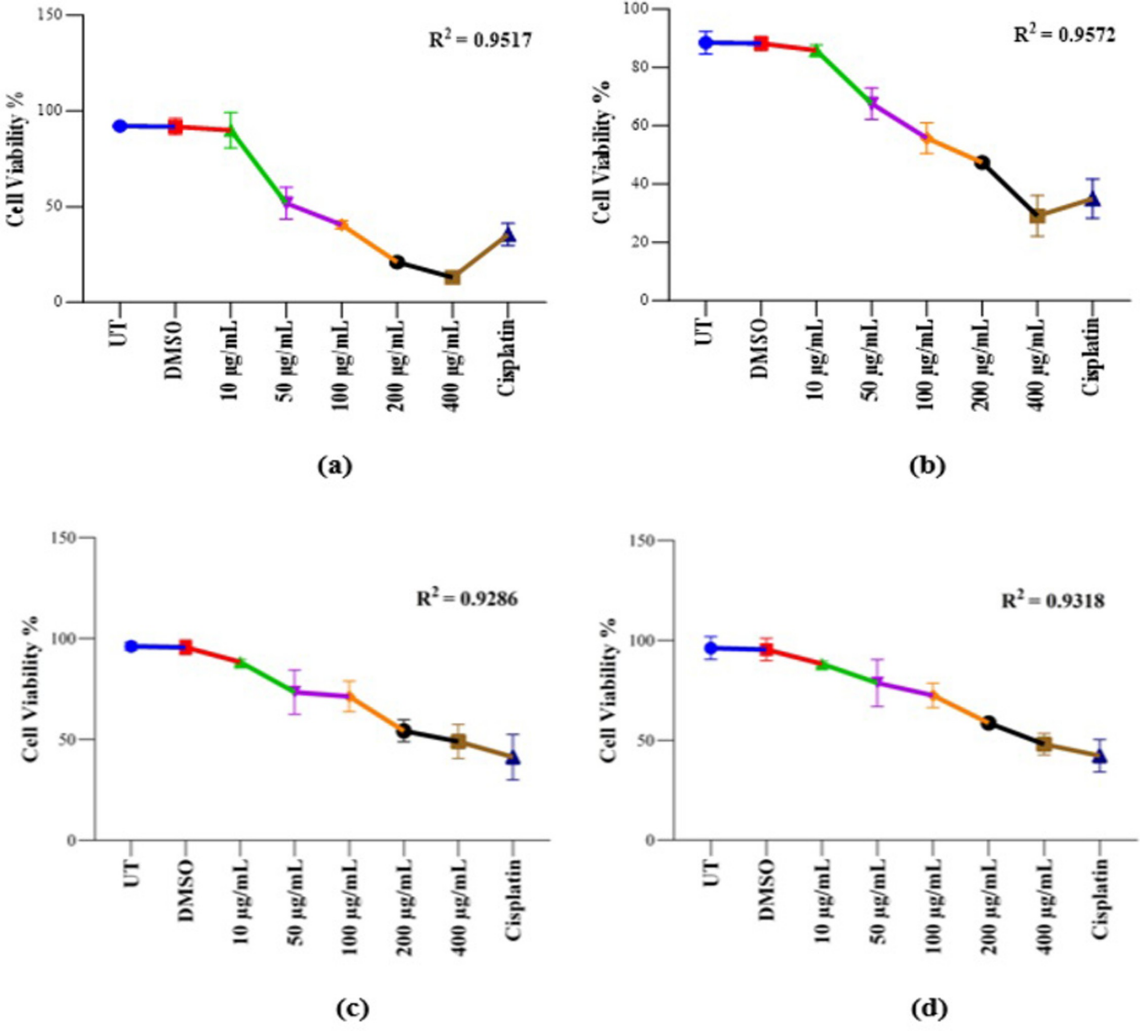
Cell viability analysis of KA extracts on MCF-7 and HEK-293T cells. (A), Cell viability was assessed by crystal violet (CV) assay. EKA concentrations on MCF-7 cells showed cellular inhibition at 72 hrs. (B), HKA concentrations on the MCF-7 cells showed inhibitory effects. (C), The activity of EKA on the HEK-293T cell line showed mild effects only at higher concentrations. (D), HEK-293T cells showed minimal activity of HKA extract. Cisplatin had strong potential against both cell lines. All the experiments were compared to the study’s control (UT) and all the data values were represented as percentages (n = 3). R2 values fall between 0–1. p-values, *p ˂ 0.05, **p ˂ 0.001, ***p ˂ 0.0001 denoted as statistically significant data. UT: Untreated; DMSO: Dimethyl sulfoxide; EKA: Ethanol extract of *K. africana*; HKA: n-hexane extract of *K. Africana*.

To observe the total percentage of cell viability, the crystal violet staining assay of various concentrations of both EKA and HKA extracts on MCF-7 and HEK-293T cell lines is summarized in Fig 8. A similar response was experienced in MCF-7 cells by crystal violet assay after different concentrations of both extracts as in the MTT assay. With increasing concentrations (10, 50, 100, 200, and 400 μg/mL), the cellular viability was reduced to 97%, 56%, 44%, 22%, and 14% for EKA as compared to 100% for untreated cells and 97%, 76%, 63%, 53%, and 32% for HKA extract as compared to 100% of UT control cells, respectively. Crystal violet assay revealed the HEK-293T cell viability recorded as 93%, 77%, 75%, 57%, and 52% for EKA and 92%, 83%, 76%, 62%, and 51% for HKA extract as compared to the 100% in control (UT) samples. Notably, the cisplatin strongly affected cancerous (MCF-7) and non-cancerous (HEK-293T) cell lines showed calculated percentages as ˂ 40% and ˂ 45%. As per calculations, reduced growth of breast cancer cells was observed. The difference in inhibition was not more noticeable at all concentrations of both extracts in the HEK-293T cell line.

For the control (UT) of this study, the percentage values were calculated as 100% and its comparison suggested the strong interference of extract concentrations with the treated groups during the MTT assay. Therefore, the percentage values of DMSO (100%) showed no particular differences in the percentage of alive cells as in the untreated group. We also analyze the coefficient of determination (R^**2**^) in a regression model and verify differences among experimental data and standards. R^**2**^ values range between 0–1 and are stated as percentages, the standard R square values that are acceptable in science research must be 0.9 or above, so in this study, we calculated R^**2**^ as (a) = 0.9996, (b) = 0.9926, (c) = 0.9286, and (d) = 0.9318, and *p*-value = < 0.05 showed the significant results (Fig 8).

### Morphological assessment

#### Morphological changes in MCF-7 and HEK-293T cells on exposure to *K. africana* ethanol extract

**Fig 9:**
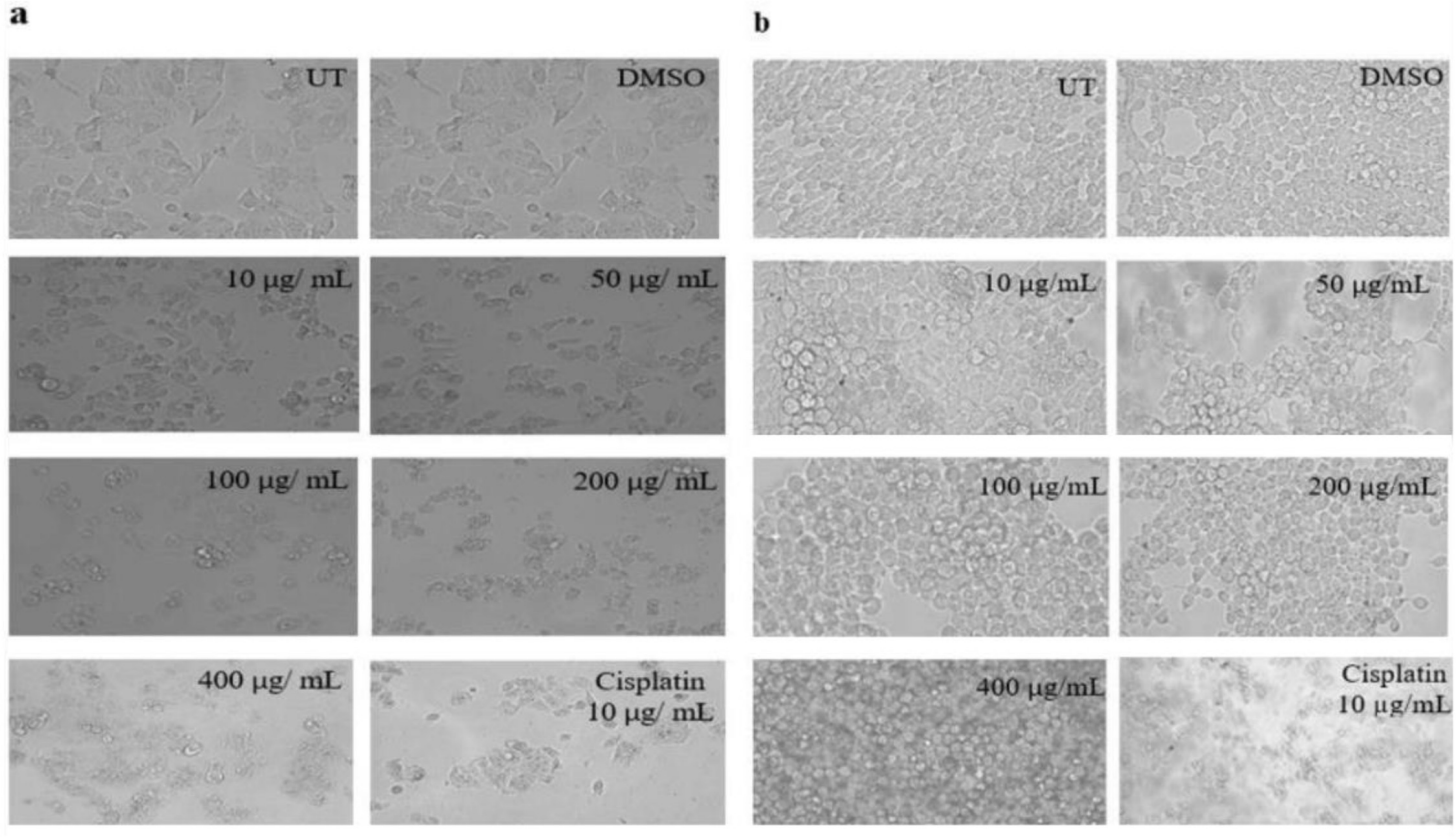
Morphological representation of MCF-7 and HEK-293T cells exposed to various concentrations of EKA. (A), Alterations in MCF-7 cancer cells were noticeable at concentrations ≥ 50 μg/mL at 72hrs. (B), Mild changes in HEK-293T cells were observed at 200, and 400 μg/mL. Positive control (Cisplatin) response revealed transformed cellular morphology in both cell lines. The scale bar was adjusted at 20x. UT: Untreated; DMSO: Dimethyl sulfoxide: EKA; Ethanol extract of *K. Africana*.

The cancer cell (MCF-7) morphology supported the EKA extract activity as shown in Fig 9. The Floid™ imaging station has revealed that MCF-7 cells lost their normal growth, proliferation, damaged membrane, and shrinkage. If we start discussing with the lowest concentration (10 μg/mL), we observe slight changes in the shape, size, and structure of cancer cells. The highest concentrations 200 and 400 μg/mL revealed the strong antiproliferative activity of extract on the receptor-based cell lines (MCF-7) and showed the formation of clusters and shrinkage. However, the morphology suggested mild effects on healthy cells compared to the control (UT) group. The growth and proliferation of cancerous and non-cancerous cells were observed to be suppressed when treated with cisplatin.

#### Morphological changes in MCF-7 and HEK-293T cells on exposure to *K. africana* n-hexane extract

**Fig 10:**
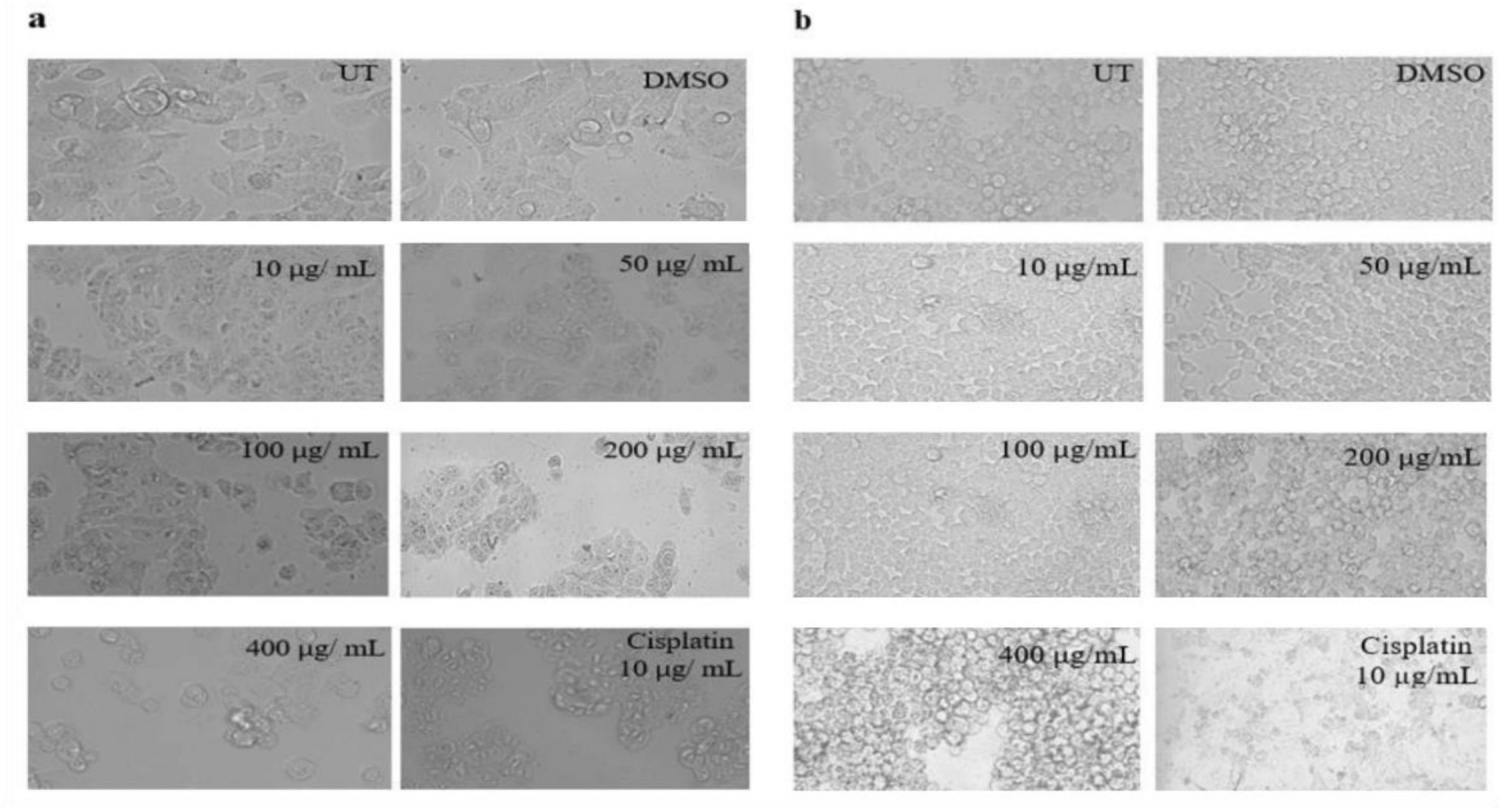
Morphological representation of MCF-7 and HEK-293T cells exposed to various concentrations of HKA. (A), Alterations in MCF-7 cancer cells were noticeable at concentrations ≥ 100 μg/mL at 72hrs. (B), Mild changes in HEK-293T cells were observed at 200, and 400 μg/mL. Positive control (Cisplatin) response revealed transformed cellular morphology in both cell lines. The scale bar was adjusted at 20x. UT: Untreated; DMSO: Dimethyl sulfoxide; HKA: n-hexane extract of *K. africana*.

Upon exposure to various concentrations of HKA extract, the morphological features of hormone-dependent MCF-7 and normal HEK-293T cells are presented in Fig 10. The untreated cells showed intact with their membrane, which suggests healthy proliferation. In contrast, breast cancer (MCF-7) cells lost their colony formation ability and a typical epithelial polygonal shape. After 72 hrs of treatment, the MCF-7 cells were contracted with a collapsed membrane and shape. The cancer cells treated with HKA extract at 10, 50, 100, 200, and 400 μg/mL concentrations showed significant differences compared to control media (UT). Fig 10 showed that HEK-293T cells significantly promoted proliferation and maintained their density with a mild effect. Cisplatin revealed cytotoxic activity on both cancer and non-cancer cell lines.

#### Effect of plant extracts on the mRNA expression levels of cancer-related genes

This study was considered to analyze the differential expression of oncogene (*BCL2*) and tumor-suppressor (*TP53*) genes in MDA-MB-231, and MCF-7 cancer cell lines for 200 ng of cDNA template per RT PCR reaction. Differences in fold change were observed after being treated with the IC_**50**_ (MDA = 20 μg/mL: MCF-7 = 32 μg/mL) values of ethanolic extracts. These values were compared with the control (UT) sample and endogenous control (*Hprt1*) levels, denoted as a housekeeping gene, to normalize the mRNA levels among samples. All the control (UT) and treated samples were analyzed by real-time PCR.

#### Effect of *K. africana* ethanol extract on the expression levels of *TP53* in MDA-MB-231 and MCF-7 cell lines

**Fig 11:**
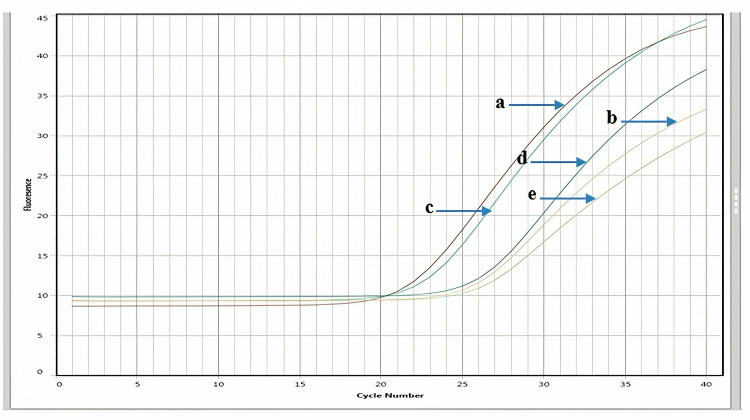
qPCR-based expression analysis of TP53 and Hprt1 genes in MDA-MB-231 and MCF-7 cells treated with *K. africana* (EKA) ethanol extract. (A), A PCR amplification curve representing high levels of TP53 in control (untreated) MDA-MB-231 cells. (B), A significantly downregulated expression of the TP53 gene in the ECA-treated MDA-MB-231cells. (C), A PCR amplification curve representing the overexpression of TP53 in control (untreated) MCF-7 cells. (D), A significantly downregulated expression of the TP53 gene in the ECA-treated MCF-7 cells. (E), The expression of the Hprt1 gene was used as a normalization control.

A difference in the mRNA expression levels of p53 in MDA-MB-231 and MCF-7 treated and control samples has been observed (Fig 11). PCR analysis evaluates the expression of TP53 at 72 hrs. of treatment. Focusing on the expression levels of the p53, the highest level with a fold change of 11.85 in triple-negative MDA-MB-231 cells and 4.98 in hormone-dependent MCF-7 cells was observed. The effect of IC_**50**_ concentration of EKA downregulates the expression of p53 in both cell lines. Fig 11 signifies a mean Ct value of 24.0 in treated and 20.1 in control samples of MDA-MB-231 cells. MCF-7 cells represented a mean Ct value of 22.35 and 20.0 in control samples after 72 hrs. of treatment. The housekeeping gene (*Hprt1*) showed an expression with a mean Ct value of 23–24 cycles in samples of both cell lines. Bar graph representation of fold change of *TP53* analyzed by real-time PCR presented in Fig 13.

#### Effect of *K. africana* ethanol extract on the expression levels of *BCL2* in MDA-MB-231 and MCF-7 cell lines

**Fig 12:**
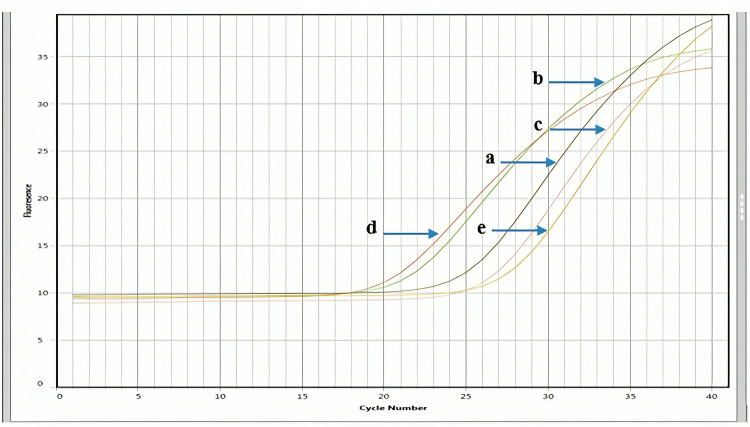
qPCR-based expression analysis of BCL2 and Hprt1 genes in MDA-MB-231 and MCF-7 cells treated with *K. africana (*EKA) ethanol extract. (A), A PCR amplification curve representing decreased BCL2 concentration in control (untreated) MDA-MB-231 cells. (B), A significantly upregulated expression of the BCL2 gene in the EKA-treated MDA-MB-231 cells. (C), A PCR amplification curve representing the downregulation of BCL2 in control (untreated) MCF-7 cells. (D), A significantly upregulated expression of the BCL2 gene in the EKA-treated MCF-7 cells; (E), The expression of the Hprt1 gene used as a normalization control.

**Fig 13:**
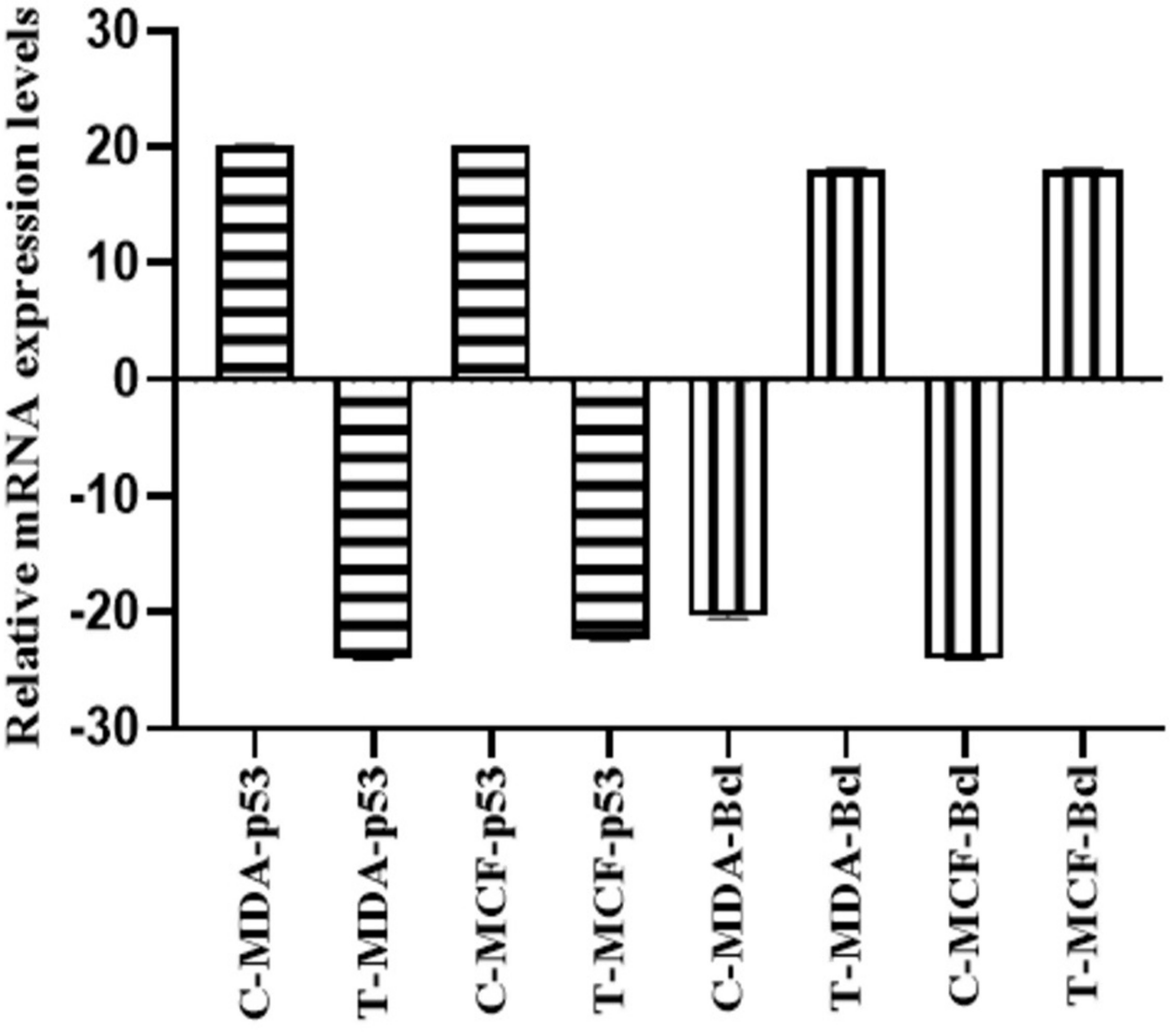
Graphical representation of mRNA expression analysis of TP53 and BCL2 genes in MDA-MB-231 and MCF-7 cell lines. The graph shows the regulation of TP53 and BCL2 in MDA-MB-231 and MCF-7-cells after EKA treatment compared with control samples (UT). Error bars show the statistically significant correlation among groups with p = ˂ 0.0001, R2 = 1.00 and n = 3; TP53: Tumor suppressor gene; BCL2: B-cell lymphoma 2 protein; MDA: MDA-MB-231 cell line; MCF: MCF-7 cell line.

The relative expression analysis of the *BCL2* gene and the therapeutic response of EKA has been examined in treated and control samples of breast cancer cell lines (Fig 12). The 20 μg/mL concentration of EKA extract was applied to MDA-MB-231, however, 32 μg/mL was given to MCF-7 cell culture. The low expression levels and the fold change of the *BCL2* gene in MDA-MB-231 (0.19) and MCF-7 (0.01) cell lines were observed. The mRNA levels of the control samples showed downregulation of *BCL2* with Ct values of 20.3 and 24.0 in both cell lines, respectively. The real-time analysis reveals that *BCL2* was preferentially expressed after treatment with calculated Ct values (18.1) and (18.0) in MDA-MB-231 and MCF-7 cell lines. The housekeeping gene (*Hprt 1*) was used as an endogenous control to justify the experimental modifications in the expression analysis of control and treated samples. The analysis revealed the expression of the reference gene in control and treated samples of both cell lines with a Ct value of 23 cycles. Bar graph representation of fold change of *BCL2* analyzed by real-time PCR presented in Fig 13.

#### Molecular docking

Drug-likeness and toxicological features are key determinants for the usage of ligands as drugs or medicines. As indicated in Table 4, using SwissADME currently, Lipinski’s rule to estimate the drug-likeliness of small molecules was achieved. Lipinski’s rule states that an orally active medication satisfies the requirements if it has no more than one violation of the criterion, and should be satisfied by compounds that weigh less than < 500 g/mol. Therefore, we concluded that as per Lipinski’s rule, the potential phytochemicals qualify as orally active drugs. After the prediction analyses, molecular docking analysis was used to dock the compounds with the designated apoptotic protein.

**Table 4:** Physio-chemical parameters of ligand molecules for Lipinski’s rule.

Compounds	MW (g/ mol)	HBA	HBD	MLog *P*_o/w_	MR	NRB	L.V	Muta- genicity	Carcino-genicity	AOT
Propanoic acid, 3-(2,3,6-trimethyl-1,4-dioxaspiro[4.4]non-7-yl)-, methyl ester	256	4	0	2.49	68.8	4	Yes, 0	None	None	III
Palmitic acid	256	2	1	5.2	80.80	2	Yes, 1	None	None	IV
Linolenic acid	278	2	1	5.09	88.99	13	Yes,1	None	None	III

**MW** = Molecular weight: **MR** = Molar refractivity: **HBA** = Hydrogen bond acceptor: **HBD** = Hydrogen bond donor: **MLog *P***_**o/w**_ = Lipophilicity: **LV** = Lipinski’s violation: **NRB** = No. of rotatable bonds: **AOT** = Acute oral toxicity

#### Molecular interaction analysis

High binding affinities and strong interactions were demonstrated by the low binding energies in between pairs. For molecular docking studies, we used AutoDock 4.2.6 to interact with targeted proteins (Bcl-2, EGFR, HER2, and TP53). Four target proteins were the subject of all four interactions. On interaction with selected compounds, the screened chemicals from KA extracts showed acceptable results. Good binding affinity was shown by TP53/ Propanoic acid, 3-(2,3,6-trimethyl-1,4-dioxaspiro-[4.4]-non-7-yl)-, methyl ester demonstrated good binding affinity (-7.1 kcal/mol); conversely, BCL2/Propanoic acid, 3-(2,3,6-trimethyl-1,4-dioxaspiro[4.4]non-7-yl)-, methyl ester showed -6.4 kcal/mol of interaction score. The computed binding score for the docked EGFR/Linolenic acid was -6.7 kcal/mol. The binding energy of HER2/Palmitic acid was -5.0 kcal/mol which is low yet within the acceptable range. All the docked complexes had RMSD values measured at 0.000 Å, with a value of ≤ 2 Å which is considered to be fairly acceptable. Table 5 contains the binding energies in kcal/mol and the binding affinities of these molecules.

**Table 5:** Binding affinity of ligand molecules with receptors.

Complexes	ΔG (kcal/mol)	Rmsd lb	Rmsd ub
**TP53|**Propanoic acid, 3-(2,3,6-trimethyl-1,4-dioxaspiro[4.4]non-7-yl)-, methyl ester	-7.1	0.000	0.000
**Bcl-2|**Propanoic acid, 3-(2,3,6-trimethyl-1,4-dioxaspiro[4.4]non-7-yl)-, methyl ester	-6.4	0.000	0.000
**EGFR|**Linolenic acid	-6.7	0.000	0.000
**HER2|**Palmitic acid	-5.0	0.000	0.000

**RMSD** = Root mean square deviation: **RMSD/lb** = Rmsd lower bond: **RMSD/ub** = Rmsd upper bond

#### Amino acid interactions with phytocompounds from *K. africana* extracts

**Fig 14:**
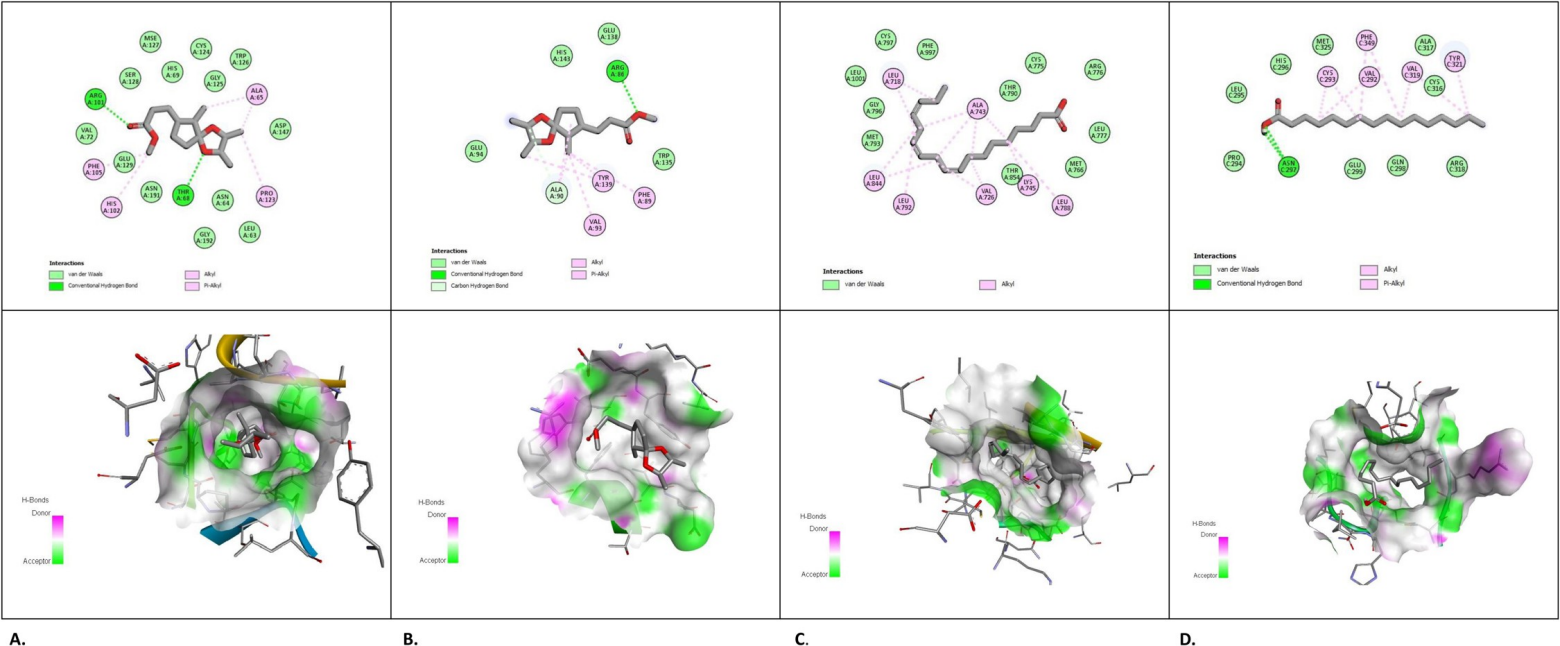
2D and 3D docking interpretations with target proteins. **(A)**, P53/ Propanoic acid, 3-(2,3,6-trimethyl-1,4-dioxaspiro[4.4]non-7-yl)-, methyl ester, (PDB ID: 2OCJ); **(B),** BCL2/ Propanoic acid, 3-(2,3,6-trimethyl-1,4-dioxaspiro[4.4]non-7-yl)-, methyl ester (PDB ID: 2W3L); **(C),** EGFR/Linolenic acid (PDB ID: 3poz); **(D),** HER2/ Palmitic acid (PDB ID: 1n8z).

The phytochemicals that were present in the KA extracts were subjected to molecular docking studies with their target proteins. The selected ligands demonstrated high binding energies towards BCL2, TP53, EGFR, and HER2. The vina score of P53/Propanoic acid, 3-(2,3,6-trimethyl-1,4-dioxaspiro[4.4]non-7-yl)-, methyl ester exhibited the best docking confirmation with a binding affinity in these top four assessments. Fig 14 reveals the molecular docking schematic process and Table 6 lists the binding interaction score (kcal/mol). Hydrogen bonds are necessary for the drug-protein interaction and the structural stability of a vast range of biological reactions. The angle of engagement, acceptor and donor atoms determine the strength of a hydrogen bond. Propanoic acid, 3-(2,3,6-trimethyl-1,4-dioxaspiro[4.4]non-7-yl)-, methyl ester was found to be the most promising phytochemical in terms of its interaction with the target tumour suppressor protein P53; there are five hydrophobic interactions with the amino acids Thr68, Phe105, Pro123, Glu129, and Asn191, and two hydrogen bonds with amino acid residues of Thr68, and Arg101. Furthermore, it demonstrated a notable affinity towards the Bcl-2 protein, forming a hydrogen bond with the amino acid residue Arg86. Seven hydrophobic bonds with Leu718, Val726, Ala743, Lys745, Leu788, Thr790, and Leu844 active site residues confer the binding characteristics of linolenic acid to EGFR tyrosine kinase. Similarly, seven hydrophobic interactions with Val292, Gln298, Glu299, Arg318, Val319, Tyr321, and Phe349, and one hydrogen bond with Asn297 are present between palmitic acid and HER2. Fig 14 displays the interactions between the selected phytochemicals and individual therapeutic targets, as well as their active sites.

**Table 6:** The residual interaction of proteins with plant components.

Complexes	Hydrogen interactions		Hydrophobic interactions	
**TP53|**Propanoic acid, 3-(2,3,6-trimethyl-1,4-dioxaspiro [4.4]non-7-yl)-, methyl ester	**Amino acids**	**Distance (Å)**	**Amino acids**	**Distance (Å)**
	THR68, ARG101	2.08,1.97	THR68, PHE105 PRO123, GLU129 ASN191	3.20,3.99,3.67,3.79,3.89
**Bcl-2|**Propanoic acid, 3-(2,3,6-trimethyl-1,4-dioxaspiro[4.4] non-7-yl)-, methyl ester	ARG86	3.17	VAL93 TYR139	3.73, 3.78
**EGFR|**Linolenic acid	-	-	LEU718,VAL726 ALA743, LYS745 LEU788, THR790 LEU844	3.59,3.76,3.48,3.40,3.85,3.47,3.92
**HER2|**Palmitic acid	ASN297	1.88	VAL292,GLN298GLU299, ARG318 VAL319, TYR321 PHE349	3.73,3.76,3.66,3.80,3.76,3.72,3.48

*Assessment of in silico ADMET prediction*. Using the AdmetSAR program, we determined the toxicity profiles of the phytocompounds. According to the previous literature, the Ames is an important test and positive results indicate a mutagenic substance, while negative results indicate a lack of mutagenicity. All the chemicals displayed a negative number showing non-mutagenicity. It was applied to these compounds to evaluate the level of their toxicity as low, moderate, or high depending on their irritant and mutagenic effects. Table 7 displays the comprehensive ADMET analysis of the top compounds with the greatest hits. All the compounds isolated from KA extracts showed moderate solubility in water and high absorption in the human gut; the compounds include linolenic acid, palmitic acid, and propanoic acid, 3-(2,3,6-trimethyl-1,4-dioxaspiro[4.4]non-7-yl)-, methyl ester. All the compounds tested negative for the P-gp substrate. All the selected chemicals demonstrated higher BBB parameters as well as higher GI absorption which are both necessary for drug absorption in the human body. CYP1A2 and CYP3A4 seem to be involved in the metabolism of linolenic acid in the liver. Nevertheless, none of the phytocompounds inhibited the activities of CYP2C19, CYP2C9, and CYP2D6 enzymes. All substances were non-Ames toxic or hepatotoxic except linolenic acid which demonstrated low hepatotoxicity. In terms of clearance values from the plasma, all the phytocompounds confirmed high values. These popular chemicals have a lot of promise for use as safe medication for both human and animal systems.

**Table 7:** ADMET profile of screened compounds.

Pharmacokinetic parameters	C I	C II	C III
Water solubility	-2.69	-5.562	-5.78
Caco-permeability	0.593	1.55	1.55
HIA	0.760	0.988	0.989
P-glycoprotein substrate	No	No	No
Blood-brain barrier	0.947	0.948	0.931
CNS permeability	-2.94	-1.81	-1.54
GI absorption	Yes	Yes	Yes
Skin permeability	-2.88	-2.71	-2.72
Skin sensitization	Yes	Yes	Yes
Subcellular localization	Mitochondria	Mitochondria	Plasma membrane
Inhibitor of CYP1A2	None	None	Yes
Inhibitor of CYP2C19	None	None	None
Inhibitor of CYP2C9	None	None	None
Inhibitor of CYP2D6	None	None	None
Inhibitor of CYP3A4	None	None	Yes
AMES toxicity	No	No	No
Hepatotoxicity	No	No	Yes
Rat oral toxicity (LD50)	2.15	1.44	1.44
Chronic rat toxicity	1.675	3.131	3.11
Tolerated dose in human	0.421	-0.708	-0.84
Drug plasma clearance	1.23	1.763	1.99
*T. pyriformis* toxicity	0.392	0.84	0.72
Minnow toxicity	1.421	-1.08	-1.18
hERG I inhibitor	No	No	No
hERG II inhibitor	No	No	No
OCT2 Renal substrate	No	No	No

**HIA =** Human Intestinal absorption: **GI =** Gastrointestinal: **CNS =** Central nervous system: C = Compound:

**C I =** Propanoic acid, 3-(2,3,6-trimethyl-1,4-dioxaspiro[4.4]non-7-yl)-, methyl ester**: C II =** Palmitic acid: **C III =** Linolenic acid:

## Discussion

At present, more than half of the medicines originate from plants. The application of phytomedicines has increased significantly in the past few years, due to their enhanced therapeutic efficacy and comparatively fewer adverse effects than allopathic medications. Phytomedicines have shown exceptional activity *in vitro*, however, *in vivo* is limited owing to their improper molecular size, limited hydrophilicity, and lipophilicity, which limit their absorption. A deeper understanding of the pharmacokinetics of phytomedicine can address some of these issues, while also enabling the design of appropriate dosage regimens [31]. Secondary metabolites present in this genus (KA) have been explored for their biological potential. However, to the best of our knowledge, the molecular docking of anticancer target proteins with components of KA and the gene expression study of these proteins related to the growth of breast tumours remain uninvestigated. The apoptotic, cytotoxic, and antioxidant abilities of medicinal plants have been reported by numerous research studies [32]. Owing to their minimal adverse effects, medicinal plants provide a great substitute for novel therapeutic agents [33]. As a result, alternative natural therapeutic approaches for breast cancer are the focus of the current study. Therefore, inflammation-induced cancer has more recently been often managed and treated with medicinal plants as they contain a diverse range of biologically active phytocompounds.

According to our understanding, both extracts showed the presence of alkaloids, flavonoids, glycosides, terpenoids, fatty acid esters, phytosterols, and phenols (Figs 1 and 2). The phytochemical study of EKA and HKA extracts demonstrated their active ingredients, and the topmost reported here with a high area percentage and biological activities. Phytol (40.37%), is a diterpene containing an essential fatty acid associated with antibacterial, anticancer, antidiuretic, anti-inflammatory, antioxidant, and neuroprotective properties [34]. Linolenic acid (17.11%), is known to induce apoptosis and is hypothesized to prevent cancer cell metastasis and survival [35], and palmitic acid (11.33%) from EKA extract, which acts as 5- an alpha-reductase inhibitor involved in developing prostate cancer (CaP) and also demonstrates antimicrobial, anti-fibrinolytic, anti-inflammatory, antioxidant, hemolytic, metabolic, and antitumor activities in several types of tumors [36]. Hentriacontane (21.12%) reduces the concentrations of mediators that cause inflammation (COX-2, iNOS, ILs, PGE, and NF-κB) and activates the caspase-1 and NF-κB in peritoneal macrophages that LPS has stimulated [37]. Octacosane (9.54%) is a long-chain alkane reported to have a cytotoxic effect against the melanoma B16F10-Nex2 cancer cell line [38]. (E, E, E, E)-Squalene (7.56%) has demonstrated effective inhibitory activity in chemically induced colon, lung, and skin tumorigenesis [39] from HKA extract were the primary constituents with higher area percentages. In small proportions, the residual compounds exist. Flavonoids have been proven operative against malignant cells by deactivating or blocking carcinogens. Additionally, they exhibited antiproliferative activity, and apoptosis induction, and caused cell cycle arrest [40]. Terpenoids are therapeutically active and a class of phytochemicals with high anticancer activity by preventing cell differentiation, inducing apoptosis, and reducing inflammation [41]. Similarly, alkaloids and saponins have been indicated to have anticancer activity towards cancer. Moreover, saponins discourage cellular proliferation and enhance apoptosis [42]. So, we can say that these highlighted compounds might be involved in regulating apoptosis in breast cancer cells.

Consequently, more studies are required to investigate the naturally existing compounds present in the EKA and HKA extracts as they may be potential biologically and pharmacologically active agents. Currently, drug development involves the widespread use of bioinformatics technologies for prediction analyses of drug-like biologically active molecules. The therapeutic benefits of phytochemicals were predicted and further validated by *in silico* and *in vitro* studies. Many studies have demonstrated the anticancer effect of therapeutic plants that constitute numerous naturally occurring phytocompounds. Though there are several anticancer mechanisms by which these phytocompounds operate, the most common is the induction of apoptosis [27]. In the present study, we observed the cytotoxic effects of KA extracts in a concentration-dependent manner against HEK-293T, MCF-7, and MDA cells using the MTT assay. Additionally, a crystal violet assay was employed to confirm these results.

Current studies revealed different cellular responses associated with varying EKA and HKA doses (10, 50, 100, 200, and 400 μg/mL). A pronounced cell growth reduction was started at 50 μg/mL of EKA extract with an IC_**50**_ concentration of 20 μg/mL in MDA triple-negative breast cancer cells has been observed. The cell population of malignant cells decreased noticeably, but these alterations showed less toxicity (IC_**50**_ = 115 μg/mL) in healthy (HEK-293T) cells (Fig 3). It can be concluded that the HKA extract also had enough potential against epithelial-like breast (MDA-MB-231) cancer with IC_**50**_ = 48 μg/mL. This extract proposes general toxicity in the HEK-293T cell line (IC_**50**_ = 158 μg/mL). Earlier researchers have documented the *in vitro* cytotoxic properties of dichloromethane and methanol extracts of the *K. africana* stem against human breast cancer cells exhibited strong activity of alcoholic extract (IC_**50**_ = 26.02 μg/mL) [43].

Different concentrations corresponding to IC_**50**_ values of 32 and 57 μg/mL for EKA and HKA were applied to MCF-7 cell lines, respectively. MTT assay performs quantitative analysis of the dose-effect relationship of multiple concentrations. HEK-293T cells (EKA: IC_**50**_ = 115 μg/mL and HKA: IC_**50**_ **=** 158 μg/mL) represent healthy control for comparing plant anticancer activity in cancerous and healthy cells (Fig 7). The extracts seem to significantly impact the cancer cell line, as indicated by the concentration value of the co-treatment. The fruit extract concentrations of *K. africana* for cytotoxicity against two breast and skin cancer cell lines are shown in one study. According to the study, some of the phytochemicals like dimethyl kigelin, 2-(1-hydroxyethyl)-naphtho [2,3-b] furan-4,9-dione, and ferulic acid, have been confirmed as the constituents believed to be accountable for having cytotoxic effects. From these, 2-(1-hydroxyethyl)-naphtho [2,3-b] furan-4,9-dione was the principal agent for the antiproliferative activity of cancer cells [44].

The primary goal of tumor treatment is to specifically target cancer cells while leaving the healthy cells unaffected which is the main constraint of chemotherapeutic agents. In the current study, it was observed that extracts from both plants exhibited cytotoxic activity against breast cancer cells, without destroying the healthy cells, conversely, cisplatin which is a platinum-based antitumor medication affects equally both cancerous and non-cancerous cells by inducing apoptosis. The underlying mechanism of cisplatin-induced cytotoxicity is caused by the formation of intra-strand and inter-strand crosslinks in DNA, which thereby obstruct DNA replication and transcription. The damaged DNA induces signalling pathways that eventually result in the apoptosis of affected cells. Due to the frequently dysregulated cell cycle checkpoints and repair mechanisms, cancer cells terminate the induction of apoptosis [45]. Thus, this plant may be able to treat breast cancer without disrupting normal cells.

Similarly, crystal violet analysis was performed following the treatment with both extracts (EKA and HKA) on cancer MDA-MB-231, MCF-7, and normal HEK-293T cells (Figs 4 and 8). Our study showed that both extracts exhibited a pronounced decrease in percentage depending on the concentrations used. Cell proliferation was significantly reduced at 100, 200, and 400 μg/mL concentrations with 44%, 22%, and 12% for EKA, and 51%, 38%, and 26% for HKA extract in MDA-MB-231 cells. The results demonstrated the decreased cell viability of MCF-7 as 44%, 22%, and 14% for EKA, and 63%, 53%, and 32% for HKA extract. The percentage viability of HEK-293T cells was recorded as 75%, 57%, and 52% for EKA, and 76%, 62%, and 51% for HKA extract at 100, 200, and 400 μg/mL showed less cytotoxicity of extracts. In 2012, Chivandi and associates explored the outcome of seed oil retrieved from *K. africana* on the proliferation of human embryonic kidney (HEK-293T), and human colon adenocarcinoma (Caco-2) cells in the culture medium. The concentrations used for treating cells at 0, 20, 40, 80, 100, and 120 mg/L of seed oil. The effect of seed oil showed an overall decrease in cancer (Caco-2) cells with increasing concentrations, while it significantly suppressed HEK-293T cells at higher concentrations [46]. The current study observed noticeable changes in the shrinkage of cells and their adhesion capacity, loss of shape, and reduced cell number at 100, 200, and 400 μg/mL concentrations in both cell lines (Figs 5, 6, 9 and 10). In contrast to extracts, cisplatin exhibited severe effects on all cell lines. One study revealed the cytotoxicity of eupatorine isolate from *Eupatorium semiserratum* on MDA-MB-231 and MCF-7 cell lines at 72 hrs. The morphological changes observed by phase contrast microscopy showed polygonal/trigonal shape, shrunken cells, and lost microvilli in cancer cell lines [47].

This study was proposed to analyze the differential expression of oncogenes in breast (MDA-MB-231, and MCF-7) cancer cells. The choice of cancer-associated proteins and their corresponding genes including *BCL2*, and *TP53*, in research may be affected by various factors, as both these genes are known to play crucial roles in cancer pathogenesis. For instance, due to the role TP53 plays in controlling cell cycle, cell death, and DNA repair, TP53 is frequently referred to as the “guardian of the genome”. Likewise, BCL2 is known for its function as an anti-apoptotic protein as it prevents apoptosis and thereby, promotes cancer cell survival. These genes and proteins are clinically significant in cancer diagnosis, prognosis, and treatment. Research on how these genes and proteins are expressed and regulated may provide important insights into cancer biology and thereby aid the development of advanced diagnostic and therapeutic options [48]. It is not reported earlier in the literature whether EKA extract regulates the mRNA expression of *TP53* and *BCL2* genes or not. In this study, Real-time PCR analysis showed that *TP53* and *BCL2* were regulated after treatment with calculated IC_**50**_ concentrations (MDA = 20; MCF-7 = 32 μg/mL) (Fig 13). While the expression of the control samples showed downregulation of *BCL2* and upregulation of *TP53* (Figs 11–13). A study’s findings showed a significant correlation between higher Ki-67 index values, nuclear grade, positive HER2, negative ER/PgR, and TP53 overexpression. Furthermore, there was a strong correlation between the subtypes of breast cancer and the TP53 status. The TN type and the HER2 enriched type had noticeably greater rates of TP53 overexpression than the luminal A and luminal B types. According to one study, *TP53* mutations are present in 17% of cases of luminal A-type, 41% of cases of luminal B-type, 50% of cases of HER2, and 88% of cases of basal-like carcinoma. As a result, high-grade, aggressive, and advanced breast cancer often express TP53 [49]. In 1993, Wang described a correlation between the *BCL2* gene and wild-type *TP53*, stating the crucial role in the induction of apoptosis. Following this scenario, in the cytoplasm of breast cancer cells, an inverse relationship has been described between *BCL2* and *TP53* [50]. It was also seen in the MCF-7 cell line at both mRNA and protein levels that mutant *TP53* overexpression generated *BCL2* downregulation by either triggering suppression of transcription instigation or destabilizing mRNA [47]. Consequently, a clear understanding of the stable reference genes in the studied cell lines is important to learn relative gene expression analysis in cancer cell lines. The stable expression of *Hprt1* with Ct value ranges between 23–24 cycles was observed in both samples (Figs 11–13). Therefore, it is proposed that on treatment with EKA extract, cells undergo an apoptosis-dependent cell death which may be a potential therapeutic choice in treating cancer.

Natural compounds can target multiple pathways that eventually impact the molecular activity of the cells. PI3K/Akt, Ras/MAP-Kinase, Wnt/β-catenin signalling pathways, and the cell cycle pathway has been among the multiple pathways that have been identified to be often changed in cancer. In the triple-negative breast cancer (MDA-MB-231) cell line, honokiol inhibited phospholipase D and Ras activation, nuclear factor-kappa B (NF-kB), COX-2, Prostaglandin E2, Src/epidermal growth factor receptor, and (AMPK/mTOR) signalling pathways. Additionally, paclitaxel, through multiple signalling pathways for example toll-like receptor-4 dependent pathway, Janus kinase- (JAK-) signal transducer, and activator of transcription factor pathway and NF-kB in breast cancer, showed anticancer effects. Furthermore, curcumin appeared as a significant inhibitor of the Janus kinase 2 (JAK2)/STAT3 signalling pathway [51]. In breast cancer, capsaicin raised the expression of Bax and activated caspase 3, and on the other hand, it minimized the expression of BCL2, survivin, and Ki-67 *in vivo* and *in vitro*. Capsaicin has been revealed to reduce the expression of cyclin-dependent kinase 8 (CDK8) in the triple-negative breast cancer cell line [52]. As in our study, we can say that BCL2, and TP53 are the crucial pathways in cancer signalling that the phytocompounds can effectively modify their signalling mechanism/expression.

A deeper understanding of the mechanisms of action of phytochemicals with their targets to activate or inhibit enzymatic pathways or proteins for the treatment of a specific disease [53]. The present study followed Lipinski’s rules that fulfil the toxicological requirements for the screening of drug-like compounds. Any compound that exceeds these limits may lose its important characteristics related to absorption, distribution, excretion, and metabolism, making it incompatible to be further explored as a drug [47]. As per these criteria, only three out of sixty-four compounds present in both plant extracts were selected. It is established that the tumour microenvironment (TME) is a complex network influenced by cellular metabolism, dysfunctional oncogenic signalling, epigenetic factors, and genetic factors that drive cancer through the stages of initiation to metastasis [54]. Breast cancer is highly heterogeneous both in terms of its histology and molecular composition, which affects treatment outcomes and response. When choosing a course of therapy or prognosis, immunohistochemical markers (IHC) are often employed to determine the expression of estrogen receptor (ER), human epidermal growth factor receptor 2 (HER2), and progesterone receptor (PR). The activity of the BCL-2 protein family is affected by tyrosine kinase-activated (RTK) pathways. In breast cancer cells, the amplification and/or overexpression of several RTK receptor types including epidermal growth factor receptor (EGFR), human epidermal growth factor2 (HER2), and insulin growth factor (IGFR) drive signalling cascades that promote cell survival and proliferation. A diverse range of signalling pathways such as the mitogen-activated protein kinase (MAPK) and the phosphatidylinositol 3-kinase/mammalian target of rapamycin (PI3K/mTOR) emerge from RTK signalling [55]. Modern theories state that the activity of transcription factors such as c-Myc, p53, p63, p73, and others activate a variety of stress factors in the intrinsic (mitochondrial) apoptotic pathway thereby causing an increased expression of the pro-apoptotic proteins Bax, Bim, Puma, and Noxa. As aforementioned, p53/TP53 is a crucial tumour suppressor factor that induces apoptosis and prevents the formation of carcinomas in organisms. It was shown by genome-wide sequencing that around 30% of breast cancers have mutations in the *TP53* gene. On the other hand, in HER2-positive tumours, 70% of the tumours carry mutations in *TP53* [56]. Furthermore, the BCL2 protein, known for its antiapoptotic nature, is implicated in tumour formation, as well as in the emergence of resistance against multiple drugs by preventing apoptosis and regulating cell growth. Several studies have reported increased BCL2 protein and mRNA levels in breast tumour tissues. Thus, a novel therapeutic strategy to overcome resistance against apoptosis in cancer cells could be devised by targeting the BCL2 protein [57]. Addressing these pathways has been demonstrated in preclinical research to effectively suppress tumorigenesis and metastasis, suggesting that they could be useful therapeutic strategies for individuals with inflammation-related breast cancer (BC) [11].

Multiple mechanisms which include phosphorylation, dephosphorylation by protein serine/threonine phosphatase-1, acetylation by the transcriptional coactivator p300/CBP, and conformational changes mediated by the prolyl isomerase Pin1 [58] have been predicted to involve in the regulation of TP53. Several transcription factors, such as E2F-1, the nuclear factor kappa B (NF-kB) family, and Janus kinase (JAK)-signal transducers and activators of transcription (STAT) can transcriptionally regulate the expression of pro-survival of the BCL2 protein family. At the transcriptional, post-transcriptional, and post-translational levels, the expression of the pro-apoptotic BCL2 family proteins is also significantly controlled. The genes that encode the BH3-only proteins PUMA and NOXA are transcriptionally amplified by the tumour suppressor TP53. Although the importance of BIM regulation by the FOXO [59] transcription of the BCL2L11 gene, encoding BIM, has been revealed to be synchronized by FOXO3a, c-MYC, NF-Y, SMAD1/3, RUNX1-3, c-Jun, and RELA.

Consequently, we have selected key anticancer targets such as TP53, BCL2, EGFR, and HER2 as they have a significant role in breast tumour formation. In the molecular docking procedure, among all compounds propanoic acid, 3-(2,3,6-trimethyl-1,4-dioxaspiro[4.4] non-7-yl)-, methyl ester, Linolenic acid, and Palmitic acid exhibited good affinity with more interactions (Fig 14) (Tables 5 and 6). The molecular docking method has been useful in identifying potential inhibitors for BCL2, TP53, EGFR, and HER-2 from KA extracts. The current study provides an essential cytotoxic profile of KA compounds, but there is a need to study how these novel therapeutic agents can be integrated into novel products or supplements. It demands an in-depth knowledge of the mechanisms of action, potential applications, regulatory requirements, and safety considerations. The development of a suitable formulation is vital following the identification of potential compounds to ensure bioavailability, stability, and targeted delivery. Formulation approaches include encapsulation within nanoparticles, liposomes, phytosomes, neosomes, nanospheres, or micelles, to enhance bioavailability, increase solubility, and target particular cells or tissues. The top-hit biologically active molecules obtained from the KA extracts exhibited increased apoptotic and antitumor potential, indicative of their potential use in the development of multiple-targeted antitumor medicines.

## Conclusion

In conclusion, this study presents the secondary metabolite composition of *K. africana* leaf extracts. When evaluated on two different breast cancer cell lines the EKA extract showed the highest cytotoxicity. The results of the cytotoxicity testing indicate that the effect is brought about by a synergy of various compounds, rather than the effect of a single compound. The most significant challenge in cancer treatment is reducing the levels of inflammatory markers that promote cancer growth and simultaneously promote the body’s natural anticancer mechanisms. To improve the quality of anticancer therapies for malignant tumours and cancers that cause high mortality, it is essential to uncover and understand the basic mechanisms of MDR-multiple drug-resistant proteins. The current study is a promising addition to the research on the proposed genetic modifications in the TP53 and BCL2 mRNA expressions in breast cancer cell lines. *In silico* characterization of phytocompounds against important oncogenic proteins concerned with genomic instability may be essential to decipher novel mechanistic insights.

To enhance the pharmacokinetic profile and minimize off-target effects, drugs should be administered through carriers that limit drug breakdown and transformation in the circulatory, as well as phagocytic pathways. Different targeting approaches employ a diversity of bioflavonoids, nanoparticles (NPs), phytocompounds, and small molecules that primarily act by causing inhibition of the MAPK, PI3K-AKT, and the STAT3 pathways, as well as by preventing TP53 formation, and by causing increased transcription of the antiapoptotic proteins including (BCL2 and BCL-xL) NF-κB, and RTK proteins that are involved in cancer-promoting mechanisms. It is necessary to develop low-toxicity combinations of anticancer drugs that target signalling pathways associated with inflammation in breast cancer cells with diverse phenotypic characteristics. As a result, it is possible that this plant and its lead constituents can potentially be developed into an effective, safe, and specific drug for managing and treating breast tumours. However, to develop novel medicinal formulations, further *in vivo* and *silico* molecular dynamic studies are essential for validating their anticancer activity by investigating the different underlying pathways implicated in breast cancer development.

## Supporting information

S1 Fig GCMS instrument control parameters start. System generated file.
(PDF)

S2 Fig GCMS instrument control parameters end. System generated file.
(PDF)

S3 Fig GCMS report of *K. Africana*- Hexane extract. (PDF)

S4 Fig GCMS report of *K. Africana*- Ethanol extract. (PDF)

S5 Fig GC-MS identified phytocompounds from *K. africana* extracts: MW: Molecular Weight; MF: Molecular Formula; RT: Retention Time. (DOCX)
