# Supplementary material for: Gene expression and anticancer evaluation of Kigelia africana (Lam.) Benth. Extracts using MDA-MB-231 and MCF-7 cell lines
Source: PLoS One. 2024 Jun 5;19(6):e0303134. doi: 10.1371/journal.pone.0303134 (PMC11152317; doi:10.1371/journal.pone.0303134)
Supplement: S1 Fig — System generated file. (PDF) [file pone.0303134.s001.pdf]

INSTRUMENT CONTROL PARAMETERS: GCMS TQQQ

D:\MassHunter\GCMS\2\methods\MASS LAB METHOD\ASIA

Control Information

Sample Inlet : GC
Injection Source : PAL Sampler
Mass Spectrometer : Enabled

Injection Volume:	1500 µl
Overlap Injection Mode:	No Overlap

PAL Method Information

Syringe:	2.5ml-HS
Cycle:	MACRO HS-NO4-V2

Parameters of PAL Cycle

Incubation Temperature (°C):	130
Incubation Time (s):	900
Agitator On Time (s):	5
Agitator Off Time (s):	2
Syringe Temperature (°C):	140
Agitator Speed (rpm):	500
Fill Speed (µl/s):	100
Fill Strokes :	0
Pullup Delay (ms):	1000
Inject to:	GC Inj1
Injection Speed (µl/s):	500
Pre Inject Delay (ms):	500
Post Inject Delay (ms):	500
Flush Time (s):	10
GC Runtime (s):	1000

No Sample Prep method has been assigned to this method.

Oven	
Equilibration Time	0.5 min
Max Temperature	340 degrees C
Slow Fan	Disabled
Oven Program	On
50 °C for 3 min	
#1 then 7 °C/min to 180 °C for 25 min	
#2 then 7 °C/min to 300 °C for 25 min	
Run Time	88.714 min

QQQ Collision Cell EPC	
He Quench Gas	Off
N2 Collision Gas	Off
