# Supplementary material for: Gene expression and anticancer evaluation of Kigelia africana (Lam.) Benth. Extracts using MDA-MB-231 and MCF-7 cell lines
Source: PLoS One. 2024 Jun 5;19(6):e0303134. doi: 10.1371/journal.pone.0303134 (PMC11152317; doi:10.1371/journal.pone.0303134)
Supplement: S2 Fig — System generated file. (PDF) [file pone.0303134.s002.pdf]

Sample Overlap
Sample overlap is not enabled

Front SS Inlet He

Mode	Split
Heater	On 260 °C
Pressure	On 9.05 psi
Total Flow	On 15.419 mL/min
Septum Purge Flow	On 3 mL/min
Gas Saver	On 20 mL/min After 2 m
Split Ratio	10 :1
Split Flow	11.29 mL/min

Thermal Aux 2 (MSD Transfer Line)

Heater	On
Temperature Program	On
260 °C for 0 min	
Run Time	88.714 min

Column #1
OPTIMA -OPTIMA 5MS
340 °C: 30 m x 250 µm x 0.25 µm
In: Front SS Inlet He
Out: Vacuum

(Initial)	50 °C
Pressure	9.05 psi
Flow	1.129 mL/min
Average Velocity	38.724 cm/sec
Holdup Time	1.2912 min
Flow Program	On
1.129 mL/min for 0 min	
Run Time	88.714 min

Signals

Signal #1: Test Plot	Save Off<br>50 Hz
Signal #2: Test Plot	Save Off<br>50 Hz
Signal #3: Test Plot	Save Off<br>50 Hz
Signal #4: Test Plot	Save Off<br>50 Hz

END OF INSTRUMENT CONTROL PARAMETERS
