# Supplementary material for: Gene expression and anticancer evaluation of Kigelia africana (Lam.) Benth. Extracts using MDA-MB-231 and MCF-7 cell lines
Source: PLoS One. 2024 Jun 5;19(6):e0303134. doi: 10.1371/journal.pone.0303134 (PMC11152317; doi:10.1371/journal.pone.0303134)
Supplement: S3 Fig — (PDF) [file pone.0303134.s003.pdf]

**Sample Name**	AASIA-KIG-HEX-2a-040	**Position**	14	**Instrument Name**	GCMS TQQQ	**User Name**	HEJ-G-104-03\Adminis
**Inj Vol**	1.5	**InjPosition**		**SampleType**		**IRM Calibration Status**	Not Applicable
**Data Filename**	AASIA-KIG-HEX-2a-040	**ACQ Method**	AASIA KALSOOM .M	**Comment**		**Acquired Time**	6/4/2022 12:21:19 PM

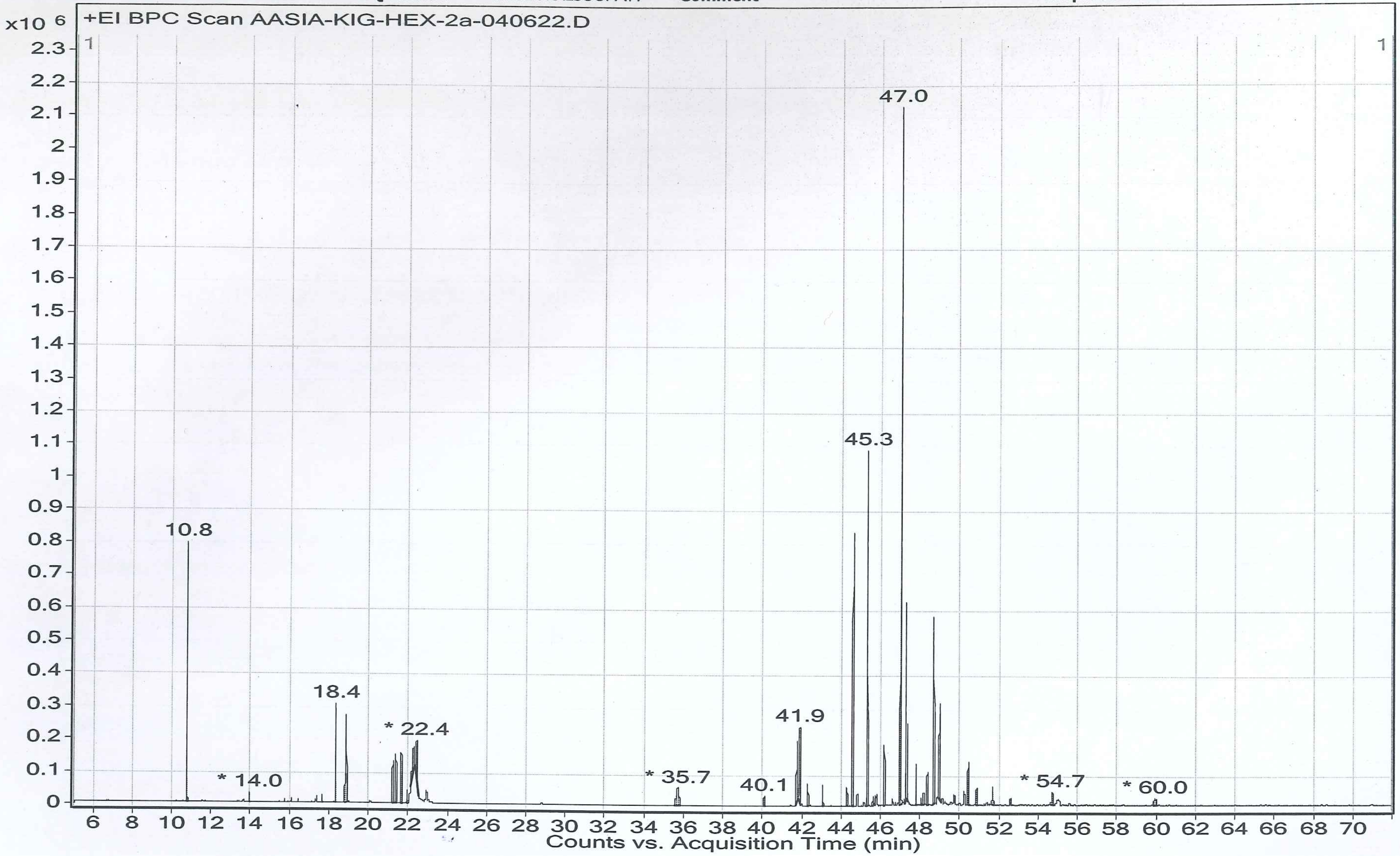

KIG-HEX-2A

Peak Number	RT	Area	Area %	Height	Width	Area Sum %	Height %
1	10.8	1283000	24.85	793210	0.1	5.25	37.37
2	14	41957	0.81	30435	0	0.17	1.43
3	17.7	33046	0.64	22998	0	0.14	1.08
4	18.4	623820	12.08	302100	0.1	2.55	14.23
5	18.9	1020520	19.77	267580	0.2	4.17	12.61
6	21.3	405275	7.85	128009	0.1	1.66	6.03
7	21.4	501996	9.72	151217	0.1	2.05	7.12
8	21.7	511539	9.91	151903	0.1	2.09	7.16
9	22	127098	2.46	40161	0.1	0.52	1.89
10	22.3	485273	9.4	70992	0.2	1.99	3.34
11	22.4	668636	12.95	117204	0.2	2.74	5.52
12	35.7	485552	9.41	52052	0.3	1.99	2.45
13	40.1	110803	2.15	30258	0.1	0.45	1.43
14	41.8	610430	11.82	183493	0.1	2.5	8.65
15	41.9	862109	16.7	228338	0.1	3.53	10.76
16	42.3	181765	3.52	68522	0.1	0.74	3.23
17	43.1	151330	2.93	62966	0.1	0.62	2.97
18	44.3	125372	2.43	55690	0.1	0.51	2.62
19	44.6	1848870	35.82	828962	0.1	7.56	39.06
20	45.3	2333089	45.2	1080698	0.1	9.54	50.92
21	45.6	51871	1	27500	0.1	0.21	1.3
22	45.7	67902	1.32	31090	0.1	0.28	1.46
23	46.2	382136	7.4	183752	0.1	1.56	8.66
24	47	5162225	100	2122500	0.2	21.12	100
25	47.3	1302993	25.24	601831	0.1	5.33	28.35
26	47.4	431083	8.35	231214	0.1	1.76	10.89
27	47.8	264466	5.12	124465	0.1	1.08	5.86
28	48.1	62026	1.2	22008	0.1	0.25	1.04
29	48.2	76441	1.48	35082	0.1	0.31	1.65
30	48.4	278183	5.39	100201	0.1	1.14	4.72
31	48.7	1468059	28.44	567077	0.1	6.01	26.72
32	49	1216565	23.57	291742	0.2	4.98	13.75
33	49.7	57675	1.12	22608	0.1	0.24	1.07
34	50.4	417595	8.09	129683	0.1	1.71	6.11
35	50.9	178933	3.47	53287	0.1	0.73	2.51
36	51.7	199876	3.87	55504	0.1	0.82	2.62
37	52.6	64733	1.25	19586	0.1	0.26	0.92
38	54.7	203849	3.95	38482	0.2	0.83	1.81
39	60	147425	2.86	21296	0.2	0.6	1
						99.99	

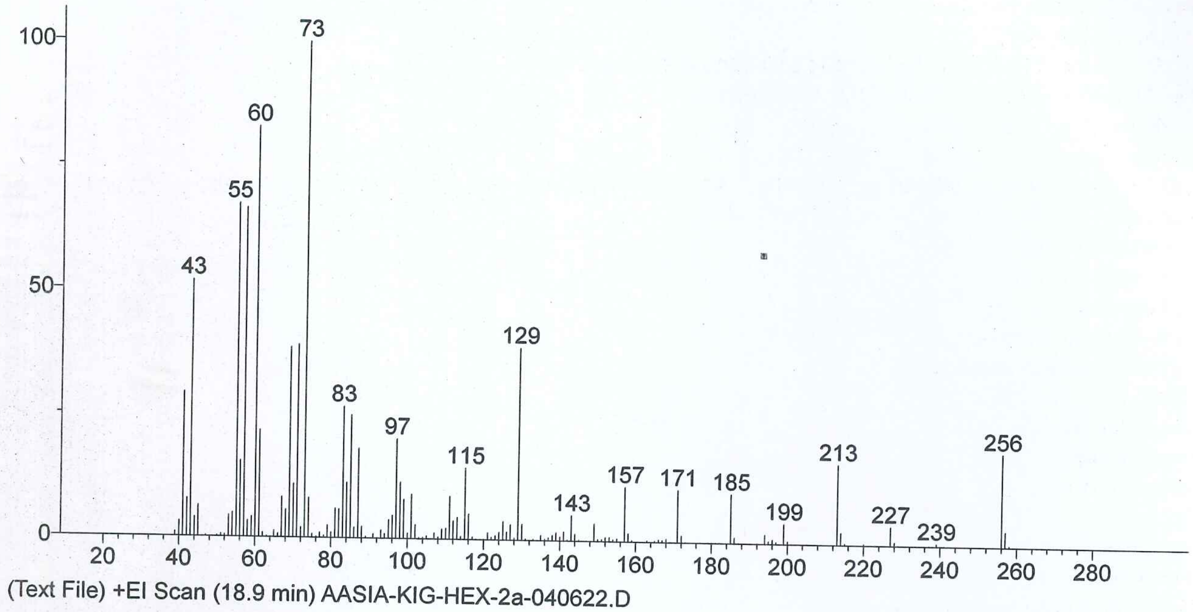

(Text File) +EI Scan (18.9 min) AASIA-KIG-HEX-2a-040622.D

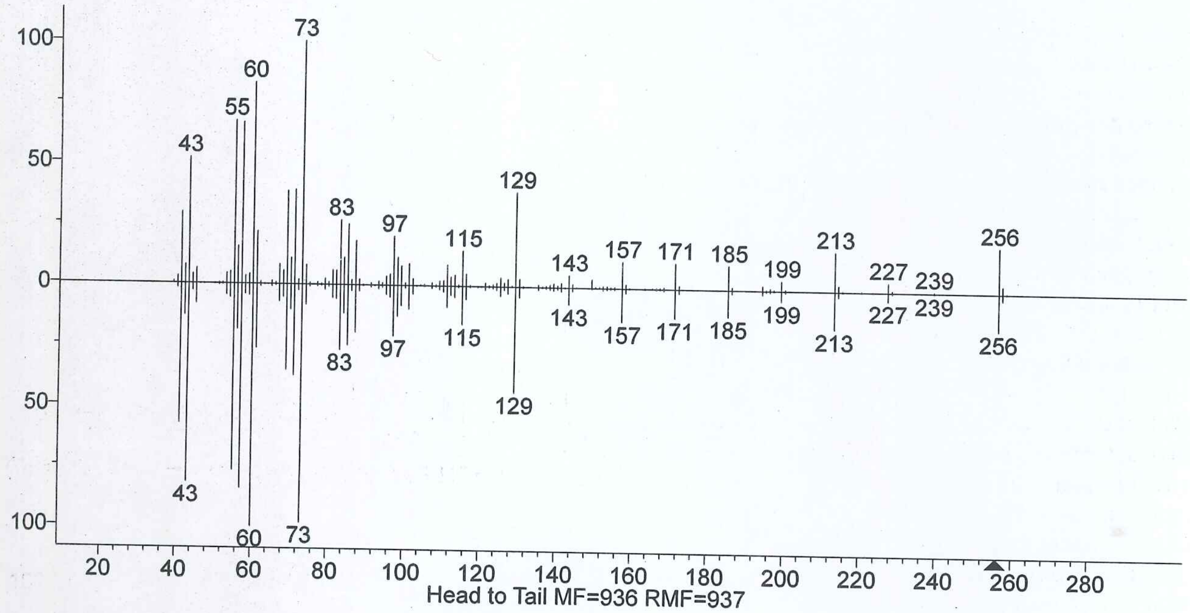

Head to Tail MF=936 RMF=937

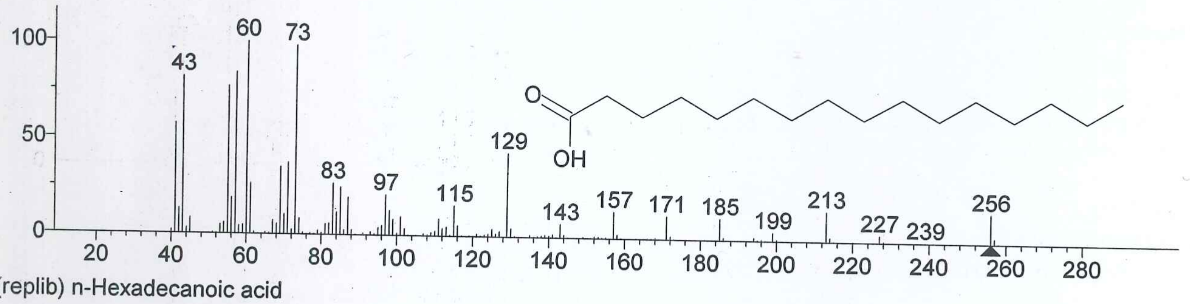

(replib) n-Hexadecanoic acid

Name: n-Hexadecanoic acid
Formula: $C_{16}H_{32}O_2$
MW: 256 CAS#: 57-10-3 NIST#: 335494 ID#: 6723 DB: replib
Other DBs: Fine, TSCA, RTECS, EPA, HODOC, NIH, EINECS, IRDB
Contributor: Drug Lab
10 largest peaks:
60 999 | 73 980 | 57 840 | 43 817 | 55 767 | 41 574 | 129 435 | 71 373 | 69 351 | 83 267 |
Synonyms:
1.Hexadecanoic acid
2.n-Hexadecoic acid
3.Palmitic acid
4.Pentadecanecarboxylic acid
5.1-Pentadecanecarboxylic acid
6.Cetylic acid
7.Emersol 140
8.Emersol 143
9.Hexadecylic acid
10.Hydrofol
11.Hystrene 8016
12.Hystrene 9016
13.Industrene 4516
14.Prifrac 2960
15.Glycon P-45
16.Prifac 2960
17.Univol U332

Estimated non-polar retention index (n-alkane scale):
Value: 1968 iu
Confidence interval (Carboxylic acids): 51(50%) 220(95%) iu

Retention index.
1. Value: 1942 iu
Column Type: Capillary
Column Class: Standard non-polar
Active Phase: RTX-1
Column
Length: 60 m
Carrier Gas: He
Column Diameter: 0.22 mm
Phase Thickness: 0.25 um
Data Type: Linear
RI
Program Type: Ramp
Start T: 60 C
End T: 230 C
Heat Rate: 2 K/min
End Time: 35 min
Source: Paolini, J.;
Muselli, A.; Bernardini, A.-F.; Bighelli, A.; Casanova, J.; Costa, J., Thymol derivatives from essential oil of Doronicum corsicum L., Flavour Fragr. J., 22, 2007, 479-487.

2. Value: 1972 iu
Column Type: Capillary
Column

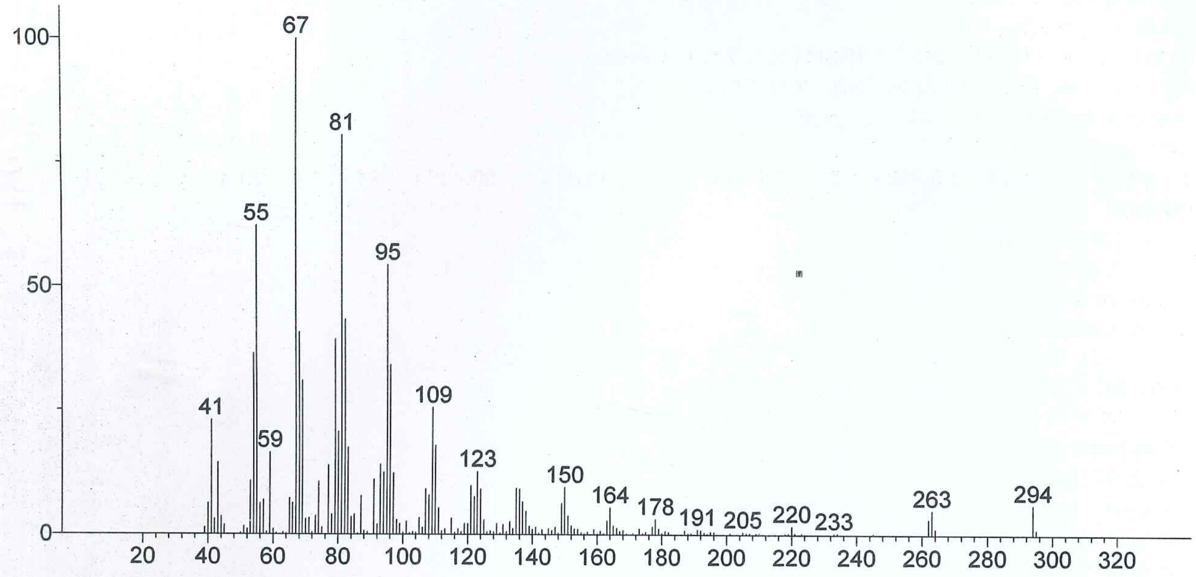

(Text File) +EI Scan (21.2 min) AASIA-KIG-HEX-2a-040622.D

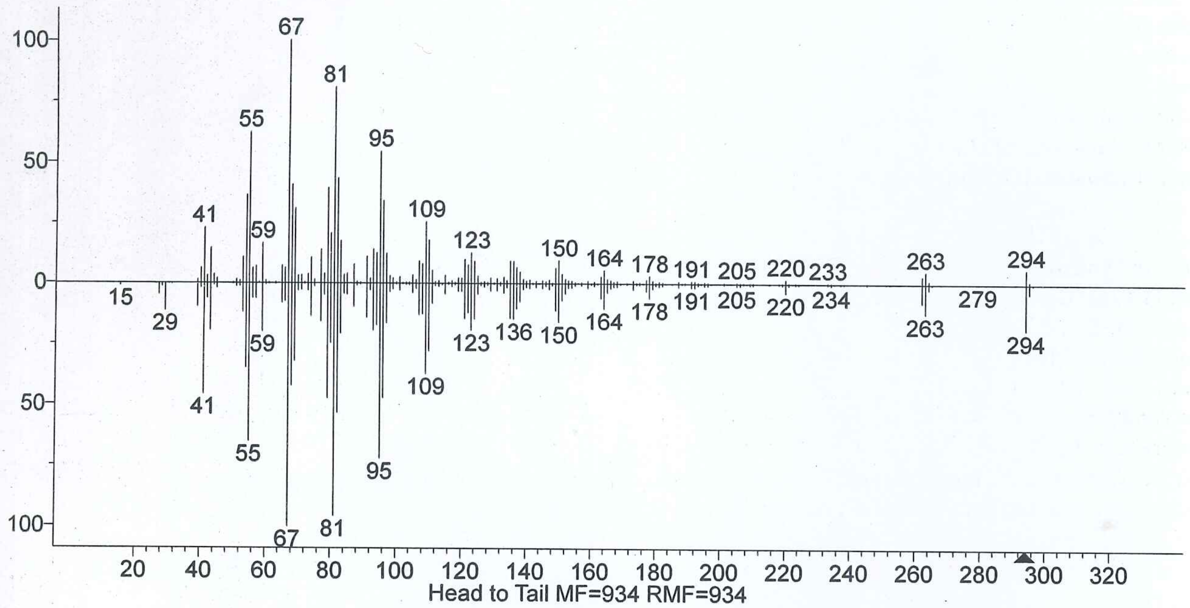

Head to Tail MF=934 RMF=934

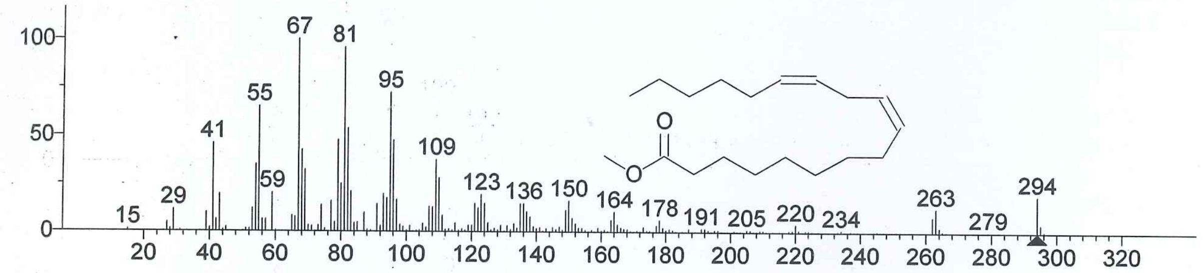

(mainlib) 9,12-Octadecadienoic acid (Z,Z)-, methyl ester

Name: 9,12-Octadecadienoic acid (Z,Z)-, methyl ester
Formula: $C_{19}H_{34}O_2$
MW: 294 CAS#: 112-63-0 NIST#: 333205 ID#: 28886 DB: mainlib
Other DBs: Fine, TSCA, EPA, HODOC, NIH, EINECS
Contributor: NIST Mass Spectrometry Data Center
10 largest peaks:
67 999 | 81 956 | 95 720 | 55 650 | 82 534 | 79 474 | 96 472 | 41 456 | 68 423 | 109 372 |
Synonyms:
1.Linoleic acid, methyl ester
2.Methyl cis,cis-9,12-octadecadienoate
3.Methyl linoleate
4.Methyl octadecadienoate
5.Methyl 9-cis,12-cis-octadecadienoate
6.Methyl (9Z,12Z)-9,12-octadecadienoate #

Estimated non-polar retention index (n-alkane scale):
Value: 2093 iu
Confidence interval (Esters): 47(50%) 201(95%) iu

Retention index.
1. Value: 2071 iu
Column Type: Capillary
Column Class: Standard non-polar
Active Phase: SPB-1
Column
Length: 30 m
Carrier Gas: He
Column Diameter: 0.25 mm
Phase Thickness: 0.25 um
Data Type: Linear
RI
Program Type: Ramp
Start T: 50 C
End T: 250 C
Heat Rate: 5 K/min
Start Time: 3 min
End Time: 15
min
Source: Blagojevic, P.; Radulovic, N.; Palic, R.; Stojanovic, G., Chemical composition of the essential oils of Serbian wild-growing Srtemisia absinthium and Artemisia vulgaris, J. Agric. Food Chem., 54, 2006, 4780-4789.

2.
Value: 2087 iu
Column Type: Capillary
Column Class: Standard non-polar
Active Phase: BP-1
Column Length:
30 m
Carrier Gas: N2
Column Diameter: 0.32 mm
Phase Thickness: 0.25 um
Data Type: Linear RI
Program
Type: Ramp

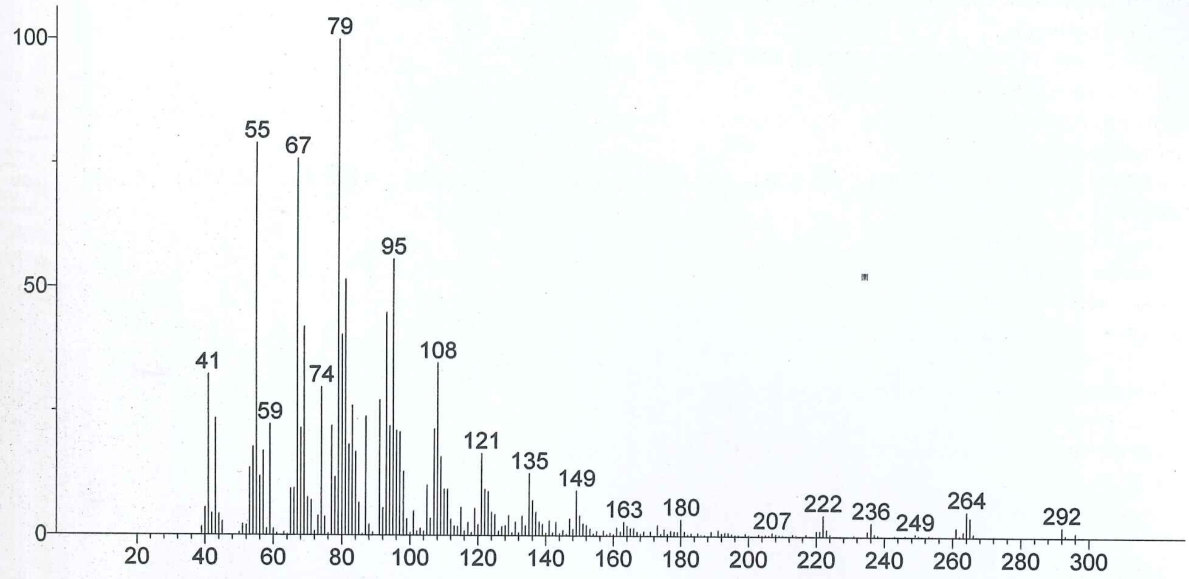

(Text File) +EI Scan (21.4 min) AASIA-KIG-HEX-2a-040622.D

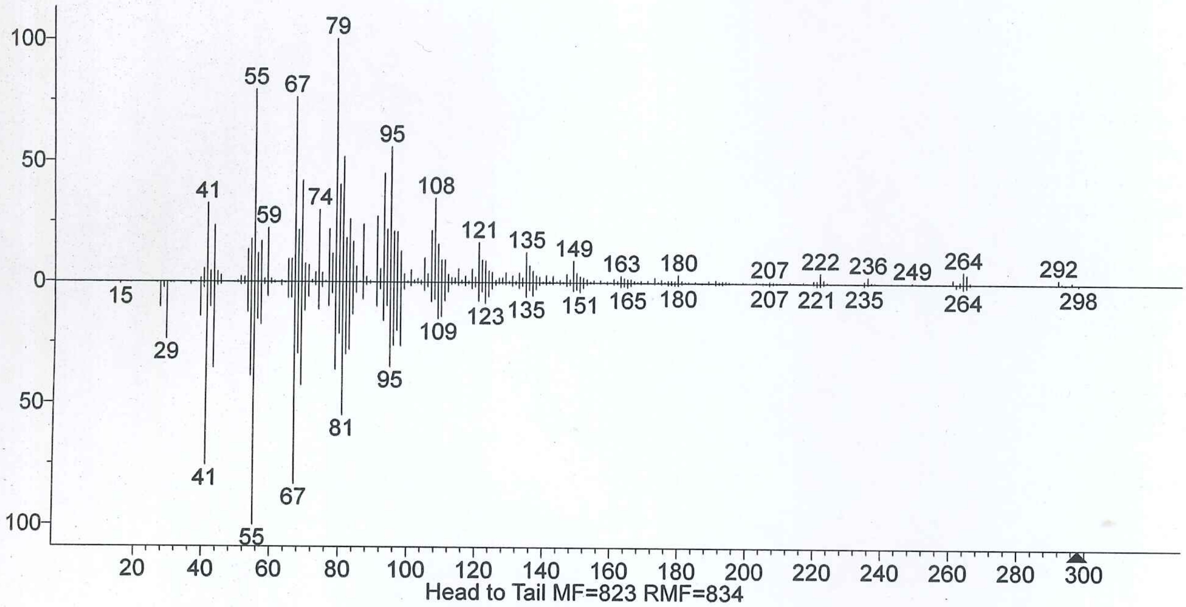

Head to Tail MF=823 RMF=834

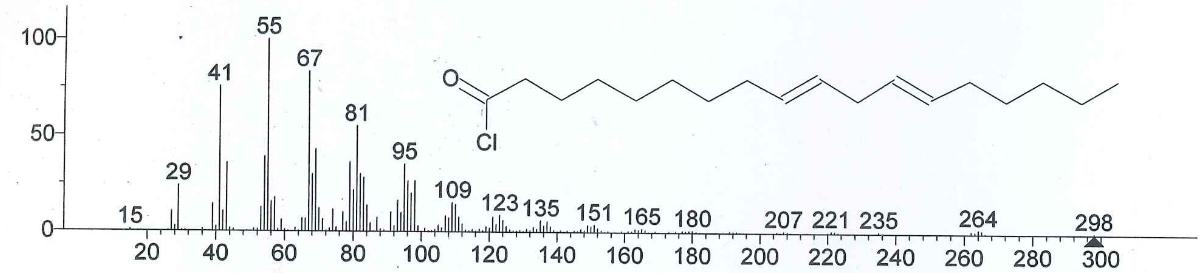

(replib) 9,12-Octadecadienoyl chloride, (Z,Z)-

Name: 9,12-Octadecadienoyl chloride, (Z,Z)-
Formula: $C_{18}H_{31}ClO$
MW: 298 CAS#: 7459-33-8 NIST#: 76312 ID#: 4450 DB: replib
Other DBs: Fine, TSCA, NIH, EINECS
Contributor: RADIAN CORP
10 largest peaks:
55 999 | 67 832 | 41 754 | 81 549 | 69 426 | 54 389 | 79 362 | 43 355 | 95 350 | 82 299 |
Synonyms:
1.Linoleoyl chloride
2.Lineoleoyl chloride
3.Linoleic acid chloride
4.(9E,12E)-9,12-Octadecadienoyl chloride #

Estimated non-polar retention index (n-alkane scale):
Value: 2139 iu
Confidence interval (Diverse functional groups): 89(50%) 382(95%) iu

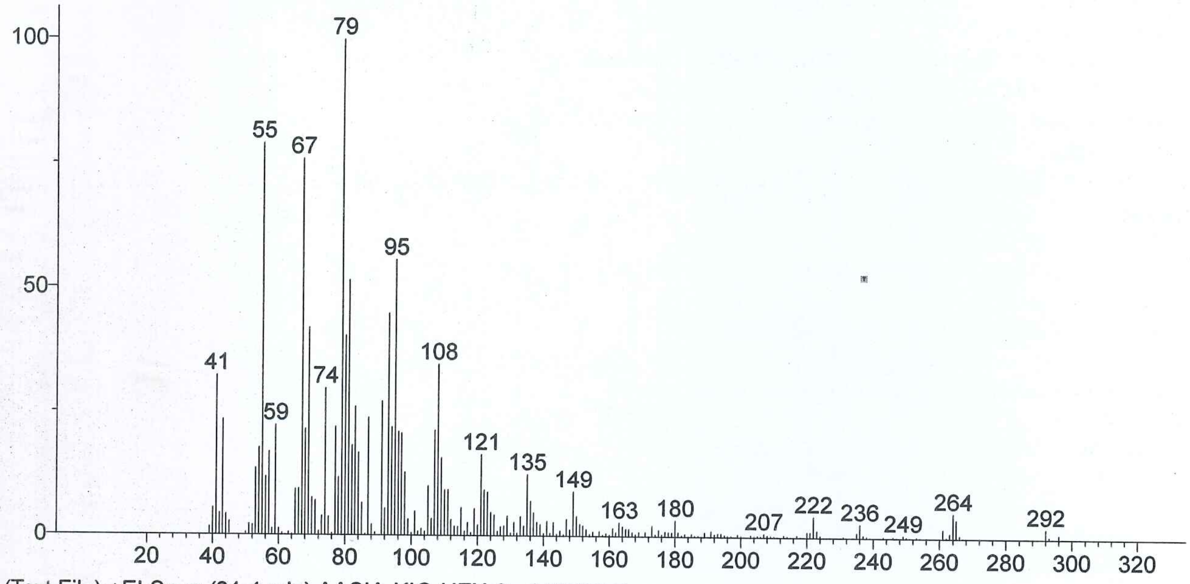

(Text File) +EI Scan (21.4 min) AASIA-KIG-HEX-2a-040622.D

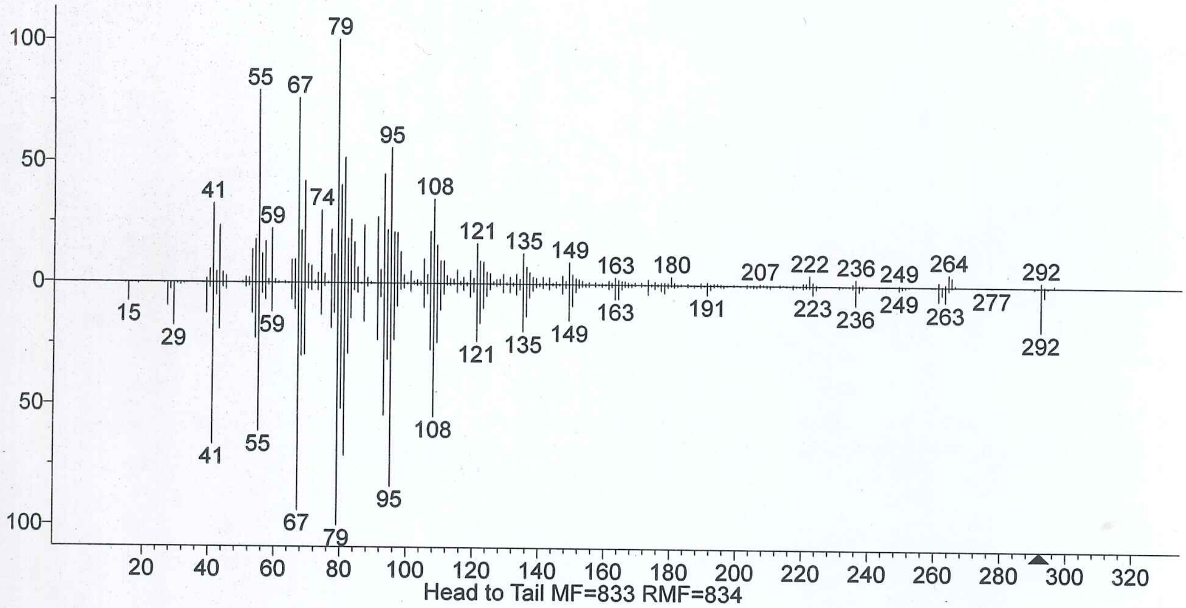

Head to Tail MF=833 RMF=834

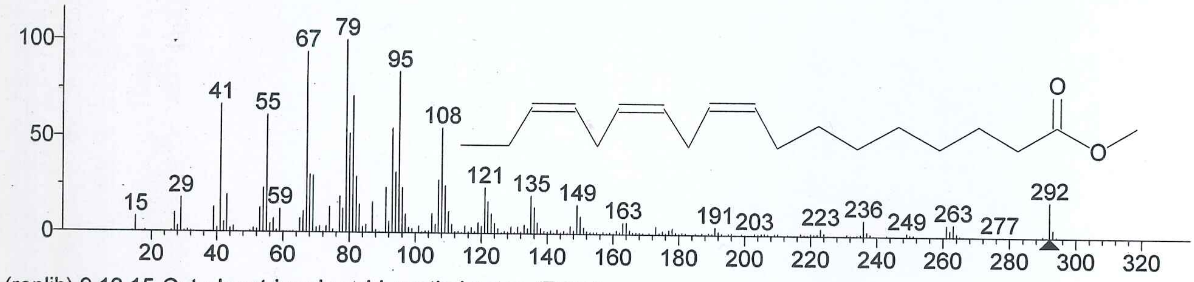

(replib) 9,12,15-Octadecatrienoic acid, methyl ester, (Z,Z,Z)-

Name: 9,12,15-Octadecatrienoic acid, methyl ester, (Z,Z,Z)-
Formula: $C_{19}H_{32}O_2$
MW: 292 CAS#: 301-00-8 NIST#: 228068 ID#: 9679 DB: replib
Other DBs: Fine, TSCA, HODOC, EINECS
Contributor: Japan AIST/NIMC Database- Spectrum MS-NW-1715
10 largest peaks:
79 999 | 67 937 | 95 837 | 81 710 | 41 664 | 55 610 | 108 547 | 93 543 | 80 515 | 94 314 |
Synonyms:
1.Linolenic acid, methyl ester
2.Methyl all-cis-9,12,15-octadecatrienoate
3.Methyl linolenate
4.Methyl (9Z,12Z,15Z)-9,12,15-octadecatrienoate #

Estimated non-polar retention index (n-alkane scale):
Value: 2101 iu
Confidence interval (Esters): 47(50%) 201(95%) iu

Retention index.
1. Value: 2077 iu
Column Type: Capillary
Column Class: Standard non-polar
Active Phase: SPB-1
Column
Length: 30 m
Carrier Gas: He
Column Diameter: 0.25 mm
Phase Thickness: 0.25 um
Data Type: Linear
RI
Program Type: Ramp
Start T: 50 C
End T: 250 C
Heat Rate: 5 K/min
Start Time: 3 min
End Time: 15
min
Source: Radulovic, N.; Lazarevic, J.; Stojanovic, G.; Palic, R., Chemotaxonomically significant 2-ethyl substituted fatty acids from Stachys milanii Petrovic (Lamiaceae), Biochem. Syst. Ecol., 34, 2006, 341-344.

2.
Value: 2098 iu
Column Type: Capillary
Column Class: Standard non-polar
Active Phase: HP-1
Column Length:
30 m
Carrier Gas: He
Column Diameter: 0.25 mm
Phase Thickness: 0.33 um
Data Type: Linear RI
Program
Type: Ramp
Start T: 40 C
End T: 260 C

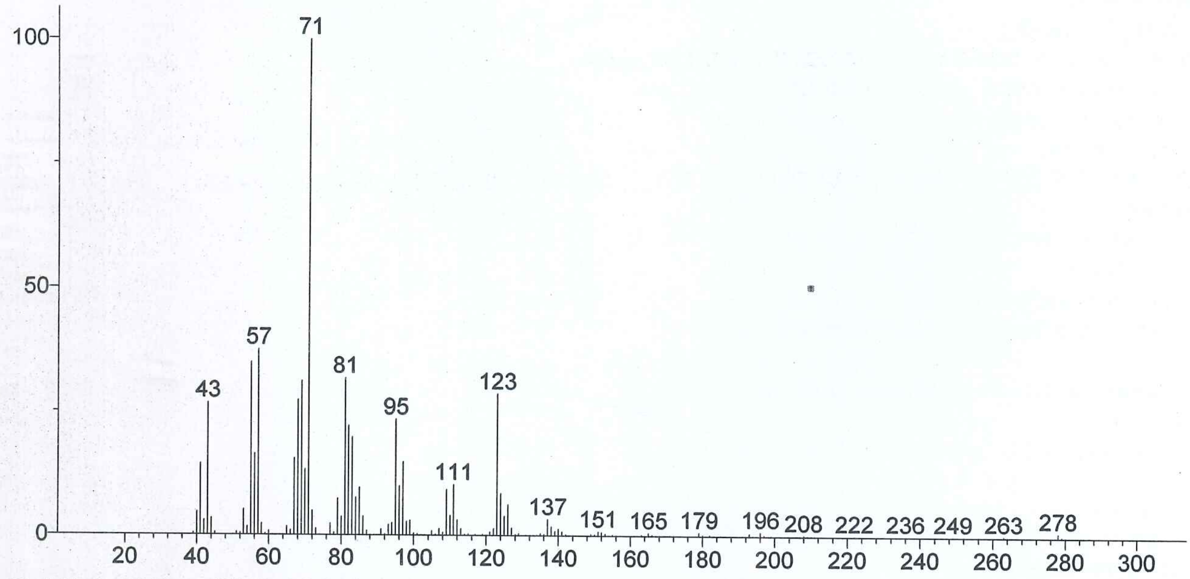

(Text File) +EI Scan (21.7 min) AASIA-KIG-HEX-2a-040622.D

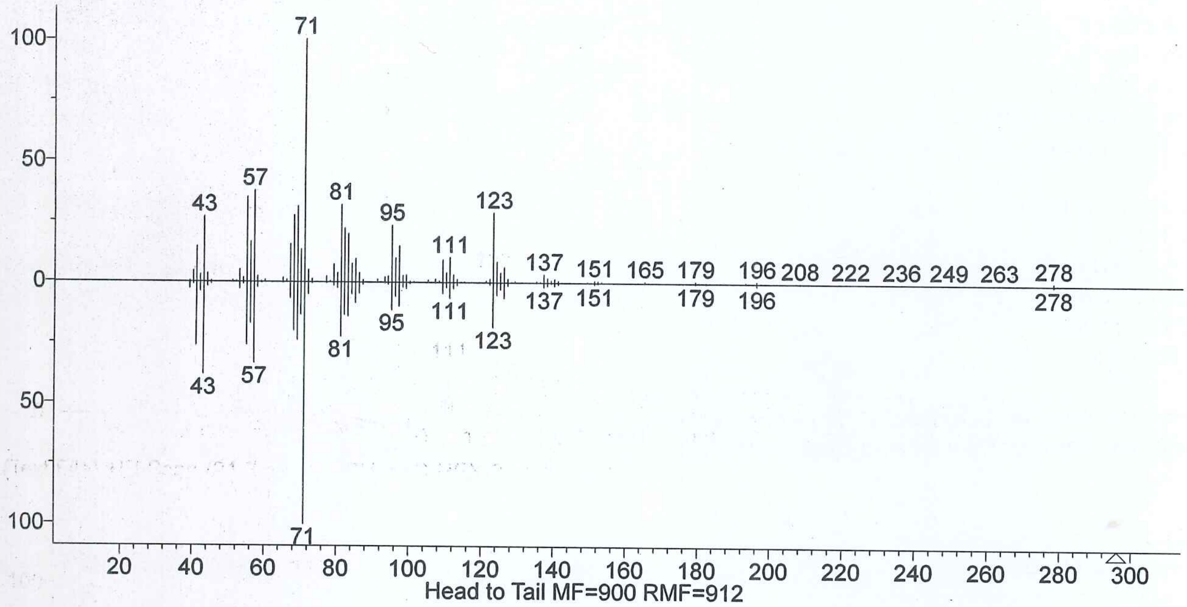

Head to Tail MF=900 RMF=912

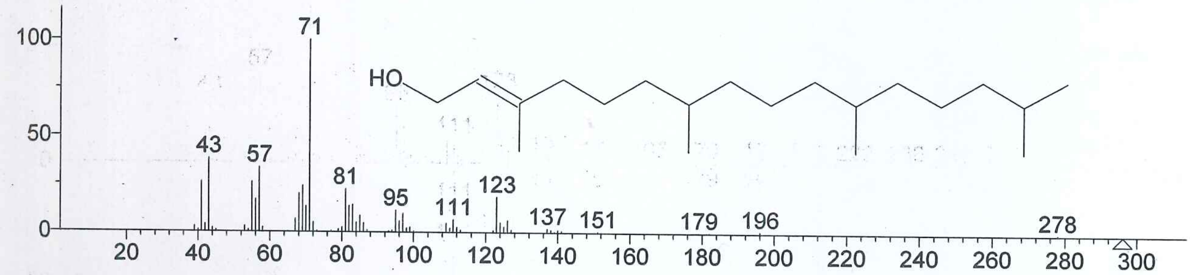

(replib) Phytol

Name: Phytol
Formula: $C_{20}H_{40}O$
MW: 296 CAS#: 150-86-7 NIST#: 108727 ID#: 8051 DB: replib
Other DBs: Fine, TSCA, RTECS, HODOC, EINECS
Contributor: Philip Morris R&D
10 largest peaks:
71 999 | 43 381 | 57 334 | 41 260 | 55 259 | 69 239 | 81 223 | 68 199 | 123 184 | 56 169 |
Synonyms:
1.2-Hexadecen-1-ol, 3,7,11,15-tetramethyl-, [R-[R*,R*-(E)]]-
2.trans-Phytol
3.3,7,11,15-Tetramethyl-2-hexadecen-1-ol
4.(2E)-3,7,11,15-Tetramethyl-2-hexadecen-1-ol #

Estimated non-polar retention index (n-alkane scale):
Value: 2045 iu
Confidence interval (Alcohols): 41(50%) 176(95%) iu

Retention index.
1. Value: 2104 iu
Column Type: Capillary
Column Class: Standard non-polar
Active Phase: SPB-1
Column
Length: 30 m
Carrier Gas: He
Column Diameter: 0.25 mm
Phase Thickness: 0.25 um
Data Type: Linear
RI
Program Type: Ramp
Start T: 50 C
End T: 250 C
Heat Rate: 5 K/min
Start Time: 3 min
End Time: 15
min
Source: Radulovic, N.; Lazarevic, J.; Stojanovic, G.; Palic, R., Chemotaxonomically significant 2-ethyl substituted fatty acids from Stachys milanii Petrovic (Lamiaceae), Biochem. Syst. Ecol., 34, 2006, 341-344.

2.
Value: 2099 iu
Column Type: Capillary
Column Class: Standard non-polar
Active Phase: RTX-1
Column Length:
60 m
Carrier Gas: He
Column Diameter: 0.22 mm
Phase Thickness: 0.25 um
Data Type: Linear RI
Program
Type: Ramp
Start T: 60 C
End T: 230 C

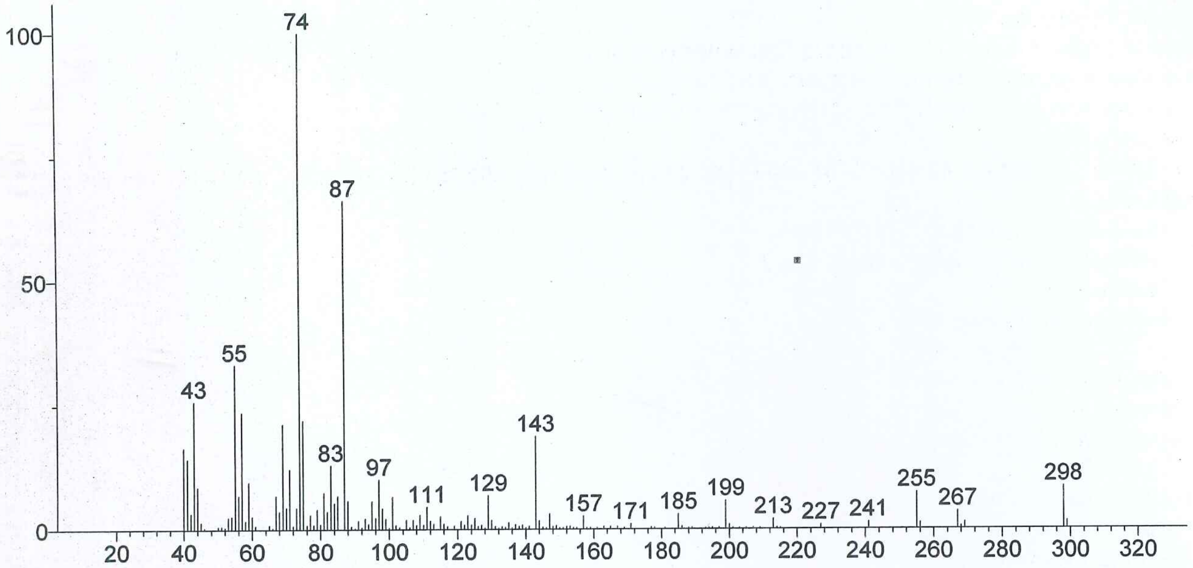

(Text File) +EI Scan (22.0 min) AASIA-KIG-HEX-2a-040622.D

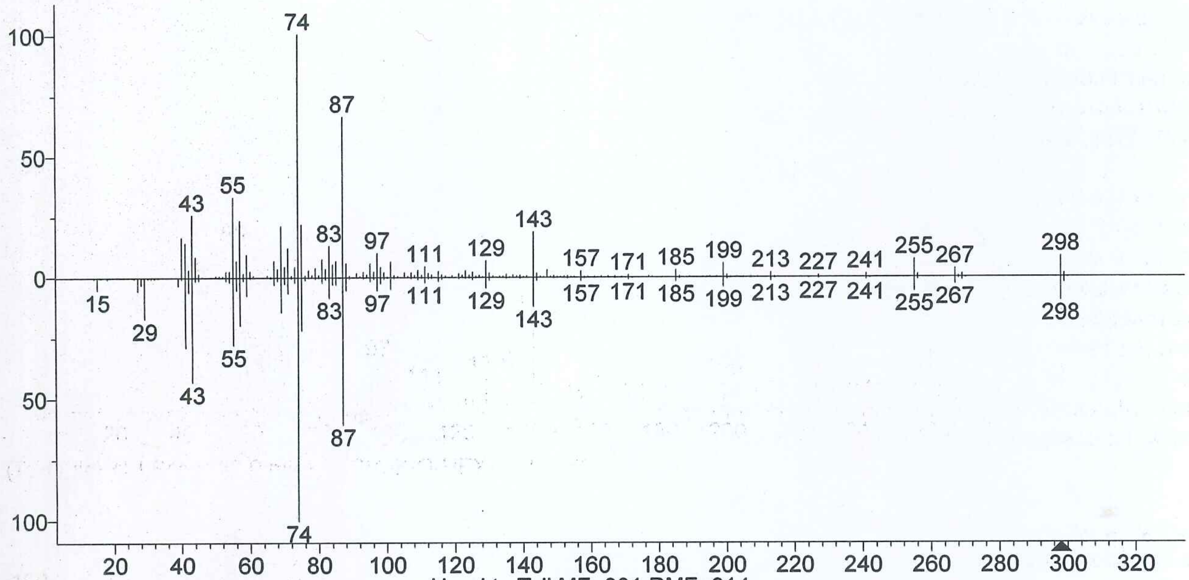

Head to Tail MF=881 RMF=914

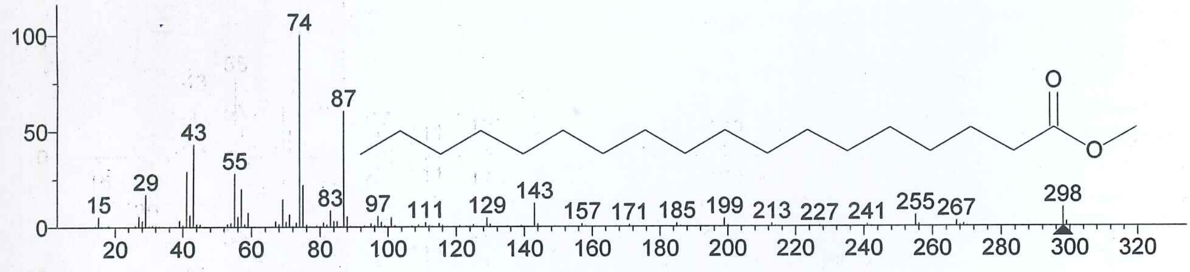

(replib) Octadecanoic acid, methyl ester

Name: Octadecanoic acid, methyl ester
Formula: $C_{19}H_{38}O_2$
MW: 298 CAS#: 112-61-8 NIST#: 79123 ID#: 9086 DB: replib
Other DBs: Fine, TSCA, RTECS, EPA, HODOC, NIH, EINECS, IRDB
Contributor: O A MAMER, MCGILL UNIVERSITY, MONTREAL,
10 largest peaks:
74 999 | 87 604 | 43 430 | 41 289 | 55 278 | 75 218 | 57 197 | 29 169 | 69 143 | 143 121 |
Synonyms:
1.Stearic acid, methyl ester
2.n-Octadecanoic acid, methyl ester
3.Kemester 9718
4.Methyl n-octadecanoate
5.Methyl octadecanoate
6.Methyl stearate
7.Metholene 2218
8.Emery 2218
9.Kemester 9018
10.Methyl ester of octadecanoic acid
11.Kemester 4516
12.Methyl (Z)-9-octadecenoate

Estimated non-polar retention index (n-alkane scale):
Value: 2077 iu
Confidence interval (Esters): 47(50%) 201(95%) iu

Retention index.
1. Value: 2133 iu
Column Type: Capillary
Column Class: Standard non-polar
Active Phase: HP-1
Column
Length: 30 m
Carrier Gas: He
Column Diameter: 0.25 mm
Phase Thickness: 0.33 um
Data Type: Linear
RI
Program Type: Ramp
Start T: 40 C
End T: 260 C
Heat Rate: 2 K/min
Start Time: 5 min
End Time: 20
min
Source: Senatore, F.; Rigano, D.; de Fusco, R.; Bruno, M., Volatile components of Centaurea cineraria L. subsp. umbrosa (lacaita) Pign. and Centaurea napifolia L. (Asteraceae), two species growing wild in Sicily, Flavour Fragr. J., 18, 2003, 248-251.

2. Value: 2111 iu
Column Type: Capillary
Column Class: Standard non
-polar
Active Phase: DB-1
Column Length: 15 m

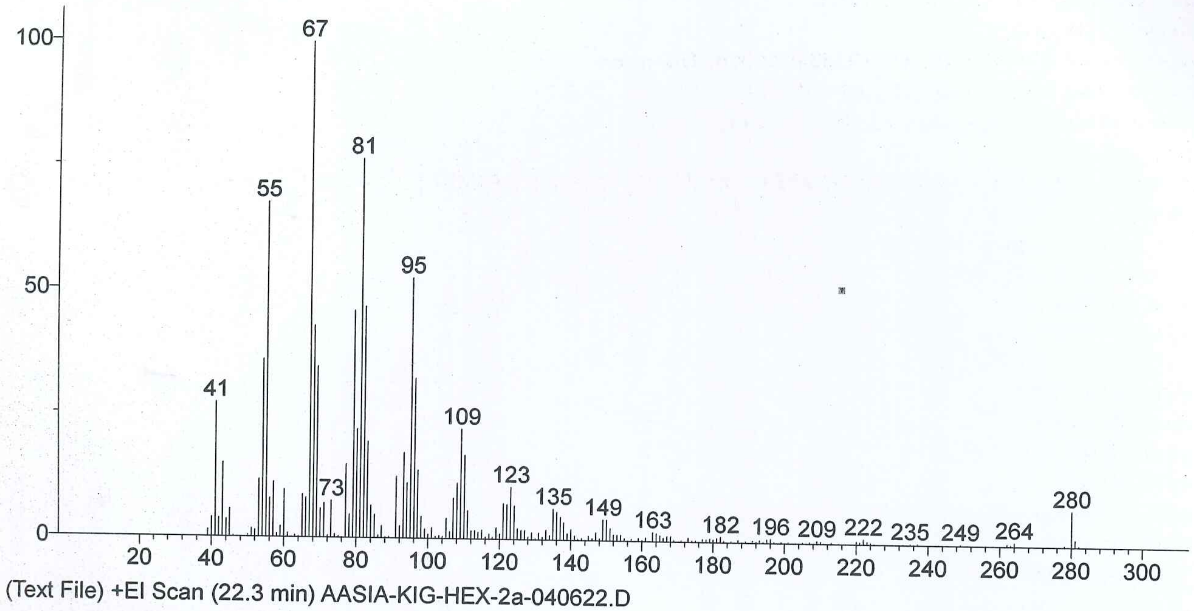

(Text File) +EI Scan (22.3 min) AASIA-KIG-HEX-2a-040622.D

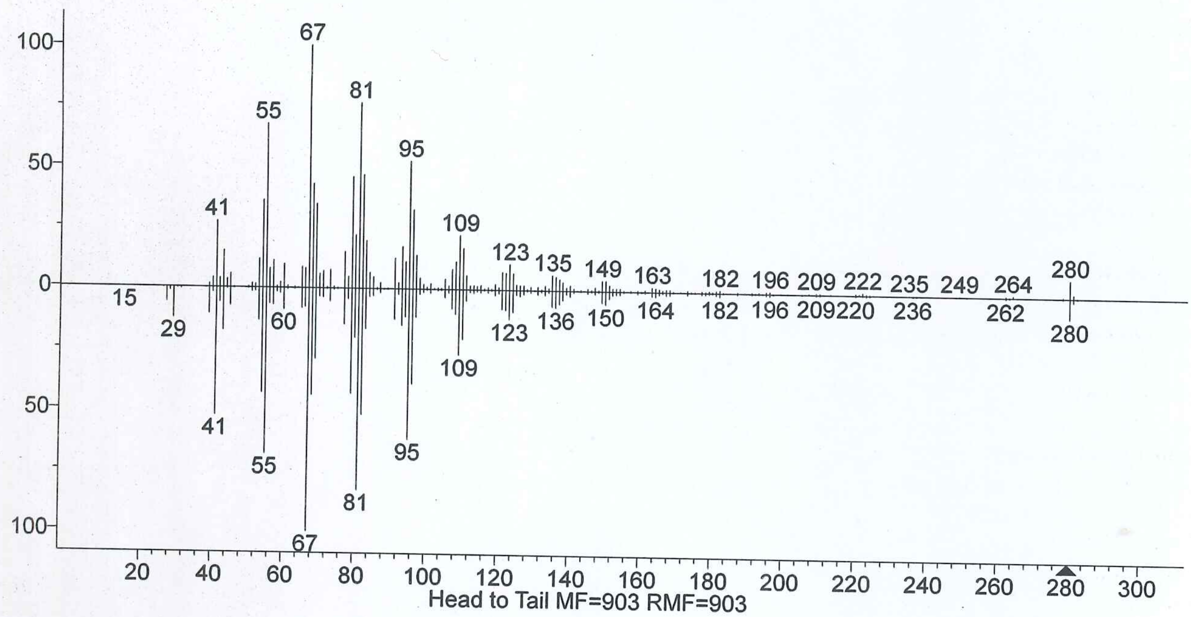

Head to Tail MF=903 RMF=903

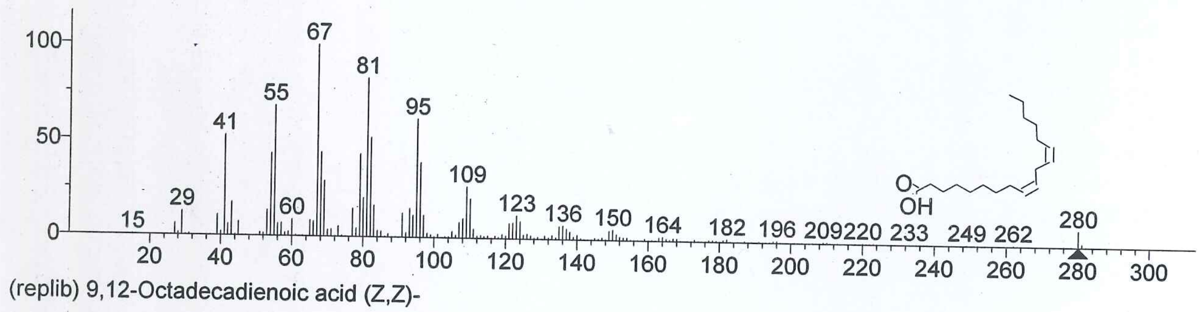

(replib) 9,12-Octadecadienoic acid (Z,Z)-

Name: 9,12-Octadecadienoic acid (Z,Z)-
Formula: $C_{18}H_{32}O_2$
MW: 280 CAS#: 60-33-3 NIST#: 333207 ID#: 7212 DB: replib
Other DBs: Fine, TSCA, RTECS, HODOC, NIH, EINECS
Contributor: NIST Mass Spectrometry Data Center
10 largest peaks:
67 999 | 81 827 | 55 680 | 95 617 | 41 523 | 82 516 | 68 437 | 79 430 | 54 429 | 96 388 |
Synonyms:
1.cis-9,cis-12-Octadecadienoic acid
2.cis,cis-Linoleic acid
3.Grape seed oil
4.Linoleic
5.Linoleic acid
6.Linolic acid
7.Polylin No. 515
8.Telfairic acid
9.Unifac 6550
10.9,12-Octadecadienoic acid
11.Leinoleic acid
12.9,12-Linoleic acid
13.cis,cis-9,12-octadecadienoic acid
14.Linoelaidic acid
15.Linoleic acid 95
16.Emersol 310
17.Emersol 315
18.Vespula pensylvanica b708568k063
19.Pamolyn
20.Pamolyn 125
21.Pamolyn 200, 240
22.Pamolyn 380

Estimated non-polar retention index (n-alkane scale):
Value: 2183 iu
Confidence interval (Carboxylic acids): 51(50%) 220(95%) iu

Retention index.
1. Value: 2095 iu
Column Type: Capillary
Column Class: Standard non-polar
Active Phase: CP Sil 5 CB
Column
Length: 25 m
Carrier Gas: H2
Column Diameter: 0.25 mm
Data Type: Linear RI
Program Type: Ramp
Start T:
80 C
End T: 270 C
Heat Rate: 10 K/min
Source: Ziegenbein, F.C.; Hanssen, H.-P.; König, W.A., Secondary metabolites from Ganoderma lucidum and Spongiporus leucomallellus, Phytochemistry, 67, 2006, 202-211.

2.

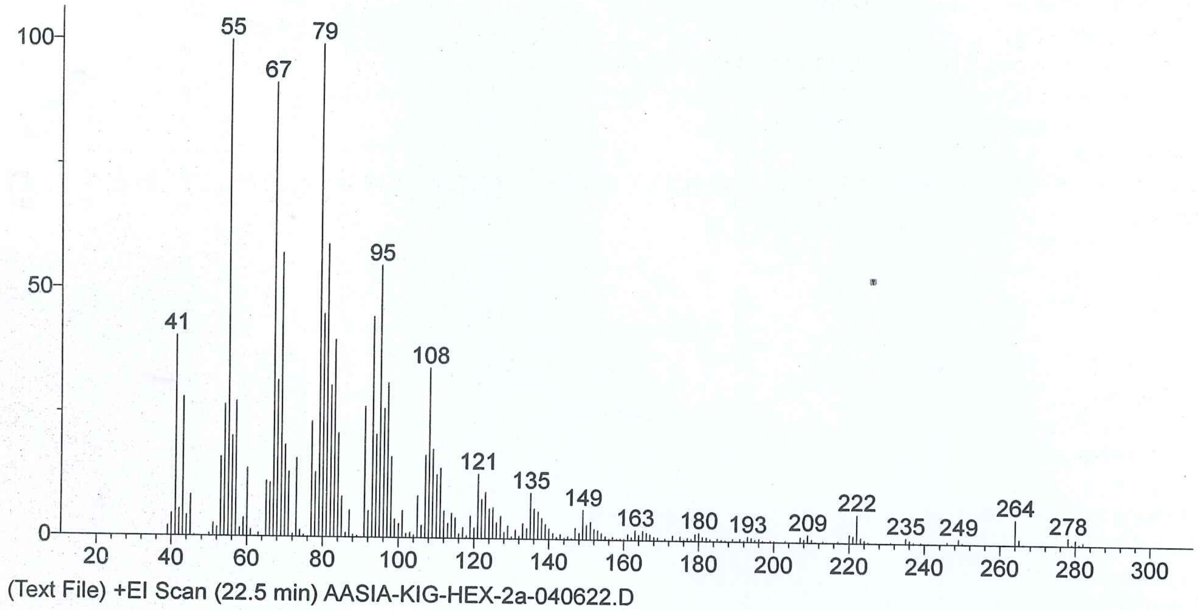

(Text File) +EI Scan (22.5 min) AASIA-KIG-HEX-2a-040622.D

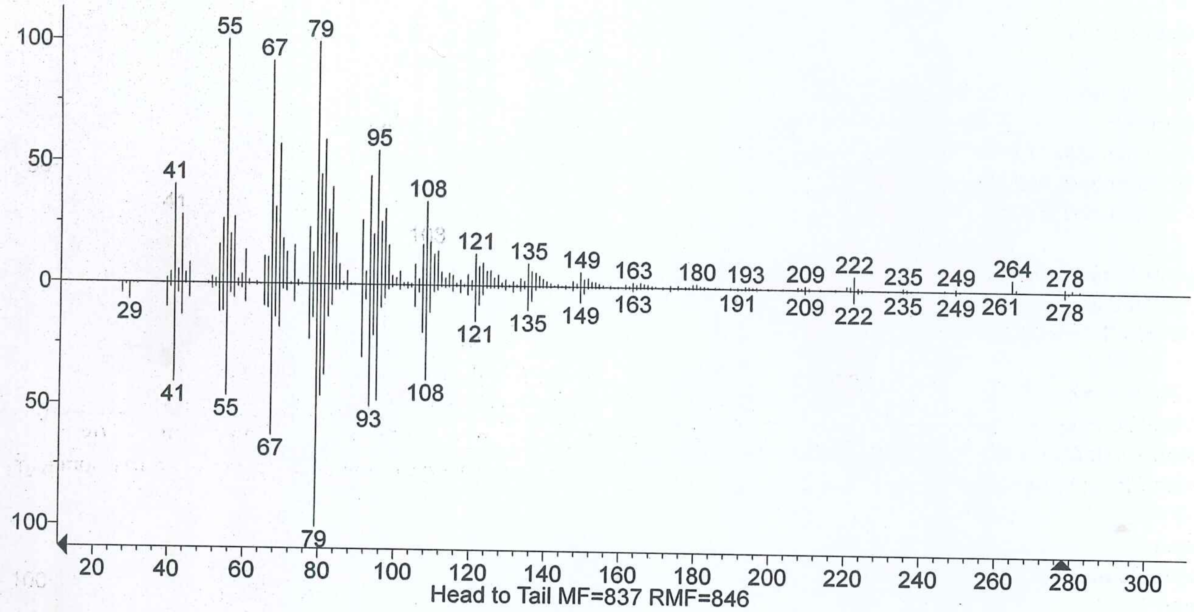

Head to Tail MF=837 RMF=846

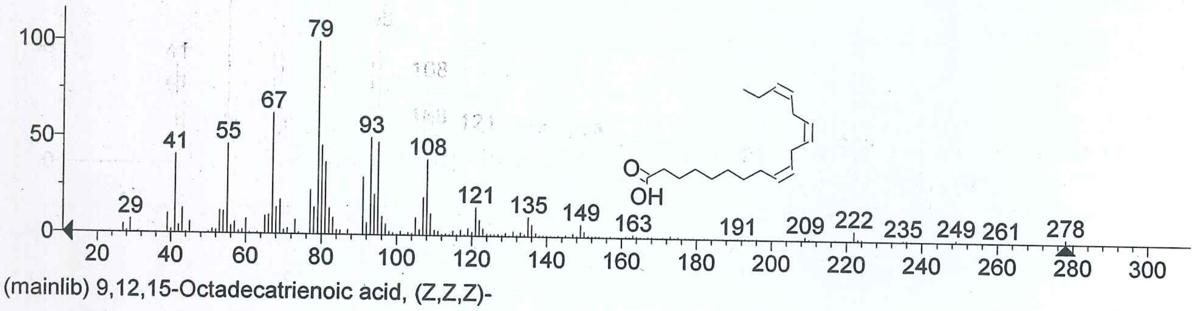

(mainlib) 9,12,15-Octadecatrienoic acid, (Z,Z,Z)-

Name: 9,12,15-Octadecatrienoic acid, (Z,Z,Z)-
Formula: $C_{18}H_{30}O_2$
MW: 278 CAS#: 463-40-1 NIST#: 333201 ID#: 41695 DB: mainlib
Other DBs: Fine, TSCA, HODOC, NIH, EINECS
Contributor: NIST Mass Spectrometry Data Center
10 largest peaks:
79 999 | 67 624 | 93 503 | 95 478 | 55 463 | 80 460 | 41 408 | 108 391 | 81 373 | 91 299 |
Synonyms:
1.Linolenic acid
2.α-Linolenic acid
3.All-cis-9,12,15-Octadecatrienoic acid
4.cis,cis,cis-9,12,15-Octadecatrienoic acid
5.(Z,Z,Z)-9,12,15-Octadecatrienoic acid
6.Industrene 120

Estimated non-polar retention index (n-alkane scale):
Value: 2191 iu
Confidence interval (Carboxylic acids): 51(50%) 220(95%) iu

Retention index.
1. Value: 2102 iu
Column Type: Capillary
Column Class: Standard non-polar
Active Phase: HP-101
Column
Length: 25 m
Carrier Gas: He
Column Diameter: 0.2 mm
Phase Thickness: 0.2 um
Data Type: Normal alkane
RI
Program Type: Ramp
Start T: 70 C
End T: 200 C
Heat Rate: 3 K/min
Start Time: 2 min
Source: Jerkovic, I.;
Mastelic, J.; Marijanovic, Z., Bound volatile compounds and essential oil from the fruit of Maclura pomifera (Raf.) Schneid. (osage orange), Flavour Fragr. J., 22, 2007, 84-88.

2. Value: 2178 iu
Column Type: Other
Column
Class: Standard non-polar
Active Phase: Methyl Silicone
Data Type: Normal alkane RI
Program Type:
Ramp
Source: Ardrey, R.E.; Moffat, A.C., Gas-liquid chromatographic retention indices of 1318 substances of toxicological interest on SE-30 or OV-1 stationary phase, J. Chromatogr., 220, 1981, 195-252.

<...>

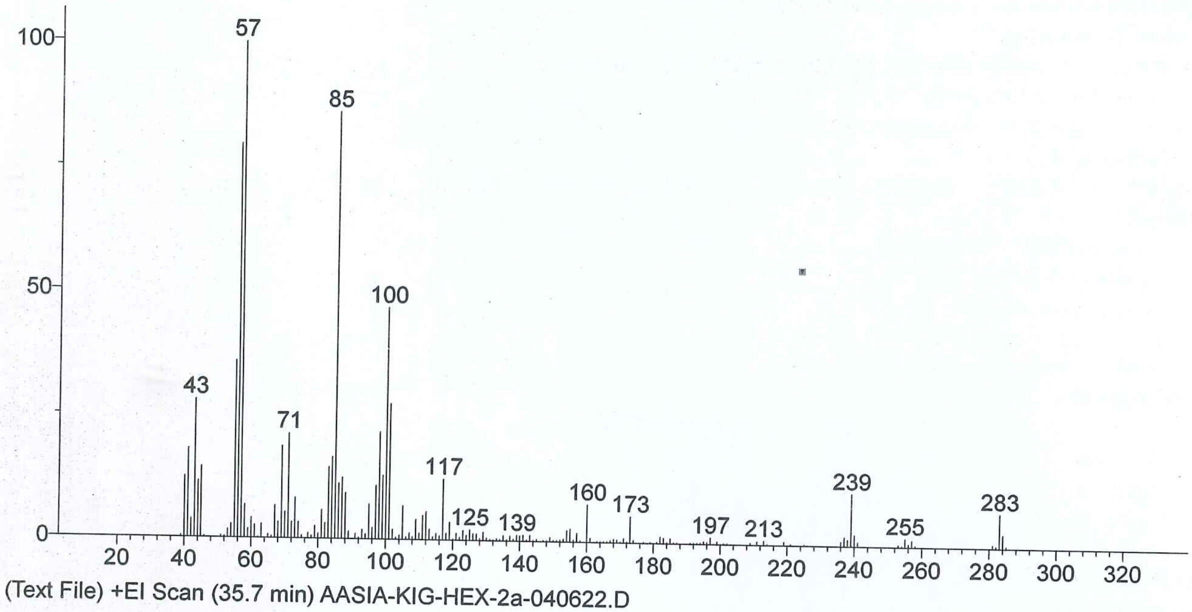

(Text File) +EI Scan (35.7 min) AASIA-KIG-HEX-2a-040622.D

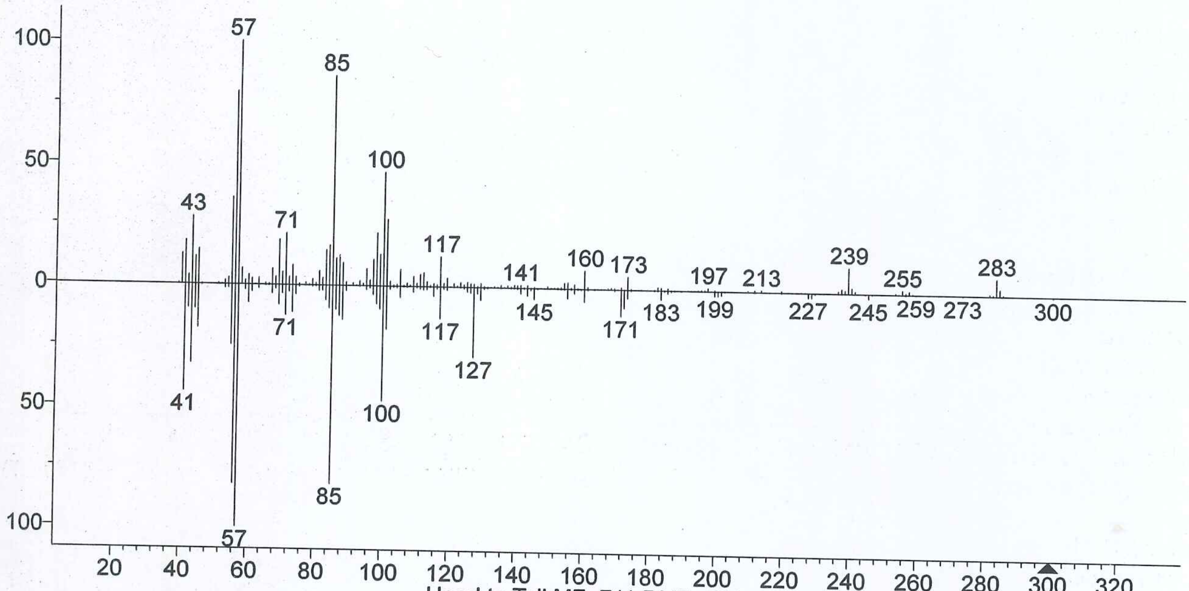

Head to Tail MF=711 RMF=766

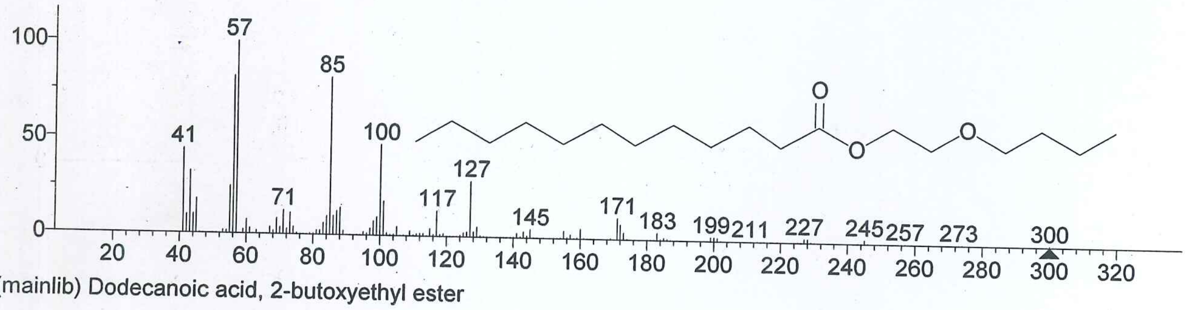

(mainlib) Dodecanoic acid, 2-butoxyethyl ester

Name: Dodecanoic acid, 2-butoxyethyl ester
Formula: $C_{18}H_{36}O_3$
MW: 300 CAS#: 109-37-5 NIST#: 127627 ID#: 22474 DB: mainlib
Other DBs: TSCA, NIH, EINECS
Contributor: Leung Pu, NIH, Bethesda, Maryland, U.S.
10 largest peaks:
57 999 | 56 819 | 85 817 | 100 472 | 41 440 | 43 325 | 127 288 | 55 248 | 45 180 | 101 178 |
Synonyms:
1.Ethylene glycol monobutyl ether laurate
2.Lauric acid, 2-butoxyethyl ester
3.2-Butoxyethyl laurate #

Estimated non-polar retention index (n-alkane scale):
Value: 2054 iu
Confidence interval (Diverse functional groups): 89(50%) 382(95%) iu

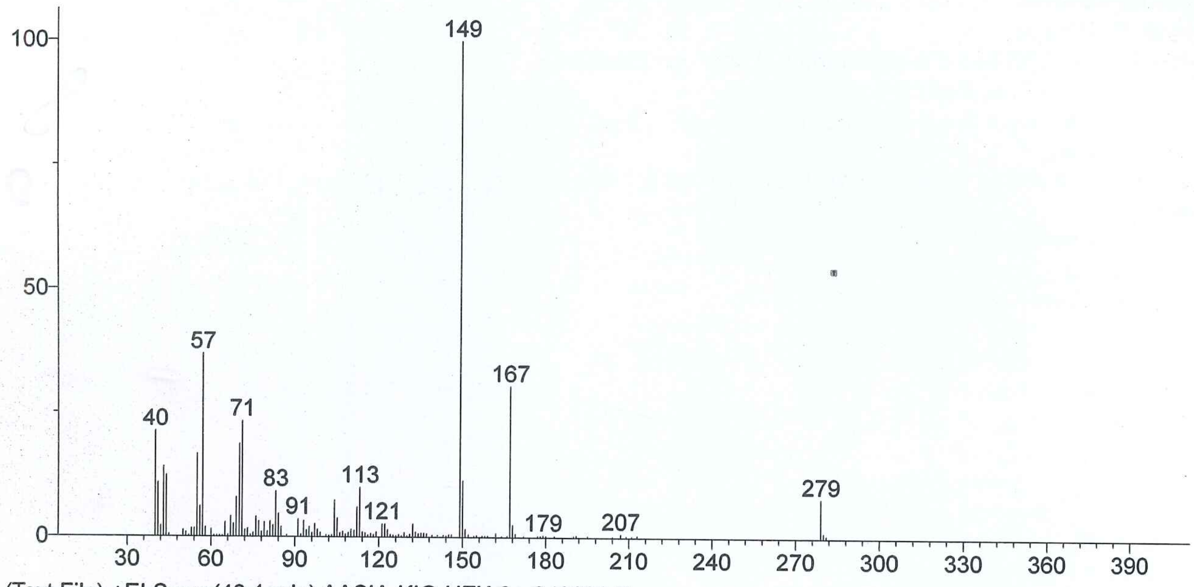

(Text File) +EI Scan (40.1 min) AASIA-KIG-HEX-2a-040622.D

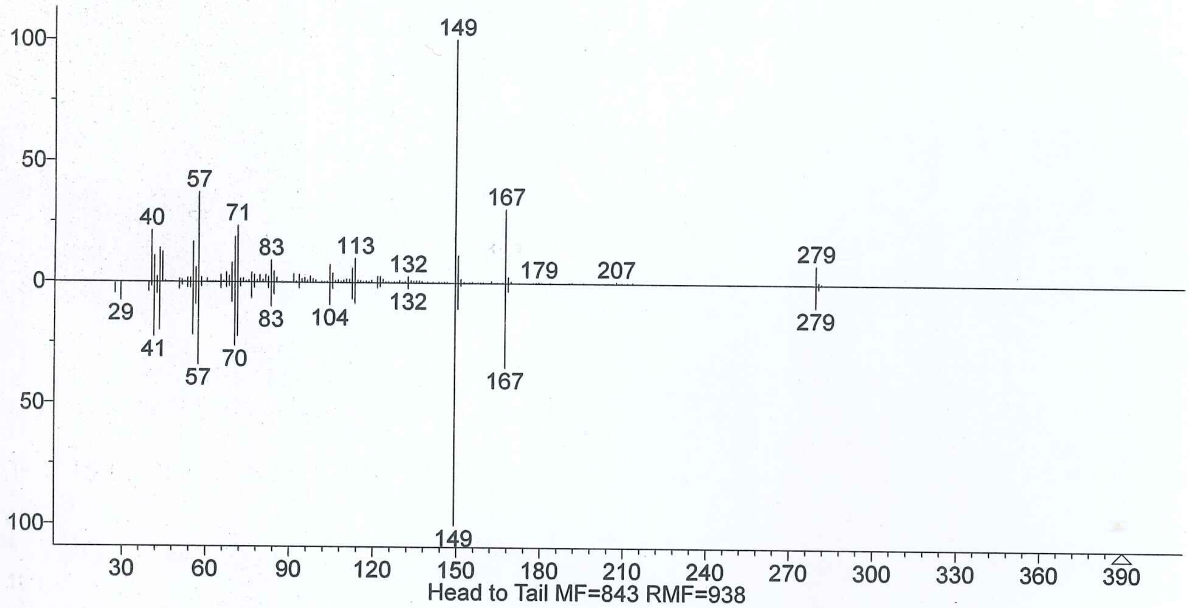

Head to Tail MF=843 RMF=938

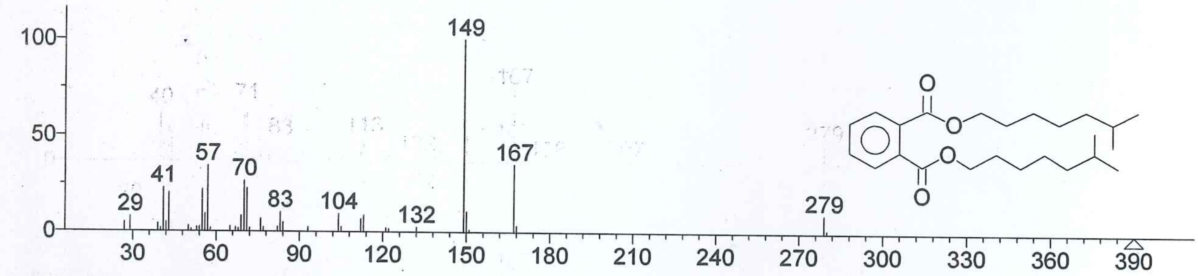

(replib) 1,2-Benzenedicarboxylic acid, diisooctyl ester

Name: 1,2-Benzenedicarboxylic acid, diisooctyl ester
Formula: $C_{24}H_{38}O_4$
MW: 390 CAS#: 27554-26-3 NIST#: 113206 ID#: 20061 DB: replib
Other DBs: Fine, TSCA, RTECS, EINECS, IRDB
Contributor: NIST Mass Spectrometry Data Center, 1990.
10 largest peaks:
149 999 | 167 350 | 57 341 | 70 264 | 41 225 | 71 224 | 55 218 | 43 200 | 150 107 | 83 100 |
Synonyms:
1.Diisooctyl phthalate
2.Hexaplas M/O
3.Isooctyl phthalate
4.Corflex 880
5.DIOP
6.Flexol plasticizer diop
7.Morflex 100
8.Palatinol D10
9.Phthalic acid, bis(6-methylheptyl) ester
10.Phthalic acid, diisooctyl ester
11.Witcizer 313
12.Bis(6-methylheptyl) phthalate #

Estimated non-polar retention index (n-alkane scale):
Value: 2704 iu
Confidence interval (Esters): 47(50%) 201(95%) iu

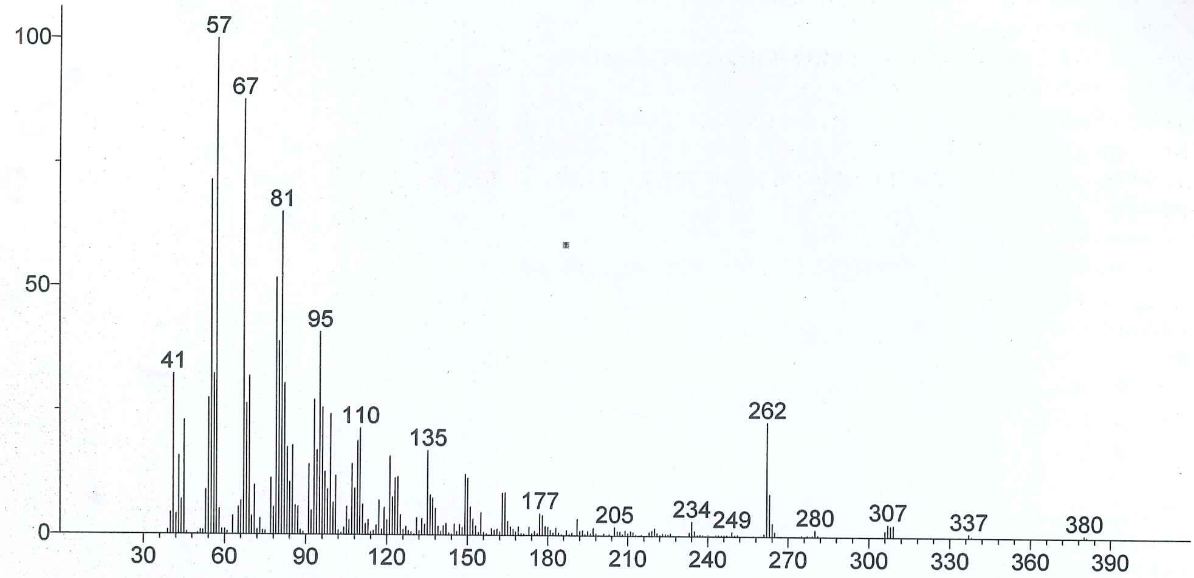

(Text File) +EI Scan (41.8 min) AASIA-KIG-HEX-2a-040622.D

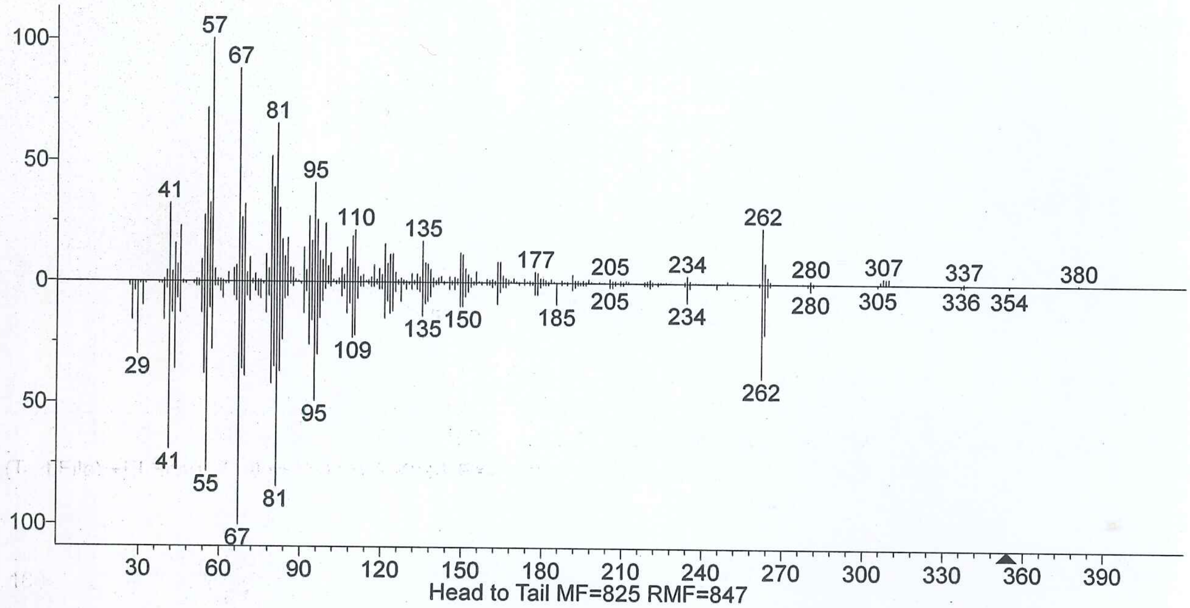

Head to Tail MF=825 RMF=847

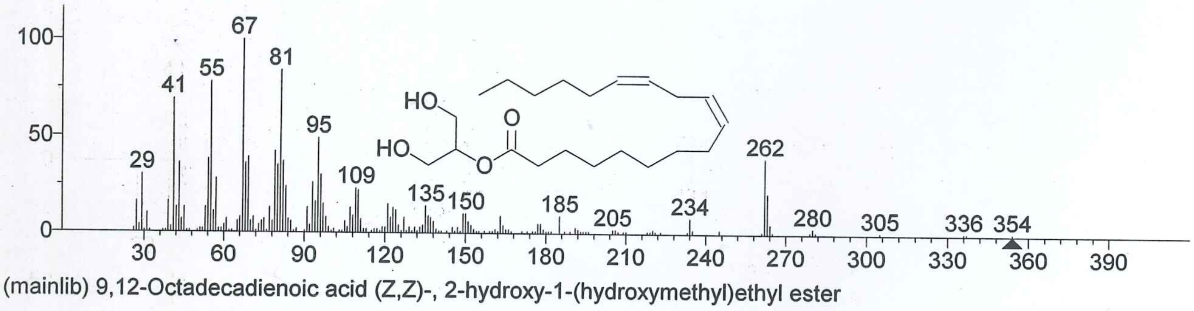

(mainlib) 9,12-Octadecadienoic acid (Z,Z)-, 2-hydroxy-1-(hydroxymethyl)ethyl ester

Name: 9,12-Octadecadienoic acid (Z,Z)-, 2-hydroxy-1-(hydroxymethyl)ethyl ester
Formula: $C_{21}H_{38}O_4$
MW: 354 CAS#: 3443-82-1 NIST#: 16013 ID#: 28833 DB: mainlib
Other DBs: None
10 largest peaks:
67 999 | 81 840 | 55 780 | 41 690 | 95 490 | 79 420 | 69 390 | 262 390 | 54 380 | 82 370 |
Synonyms:
1.Linolein, 2-mono-
2.β-Monolinolein
3.2-Hydroxy-1-(hydroxymethyl)ethyl (9Z,12Z)-9,12-octadecadienoate #

Estimated non-polar retention index (n-alkane scale):
Value: 2713 iu
Confidence interval (Diverse functional groups): 89(50%) 382(95%) iu

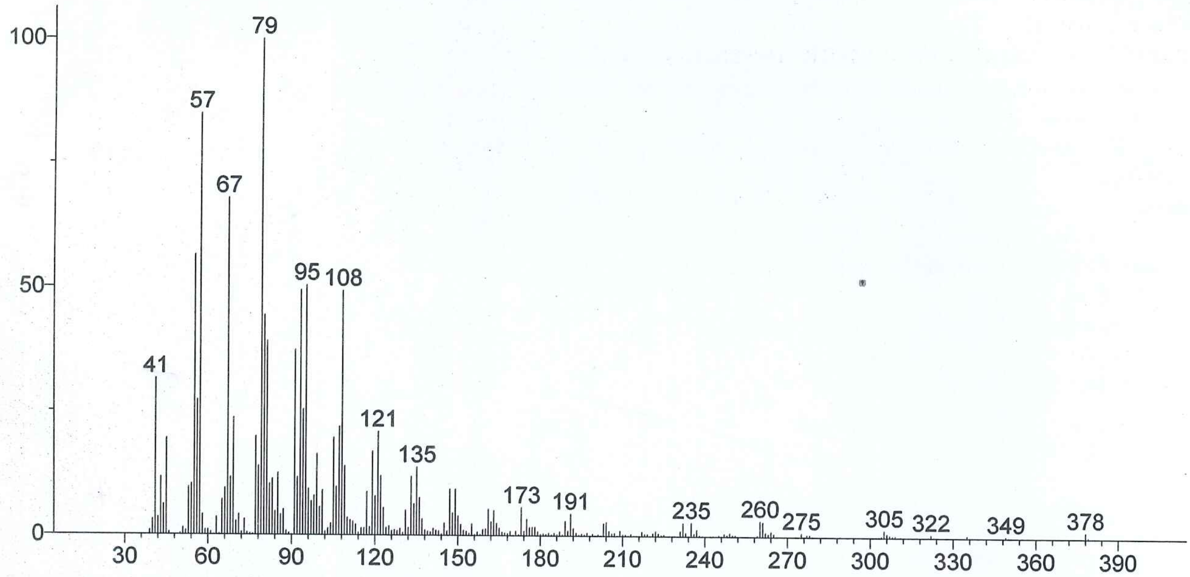

(Text File) +EI Scan (41.9 min) AASIA-KIG-HEX-2a-040622.D

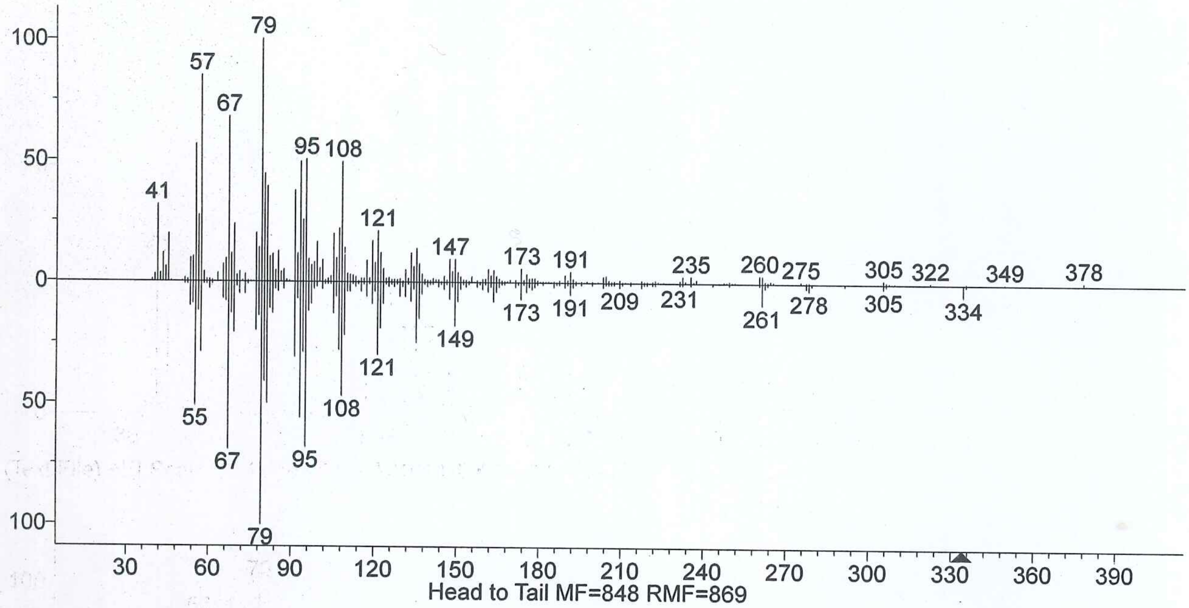

Head to Tail MF=848 RMF=869

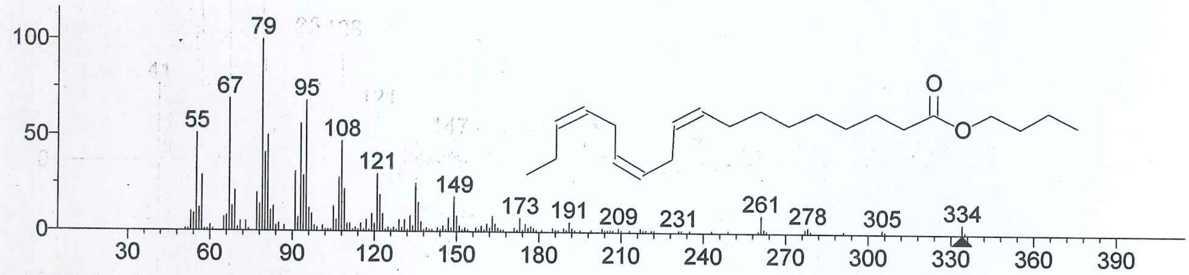

(mainlib) Butyl 9,12,15-octadecatrienoate

Name: Butyl 9,12,15-octadecatrienoate
Formula: $C_{22}H_{38}O_2$
MW: 334 NIST#: 336546 ID#: 41708 DB: mainlib
Contributor: William W. Christie, Mylnefield Lipid Analysis, Invergowrie, Dundee, Scotland, UK
10 largest peaks:
79 999 | 67 689 | 95 679 | 93 559 | 55 509 | 81 499 | 108 469 | 80 409 | 91 309 | 121 299 |
Synonyms:
no synonyms.

Estimated non-polar retention index (n-alkane scale):
Value: 2399 iu
Confidence interval (Esters): 47(50%) 201(95%) iu

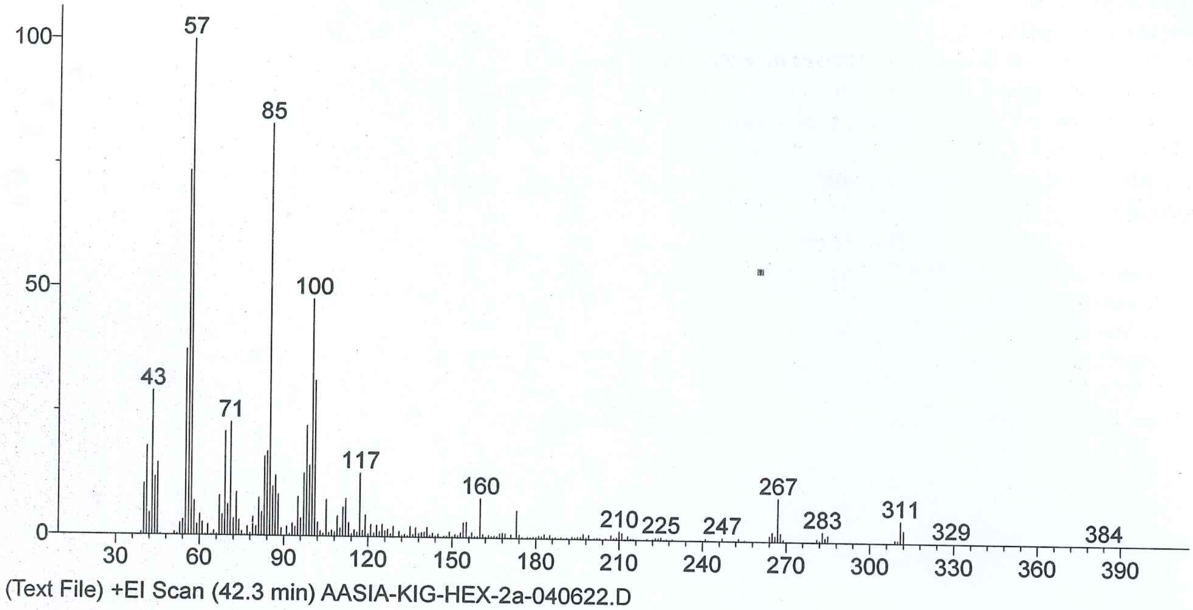

(Text File) +EI Scan (42.3 min) AASIA-KIG-HEX-2a-040622.D

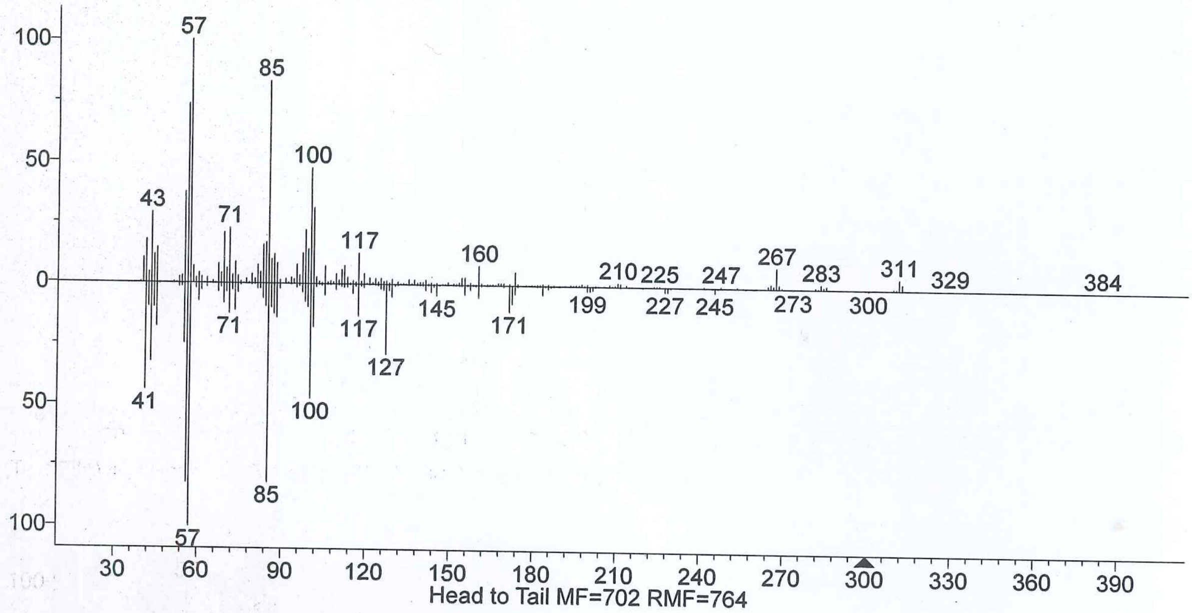

Head to Tail MF=702 RMF=764

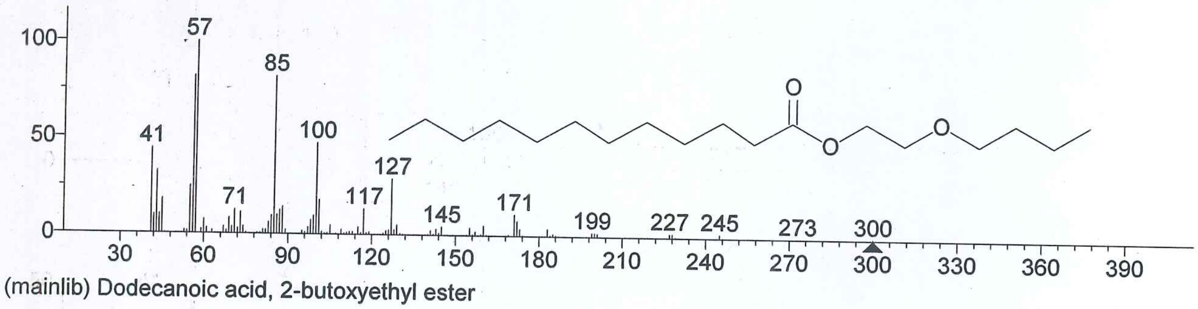

(mainlib) Dodecanoic acid, 2-butoxyethyl ester

Name: Dodecanoic acid, 2-butoxyethyl ester
Formula: $C_{18}H_{36}O_3$
MW: 300 CAS#: 109-37-5 NIST#: 127627 ID#: 22474 DB: mainlib
Other DBs: TSCA, NIH, EINECS
Contributor: Leung Pu, NIH, Bethesda, Maryland, U.S.
10 largest peaks:
57 999 | 56 819 | 85 817 | 100 472 | 41 440 | 43 325 | 127 288 | 55 248 | 45 180 | 101 178 |
Synonyms:
1.Ethylene glycol monobutyl ether laurate
2.Lauric acid, 2-butoxyethyl ester
3.2-Butoxyethyl laurate #

Estimated non-polar retention index (n-alkane scale):
Value: 2054 iu
Confidence interval (Diverse functional groups): 89(50%) 382(95%) iu

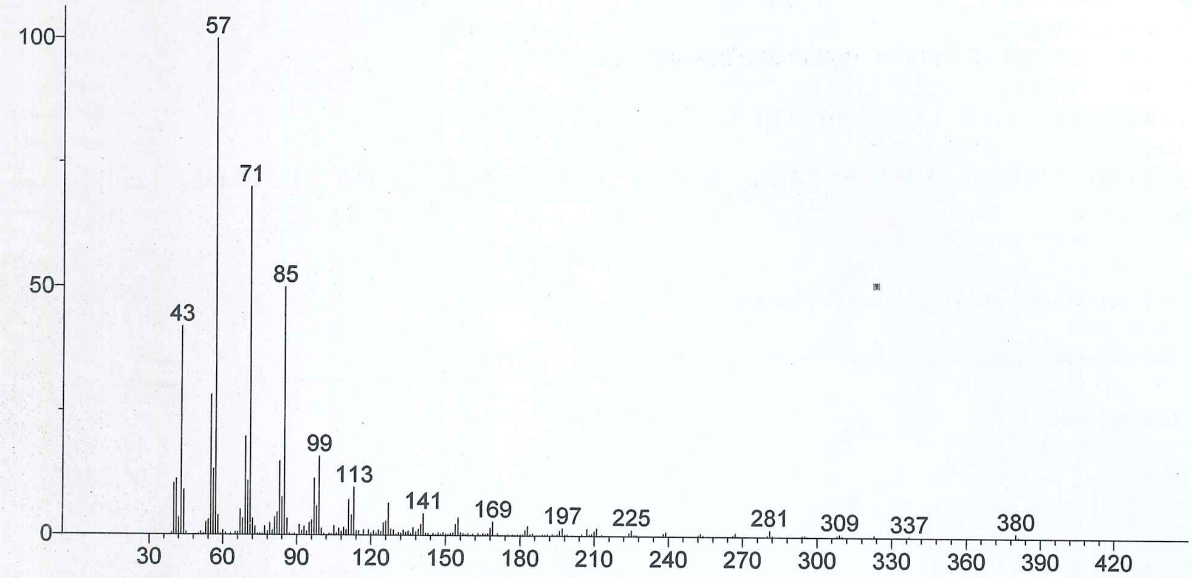

(Text File) +EI Scan (43.1 min) AASIA-KIG-HEX-2a-040622.D

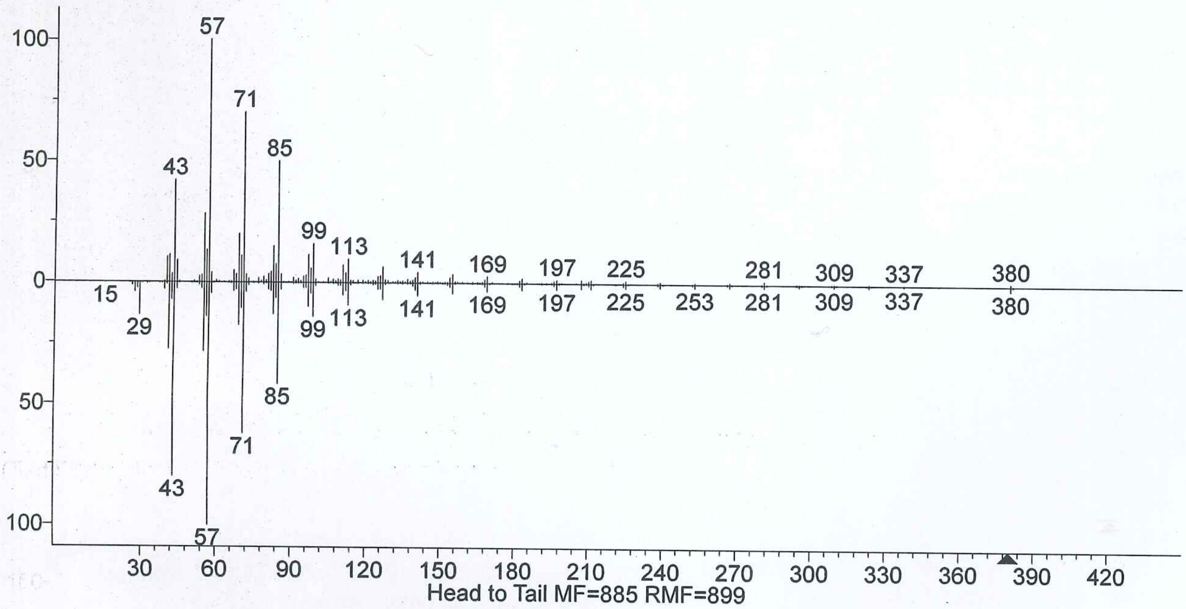

Head to Tail MF=885 RMF=899

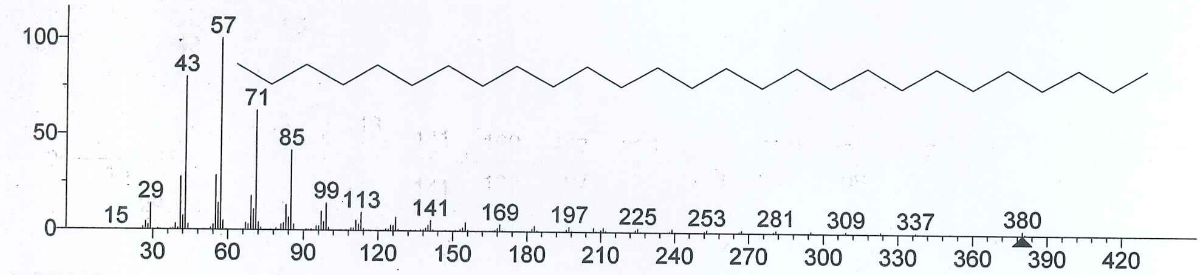

(replib) Heptacosane

Name: Heptacosane
Formula: $C_{27}H_{56}$
MW: 380 CAS#: 593-49-7 NIST#: 79427 ID#: 5508 DB: replib
Other DBs: Fine, EPA, HODOC, EINECS
Contributor: O A MAMER, MCGILL UNIVERSITY, MONTREAL, CANA
10 largest peaks:
57 999 | 43 798 | 71 622 | 85 416 | 55 283 | 41 275 | 69 176 | 99 140 | 56 139 | 29 135 |
Synonyms:
1.n-Heptacosane

Estimated non-polar retention index (n-alkane scale):
Value: 2705 iu
Confidence interval (Hydrocarbons): 39(50%) 167(95%) iu

Retention index.
1. Value: 2700 iu
Column Class: All column types
Data Type: Normal alkane RI value specified by scale definition
Source: von Kováts, E., 206. Gas-chromatographische Charakterisierung organischer Verbindungen. Teil 1: Retentionsindices aliphatischer Halogenide, Alkohole, Aldehyde und Ketone, Helv. Chim. Acta, 41(7), 1958, 1915 -1932.

2. Value: 447.8 iu
Column Type: Capillary
Column Class: Standard non-polar
Active Phase: DB
-1
Column Length: 30 m
Column Diameter: 0.2 mm
Phase Thickness: 0.25 um
Data Type: Lee RI
Program
Type: Ramp
Start T: 50 C
End T: 300 C
Heat Rate: 5 K/min
Start Time: 2 min
End Time: 5 min
Source:
Johnson, C.I.; Urso, A.; Geleta, L., Broad spectrum analysis of municipal and industrial effluents discharged into the Peace, Athabasca and Slave river basins: characterization of effluent samples, 1994 - Volume 1 of 2, Northern River Basins Study Project Report No. 121, Norther River Basins Study, Edmonton, Alberta, 1997, 27.

<...>

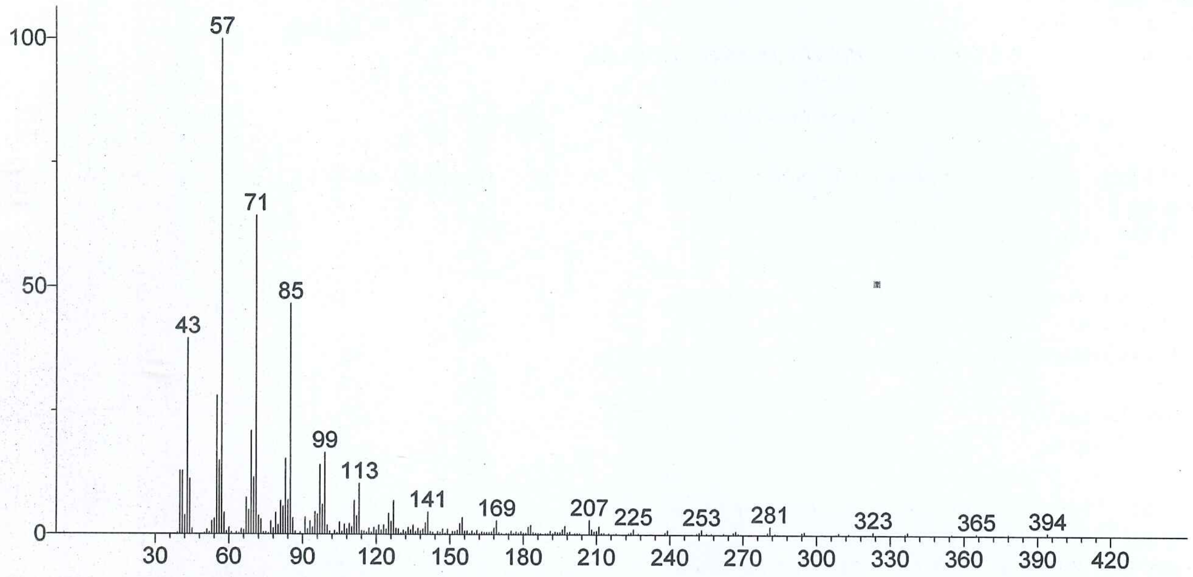

(Text File) +EI Scan (44.3 min) AASIA-KIG-HEX-2a-040622.D

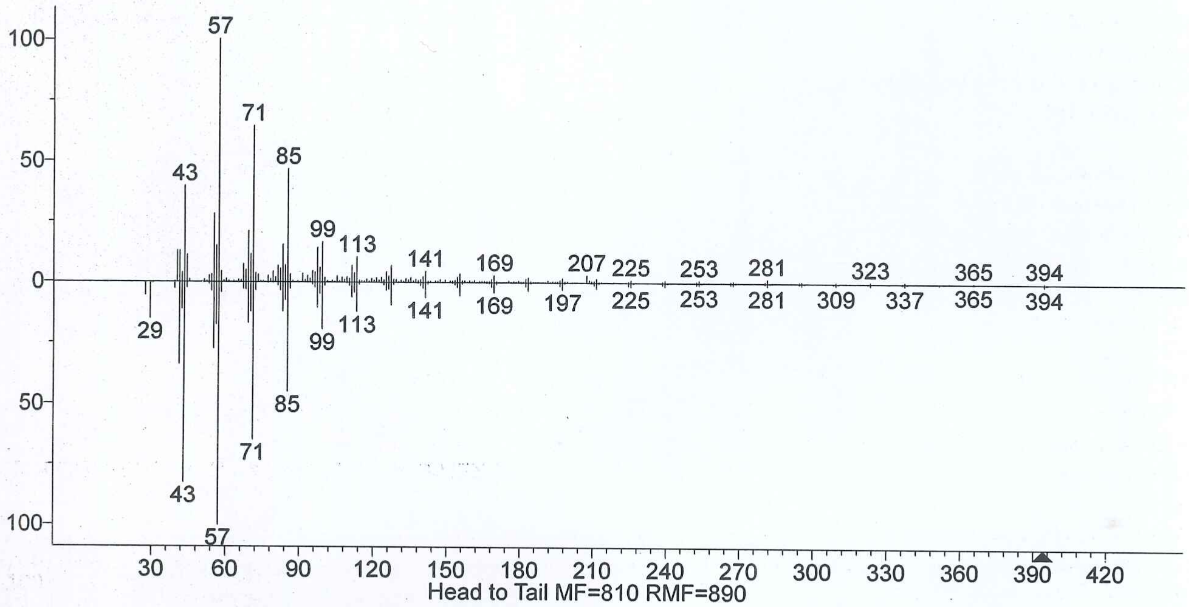

Head to Tail MF=810 RMF=890

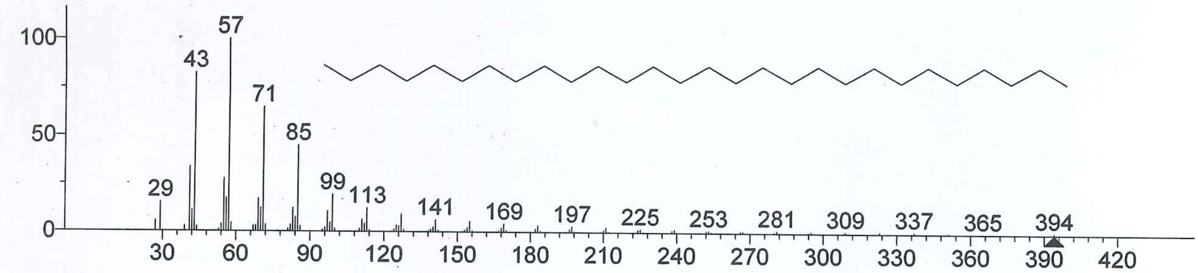

(replib) Octacosane

Name: Octacosane
Formula: $C_{28}H_{58}$
MW: 394 CAS#: 630-02-4 NIST#: 134306 ID#: 5471 DB: replib
Other DBs: Fine, TSCA, EPA, HODOC, NIH, EINECS, IRDB
Contributor: NIST Mass Spectrometry Data Center, 1994
10 largest peaks:
57 999 | 43 823 | 71 647 | 85 449 | 41 337 | 55 274 | 99 193 | 56 174 | 69 168 | 29 151 |
Synonyms:
1.n-Octacosane

Estimated non-polar retention index (n-alkane scale):
Value: 2804 iu
Confidence interval (Hydrocarbons): 39(50%) 167(95%) iu

Retention index.
1. Value: 2800 iu
Column Class: All column types
Data Type: Normal alkane RI value specified by scale definition
Source: von Kováts, E., 206. Gas-chromatographische Charakterisierung organischer Verbindungen. Teil 1: Retentionsindices aliphatischer Halogenide, Alkohole, Aldehyde und Ketone, Helv. Chim. Acta, 41(7), 1958, 1915 -1932.

2. Value: 461.8 iu
Column Type: Capillary
Column Class: Standard non-polar
Active Phase: DB
-1
Column Length: 30 m
Column Diameter: 0.2 mm
Phase Thickness: 0.25 um
Data Type: Lee RI
Program
Type: Ramp
Start T: 50 C
End T: 300 C
Heat Rate: 5 K/min
Start Time: 2 min
End Time: 5 min
Source:
Johnson, C.I.; Urso, A.; Geleta, L., Broad spectrum analysis of municipal and industrial effluents discharged into the Peace, Athabasca and Slave river basins: characterization of effluent samples, 1994 - Volume 1 of 2, Northern River Basins Study Project Report No. 121, Norther River Basins Study, Edmonton, Alberta, 1997, 27.

<...>

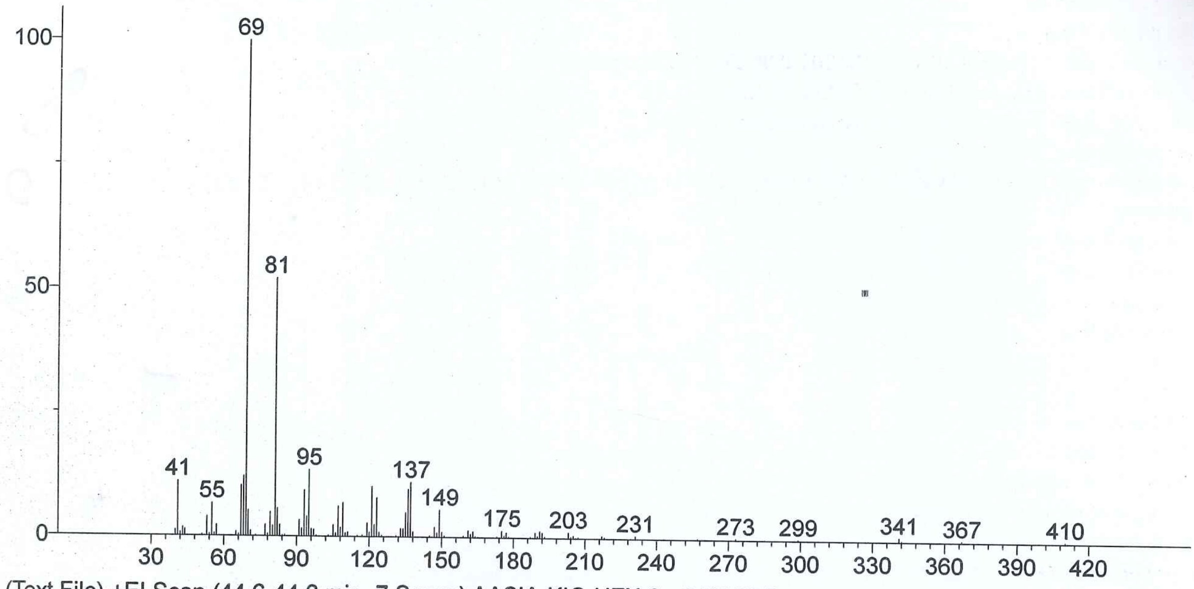

(Text File) +EI Scan (44.6-44.6 min, 7 Scans) AASIA-KIG-HEX-2a-040622.D

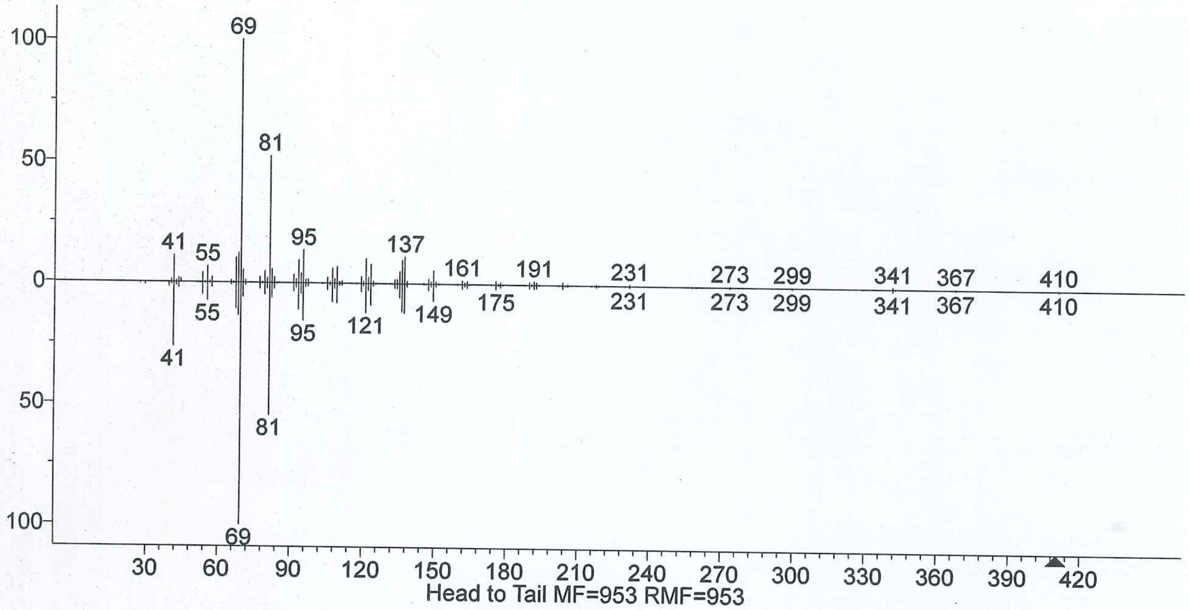

Head to Tail MF=953 RMF=953

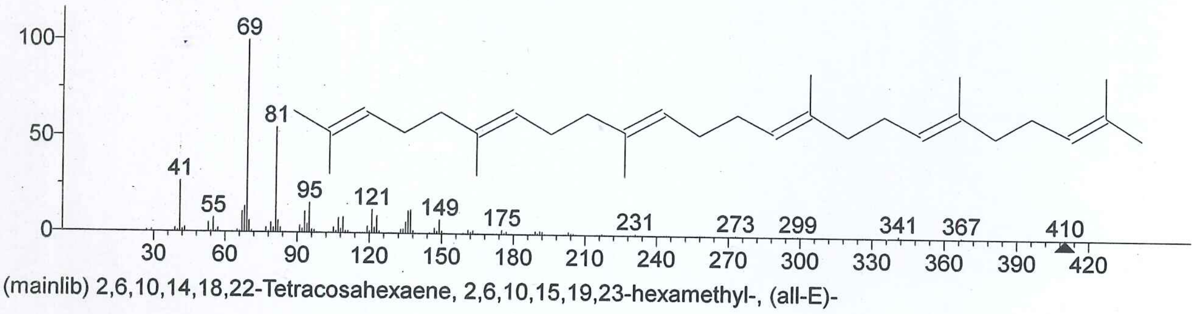

(mainlib) 2,6,10,14,18,22-Tetracosahexaene, 2,6,10,15,19,23-hexamethyl-, (all-E)-

Name: 2,6,10,14,18,22-Tetracosahexaene, 2,6,10,15,19,23-hexamethyl-, (all-E)-
Formula: $C_{30}H_{50}$
MW: 410 CAS#: 111-02-4 NIST#: 290792 ID#: 30950 DB: mainlib
Other DBs: Fine, TSCA, RTECS, HODOC, EINECS
Contributor: NIST Mass Spectrometry Data Center, 1998.
10 largest peaks:
69 999 | 81 545 | 41 264 | 95 155 | 68 134 | 121 121 | 137 121 | 136 116 | 93 108 | 67 107 |
Synonyms:
1.All-trans-Squalene
2.trans-Squalene
3.Spinacen
4.Spinacene
5.Squalen
6.Squalene
7.Supraene
8.2,6,10,15,19,23-Hexamethyl-2,6,10,14,18,22-Tetracosahexaene
9.2,6,10,15,19,23-Hexamethyltetracosa-2,6,10,14,18,22-hexaene
10.(All-E)-2,6,10,15,19,23-hexamethyl-2,6,10,14,18,22-tetracosahexaene
11.trans-Spinacene
12.(6E,10E,14E,18E)-2,6,10,15,19,23-Hexamethyl-2,6,10,14,18,22-tetracosahexaene #

Estimated non-polar retention index (n-alkane scale):
Value: 2914 iu
Confidence interval (Hydrocarbons): 39(50%) 167(95%) iu

Retention index.
1. Value: 2847.1 iu
Column Type: Capillary
Column Class: Semi-standard non-polar
Active Phase: HP
-5MS
Column Length: 30 m
Carrier Gas: He
Column Diameter: 0.25 mm
Phase Thickness: 0.25 um
Data Type:
Linear RI
Program Type: Ramp
Start T: 80 C
End T: 300 C
Heat Rate: 4 K/min
Source: Zhao C.X.; Li, X.N.;
Liang Y.Z.; Fang H.Z.; Huang L.F.; Guo F.Q., Comparative analysis of chemical components of essential oils from different samples of Rhododendron with the help of chemometrics methods, Chemom. Intell. Lab. Syst., 82, 2006, 218-228.

2. Value: 2809.1 iu
Column Type: Capillary
Column Class: Semi-standard non-polar
Active Phase:
DB-5
Column Length: 30 m
Carrier Gas: He
Column Diameter: 0.32 mm

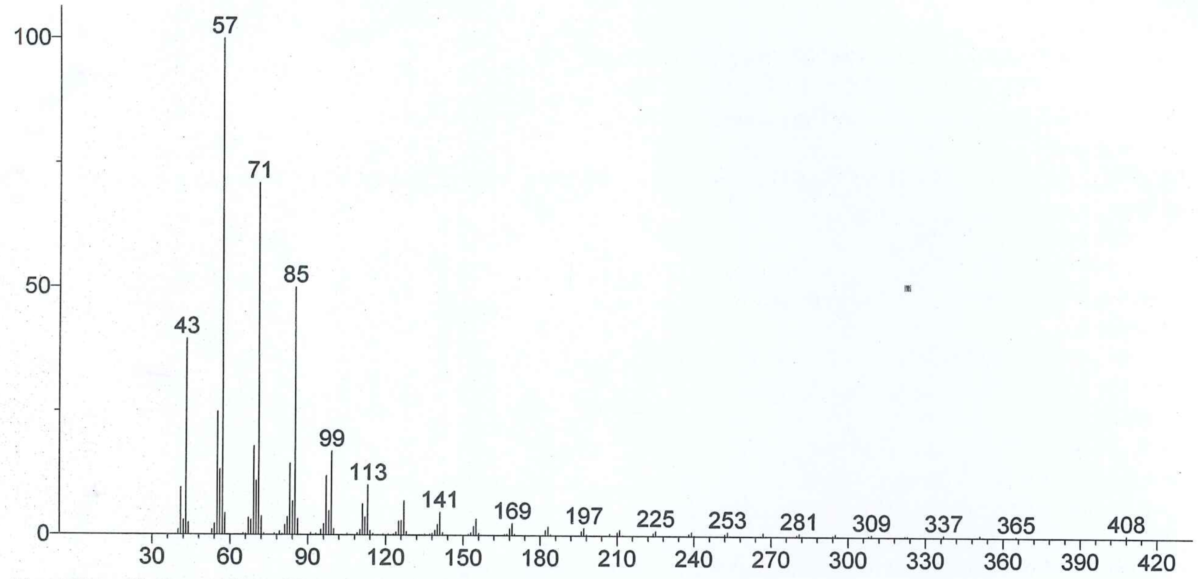

(Text File) +EI Scan (45.3-45.4 min, 13 Scans) AASIA-KIG-HEX-2a-040622.D

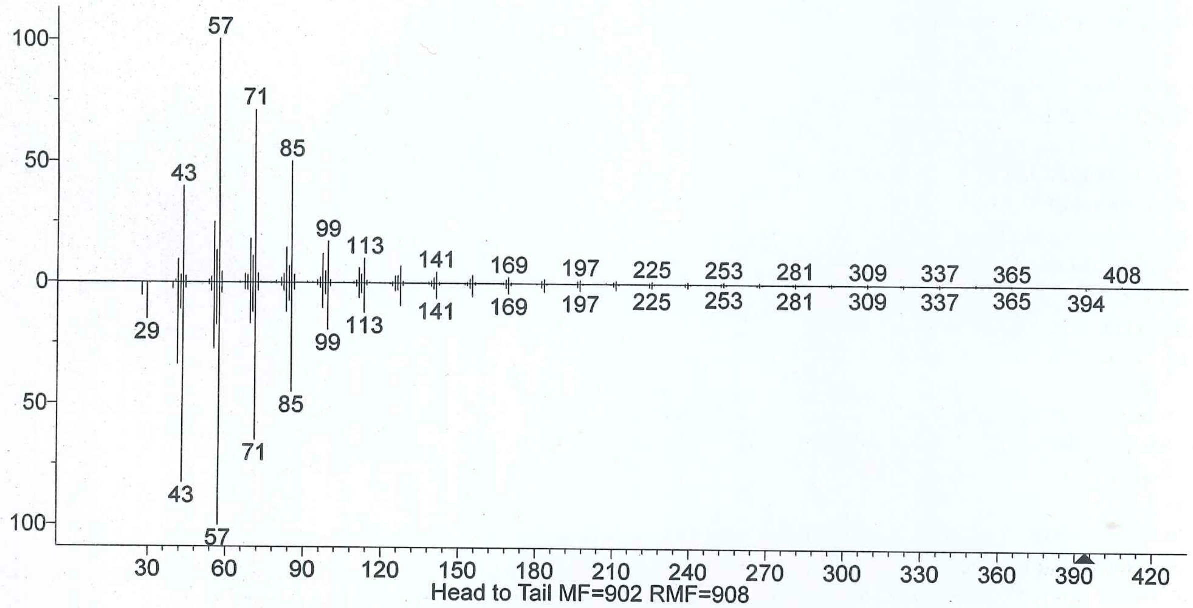

Head to Tail MF=902 RMF=908

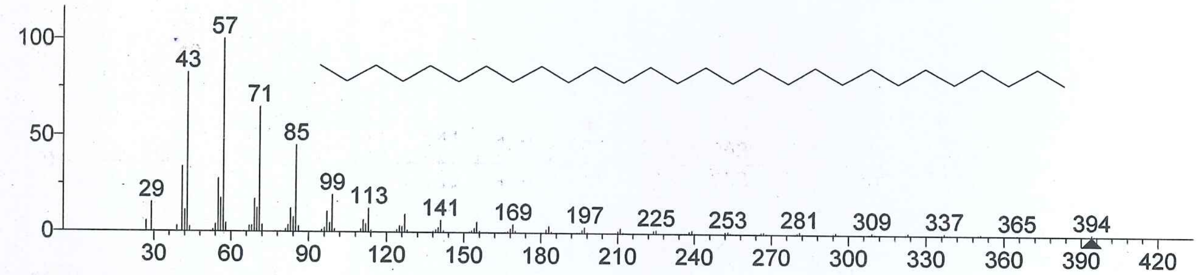

(replib) Octacosane

Name: Octacosane
Formula: $C_{28}H_{58}$
MW: 394 CAS#: 630-02-4 NIST#: 134306 ID#: 5471 DB: replib
Other DBs: Fine, TSCA, EPA, HODOC, NIH, EINECS, IRDB
Contributor: NIST Mass Spectrometry Data Center, 1994
10 largest peaks:
57 999 | 43 823 | 71 647 | 85 449 | 41 337 | 55 274 | 99 193 | 56 174 | 69 168 | 29 151 |
Synonyms:
1.n-Octacosane

Estimated non-polar retention index (n-alkane scale):
Value: 2804 iu
Confidence interval (Hydrocarbons): 39(50%) 167(95%) iu

Retention index.
1. Value: 2800 iu
Column Class: All column types
Data Type: Normal alkane RI value specified by scale definition
Source: von Kováts, E., 206. Gas-chromatographische Charakterisierung organischer Verbindungen. Teil 1: Retentionsindices aliphatischer Halogenide, Alkohole, Aldehyde und Ketone, Helv. Chim. Acta, 41(7), 1958, 1915 -1932.

2. Value: 461.8 iu
Column Type: Capillary
Column Class: Standard non-polar
Active Phase: DB
-1
Column Length: 30 m
Column Diameter: 0.2 mm
Phase Thickness: 0.25 um
Data Type: Lee RI
Program
Type: Ramp
Start T: 50 C
End T: 300 C
Heat Rate: 5 K/min
Start Time: 2 min
End Time: 5 min
Source:
Johnson, C.I.; Urso, A.; Geleta, L., Broad spectrum analysis of municipal and industrial effluents discharged into the Peace, Athabasca and Slave river basins: characterization of effluent samples, 1994 - Volume 1 of 2, Northern River Basins Study Project Report No. 121, Norther River Basins Study, Edmonton, Alberta, 1997, 27.

<...>

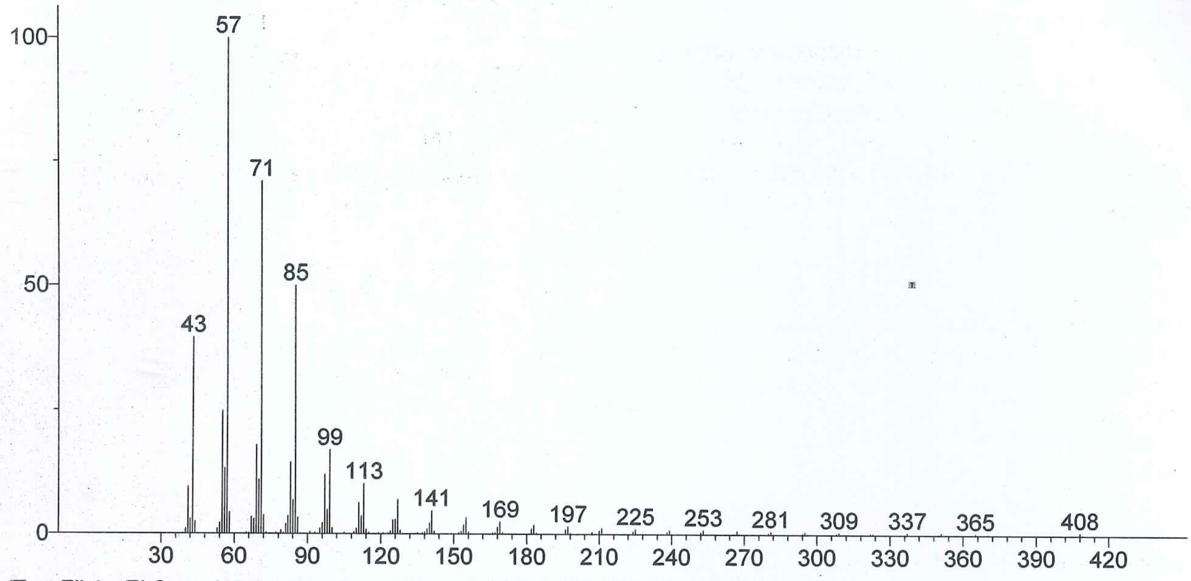

(Text File) +EI Scan (45.3-45.4 min, 13 Scans) AASIA-KIG-HEX-2a-040622.D

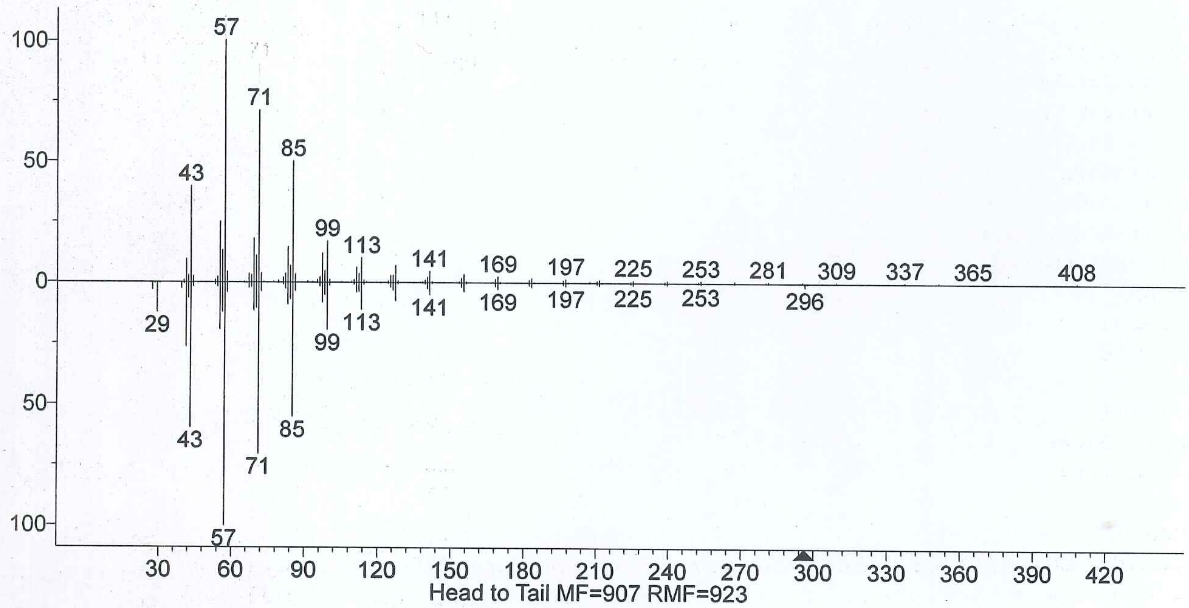

Head to Tail MF=907 RMF=923

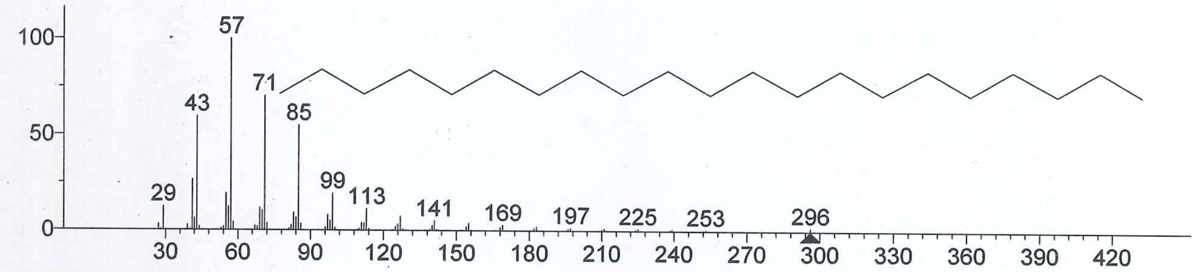

(replib) Heneicosane

Name: Heneicosane
Formula: $C_{21}H_{44}$
MW: 296 CAS#: 629-94-7 NIST#: 107569 ID#: 5753 DB: replib
Other DBs: Fine, TSCA, EPA, HODOC, EINECS, IRDB
Contributor: Chuck Anderson, Aldrich Chemical Co.
10 largest peaks:
57 999 | 71 702 | 43 594 | 85 549 | 41 263 | 55 193 | 99 193 | 29 122 | 56 122 | 69 117 |
Synonyms:
1.n-Heneicosane
2.Henicosane #

Estimated non-polar retention index (n-alkane scale):
Value: 2109 iu
Confidence interval (Hydrocarbons): 39(50%) 167(95%) iu

Retention index.
1. Value: 2100 iu
Column Class: All column types
Data Type: Normal alkane RI value specified by scale
definition
Source: von Kováts, E., 206. Gas-chromatographische Charakterisierung organischer Verbindungen. Teil
1: Retentionsindices aliphatischer Halogenide, Alkohole, Aldehyde und Ketone, Helv. Chim. Acta, 41(7), 1958, 1915
-1932.

2. Value: 360.4 iu
Column Type: Capillary
Column Class: Standard non-polar
Active Phase: DB
-1
Column Length: 30 m
Column Diameter: 0.2 mm
Phase Thickness: 0.25 um
Data Type: Lee RI
Program
Type: Ramp
Start T: 50 C
End T: 300 C
Heat Rate: 5 K/min
Start Time: 2 min
End Time: 5 min
Source:
Johnson, C.I.; Urso, A.; Geleta, L., Broad spectrum analysis of municipal and industrial effluents discharged into the Peace, Athabasca and Slave river basins: characterization of effluent samples, 1994 - Volume 1 of 2, Northern River Basins Study Project Report No. 121, Norther River Basins Study, Edmonton, Alberta, 1997, 27.

<...>

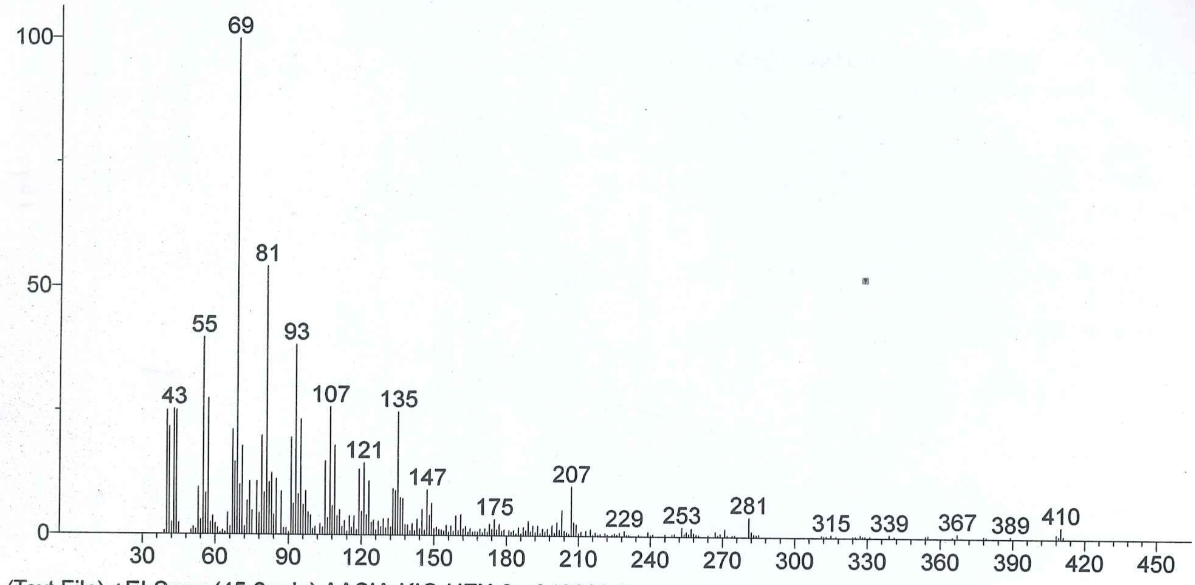

(Text File) +EI Scan (45.6 min) AASIA-KIG-HEX-2a-040622.D

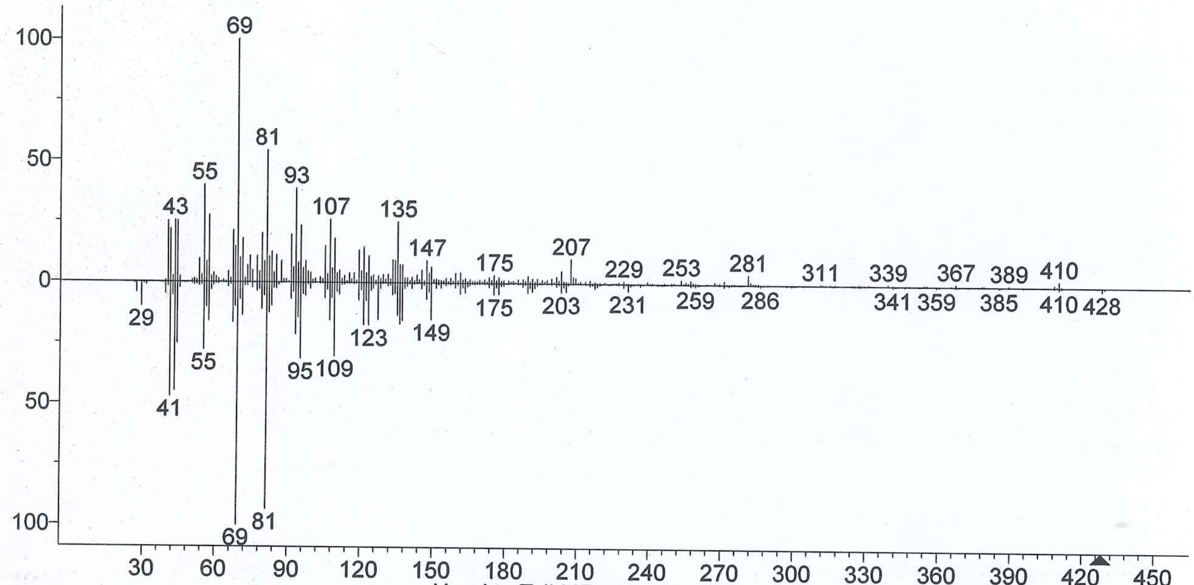

Head to Tail MF=772 RMF=784

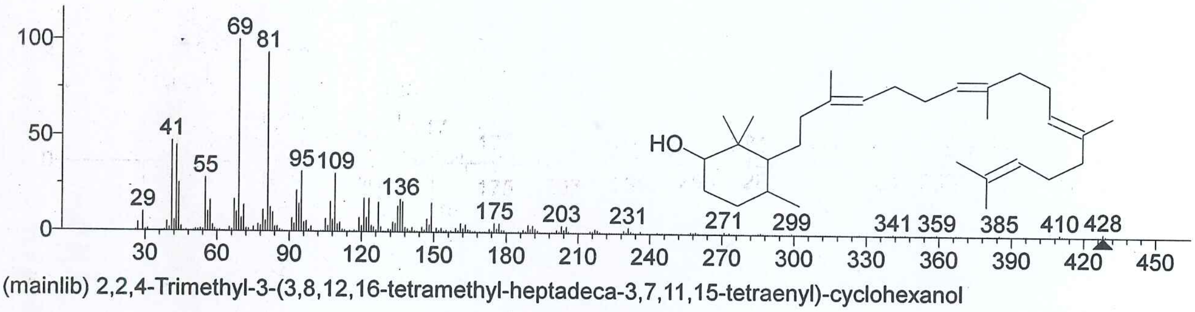

(mainlib) 2,2,4-Trimethyl-3-(3,8,12,16-tetramethyl-heptadeca-3,7,11,15-tetraenyl)-cyclohexanol

Name: 2,2,4-Trimethyl-3-(3,8,12,16-tetramethyl-heptadeca-3,7,11,15-tetraenyl)-cyclohexanol
Formula: $C_{30}H_{52}O$
MW: 428 NIST#: 194014 ID#: 30936 DB: mainlib
Contributor: Chemical Concepts
10 largest peaks:
69 999 | 81 933 | 41 470 | 43 447 | 95 313 | 109 302 | 55 277 | 44 251 | 93 213 | 123 174 |
Synonyms:
no synonyms.

Estimated non-polar retention index (n-alkane scale):
Value: 3093 iu
Confidence interval (Alcohols): 41(50%) 176(95%) iu

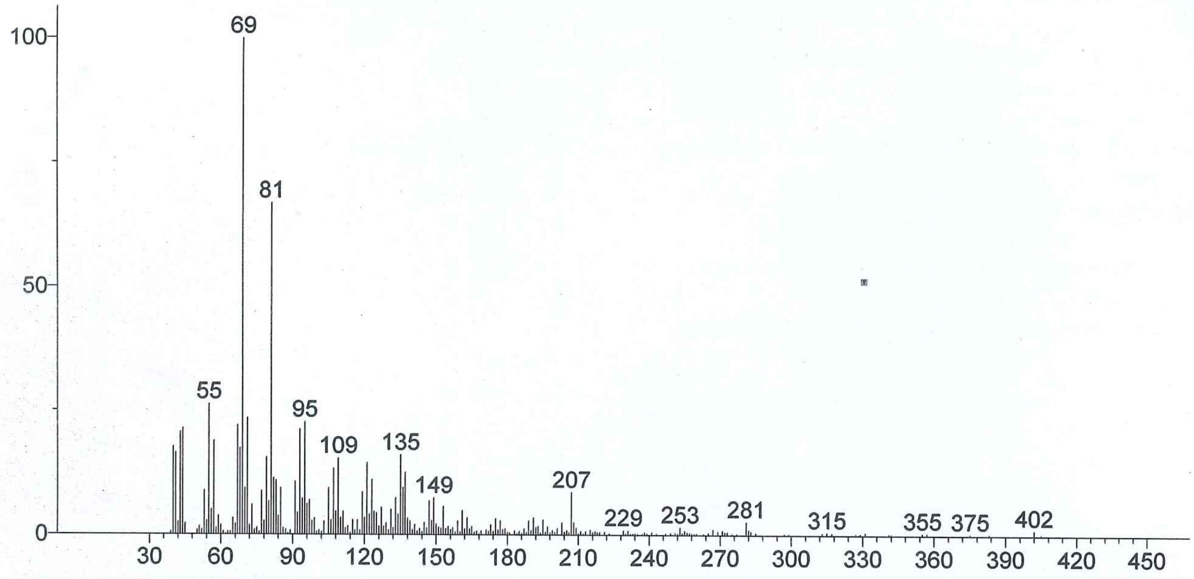

(Text File) +EI Scan (45.7 min) AASIA-KIG-HEX-2a-040622.D

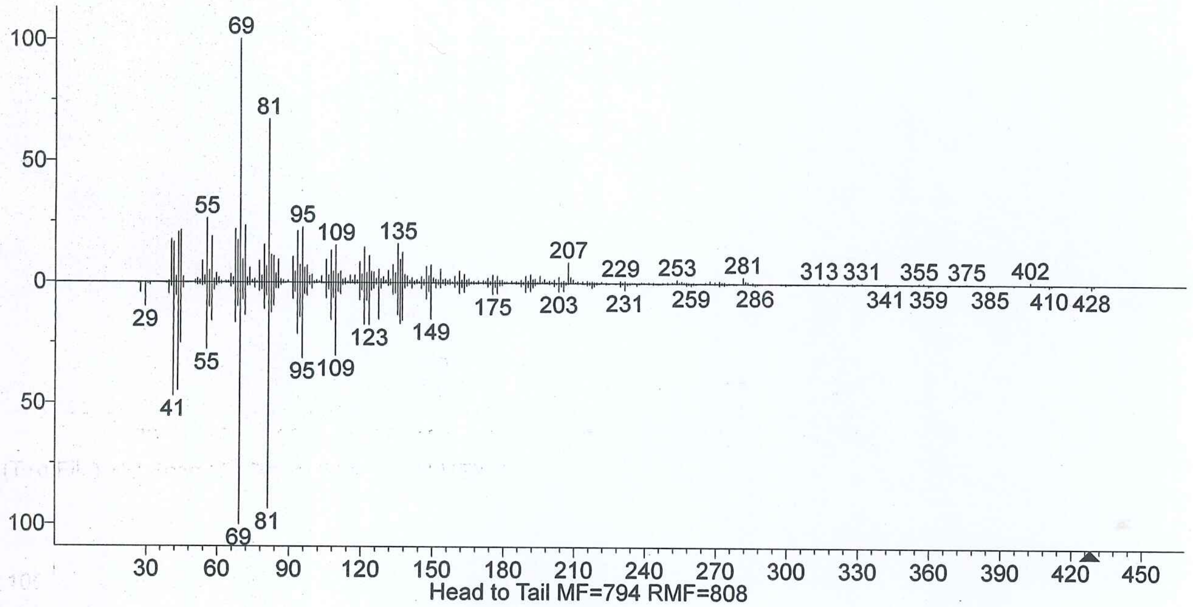

Head to Tail MF=794 RMF=808

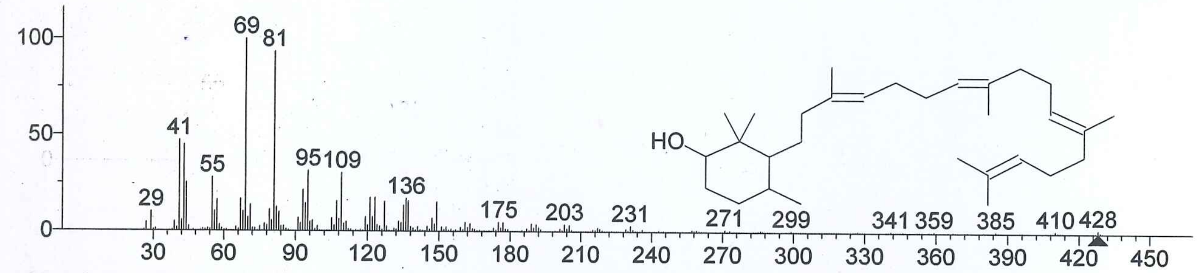

(mainlib) 2,2,4-Trimethyl-3-(3,8,12,16-tetramethyl-heptadeca-3,7,11,15-tetraenyl)-cyclohexanol

Name: 2,2,4-Trimethyl-3-(3,8,12,16-tetramethyl-heptadeca-3,7,11,15-tetraenyl)-cyclohexanol
Formula: $C_{30}H_{52}O$
MW: 428 NIST#: 194014 ID#: 30936 DB: mainlib
Contributor: Chemical Concepts
10 largest peaks:
69 999 | 81 933 | 41 470 | 43 447 | 95 313 | 109 302 | 55 277 | 44 251 | 93 213 | 123 174 |
Synonyms:
no synonyms.

Estimated non-polar retention index (n-alkane scale):
Value: 3093 iu
Confidence interval (Alcohols): 41(50%) 176(95%) iu

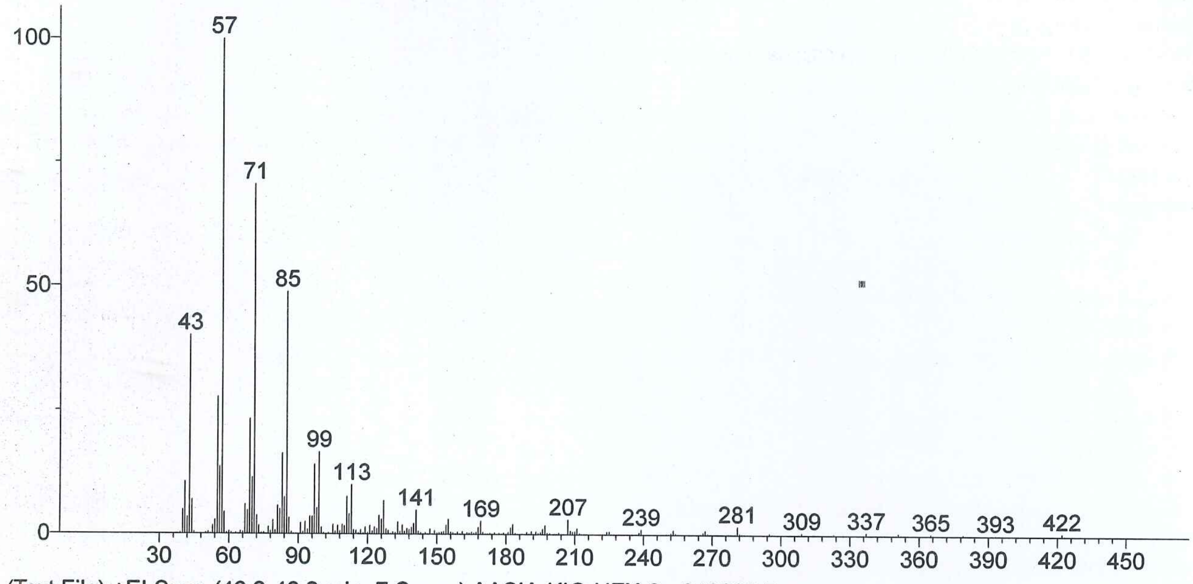

(Text File) +EI Scan (46.2-46.2 min, 7 Scans) AASIA-KIG-HEX-2a-040622.D

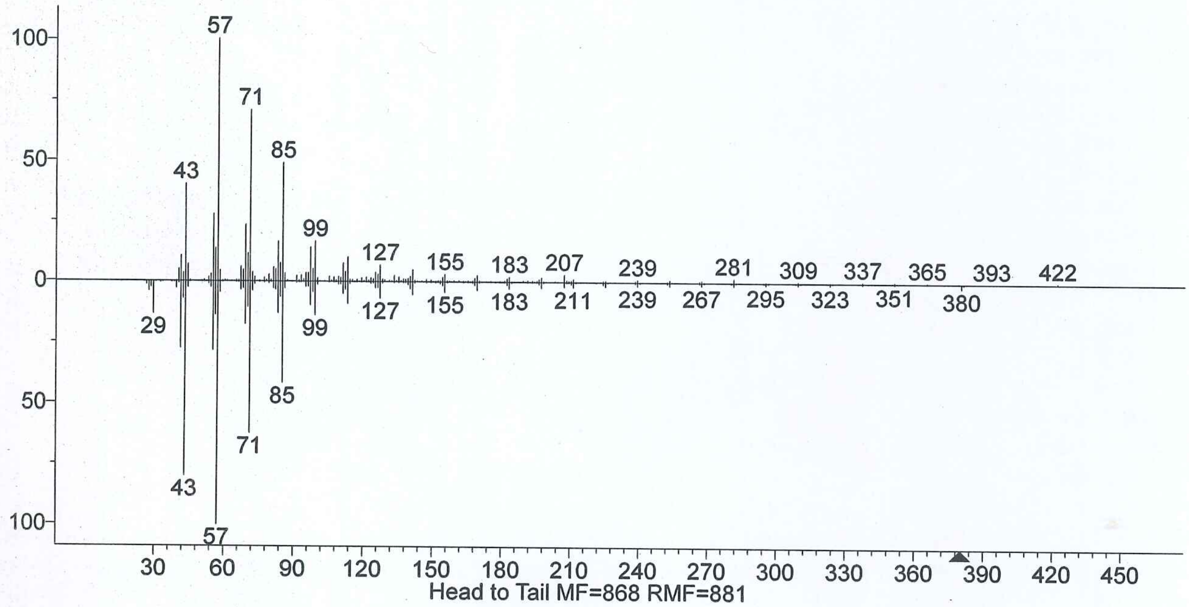

Head to Tail MF=868 RMF=881

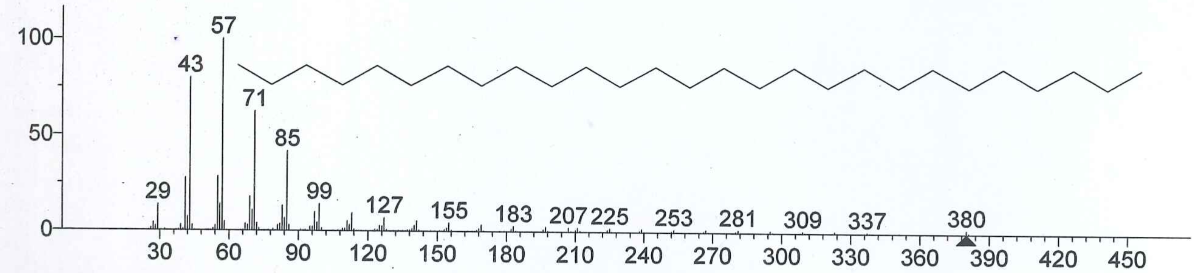

(replib) Heptacosane

Name: Heptacosane
Formula: $C_{27}H_{56}$
MW: 380 CAS#: 593-49-7 NIST#: 79427 ID#: 5508 DB: replib
Other DBs: Fine, EPA, HODOC, EINECS
Contributor: O A MAMER, MCGILL UNIVERSITY, MONTREAL, CANA
10 largest peaks:
57 999 | 43 798 | 71 622 | 85 416 | 55 283 | 41 275 | 69 176 | 99 140 | 56 139 | 29 135 |
Synonyms:
1.n-Heptacosane

Estimated non-polar retention index (n-alkane scale):
Value: 2705 iu
Confidence interval (Hydrocarbons): 39(50%) 167(95%) iu

Retention index.
1. Value: 2700 iu
Column Class: All column types
Data Type: Normal alkane RI value specified by scale definition
Source: von Kováts, E., 206. Gas-chromatographische Charakterisierung organischer Verbindungen. Teil 1: Retentionsindices aliphatischer Halogenide, Alkohole, Aldehyde und Ketone, Helv. Chim. Acta, 41(7), 1958, 1915 -1932.

2. Value: 447.8 iu
Column Type: Capillary
Column Class: Standard non-polar
Active Phase: DB
-1
Column Length: 30 m
Column Diameter: 0.2 mm
Phase Thickness: 0.25 um
Data Type: Lee RI
Program
Type: Ramp
Start T: 50 C
End T: 300 C
Heat Rate: 5 K/min
Start Time: 2 min
End Time: 5 min
Source:
Johnson, C.I.; Urso, A.; Geleta, L., Broad spectrum analysis of municipal and industrial effluents discharged into the Peace, Athabasca and Slave river basins: characterization of effluent samples, 1994 - Volume 1 of 2, Northern River Basins Study Project Report No. 121, Norther River Basins Study, Edmonton, Alberta, 1997, 27.

<...>

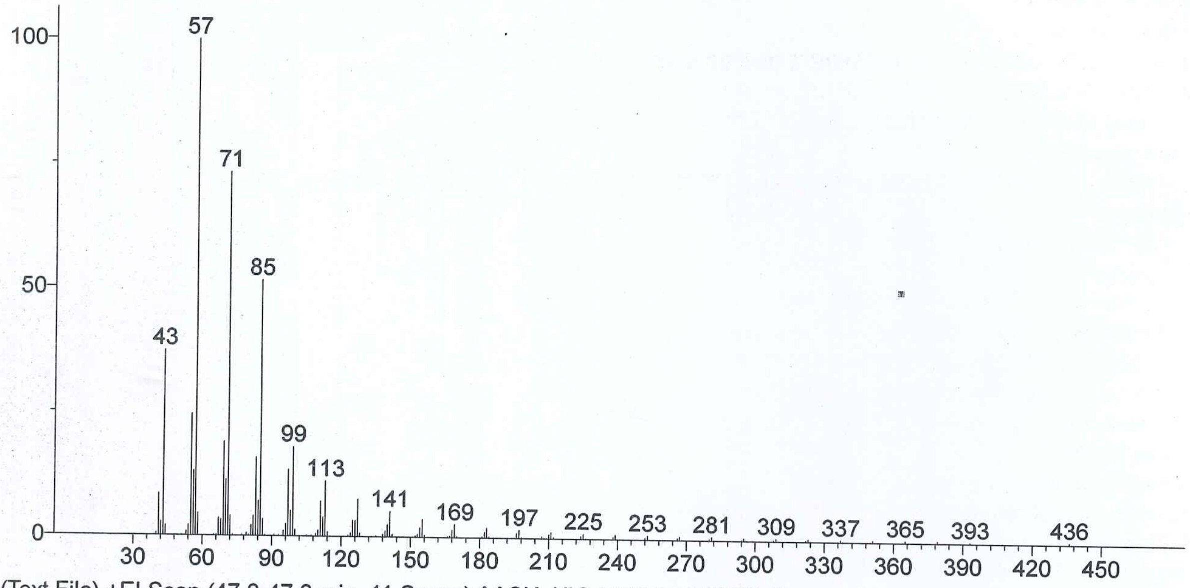

(Text File) +EI Scan (47.0-47.0 min, 11 Scans) AASIA-KIG-HEX-2a-040622.D

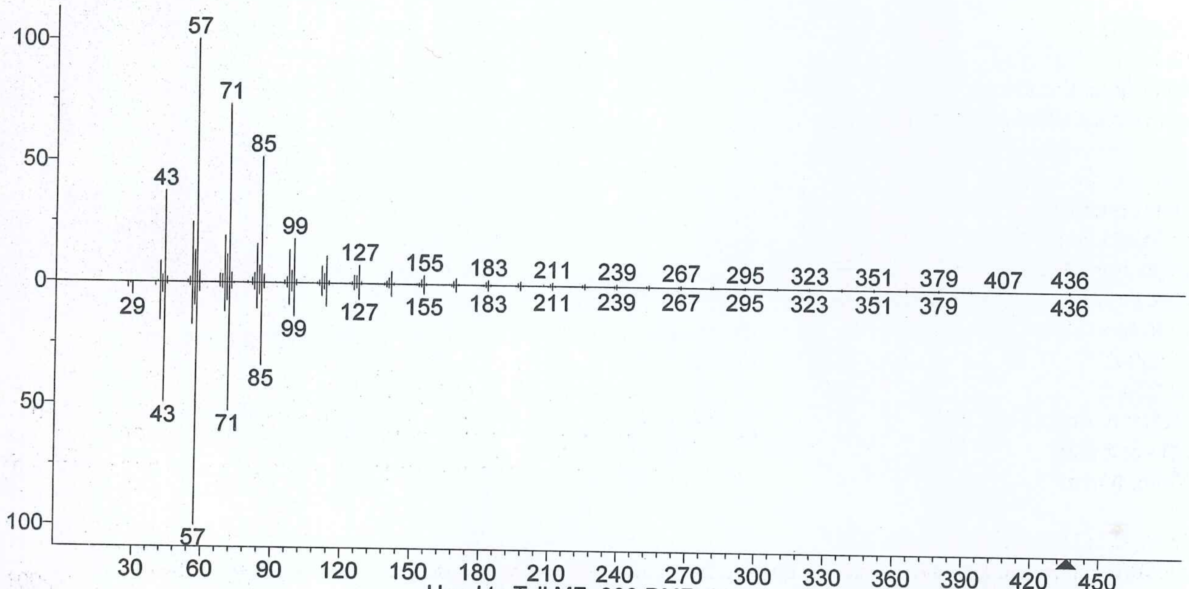

Head to Tail MF=898 RMF=899

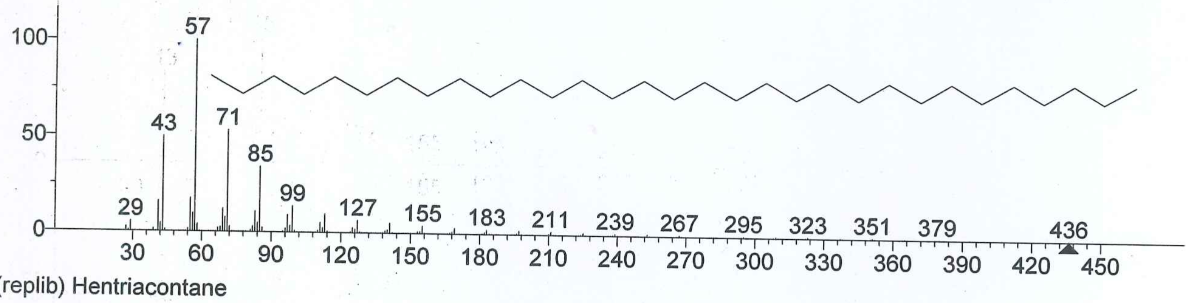

(replib) Hentriacontane

Name: Hentriacontane
Formula: $C_{31}H_{64}$
MW: 436 CAS#: 630-04-6 NIST#: 150572 ID#: 5728 DB: replib
Other DBs: None
Contributor: Chemical Concepts
10 largest peaks:
57 999 | 71 528 | 43 494 | 85 340 | 55 173 | 41 157 | 99 136 | 69 120 | 83 107 | 56 95 |
Synonyms:
1.n-Hentriacontane
2.Untriacontane

Estimated non-polar retention index (n-alkane scale):
Value: 3103 iu
Confidence interval (Hydrocarbons): 39(50%) 167(95%) iu

Retention index.
1. Value: 3100 iu
Column Class: All column types
Data Type: Normal alkane RI value specified by scale definition
Source: von Kováts, E., 206. Gas-chromatographische Charakterisierung organischer Verbindungen. Teil 1: Retentionsindices aliphatischer Halogenide, Alkohole, Aldehyde und Ketone, Helv. Chim. Acta, 41(7), 1958, 1915 -1932.

2. Value: 472.7 iu
Column Type: Capillary
Column Class: Semi-standard non-polar
Active Phase: DB
-5
Column Length: 60 m
Data Type: Lee RI
Program Type: Ramp
Source: Fuentes, M.J.; Font, R.; Gomez-Rico, M.F.; Martin-Gullon, I., Pyrolysis and combustion of waste lubricant oil from diesel cars: Decomposition and pollutants, J. Anal. Appl. Pyrolysis, 79, 2007, 215-226.

<...>

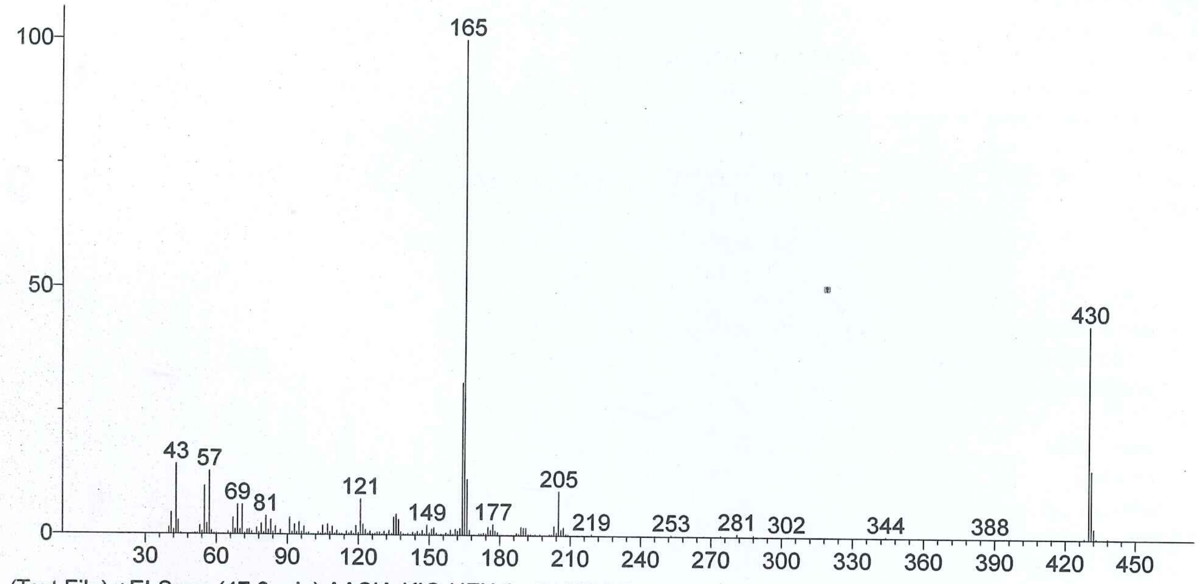

(Text File) +EI Scan (47.3 min) AASIA-KIG-HEX-2a-040622.D

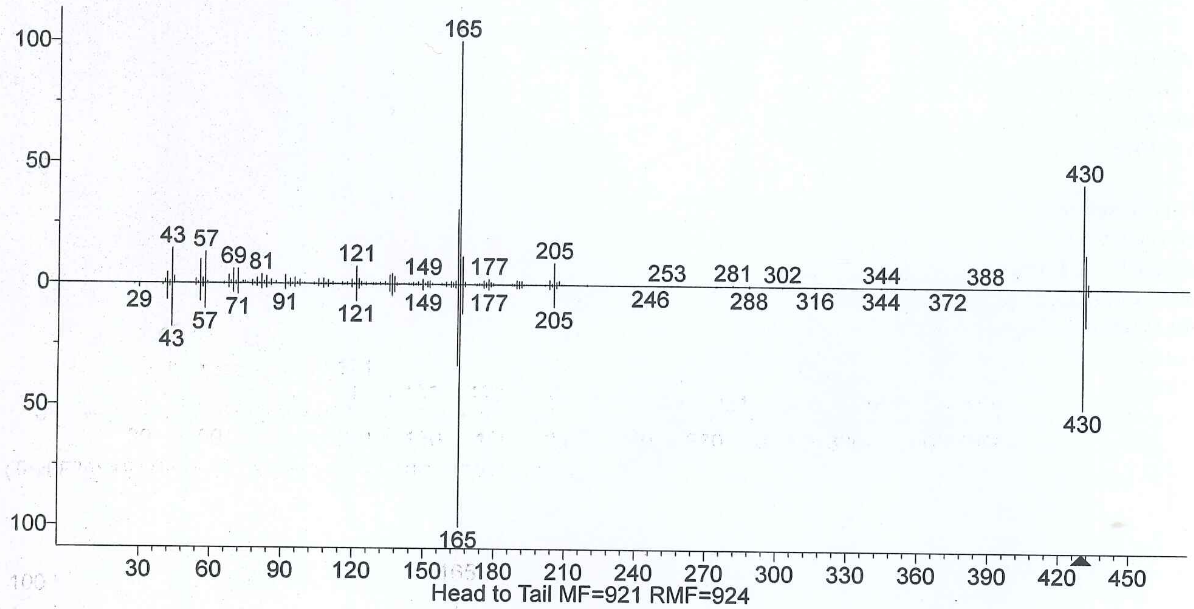

Head to Tail MF=921 RMF=924

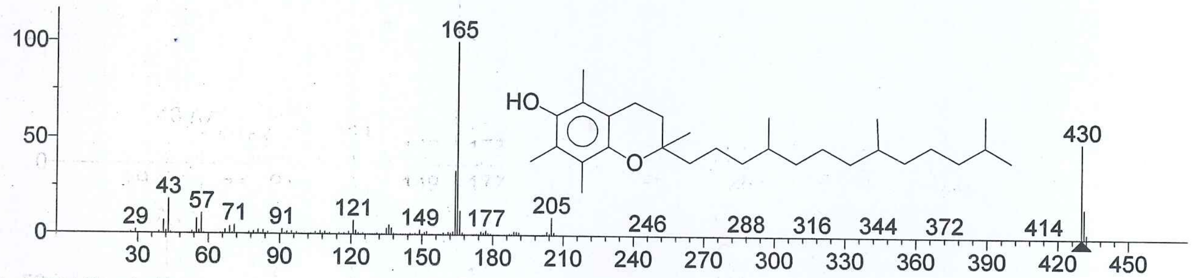

(replib) Vitamin E

Name: Vitamin E
Formula: $C_{29}H_{50}O_2$
MW: 430 CAS#: 59-02-9 NIST#: 290780 ID#: 21723 DB: replib
Other DBs: TSCA, RTECS, HODOC, NIH, EINECS
Contributor: NIST Mass Spectrometry Data Center, 1998.
10 largest peaks:
165 999 | 430 494 | 164 332 | 43 178 | 431 155 | 166 122 | 57 104 | 205 91 | 55 75 | 121 71 |
Synonyms:
1.2H-1-Benzopyran-6-ol, 3,4-dihydro-2,5,7,8-tetramethyl-2-(4,8,12-trimethyltridecyl)-, [2R-[2R*(4R*,8R*)]]-
2.α-Tocopherol
3.α-Tokoferol
4.(All-R)-α-Tocopherol
5.(2R,4'R,8'R)-α-Tocopherol
6.D-α-tocopherol
7.Almefrol
8.Antisterility vitamin
9.Denamone
10.Emipherol
11.Endo E
12.Ephynal
13.Eprolin
14.Eprolin S
15.Epsilan
16.Esorb
17.Etamican
18.Etavit
19.Evion
20.Evitaminum
21.Ilitia
22.Phytogermine
23.Profecundin
24.Spavit E
25.Syntopherol
26.Tokopharm
27.Vascuals
28.Verrol
29.Vi-E
30.Vitaplex E
31.Vitayonon
32.Viteolin
33.5,7,8-Trimethyltocol
34.component of E and C-Level
35.component of Estopherol
36.Aquasol E
37.Lan-E
38.Med-E
39.Vita E
40.Covi-ox
41.Spavit
42.(R,R,R)-α-Tocopherol
43.(+)-α-Tocopherol
44.(+)-α-Tocopherolα-tocopherolantisterility vitamin
45.[2R-2R*(4R*,8R*)]-3,4-Dihydro-2,5,7,8-tetramethyl-2-(4,8,12-trimethyltridecyl)-2H-1-benzopyran-6-ol
46.2,5,7,8-Tetramethyl-2-(4',8',12'-trimethyltridecyl)-6-chromanol

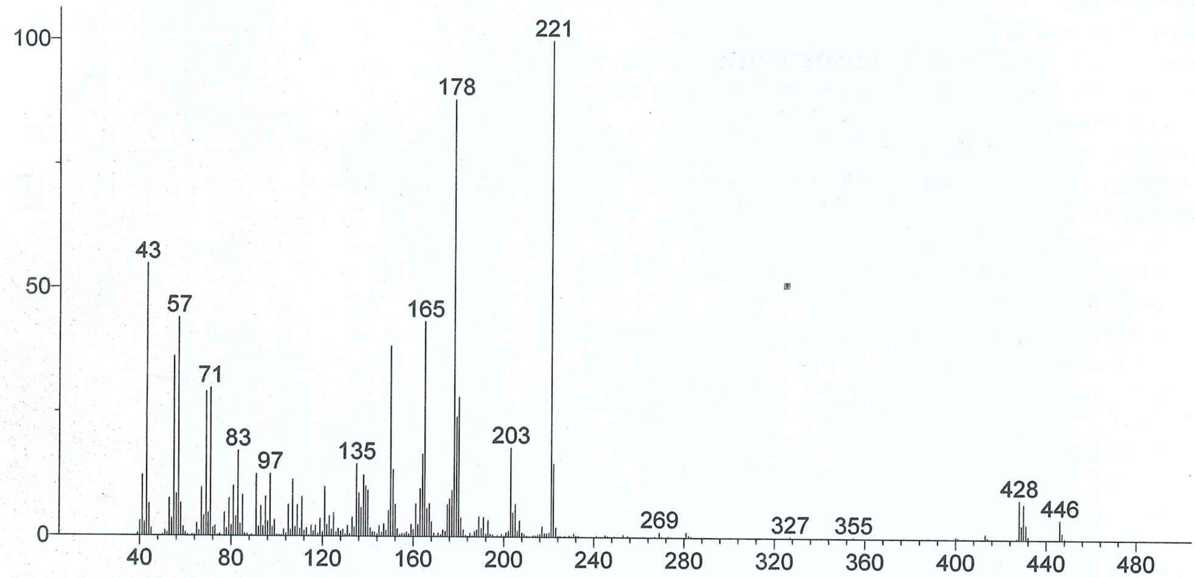

(Text File) +EI Scan (47.4 min) AASIA-KIG-HEX-2a-040622.D

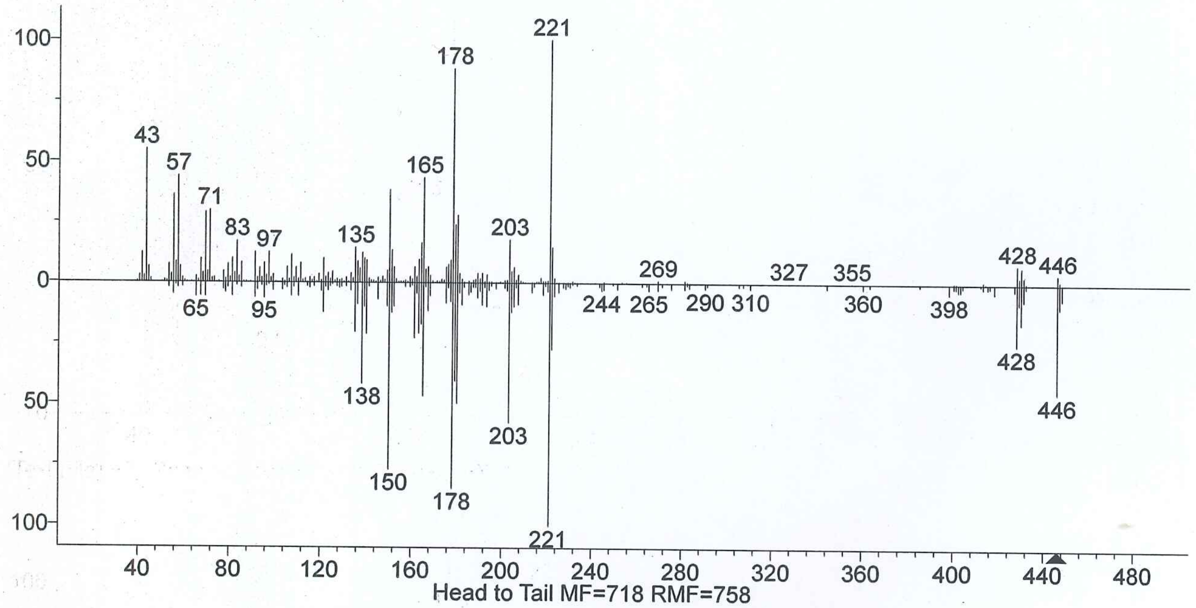

Head to Tail MF=718 RMF=758

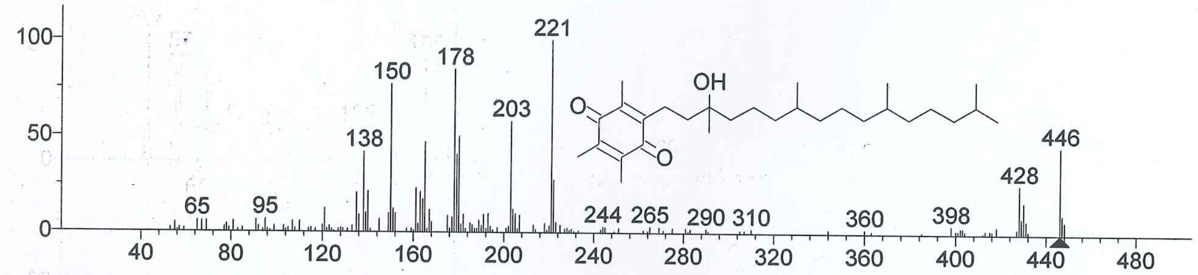

(mainlib) α-Tocopherolquinone

Name: α-Tocopherolquinone
Formula: $C_{29}H_{50}O_3$
MW: 446 NIST#: 314346 ID#: 155063 DB: mainlib
Contributor: H.-U. Melchert ET AL, J.Chrom. A, 976,215(2002)
10 largest peaks:
221 999 | 178 846 | 150 768 | 203 576 | 180 498 | 165 467 | 446 451 | 138 417 | 179 406 | 222 275 |
Synonyms:
no synonyms.

Estimated non-polar retention index (n-alkane scale):
Value: 3172 iu
Confidence interval (Diverse functional groups): 89(50%) 382(95%) iu

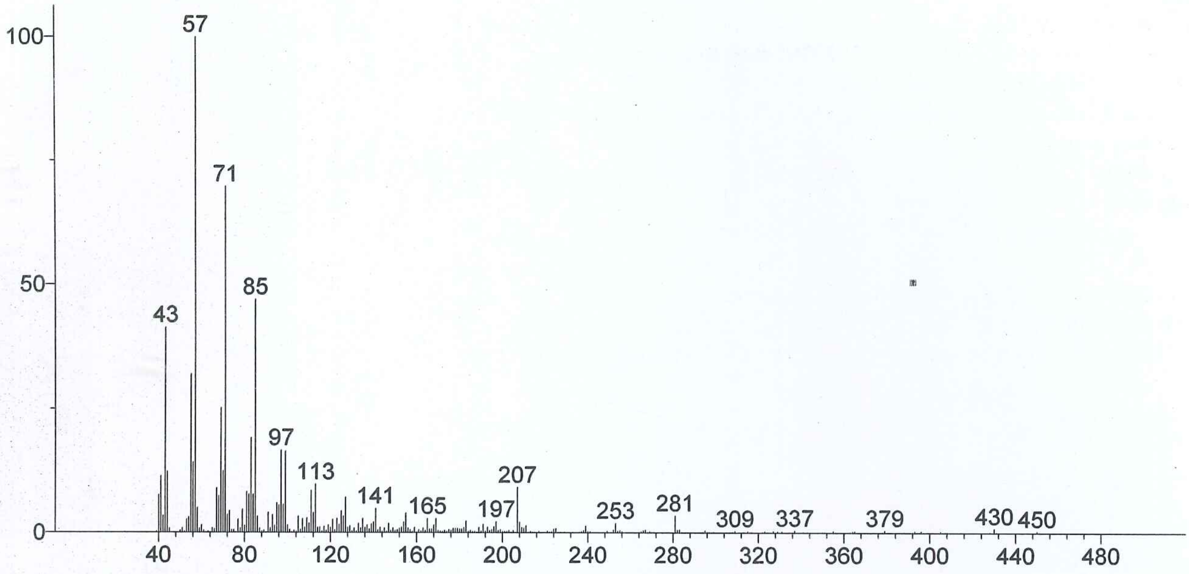

(Text File) +EI Scan (47.8-47.8 min, 11 Scans) AASIA-KIG-HEX-2a-040622.D

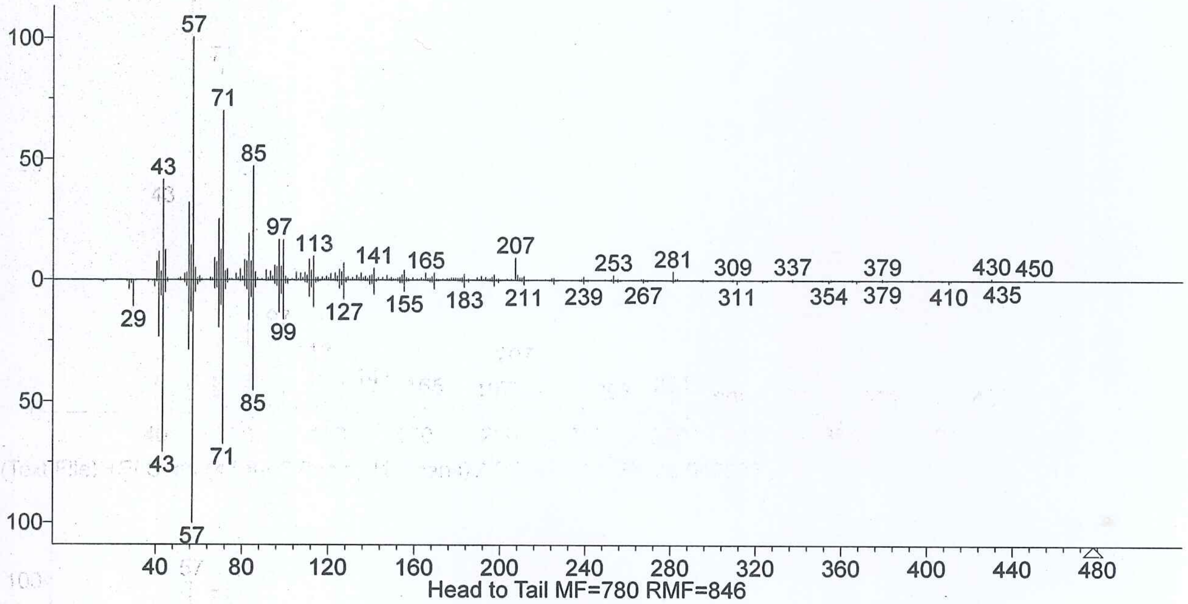

Head to Tail MF=780 RMF=846

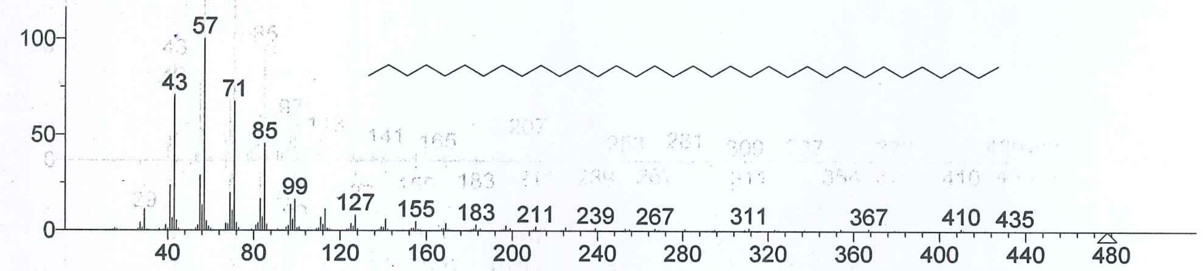

(replib) Tetratriacontane

Name: Tetratriacontane
Formula: $C_{34}H_{70}$
MW: 478 CAS#: 14167-59-0 NIST#: 17995 ID#: 5491 DB: replib
Other DBs: Fine, HODOC, NIH, EINECS
Contributor: D. LUKE UNILEVER RESEARCH LAB., PORT SUNLIGHT
10 largest peaks:
57 999 | 43 705 | 71 673 | 85 453 | 55 286 | 41 233 | 69 196 | 83 164 | 99 162 | 97 135 |
Synonyms:
1.n-Tetratriacontane

Estimated non-polar retention index (n-alkane scale):
Value: 3401 iu
Confidence interval (Hydrocarbons): 39(50%) 167(95%) iu

Retention index.
1. Value: 3400 iu
Column Class: All column types
Data Type: Normal alkane RI value specified by scale definition
Source: von Kováts, E., 206. Gas-chromatographische Charakterisierung organischer Verbindungen. Teil 1: Retentionsindices aliphatischer Halogenide, Alkohole, Aldehyde und Ketone, Helv. Chim. Acta, 41(7), 1958, 1915 -1932.

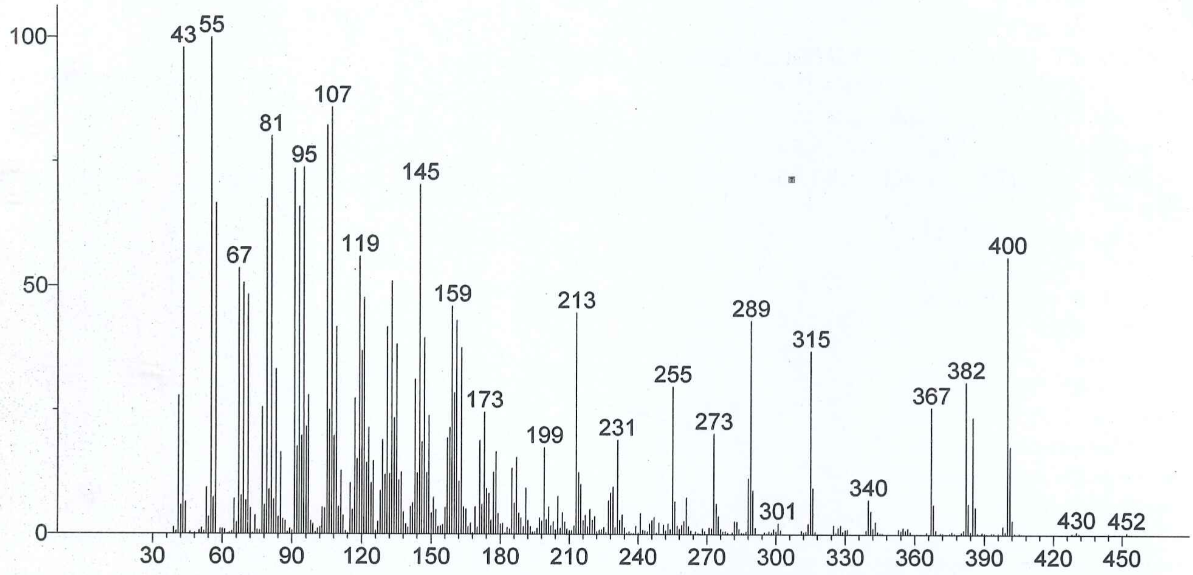

(Text File) +EI Scan (48.1-48.1 min, 10 Scans) AASIA-KIG-HEX-2a-040622.D Subtract

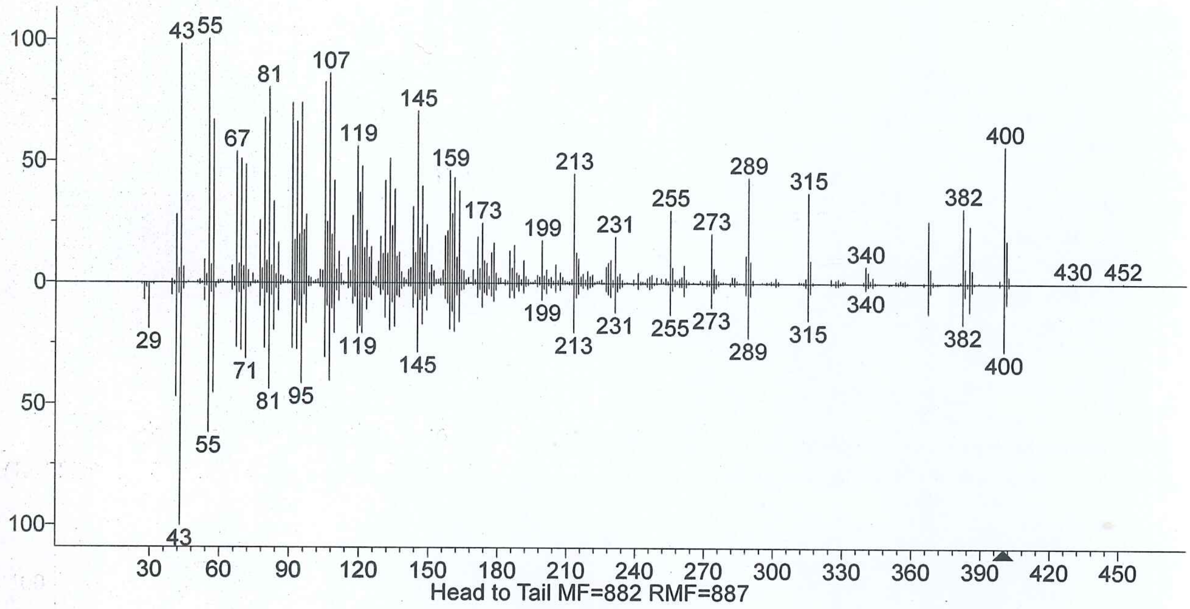

Head to Tail MF=882 RMF=887

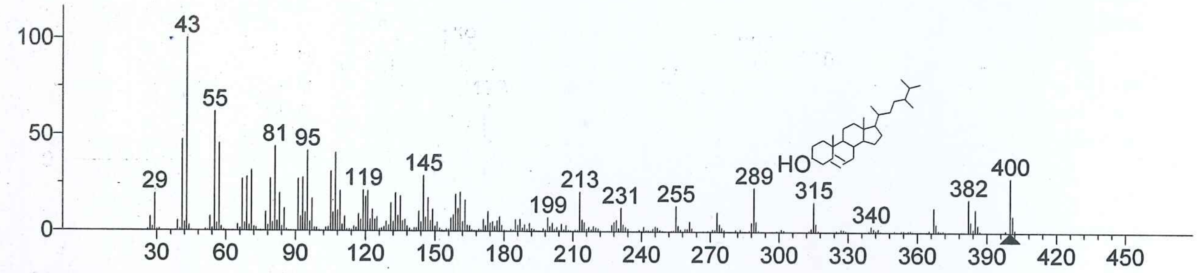

(mainlib) Campesterol

Name: Campesterol
Formula: $C_{28}H_{48}O$
MW: 400 CAS#: 474-62-4 NIST#: 151556 ID#: 6613 DB: mainlib
Other DBs: Fine, HODOC, NIH, EINECS
Contributor: Chemical Concepts
10 largest peaks:
43 999 | 55 617 | 41 470 | 57 452 | 81 436 | 95 414 | 107 403 | 71 312 | 105 306 | 145 285 |
Synonyms:
1.Ergost-5-en-3-ol, (3β,24R)-
2.Ergost-5-en-3β-ol, (24R)-
3.(24R)-5-Ergosten-3β-ol
4.Campesterin
5.Campestrol
6.24α-Methylcholesterol
7.24α-Methyl-5-cholesten-3β-ol
8.(24R)-Methylcholest-5-en-3β-ol
9..DELTA.5-24-Isoergosten-3β-ol
10.Ergost-5-en-3-ol #

Estimated non-polar retention index (n-alkane scale):
Value: 2632 iu
Confidence interval (Low reliability): 174(50%) 752(95%) iu

Retention index.
1. Value: 3193.06 iu
Column Type: Capillary
Column Class: Standard non-polar
Active Phase: DB-1
Column
Length: 30 m
Carrier Gas: H2
Column Diameter: 0.25 mm
Phase Thickness: 0.25 um
Data Type: Kovats
RI
Program Type: Isothermal
Start T: 270 C
Source: Stránský, K.; Valterová, I.; Fiedler, P., Nonsaponifiable lipid
components of the pollen of elder (Sambucus nigra L.), J. Chromatogr. A, 936, 2001, 173-181.
2. Value: 3193.53
iu
Column Type: Capillary
Column Class: Standard non-polar
Active Phase: DB-1
Column Length: 30
m
Carrier Gas: H2
Column Diameter: 0.25 mm
Phase Thickness: 0.25 um
Data Type: Kovats RI
Program
Type: Isothermal
Start T: 270 C

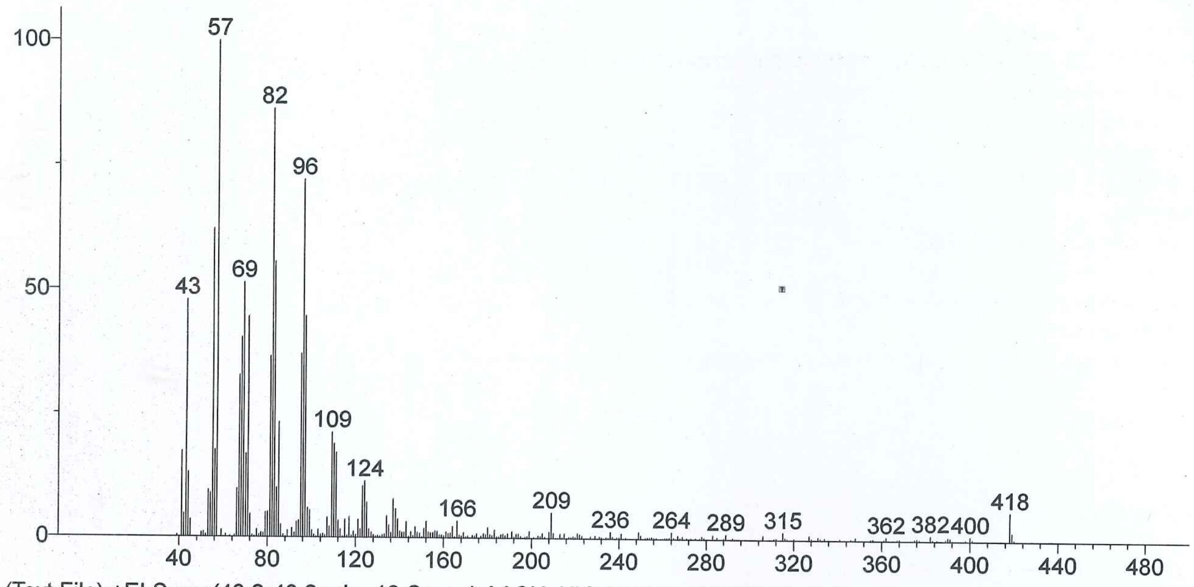

(Text File) +EI Scan (48.2-48.2 min, 12 Scans) AASIA-KIG-HEX-2a-040622.D Subtract

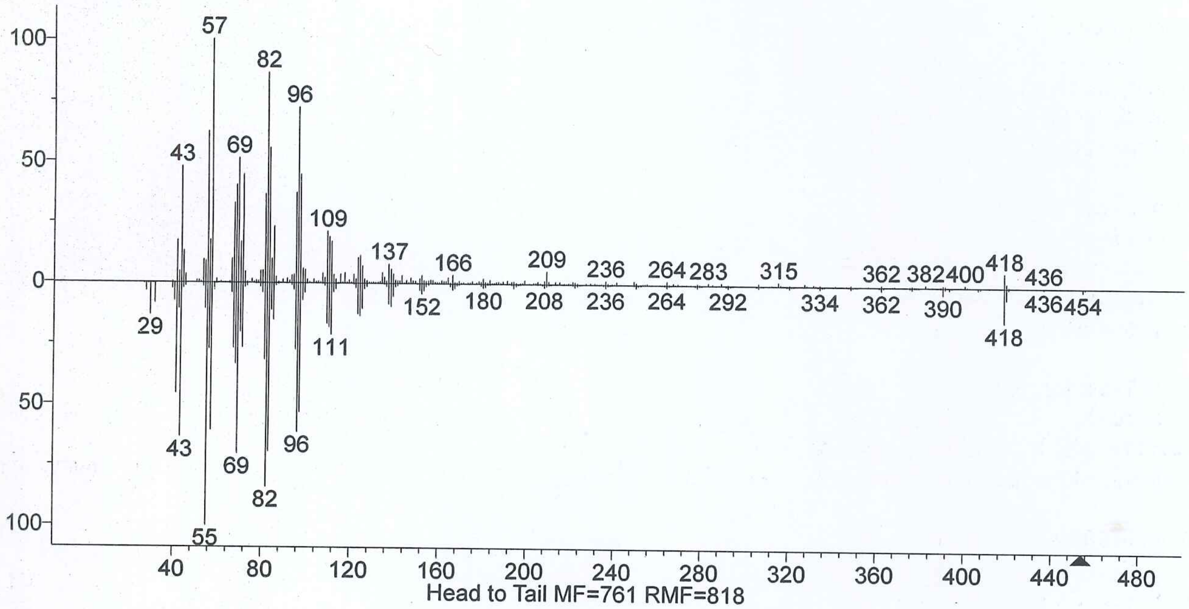

Head to Tail MF=761 RMF=818

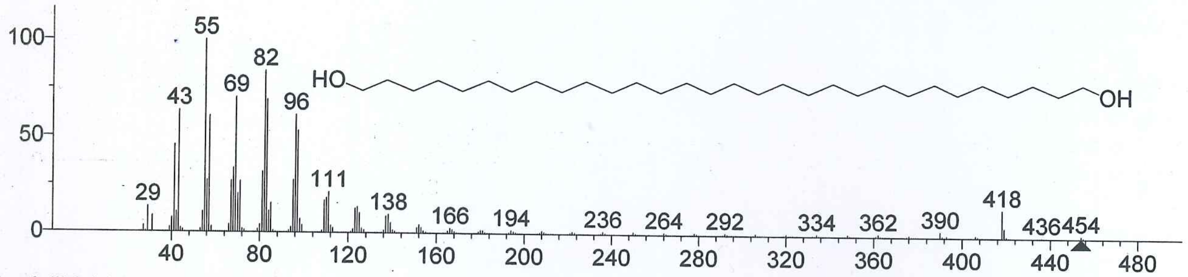

(mainlib) 1,30-Triacontanediol

Name: 1,30-Triacontanediol
Formula: $C_{30}H_{62}O_2$
MW: 454 CAS#: 36645-68-8 NIST#: 159375 ID#: 18655 DB: mainlib
Other DBs: None
Contributor: Chemical Concepts
10 largest peaks:
55 999 | 82 838 | 69 701 | 83 691 | 43 633 | 96 611 | 57 605 | 97 530 | 41 454 | 68 334 |
Synonyms:
no synonyms.

Estimated non-polar retention index (n-alkane scale):
Value: 3489 iu
Confidence interval (Alcohols): 41(50%) 176(95%) iu

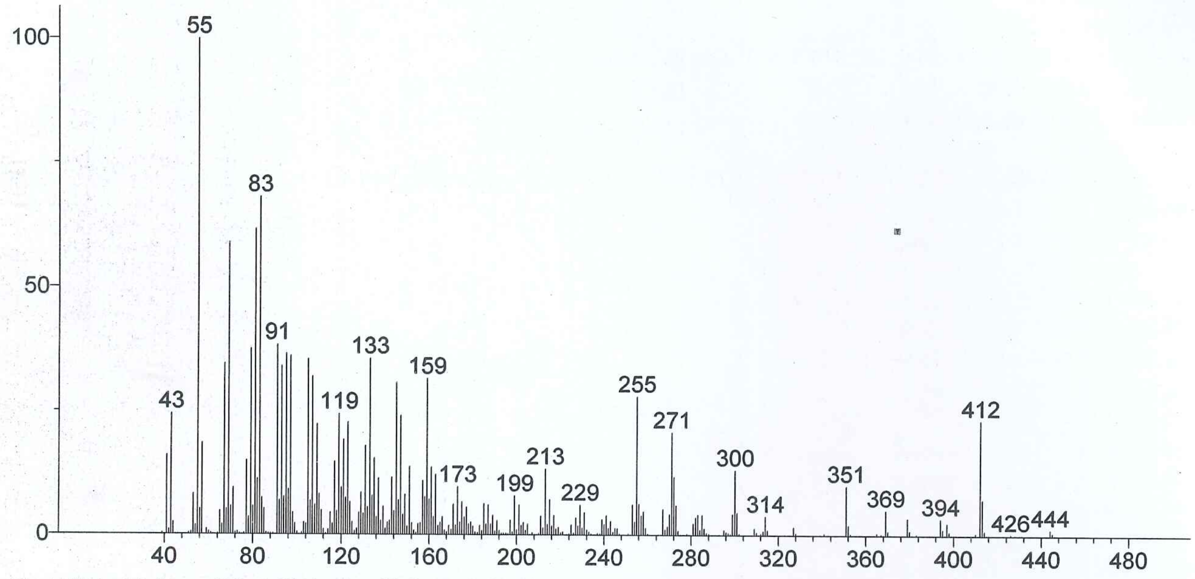

(Text File) +EI Scan (48.4-48.4 min, 11 Scans) AASIA-KIG-HEX-2a-040622.D Subtract

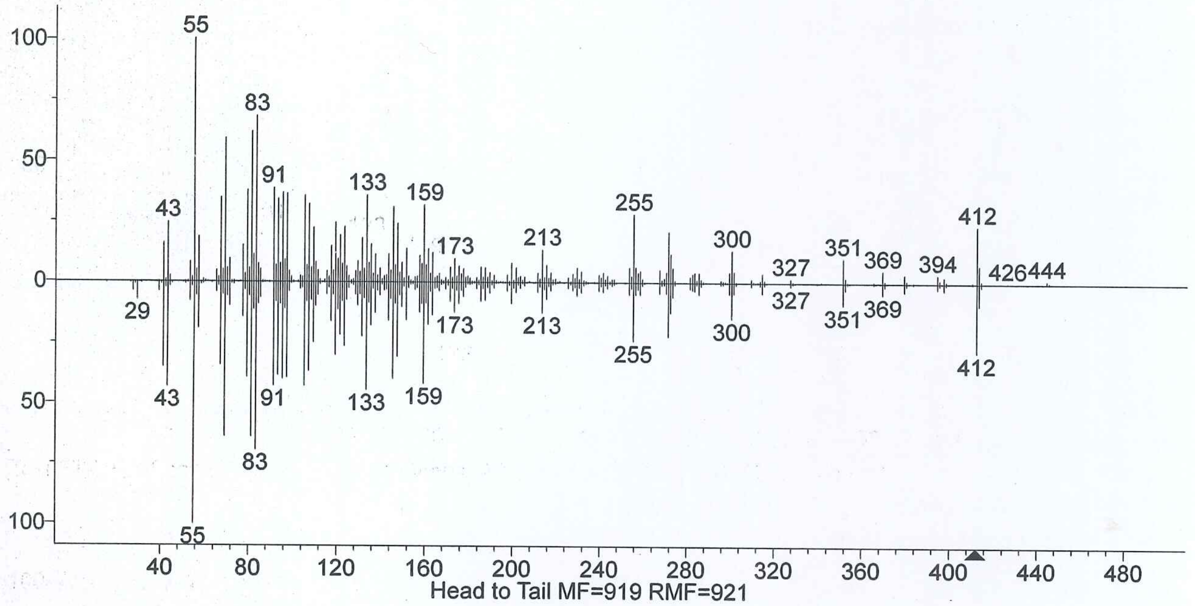

Head to Tail MF=919 RMF=921

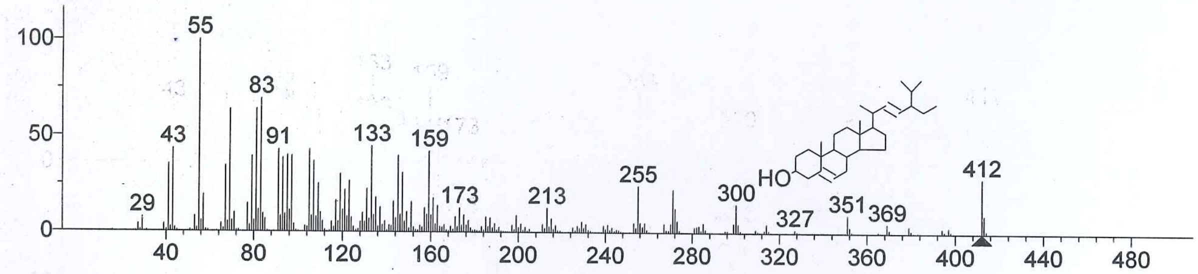

(mainlib) Stigmasterol

Name: Stigmasterol
Formula: $C_{29}H_{48}O$
MW: 412 CAS#: 83-48-7 NIST#: 352610 ID#: 18876 DB: mainlib
Other DBs: Fine, HODOC, NIH, EINECS
Contributor: NIST Mass Spectrometry Data Center
10 largest peaks:
55 999 | 83 692 | 81 638 | 69 636 | 133 444 | 43 431 | 91 427 | 105 427 | 159 418 | 95 398 |
Synonyms:
1.Stigmasta-5,22-dien-3-ol, (3β,22E)-
2.Stigmasta-5,22-dien-3β-ol
3.β-Stigmasterol
4.(24S)-5,22-Stigmastadien-3β-ol
5.Stigmasta-5,22-dien-3-ol, (3β)-
6.Stigmasterin
7.Phytosterol
8.5,22-Cholestadien-24-ethyl-3β-ol
9..DELTA.5,22-Stigmastadien-3β-ol
10.I-Stigmasterol
11.Stigmasta-5,22-dien-3-ol
12.(22E)-Stigmasta-5,22-dien-3-ol #
Estimated non-polar retention index (n-alkane scale):
Value: 2739 iu
Confidence interval (Low reliability): 174(50%) 752(95%) iu

Retention index.
1. Value: 3221.93 iu
Column Type: Capillary
Column Class: Standard non-polar
Active Phase: DB-1
Column
Length: 30 m
Carrier Gas: H2
Column Diameter: 0.25 mm
Phase Thickness: 0.25 um
Data Type: Kovats
RI
Program Type: Isothermal
Start T: 270 C
Source: Stránský, K.; Valterová, I.; Fiedler, P., Nonsaponifiable lipid components of the pollen of elder (Sambucus nigra L.), J. Chromatogr. A, 936, 2001, 173-181.

2. Value: 3222.15
iu
Column Type: Capillary
Column Class: Standard non-polar
Active Phase: DB-1
Column Length: 30
m
Carrier Gas: H2
Column Diameter: 0.25 mm
Phase Thickness: 0.25 um
Data Type: Kovats RI
Program

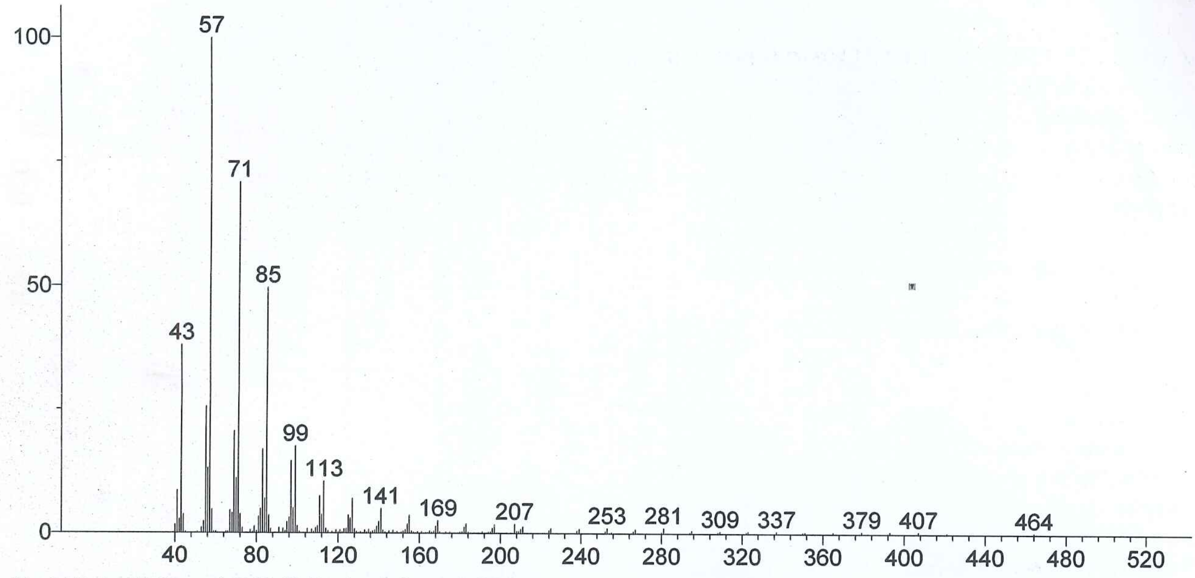

(Text File) +EI Scan (48.7-48.7 min, 9 Scans) AASIA-KIG-HEX-2a-040622.D

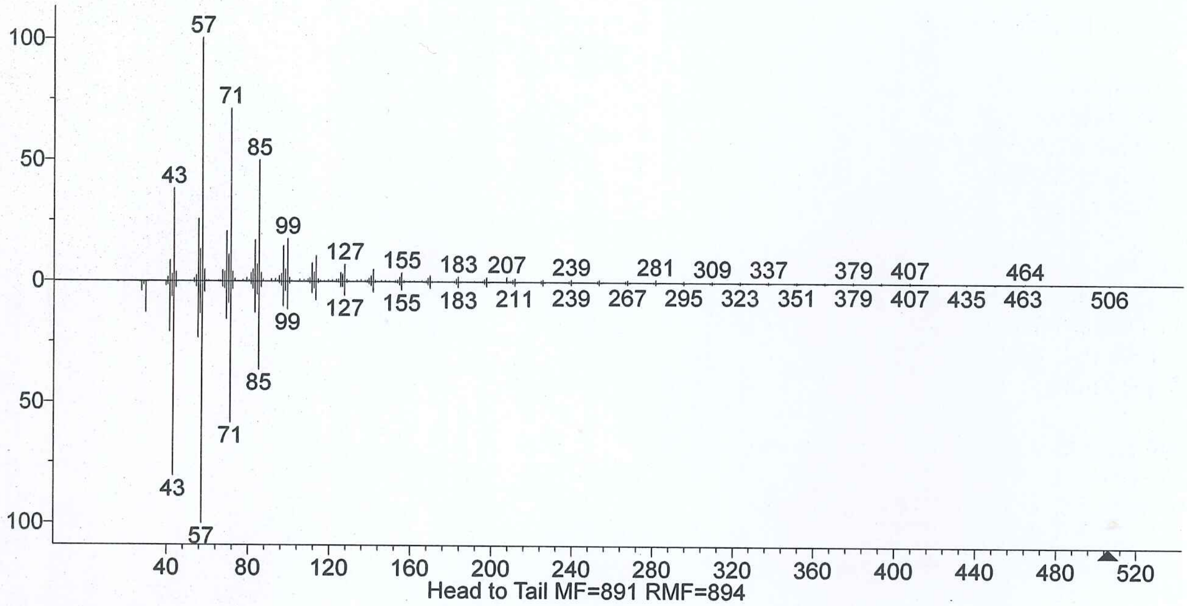

Head to Tail MF=891 RMF=894

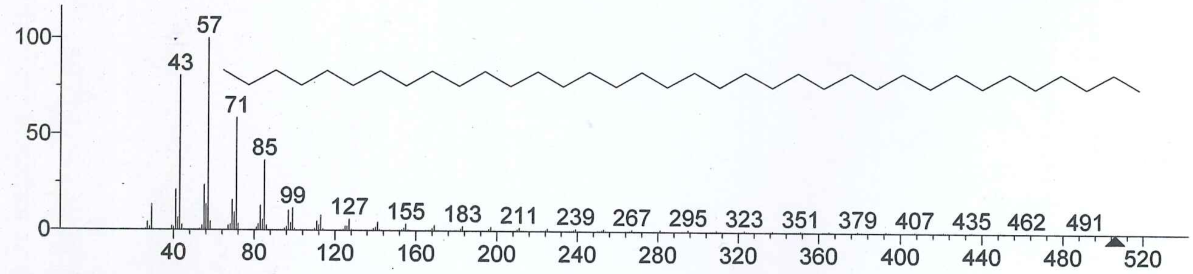

(replib) Hexatriacontane

Name: Hexatriacontane
Formula: $C_{36}H_{74}$
MW: 506 CAS#: 630-06-8 NIST#: 18164 ID#: 5498 DB: replib
Other DBs: Fine, TSCA, HODOC, NIH, EINECS
10 largest peaks:
57 999 | 43 803 | 71 583 | 85 363 | 55 234 | 41 208 | 69 156 | 56 133 | 29 130 | 83 128 |
Synonyms:
1.n-Hexatriacontane

Estimated non-polar retention index (n-alkane scale):
Value: 3600 iu
Confidence interval (Hydrocarbons): 39(50%) 167(95%) iu

Retention index.
1. Value: 3600 iu
Column Class: All column types
Data Type: Normal alkane RI value specified by scale definition
Source: von Kováts, E., 206. Gas-chromatographische Charakterisierung organischer Verbindungen. Teil 1: Retentionsindices aliphatischer Halogenide, Alkohole, Aldehyde und Ketone, Helv. Chim. Acta, 41(7), 1958, 1915 -1932.

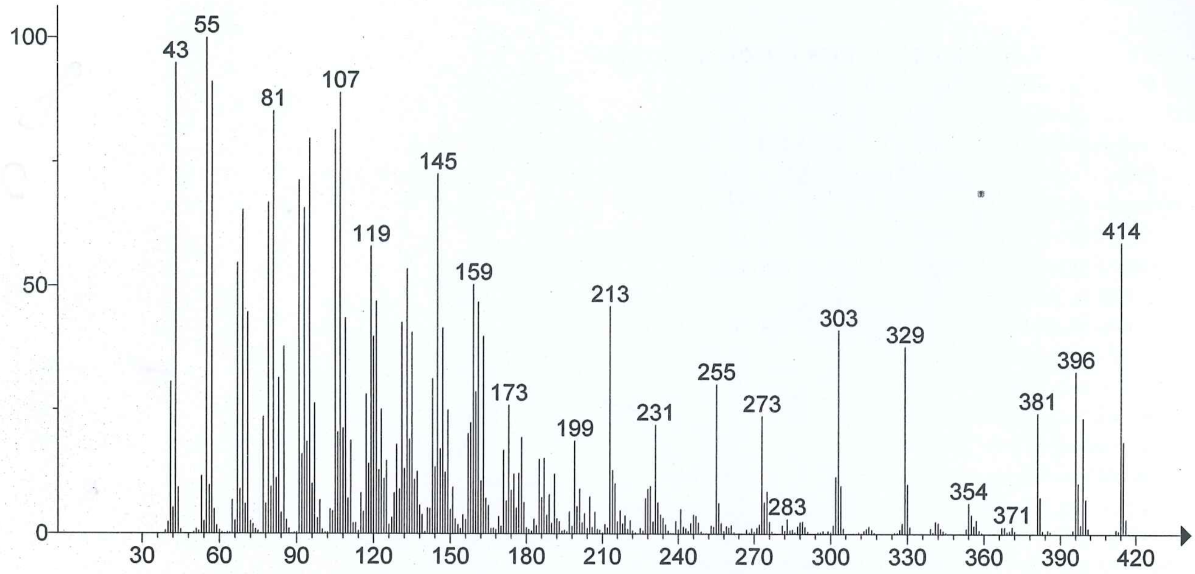

(Text File) +EI Scan (49.0-49.0 min, 8 Scans) AASIA-KIG-HEX-2a-040622.D

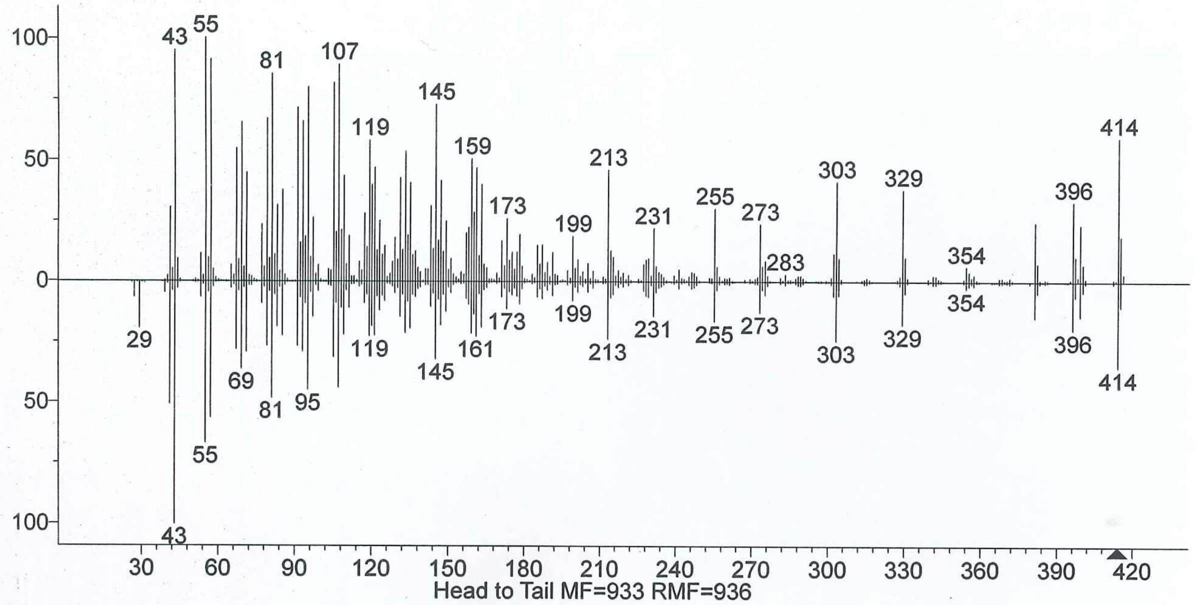

Head to Tail MF=933 RMF=936

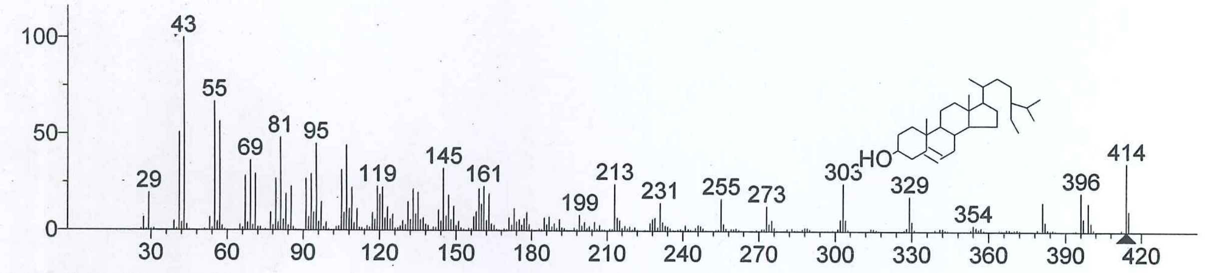

(mainlib) γ-Sitosterol

Name: γ-Sitosterol
Formula: $C_{29}H_{50}O$
MW: 414 CAS#: 83-47-6 NIST#: 151558 ID#: 6743 DB: mainlib
Other DBs: HODOC, EINECS
Contributor: Chemical Concepts
10 largest peaks:
43 999 | 55 667 | 57 563 | 41 507 | 81 481 | 95 449 | 107 440 | 69 362 | 414 354 | 145 321 |
Synonyms:
1.Stigmast-5-en-3-ol, (3β,24S)-
2.Stigmast-5-en-3β-ol, (24S)-
3.Clionasterol
4.Fucosterol, β-dihydro-
5.24β-Ethyl-5-cholesten-3β-ol
6.β-Dihydrofucosterol
7.22,23-Dihydroporiferasterol
8.24S-Ethylcholest-5-en-3β-ol
9.24β-Ethylcholesterol
10.Stigmast-5-en-3-ol #

Estimated non-polar retention index (n-alkane scale):
Value: 2731 iu
Confidence interval (Low reliability): 174(50%) 752(95%) iu

Retention index.
1. Value: 3066 iu
Column Type: Capillary
Column Class: Semi-standard non-polar
Active Phase: HP-5
Column
Length: 30 m
Carrier Gas: He
Column Diameter: 0.25 mm
Phase Thickness: 0.25 um
Data Type: Normal
alkane RI
Program Type: Complex
Description: 60C(5min) =>3C/min =>120C (2min) =>2C/min =>200C (2min)
=>3C/min =>320C
Source: Yasar, A.; Üçüncü, O.; Güleç, C.; Inceer, H.; Ayaz, S.; Yayh, N., GC-MS analysis of chloroform extracts in flowers, stems, and roots of Tripleurospermum callosum, Pharm. Biol., 43(2), 2005, 108-112.

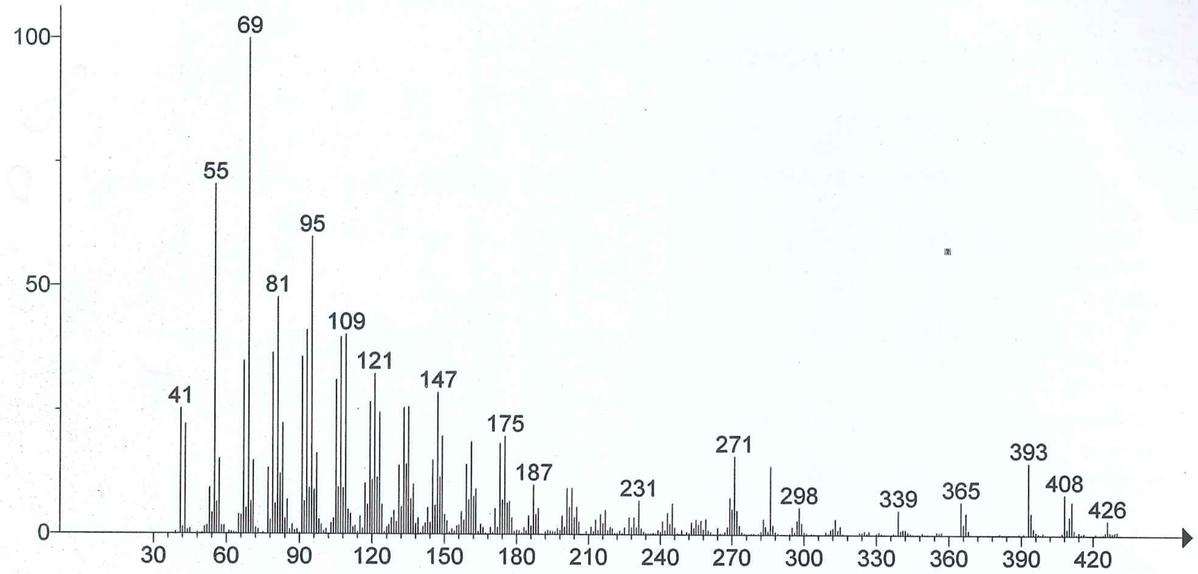

(Text File) +EI Scan (49.7-49.7 min, 8 Scans) AASIA-KIG-HEX-2a-040622.D Subtract

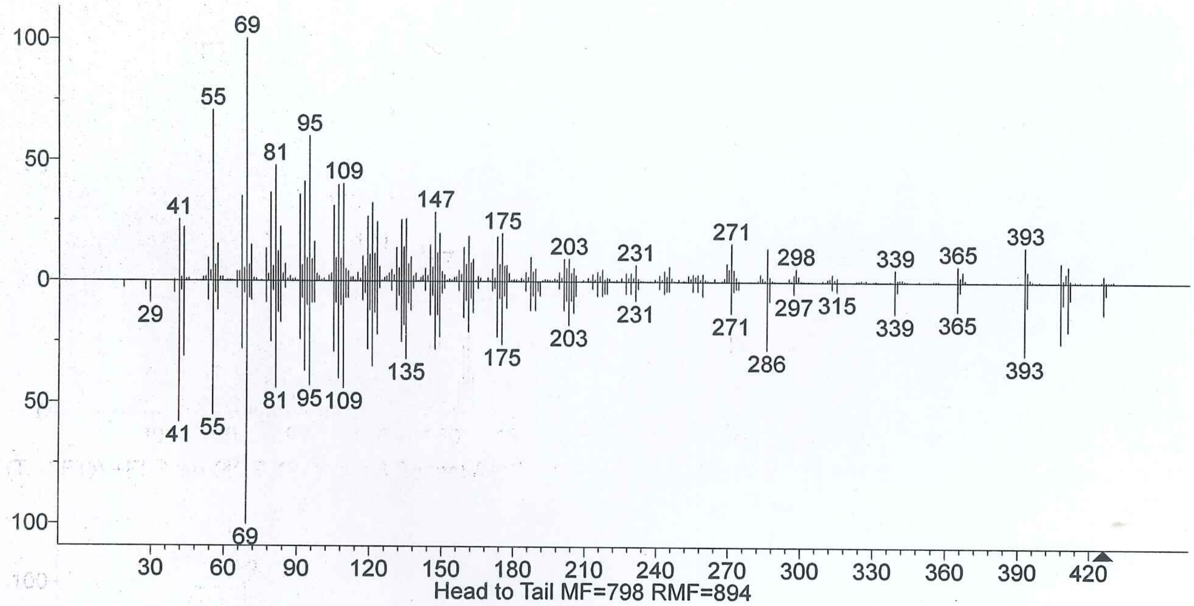

Head to Tail MF=798 RMF=894

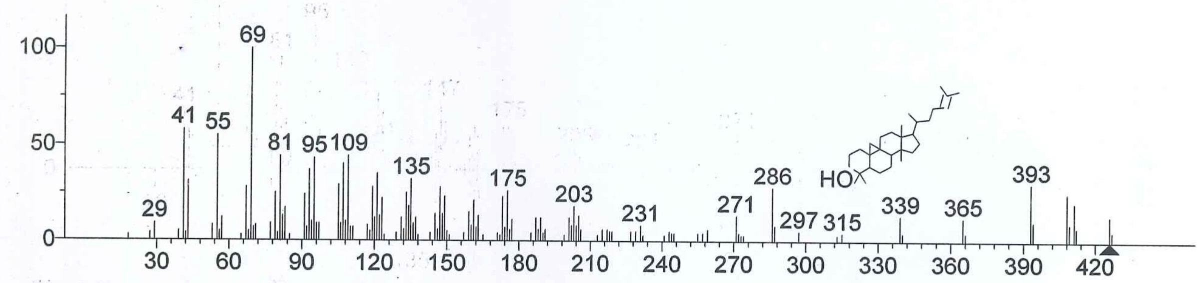

(mainlib) 9,19-Cyclolanost-24-en-3-ol, (3β)-

Name: 9,19-Cyclolanost-24-en-3-ol, (3β)-
Formula: $C_{30}H_{50}O$
MW: 426 CAS#: 469-38-5 NIST#: 36782 ID#: 29955 DB: mainlib
Other DBs: None
Contributor: DR GLENN PATTERSON
10 largest peaks:
69 999 | 41 579 | 55 549 | 81 440 | 109 440 | 95 430 | 107 400 | 93 370 | 121 350 | 135 320 |
Synonyms:
1.9,19-Cyclo-9β-lanost-24-en-3β-ol
2.Cycloartenol
3.Handianol
4.1-(1,5-Dimethyl-4-hexenyl)-3a,6,6,12a-tetramethyltetradecahydro-1H-cyclopenta[a]cyclopropa[e]phenanthren-7-ol
#

Estimated non-polar retention index (n-alkane scale):
Value: 2816 iu
Confidence interval (Low reliability): 174(50%) 752(95%) iu

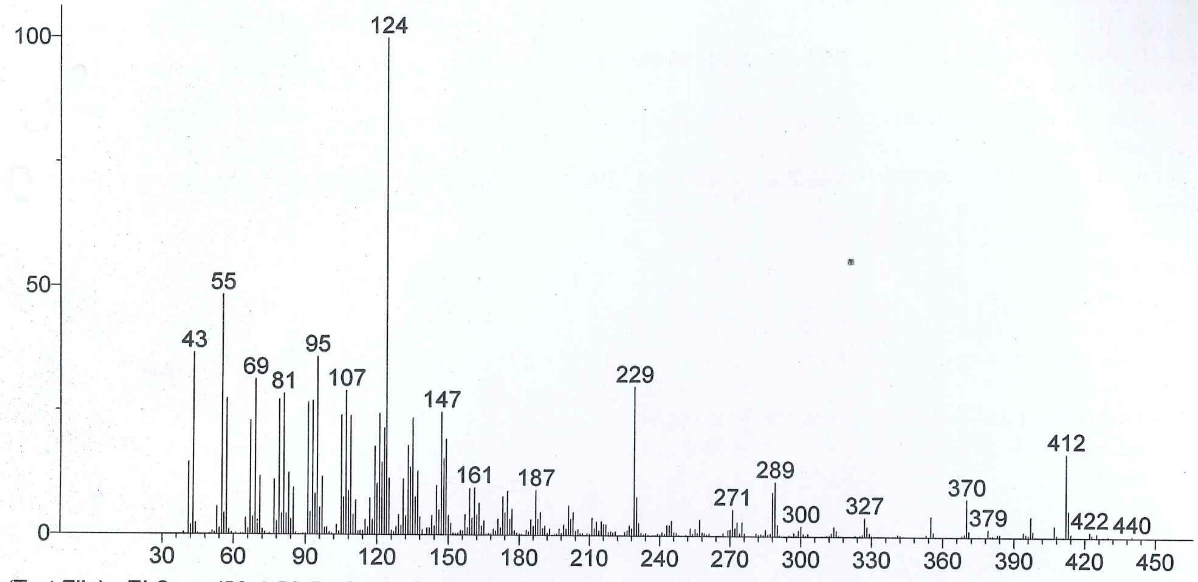

(Text File) +EI Scan (50.4-50.5 min, 16 Scans) AASIA-KIG-HEX-2a-040622.D Subtract

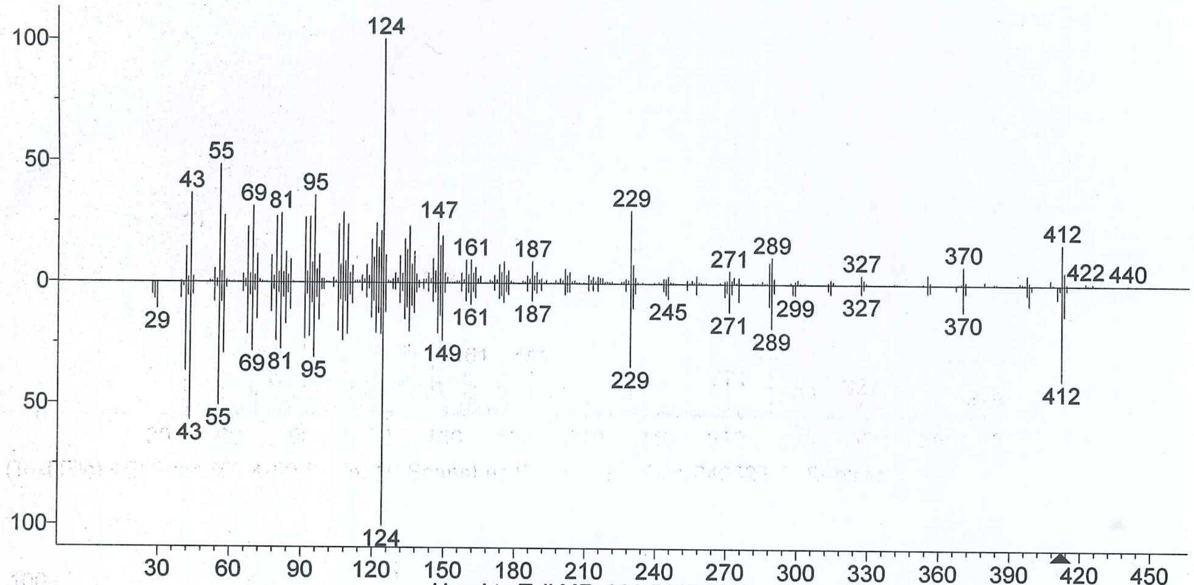

Head to Tail MF=829 RMF=895

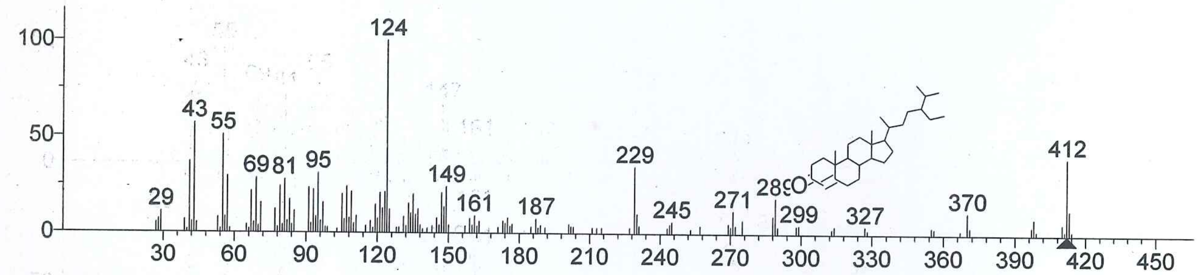

(mainlib) Stigmast-4-en-3-one

Name: Stigmast-4-en-3-one
Formula: $C_{29}H_{48}O$
MW: 412 CAS#: 1058-61-3 NIST#: 17165 ID#: 87236 DB: mainlib
Other DBs: NIH
Contributor: S.HAYASHI, DEPT. OF CHEM., HIROSHIMA UNIV., HIROSHIMA, JAPAN.
10 largest peaks:
124 999 | 43 567 | 55 506 | 412 394 | 41 366 | 229 344 | 95 308 | 57 292 | 69 283 | 81 275 |
Synonyms:
1.4-Stigmasten-3-one
2.Sitostenone
3..DELTA.4-Sitosterol-3-one

Estimated non-polar retention index (n-alkane scale):
Value: 2714 iu
Confidence interval (Low reliability): 174(50%) 752(95%) iu

Retention index.
1. Value: 3108 iu
Column Type: Capillary
Column Class: Semi-standard non-polar
Active Phase: HP-5
Column
Length: 30 m
Carrier Gas: He
Column Diameter: 0.25 mm
Phase Thickness: 0.25 um
Data Type: Normal
alkane RI
Program Type: Complex
Description: 60C(5min) =>3C/min =>120C (2min) =>2C/min =>200C (2min)
=>3C/min =>320C
Source: Yasar, A.; Üçüncü, O.; Güleç, C.; Inceer, H.; Ayaz, S.; Yayh, N., GC-MS analysis of chloroform extracts in flowers, stems, and roots of Tripleurospermum callosum, Pharm. Biol., 43(2), 2005, 108-112.

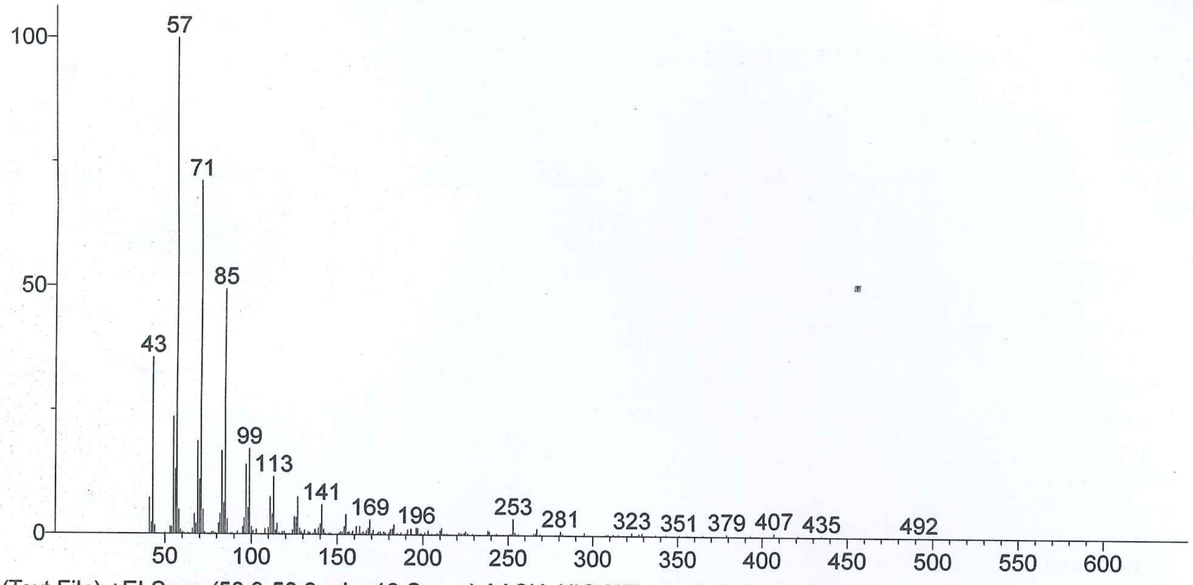

(Text File) +EI Scan (50.9-50.9 min, 12 Scans) AASIA-KIG-HEX-2a-040622.D Subtract

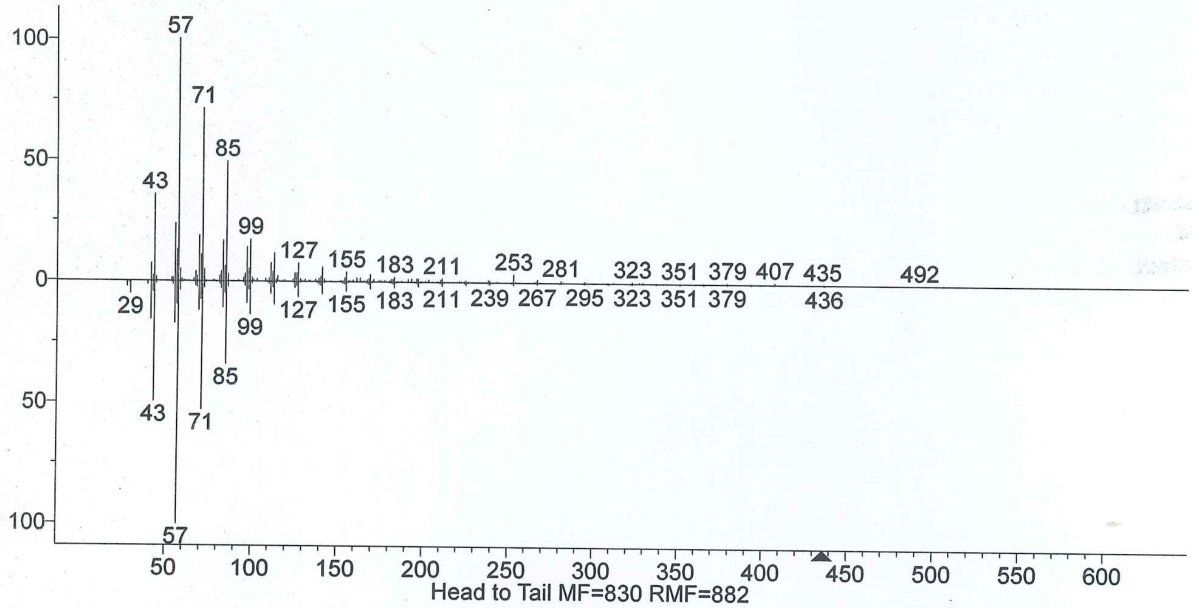

Head to Tail MF=830 RMF=882

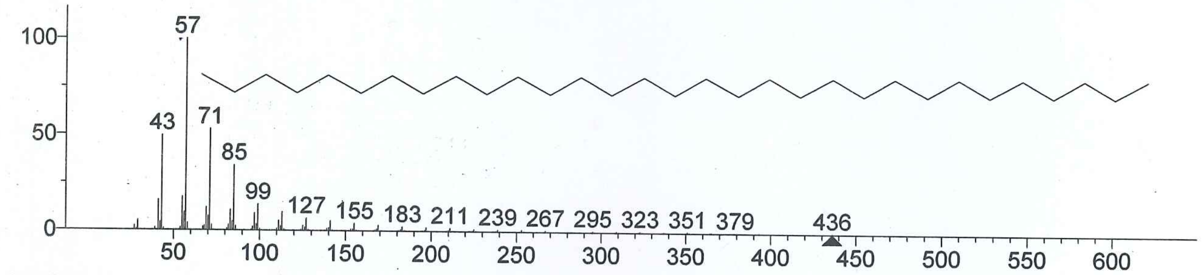

(replib) Hentriacontane

Name: Hentriacontane
Formula: $C_{31}H_{64}$
MW: 436 CAS#: 630-04-6 NIST#: 150572 ID#: 5728 DB: replib
Other DBs: None
Contributor: Chemical Concepts
10 largest peaks:
57 999 | 71 528 | 43 494 | 85 340 | 55 173 | 41 157 | 99 136 | 69 120 | 83 107 | 56 95 |
Synonyms:
1.n-Hentriacontane
2.Untriacontane

Estimated non-polar retention index (n-alkane scale):
Value: 3103 iu
Confidence interval (Hydrocarbons): 39(50%) 167(95%) iu

Retention index.
1. Value: 3100 iu
Column Class: All column types
Data Type: Normal alkane RI value specified by scale definition
Source: von Kováts, E., 206. Gas-chromatographische Charakterisierung organischer Verbindungen. Teil 1: Retentionsindices aliphatischer Halogenide, Alkohole, Aldehyde und Ketone, Helv. Chim. Acta, 41(7), 1958, 1915 -1932.

2. Value: 472.7 iu
Column Type: Capillary
Column Class: Semi-standard non-polar
Active Phase: DB
-5
Column Length: 60 m
Data Type: Lee RI
Program Type: Ramp
Source: Fuentes, M.J.; Font, R.; Gomez-Rico, M.F.; Martin-Gullon, I., Pyrolysis and combustion of waste lubricant oil from diesel cars: Decomposition and pollutants, J. Anal. Appl. Pyrolysis, 79, 2007, 215-226.

<...>

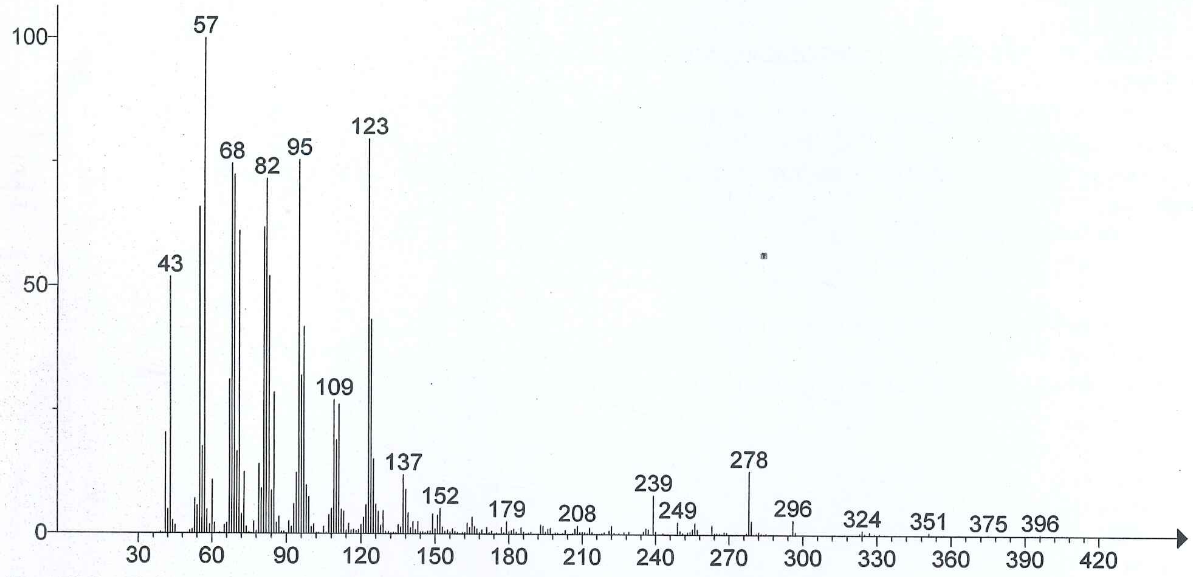

(Text File) +EI Scan (51.7-51.7 min, 17 Scans) AASIA-KIG-HEX-2a-040622.D Subtract

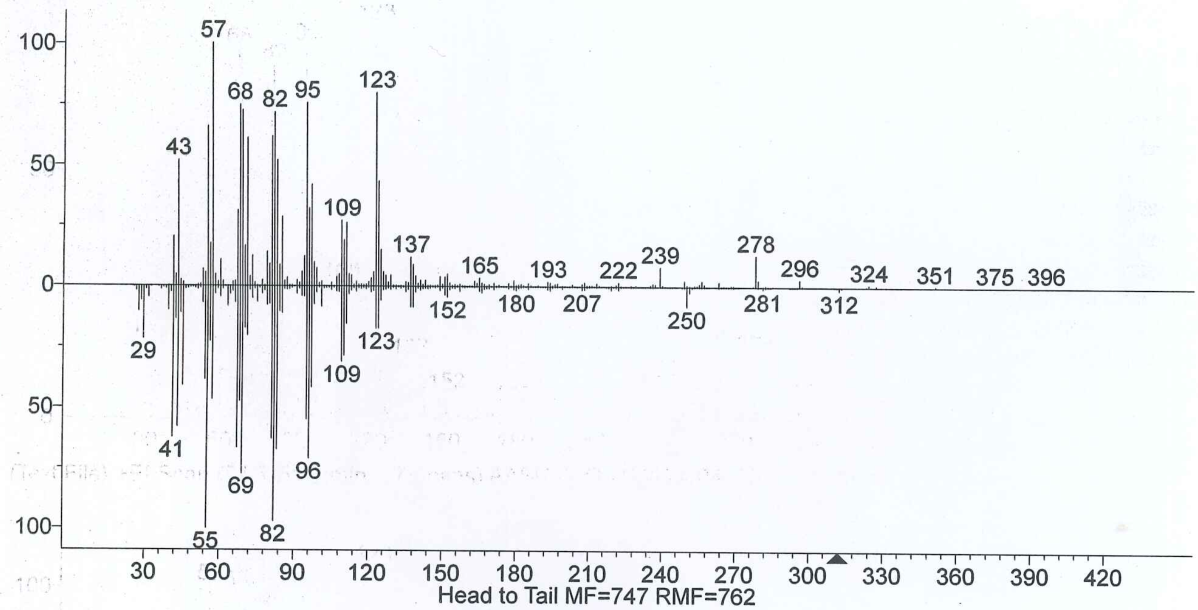

Head to Tail MF=747 RMF=762

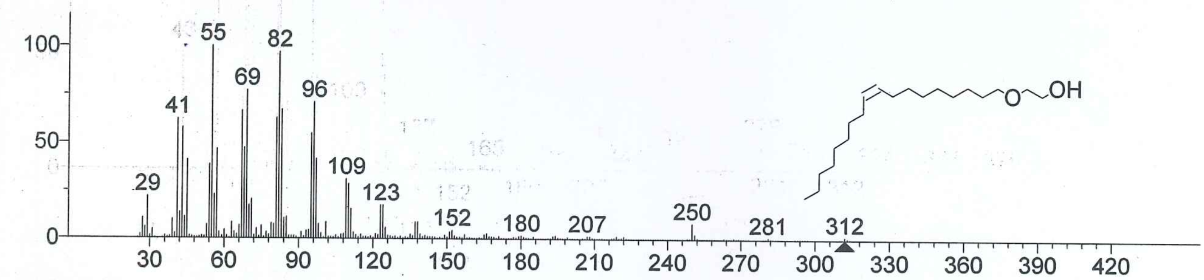

(mainlib) Ethanol, 2-(9-octadecenyloxy)-, (Z)-

Name: Ethanol, 2-(9-octadecenyloxy)-, (Z)-
Formula: $C_{20}H_{40}O_2$
MW: 312 CAS#: 5353-25-3 NIST#: 36235 ID#: 18654 DB: mainlib
Other DBs: None
Contributor: R.T.HOLMAN,UNIVERSITY OF MINNESOTA
10 largest peaks:
55 999 | 82 968 | 69 770 | 96 708 | 83 670 | 67 664 | 81 627 | 41 621 | 43 577 | 95 546 |
Synonyms:
1.2-cis-9-Octadecenyloxyethanol
2.2-[(9Z)-9-Octadecenyloxy]ethanol #

Estimated non-polar retention index (n-alkane scale):
Value: 2336 iu
Confidence interval (Diverse functional groups): 89(50%) 382(95%) iu

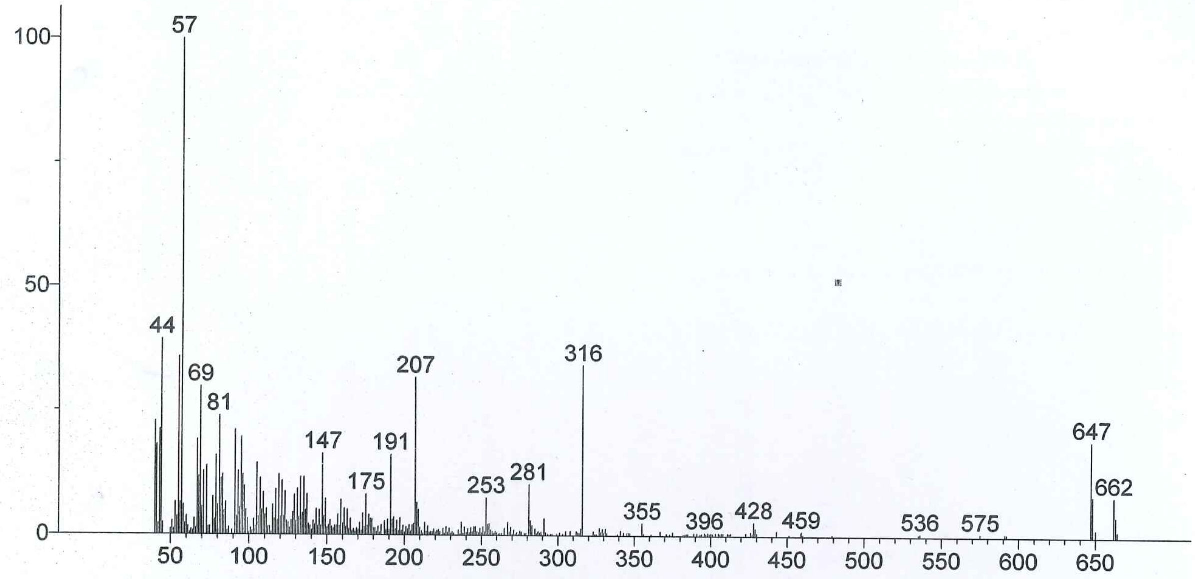

(Text File) +EI Scan (52.6 min) AASIA-KIG-HEX-2a-040622.D

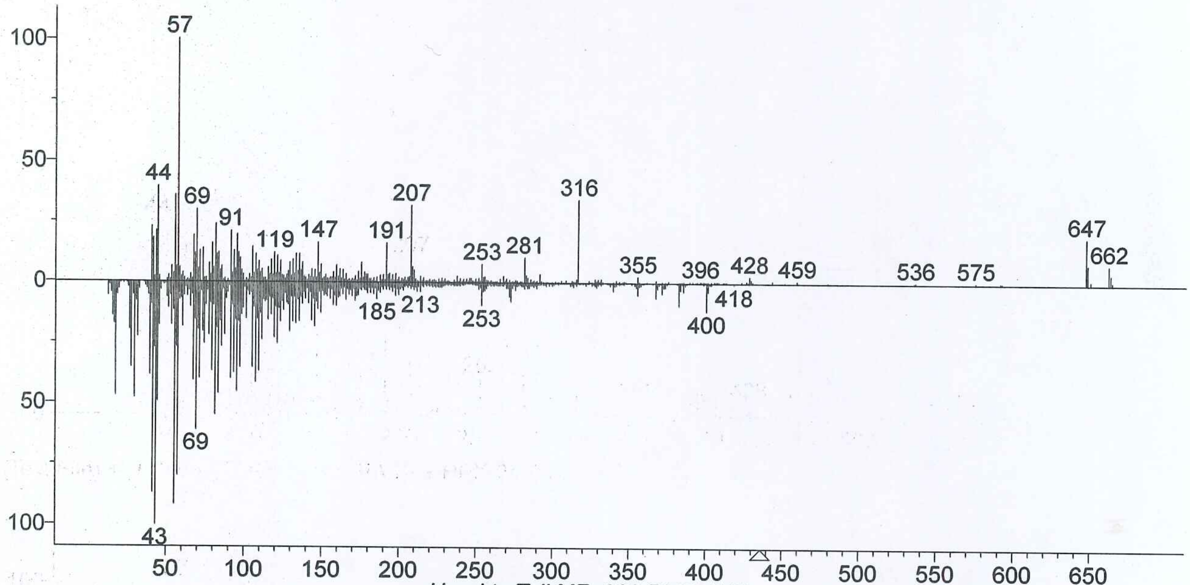

Head to Tail MF=662 RMF=742

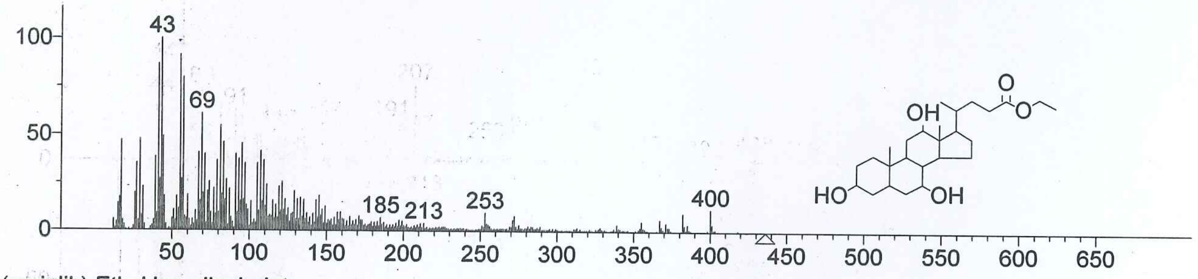

(mainlib) Ethyl iso-allocholate

Name: Ethyl iso-allocholate
Formula: $C_{26}H_{44}O_5$
MW: 436 NIST#: 43053 ID#: 6556 DB: mainlib
Contributor: R RYHAGE MS-LAB KAROLINSKA INSTITUTET STOCKHOLM SWEDEN
10 largest peaks:
43 999 | 55 914 | 41 867 | 57 797 | 69 609 | 81 547 | 44 492 | 29 476 | 17 469 | 83 460 |
Synonyms:
no synonyms.

Estimated non-polar retention index (n-alkane scale):
Value: 3094 iu
Confidence interval (Low reliability): 174(50%) 752(95%) iu

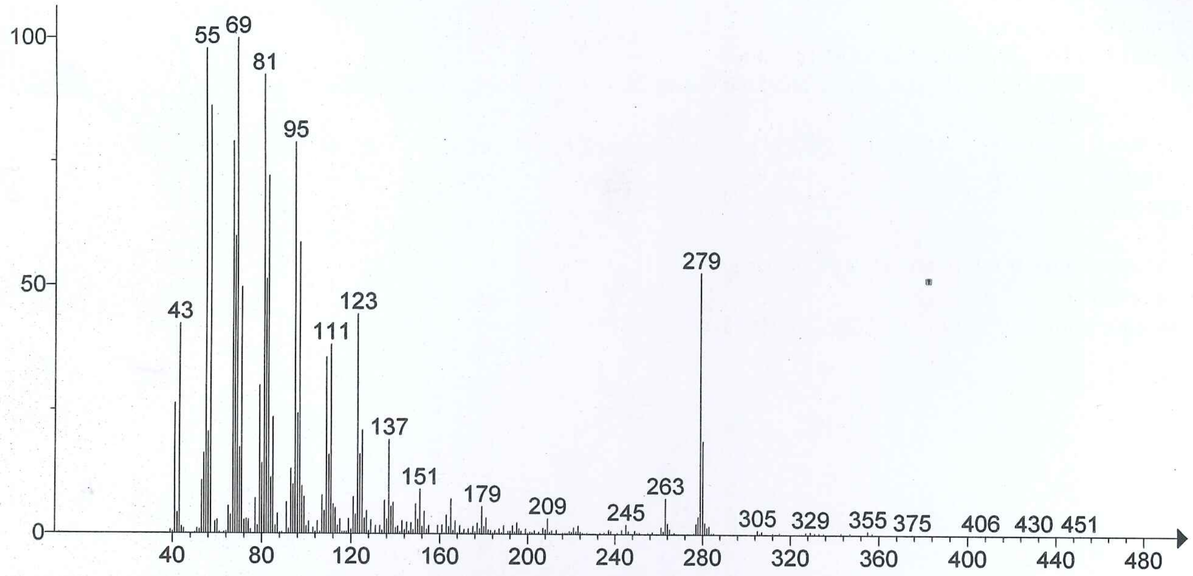

(Text File) +EI Scan (54.7-54.8 min, 24 Scans) AASIA-KIG-HEX-2a-040622.D Subtract

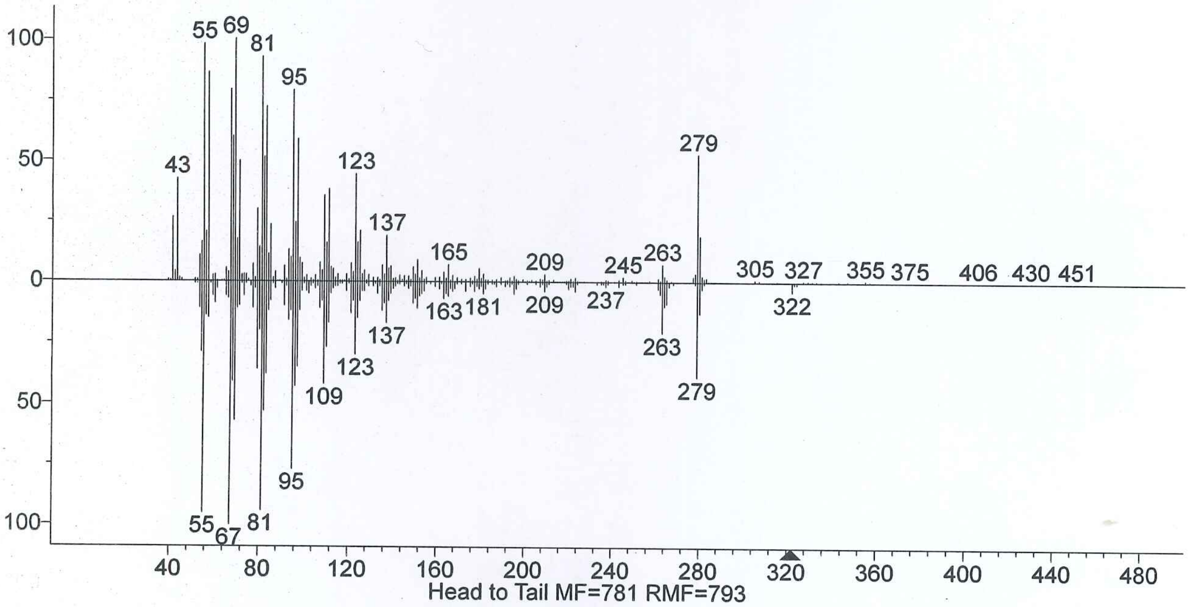

Head to Tail MF=781 RMF=793

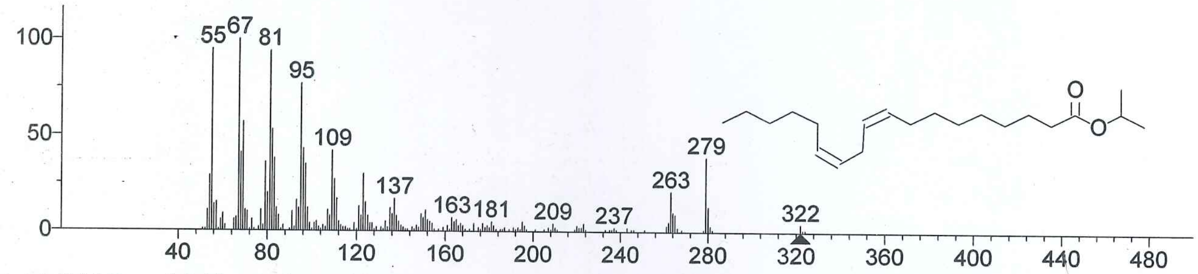

(mainlib) i-Propyl 9,12-octadecenadienoate

Name: i-Propyl 9,12-octadecenadienoate
Formula: $C_{21}H_{38}O_2$
MW: 322 NIST#: 336798 ID#: 28653 DB: mainlib
Contributor: William W. Christie, Mylnefield Lipid Analysis, Invergowrie, Dundee, Scotland, UK
10 largest peaks:
67 999 | 55 949 | 81 939 | 95 769 | 69 569 | 82 529 | 96 429 | 109 419 | 68 409 | 279 389 |
Synonyms:
no synonyms.

Estimated non-polar retention index (n-alkane scale):
Value: 2228 iu
Confidence interval (Esters): 47(50%) 201(95%) iu

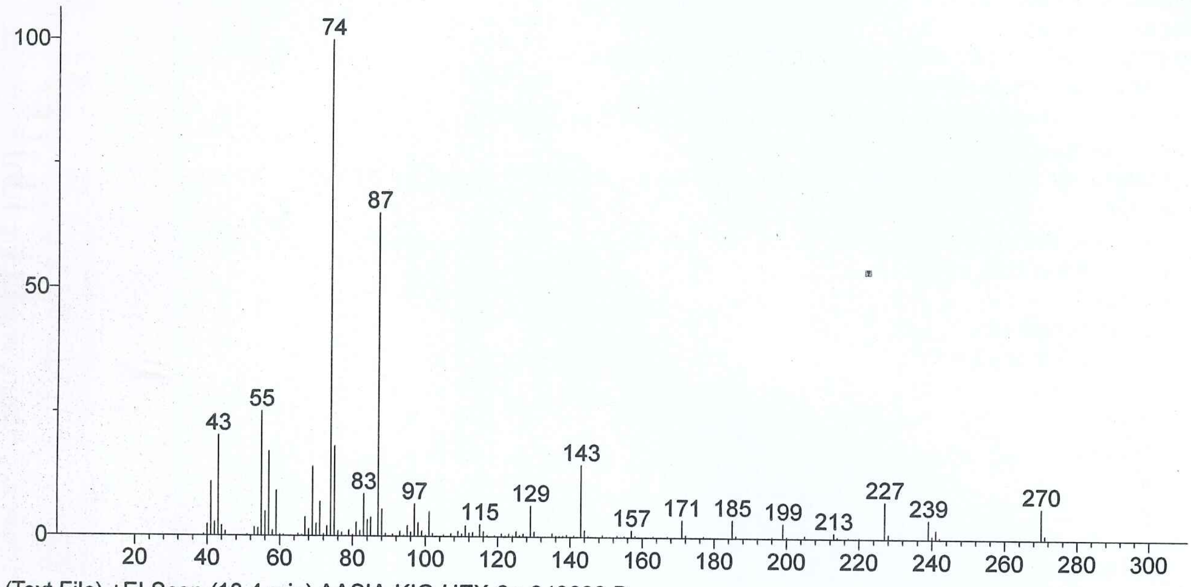

(Text File) +EI Scan (18.4 min) AASIA-KIG-HEX-2a-040622.D

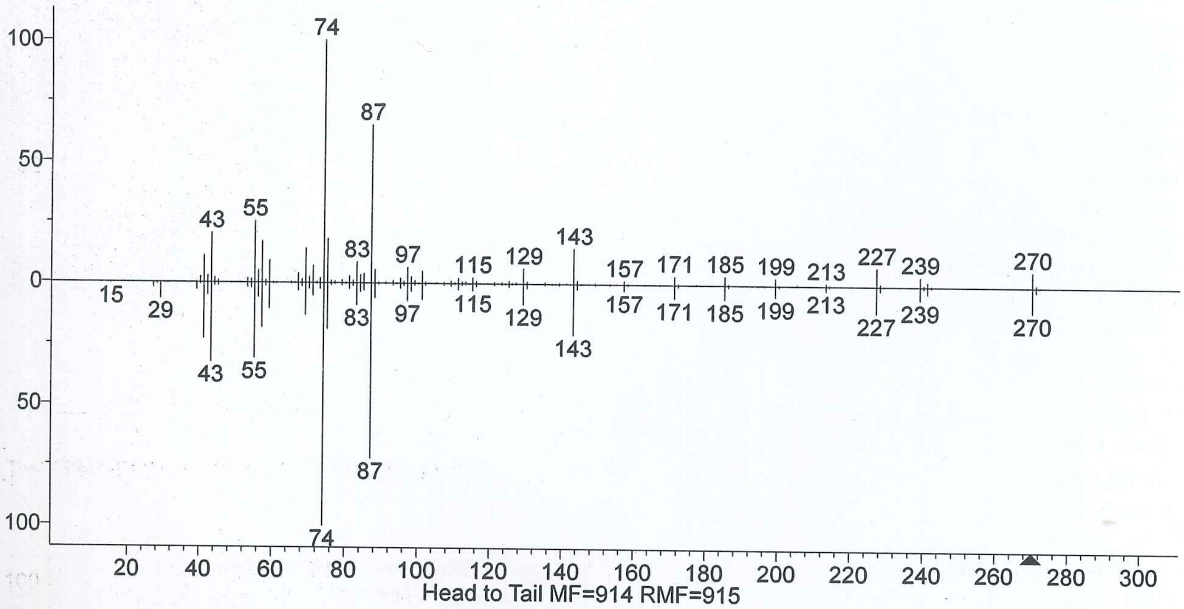

Head to Tail MF=914 RMF=915

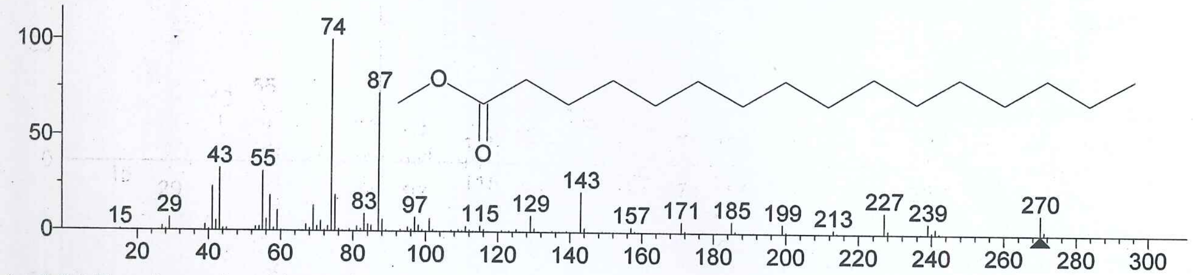

(mainlib) Hexadecanoic acid, methyl ester

Name: Hexadecanoic acid, methyl ester
Formula: $C_{17}H_{34}O_2$
MW: 270 CAS#: 112-39-0 NIST#: 333716 ID#: 38248 DB: mainlib
Other DBs: Fine, TSCA, EPA, HODOC, NIH, EINECS
Contributor: NIST Mass Spectrometry Data Center
10 largest peaks:
74 999 | 87 720 | 43 325 | 55 310 | 41 228 | 143 208 | 75 188 | 57 183 | 69 132 | 227 110 |
Synonyms:
1.Palmitic acid, methyl ester
2.n-Hexadecanoic acid methyl ester
3.Metholene 2216
4.Methyl hexadecanoate
5.Methyl n-hexadecanoate
6.Methyl palmitate
7.Uniphat A60
8.Emery 2216
9.Radia 7120

Estimated non-polar retention index (n-alkane scale):
Value: 1878 iu
Confidence interval (Esters): 47(50%) 201(95%) iu

Retention index.
1. Value: 1908 iu
Column Type: Capillary
Column Class: Standard non-polar
Active Phase: SPB-1
Column
Length: 30 m
Carrier Gas: He
Column Diameter: 0.25 mm
Phase Thickness: 0.25 um
Data Type: Linear
RI
Program Type: Ramp
Start T: 50 C
End T: 250 C
Heat Rate: 5 K/min
Start Time: 3 min
End Time: 15
min
Source: Blagojevic, P.; Radulovic, N.; Palic, R.; Stojanovic, G., Chemical composition of the essential oils of Serbian wild-growing Srtemisia absinthium and Artemisia vulgaris, J. Agric. Food Chem., 54, 2006, 4780-4789.

2.
Value: 1909 iu
Column Type: Capillary
Column Class: Standard non-polar
Active Phase: SPB-1
Column Length:
30 m
Carrier Gas: He
Column Diameter: 0.25 mm
Phase Thickness: 0.25 um

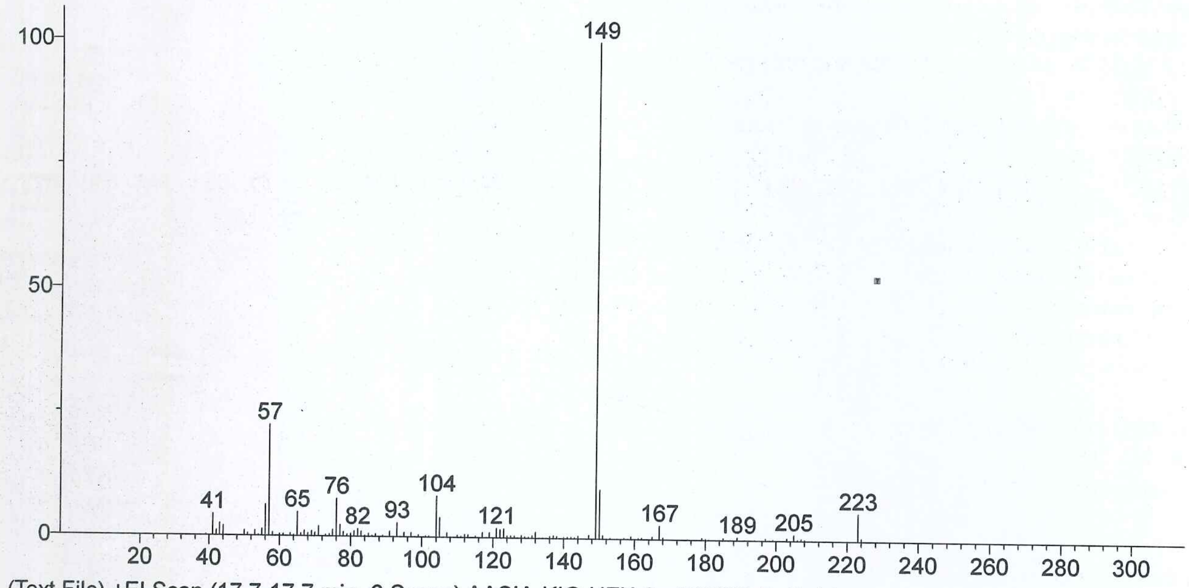

(Text File) +EI Scan (17.7-17.7 min, 6 Scans) AASIA-KIG-HEX-2a-040622.D Subtract

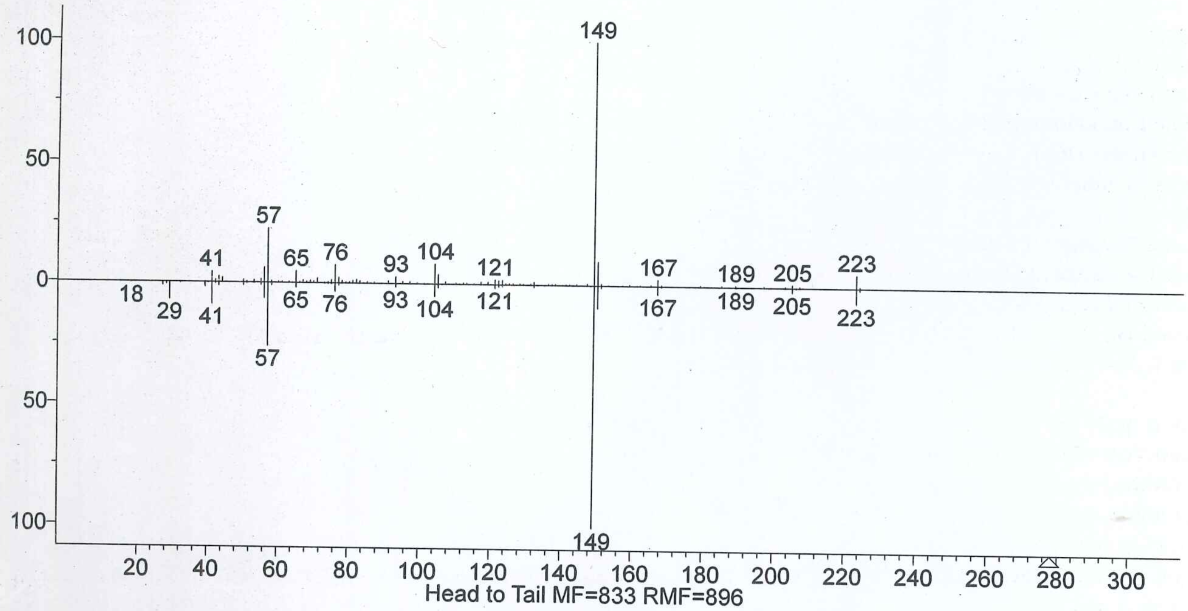

Head to Tail MF=833 RMF=896

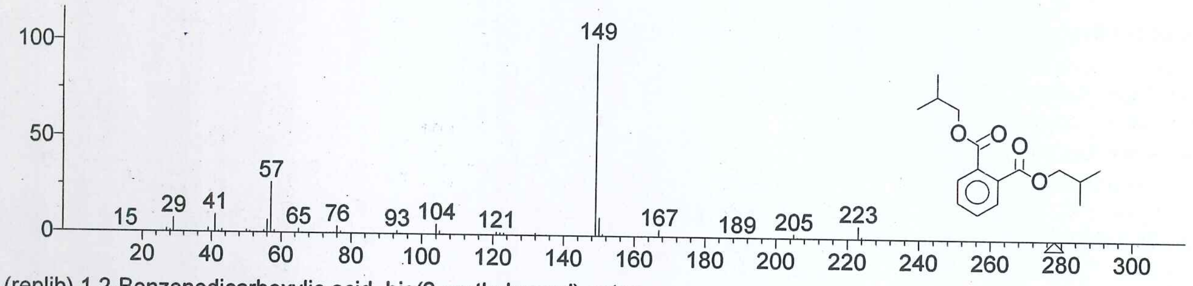

(replib) 1,2-Benzenedicarboxylic acid, bis(2-methylpropyl) ester

Name: 1,2-Benzenedicarboxylic acid, bis(2-methylpropyl) ester
Formula: $C_{16}H_{22}O_4$
MW: 278 CAS#: 84-69-5 NIST#: 229506 ID#: 19975 DB: replib
Other DBs: Fine, TSCA, RTECS, EPA, HODOC, NIH, EINECS, IRDB
Contributor: Japan AIST/NIMC Database- Spectrum MS-NW-2701
10 largest peaks:
149 999 | 57 261 | 150 94 | 41 91 | 29 72 | 56 65 | 223 64 | 104 51 | 76 35 | 167 34 |
Synonyms:
1.Phthalic acid, diisobutyl ester
2.Diisobutyl phthalate
3.Hexaplas M/1B
4.Isobutyl phthalate
5.Palatinol IC
6.Dibp
7.Diisobutylester kyseliny ftalove
8.Kodaflex DIBP
9.Palatinol 1C
10.Uniplex 155

Estimated non-polar retention index (n-alkane scale):
Value: 1908 iu
Confidence interval (Esters): 47(50%) 201(95%) iu

Retention index.
1. Value: 1819 iu
Column Type: Capillary
Column Class: Standard non-polar
Active Phase: DB-1
Column
Length: 30 m
Column Diameter: 0.2 mm
Phase Thickness: 0.25 um
Data Type: Linear RI
Program Type:
Ramp
Start T: 50 C
End T: 300 C
Heat Rate: 5 K/min
Start Time: 2 min
End Time: 5 min
Source: Johnson, C.
I.; Urso, A.; Geleta, L., Broad spectrum analysis of municipal and industrial effluents discharged into the Peace, Athabasca and Slave river basins: characterization of effluent samples, 1994 - Volume 1 of 2, Northern River Basins Study Project Report No. 121, Norther River Basins Study, Edmonton, Alberta, 1997, 27.

2. Value: 1863
iu
Column Type: Packed
Column Class: Standard non-polar
Active Phase: SE-30
Column Length: 1.5
m
Carrier Gas: He
Substrate: Chromosorb G HP (80-100 mesh)

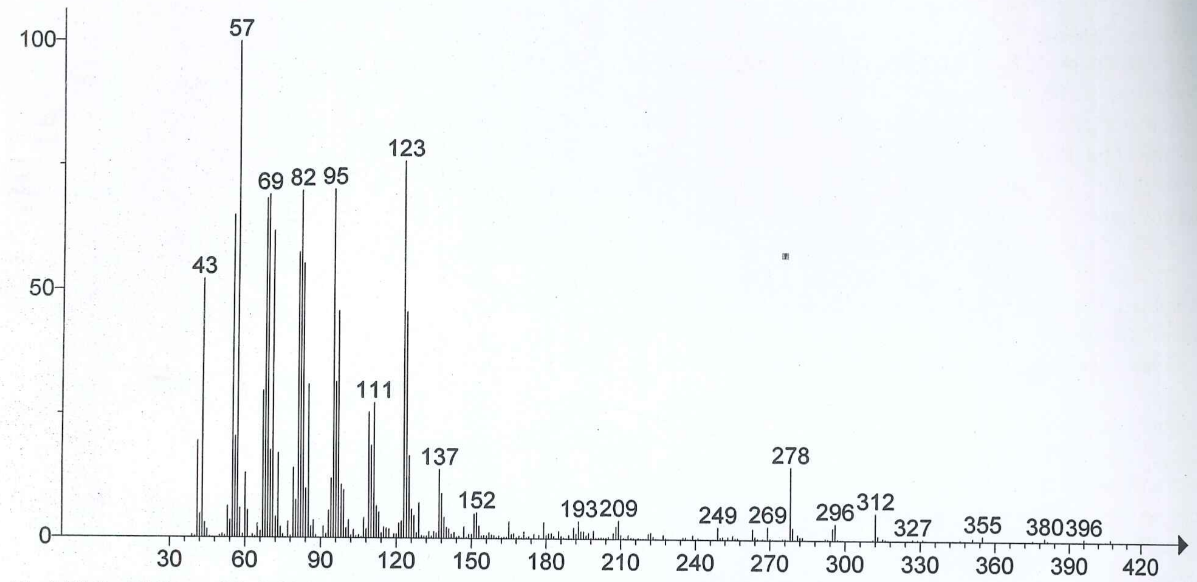

(Text File) +EI Scan (59.9-60.0 min, 29 Scans) AASIA-KIG-HEX-2a-040622.D Subtract

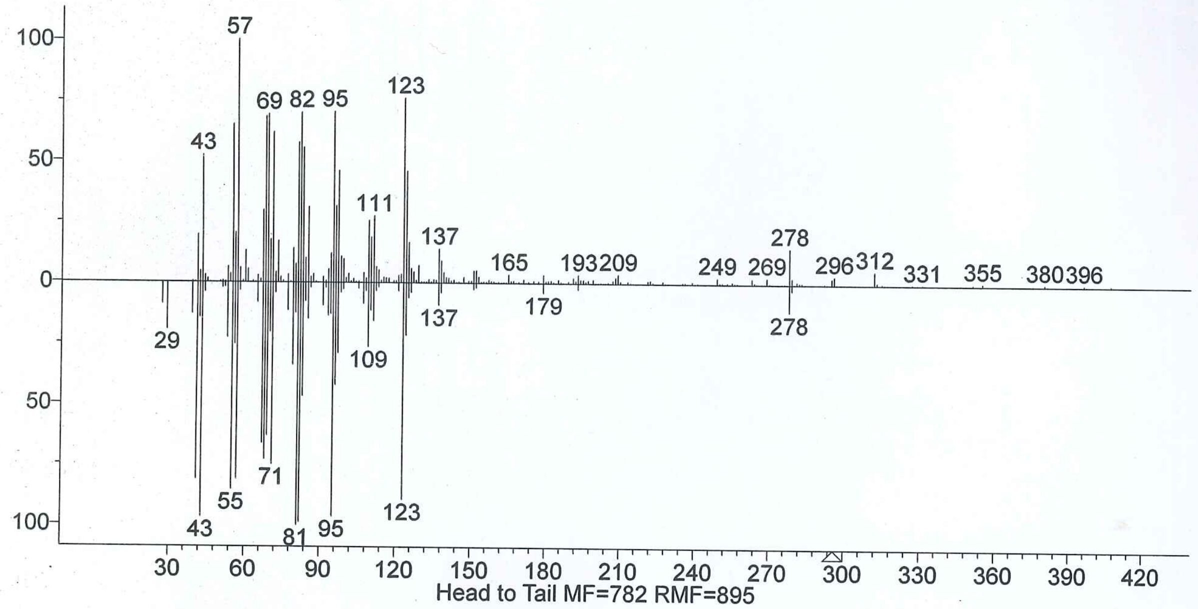

Head to Tail MF=782 RMF=895

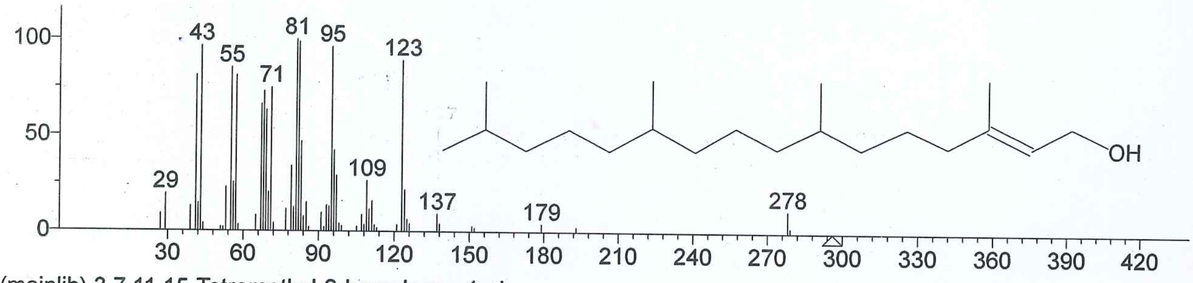

(mainlib) 3,7,11,15-Tetramethyl-2-hexadecen-1-ol

Name: 3,7,11,15-Tetramethyl-2-hexadecen-1-ol
Formula: $C_{20}H_{40}O$
MW: 296 CAS#: 102608-53-7 NIST#: 114703 ID#: 43206 DB: mainlib
Other DBs: IRDB
Contributor: NIST Mass Spectrometry Data Center, 1990.
10 largest peaks:
81 999 | 82 986 | 43 965 | 95 962 | 123 892 | 55 852 | 41 811 | 57 811 | 71 748 | 68 728 |
Synonyms:
1.(2E)-3,7,11,15-Tetramethyl-2-hexadecen-1-ol #

Estimated non-polar retention index (n-alkane scale):
Value: 2045 iu
Confidence interval (Alcohols): 41(50%) 176(95%) iu

Retention index.
1. Value: 2119.33 iu
Column Type: Capillary
Column Class: Semi-standard non-polar
Active Phase: SE
-54
Column Length: 25 m
Column Diameter: 0.31 mm
Data Type: Linear RI
Program Type: Ramp
Start T: 35
C
End T: 230 C
Heat Rate: 4 K/min
Start Time: 3 min
End Time: 10 min
Source: Yin, W.; Xiu, Z.; Aijin, H.,
Analysis of the volatile components in trogopterorum feces by capillary gas chromatography and gas chromatography/mass spectrometry, Fenxi Huaxue, 29(2), 2001, 195-198.

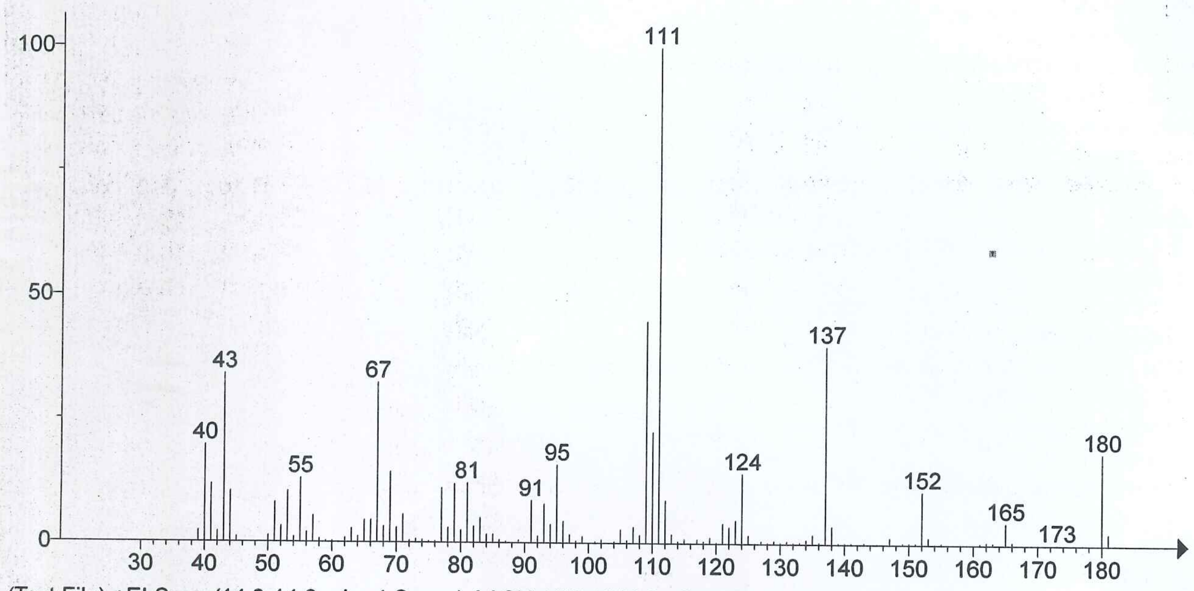

(Text File) +EI Scan (14.0-14.0 min, 4 Scans) AASIA-KIG-HEX-2a-040622.D

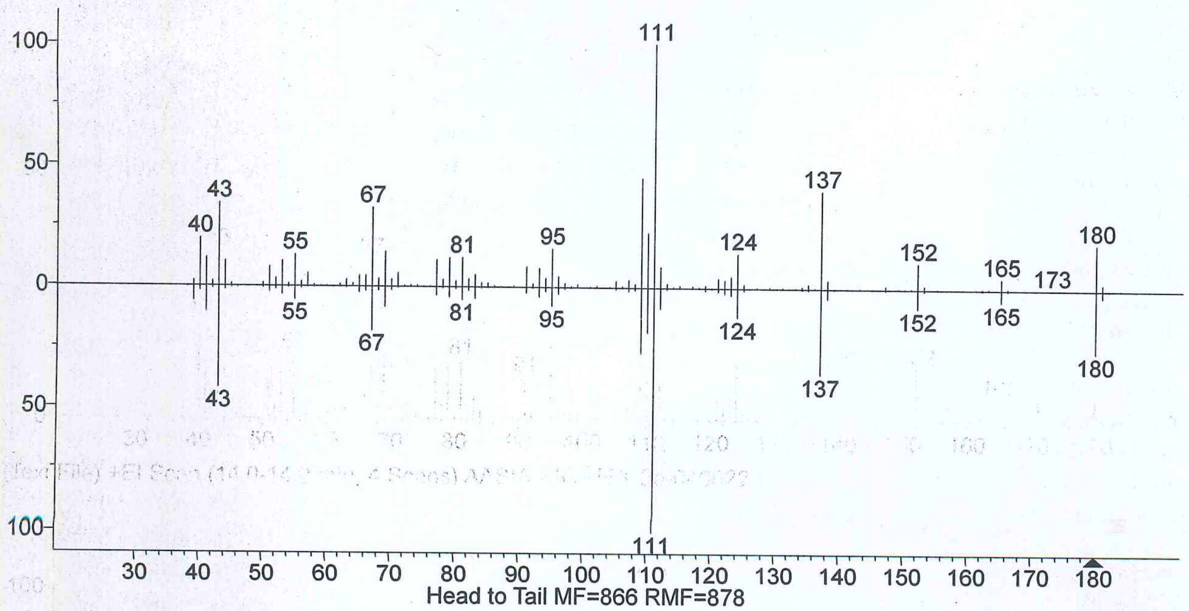

Head to Tail MF=866 RMF=878

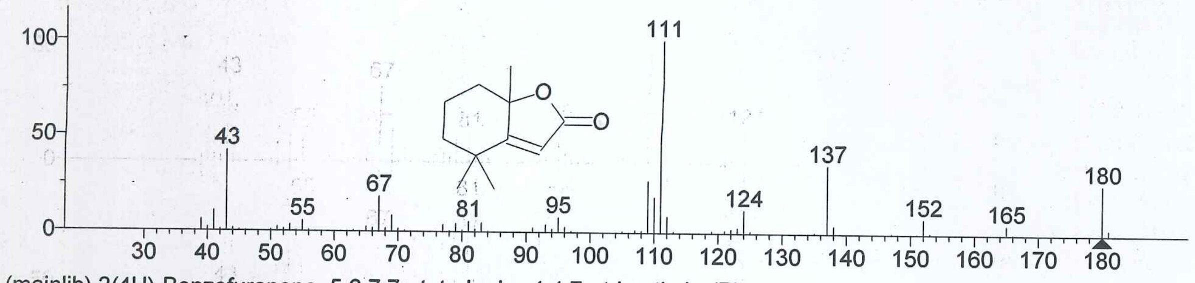

(mainlib) 2(4H)-Benzofuranone, 5,6,7,7a-tetrahydro-4,4,7a-trimethyl-, (R)-

Name: 2(4H)-Benzofuranone, 5,6,7,7a-tetrahydro-4,4,7a-trimethyl-, (R)-
Formula: $C_{11}H_{16}O_2$
MW: 180 CAS#: 17092-92-1 NIST#: 108912 ID#: 74534 DB: mainlib
Other DBs: NIH
Contributor: Philip Morris R&D
10 largest peaks:
111 999 | 43 418 | 137 353 | 109 268 | 180 259 | 110 184 | 67 181 | 124 119 | 41 106 | 112 85 |
Synonyms:
1.2(4H)-Benzofuranone, 5,6,7,7a-tetrahydro-4,4,7a-trimethyl-
2.Actinidiolide, dihydro-
3.2(4H)-Benzofuranone, 5,6,7,7a-tetrahydro-4,4,7a-trimethyl-, (S)-
4.Dihydroactinidiolide
5.4,4,7a-Trimethyl-5,6,7,7a-tetrahydro-1-benzofuran-2(4H)-one #

Estimated non-polar retention index (n-alkane scale):
Value: 1426 iu
Confidence interval (Esters): 47(50%) 201(95%) iu

Retention index.
1. Value: 1532 iu
Column Type: Capillary
Column Class: Standard non-polar
Active Phase: HP-1
Column
Length: 50 m
Carrier Gas: He
Column Diameter: 0.32 mm
Phase Thickness: 1.05 um
Data Type: Linear
RI
Program Type: Complex
Description: 20C(0.5min) =>60C=>4C/min=>250C
Source: Sing, A.S.C.; Smadja, J.;
Brevard, H.; Maignial, L.; Chaintreau, A.; Marion, J.-P., Volatile constituents of faham (Jumellea fragrans (Thou.) Schltr.), J. Agric. Food Chem., 40, 1992, 642-646.

2. Value: 1471 iu
Column Type: Capillary
Column Class:
Standard non-polar
Active Phase: SPB-1
Column Length: 30 m
Carrier Gas: He
Column Diameter: 0.32
mm
Phase Thickness: 0.25 um
Data Type: Kovats RI
Program Type: Complex
Description: 40C(3min)
=>2C/min =>100C=>4C/min =>220C (7min)
Source: Borse, B.B.; Jagan Mohan Rao, L.; Nagalakshmi, S.;
Krishnamurthy, N., Fingerprint of black teas from India: identification of the regio-specific characteristics, Food Chem., 79, 2002, 419-424.

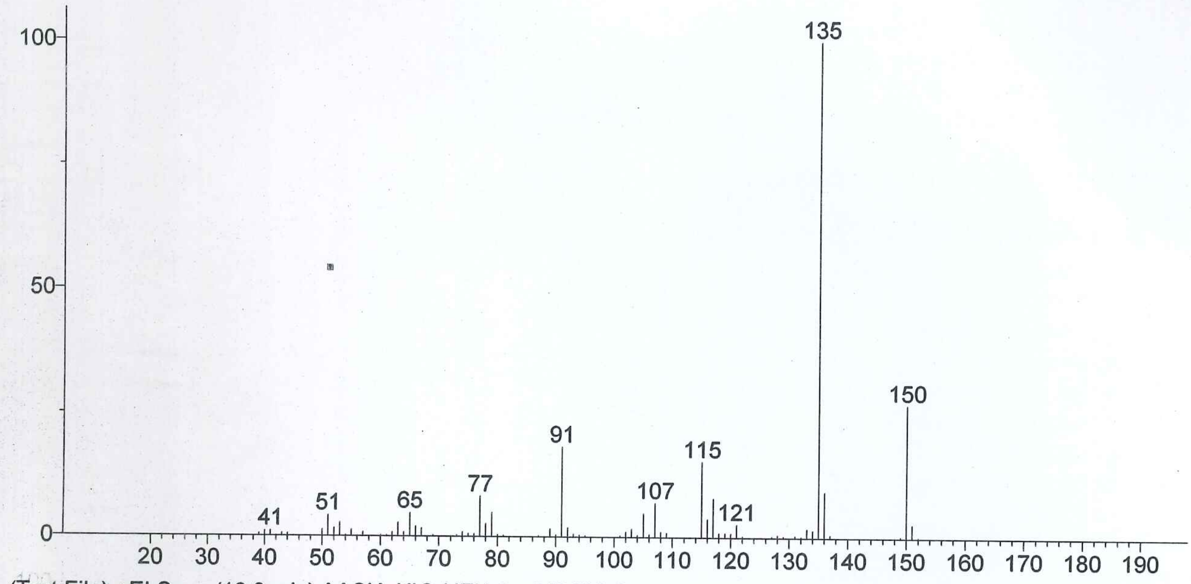

(Text File) +EI Scan (10.8 min) AASIA-KIG-HEX-2a-040622.D

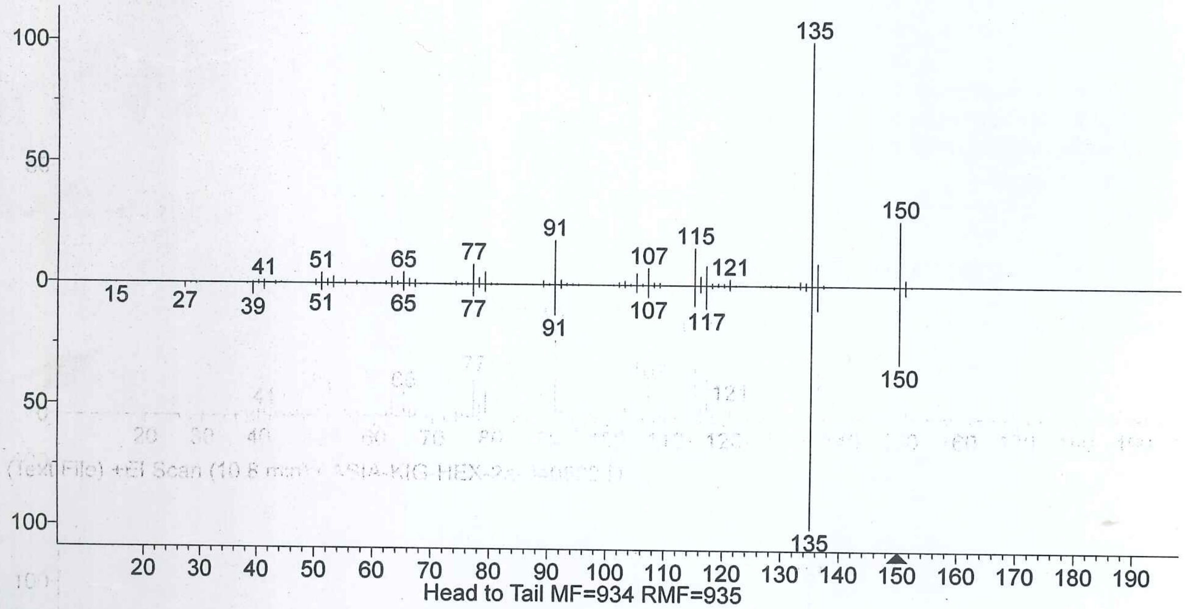

Head to Tail MF=934 RMF=935

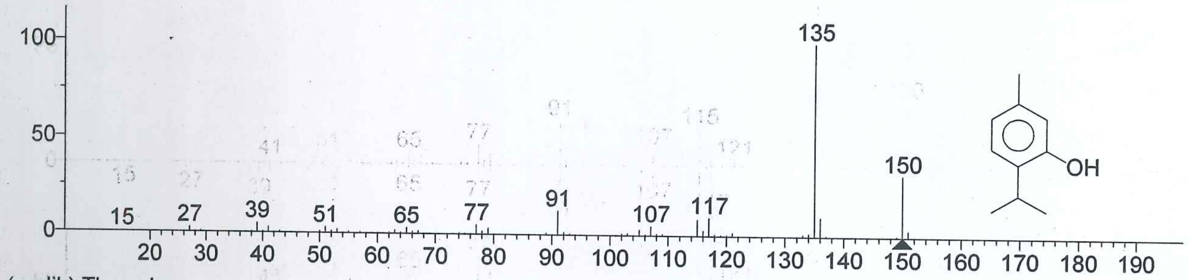

(replib) Thymol

Name: Thymol
Formula: $C_{10}H_{14}O$
MW: 150 CAS#: 89-83-8 NIST#: 229220 ID#: 18429 DB: replib
Other DBs: Fine, TSCA, RTECS, USP, HODOC, NIH, EINECS
Contributor: Japan AIST/NIMC Database- Spectrum MS-NW-1469
10 largest peaks:
135 999 | 150 320 | 91 125 | 136 99 | 117 94 | 115 85 | 77 49 | 107 49 | 39 48 | 151 34 |
Synonyms:
1.Phenol, 5-methyl-2-(1-methylethyl)-
2.p-Cymen-3-ol
3.Thyme camphor
4.2-Isopropyl-5-methylphenol
5.3-Hydroxy-p-cymene
6.3-Methyl-6-isopropylphenol
7.5-Methyl-2-isopropylphenol
8.6-Isopropyl-m-cresol
9.6-Isopropyl-3-methylphenol
10.m-Cresol, 6-isopropyl-
11.p-Cymene, 3-hydroxy-
12.Isopropyl cresol
13.Phenol, 2-isopropyl-5-methyl-
14.Thymic acid
15.1-Hydroxy-5-methyl-2-isopropylbenzene
16.1-Methyl-3-hydroxy-4-isopropylbenzene
17.3-p-Cymenol
18.3-Hydroxy-1-methyl-4-isopropylbenzene
19.5-Methyl-2-isopropyl-1-phenol
20.5-Methyl-2-(1-methylethyl)phenol
21.Isopropyl-m-cresol
22.m-Thymol

Estimated non-polar retention index (n-alkane scale):
Value: 1262 iu
Confidence interval (Phenols): 70(50%) 301(95%) iu

Retention index.
1. Value: 1266 iu
Column Type: Capillary
Column Class: Standard non-polar
Active Phase: RTX-1
Column
Length: 60 m
Carrier Gas: He
Column Diameter: 0.22 mm
Phase Thickness: 0.25 um
Data Type: Linear
RI
Program Type: Ramp
Start T: 60 C
End T: 230 C
Heat Rate: 2 K/min
End Time: 30 min
Source: Bendahou,
M.; Muselli, A.; Grignon-Dubois, M.; Benyoucef, M.; Desjobert, J.-M.; Bernardini, A.-F.; Costa, J., Antimicrobial
