# Supplementary material for: Gene expression and anticancer evaluation of Kigelia africana (Lam.) Benth. Extracts using MDA-MB-231 and MCF-7 cell lines
Source: PLoS One. 2024 Jun 5;19(6):e0303134. doi: 10.1371/journal.pone.0303134 (PMC11152317; doi:10.1371/journal.pone.0303134)
Supplement: S4 Fig — (PDF) [file pone.0303134.s004.pdf]

**Sample Name**	AASIA-KIG-HEX-1a-040	**Position**	8	**Instrument Name**	GCMS TQQQ	**User Name**	HEJ-G-104-03\Adminis
**Inj Vol**		**InjPosition**		**SampleType**		**IRM Calibration Status**	Not Applicable
**Data Filename**	AASIA-KIG-HEX-1a-040	**ACQ Method**	AASIA KALSOOM .M	**Comment**		**Acquired Time**	6/5/2022 2:09:30 AM

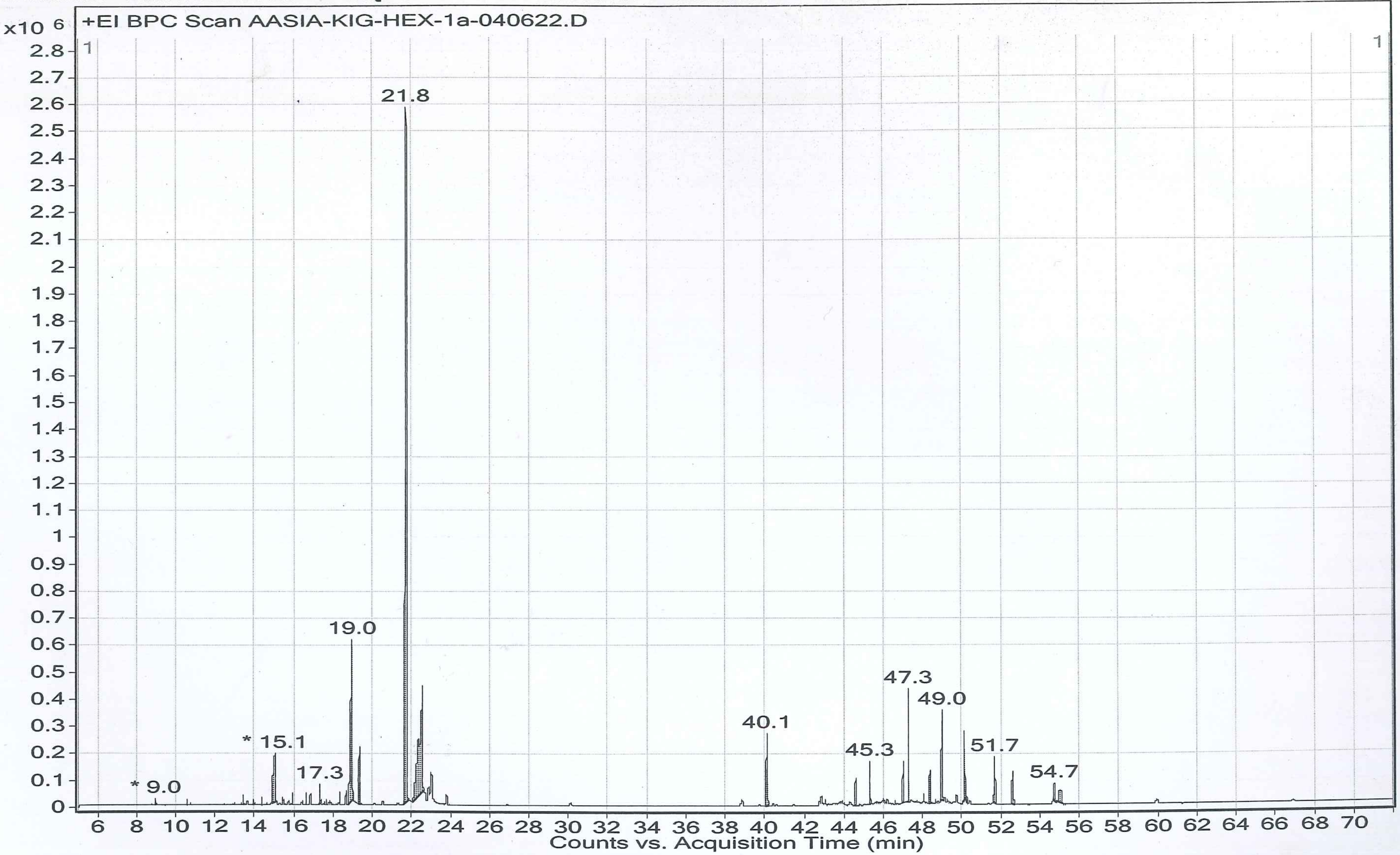

KIG-HEX-1A

Peak Number	RT	Area	Area %	Height	Width	Area Sum %	Height %
1	9	48802	0.39	23245	0.1	0.16	0.9
2	10.6	24800	0.2	20492	0	0.08	0.8
3	13.5	48048	0.38	36603	0	0.15	1.42
4	14	21582	0.17	17743	0	0.07	0.69
5	14.4	43193	0.34	31146	0	0.14	1.21
6	15.1	1464096	11.57	182457	0.3	4.67	7.1
7	17.3	128680	1.02	76011	0.1	0.41	2.96
8	19	3549815	28.06	596780	0.3	11.33	23.22
9	19.4	515844	4.08	216780	0.1	1.65	8.44
10	21.8	12649404	100	2569603	0.3	40.37	100
11	22.6	5361610	42.39	394294	0.6	17.11	15.34
12	40.1	1132425	8.95	266990	0.2	3.61	10.39
13	44.6	219605	1.74	100890	0.1	0.7	3.93
14	45.3	322939	2.55	156886	0.1	1.03	6.11
15	47	294304	2.33	152081	0.1	0.94	5.92
16	47.3	834961	6.6	415694	0.1	2.66	16.18
17	48.4	342992	2.71	121041	0.1	1.09	4.71
18	49	1427969	11.29	332456	0.2	4.56	12.94
19	50.1	750742	5.93	252936	0.1	2.4	9.84
20	50.2	139829	1.11	57589	0.1	0.45	2.24
21	51.7	688698	5.44	171057	0.2	2.2	6.66
22	52.6	557940	4.41	120159	0.2	1.78	4.68
23	54.7	331531	2.62	65401	0.2	1.06	2.55
24	55.1	432606	3.42	48892	0.3	1.38	1.9
						100	

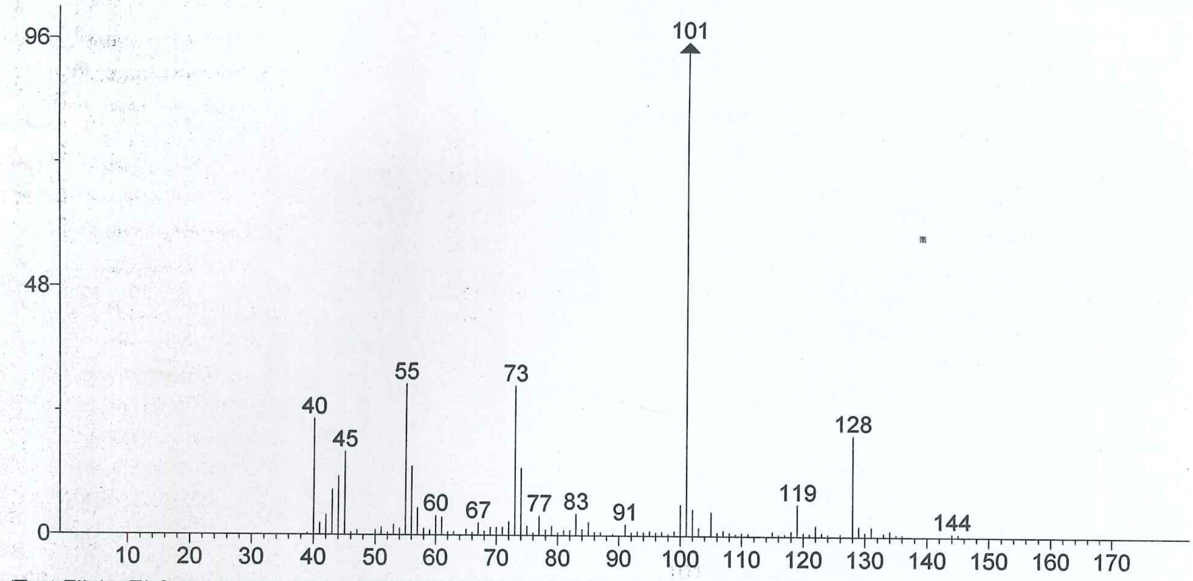

(Text File) +EI Scan (8.981 min) AASIA-KIG-HEX-1a-040622.D

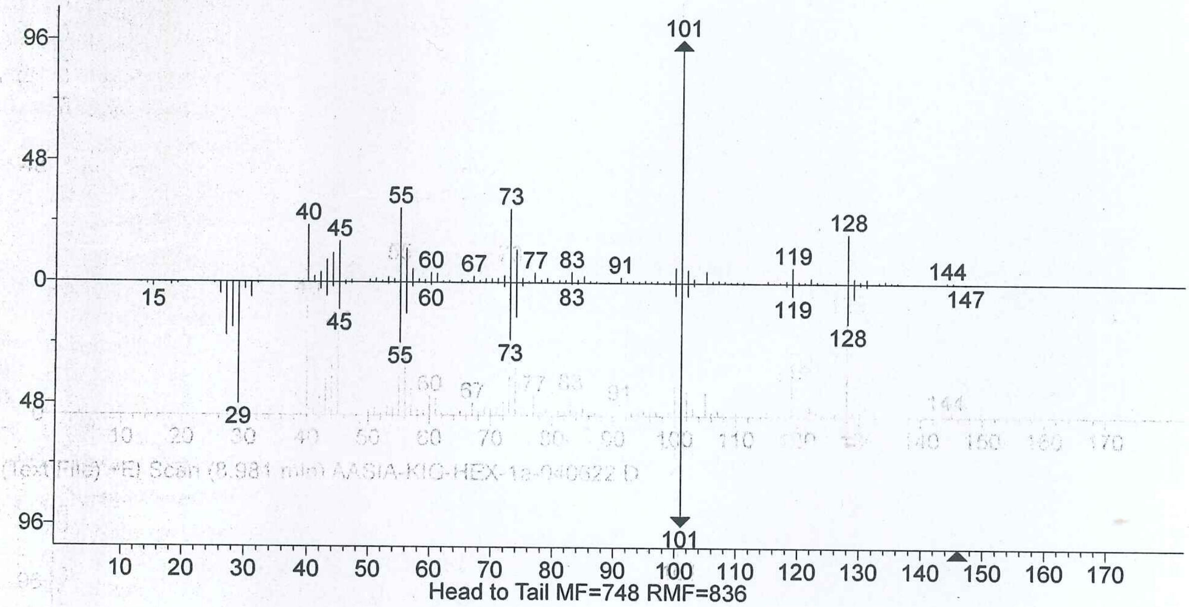

Head to Tail MF=748 RMF=836

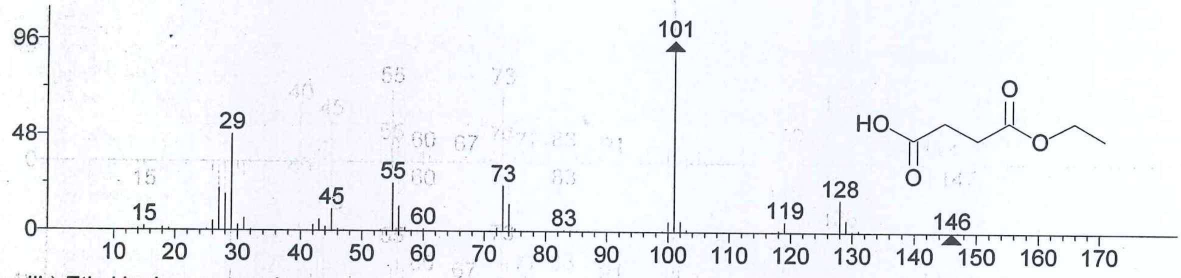

(replib) Ethyl hydrogen succinate

Name: Ethyl hydrogen succinate
Formula: $C_6H_{10}O_4$
MW: 146 CAS#: 1070-34-4 NIST#: 234868 ID#: 13223 DB: replib
Other DBs: Fine, TSCA, EINECS
Contributor: Japan AIST/NIMC Database- Spectrum MS-NW-5798
10 largest peaks:
101 999 | 29 489 | 55 242 | 73 229 | 27 213 | 28 181 | 128 161 | 74 137 | 56 125 | 45 109 |
Synonyms:
1.Butanedioic acid, monoethyl ester
2.4-Ethoxy-4-oxobutanoic acid #

Estimated non-polar retention index (n-alkane scale):
Value: 1141 iu
Confidence interval (Diverse functional groups): 89(50%) 382(95%) iu

Retention index.
1. Value: 1079.3 iu
Column Type: Capillary
Column Class: Standard non-polar
Active Phase: HP-1
Column
Length: 20 m
Carrier Gas: He
Data Type: Normal alkane RI
Program Type: Ramp
Start T: 40 C
End T: 280
C
Heat Rate: 6 K/min
Start Time: 3 min
Source: Wilkins, A.L.; Lu, Y.; Tan, S.-T., Extractives from New Zealand
honeys. 5. Aliphatic dicarboxylic acids in New Zealand rewarewa (Knightea excelsa) honey, J. Agric. Food Chem., 43, 1995, 3021-3025.

2. Value: 2440 iu
Column Type: Capillary
Column Class: Standard polar
Active Phase:
DB-Wax
Column Length: 30 m
Carrier Gas: H2
Column Diameter: 0.32 mm
Phase Thickness: 0.5 um
Data
Type: Linear RI
Program Type: Complex
Description: 60C(3min)=>2C/min=>220C=>3C/min=>245C (20min)

Source: Selli, S.; Canbas, A.; Cabaroglu, T.; Erten, H.; Gunata, Z., Aroma components of cv. Muscat of Bornova wines and influence of skin contact treatment, Food Chem., 94, 2006, 319-326.
<...>

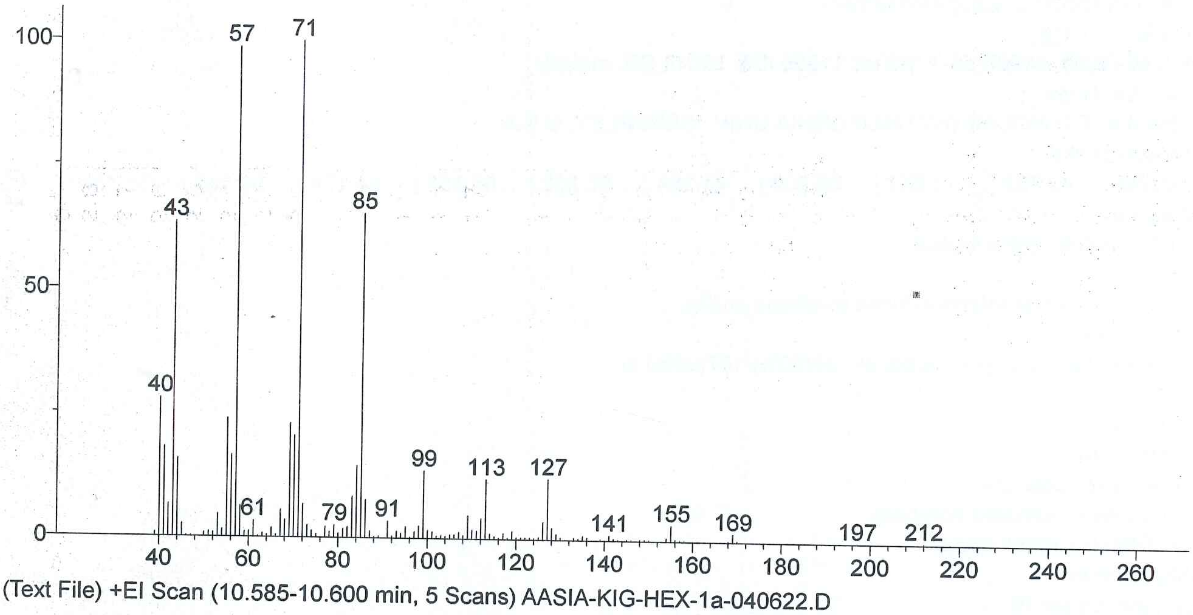

(Text File) +EI Scan (10.585-10.600 min, 5 Scans) AASIA-KIG-HEX-1a-040622.D

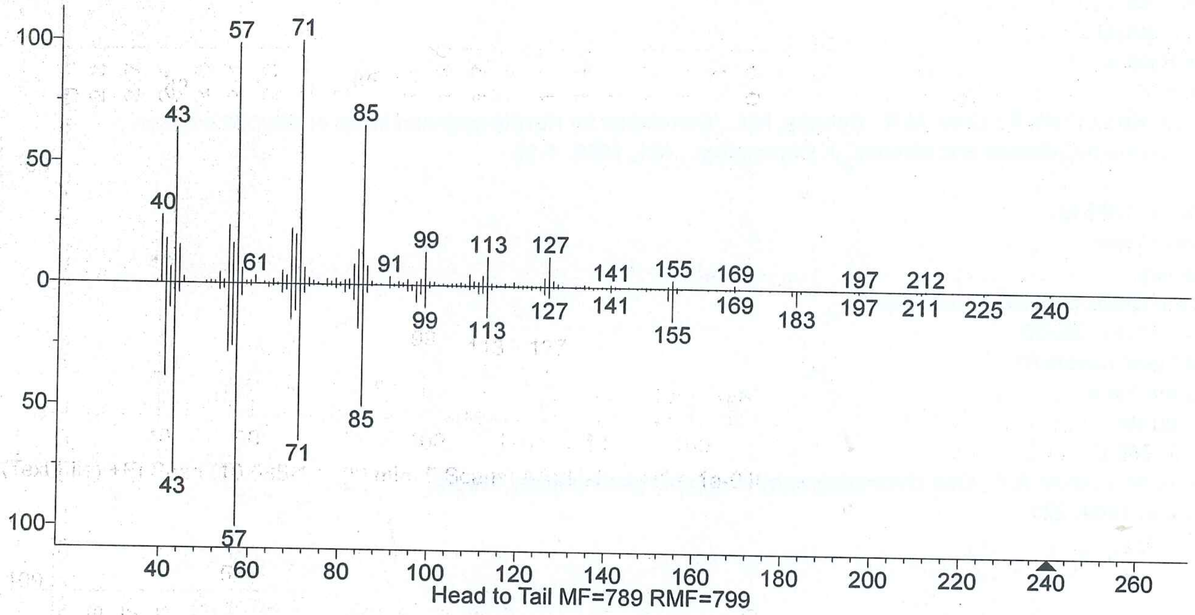

Head to Tail MF=789 RMF=799

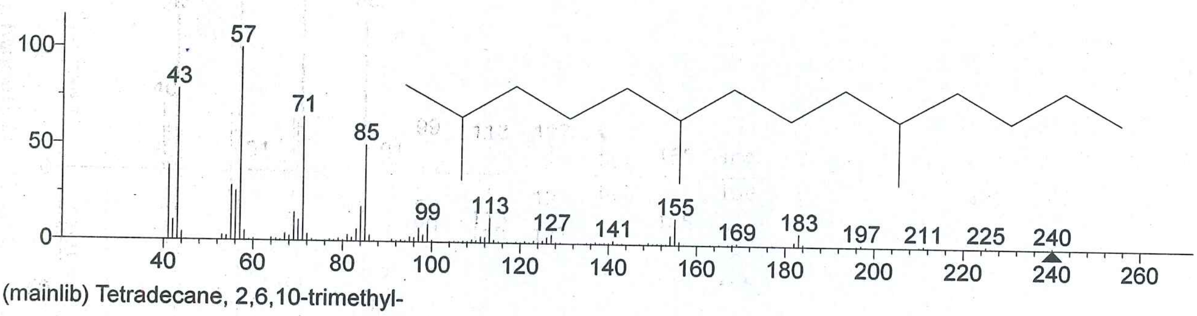

(mainlib) Tetradecane, 2,6,10-trimethyl-

Name: Tetradecane, 2,6,10-trimethyl-
Formula: $C_{17}H_{36}$
MW: 240 CAS#: 14905-56-7 NIST#: 11556 ID#: 22011 DB: mainlib
Other DBs: None
Contributor: E.D.MCCARTHY CALIFORNIA UNIV., BERKELEY, U.S.A
10 largest peaks:
57 999 | 43 782 | 71 641 | 85 500 | 41 384 | 55 282 | 56 256 | 84 179 | 69 147 | 155 134 |
Synonyms:
1.2,6,10-Trimethyltetradecane

Estimated non-polar retention index (n-alkane scale):
Value: 1519 iu
Confidence interval (Hydrocarbons): 39(50%) 167(95%) iu

Retention index.
1. Value: 1557 iu
Column Type: Capillary
Column Class: Standard non-polar
Active Phase: Cross-Linked
Methylsilicone
Data Type: Linear RI
Program Type: Ramp
Start T: 40 C
End T: 300 C
Heat Rate: 5
K/min
Source: Khorasheh, F.; Gray, M.R.; Selucky, M.L., Correlation for Kováts retention index of C9-C26 monoalkyl and polymethyl alkanes and alkenes, J. Chromatogr., 481, 1989, 1-16.

2. Value: 1555 iu
Column Type:
Capillary
Column Class: Standard non-polar
Active Phase: SE-30
Data Type: Kovats RI
Program Type:
Isothermal
Start T: 200 C
Source: Shlyakhov, A.F., Gas chromatography in organic geochemistry, Nedra, Moscow, 1984, 221.

<...>

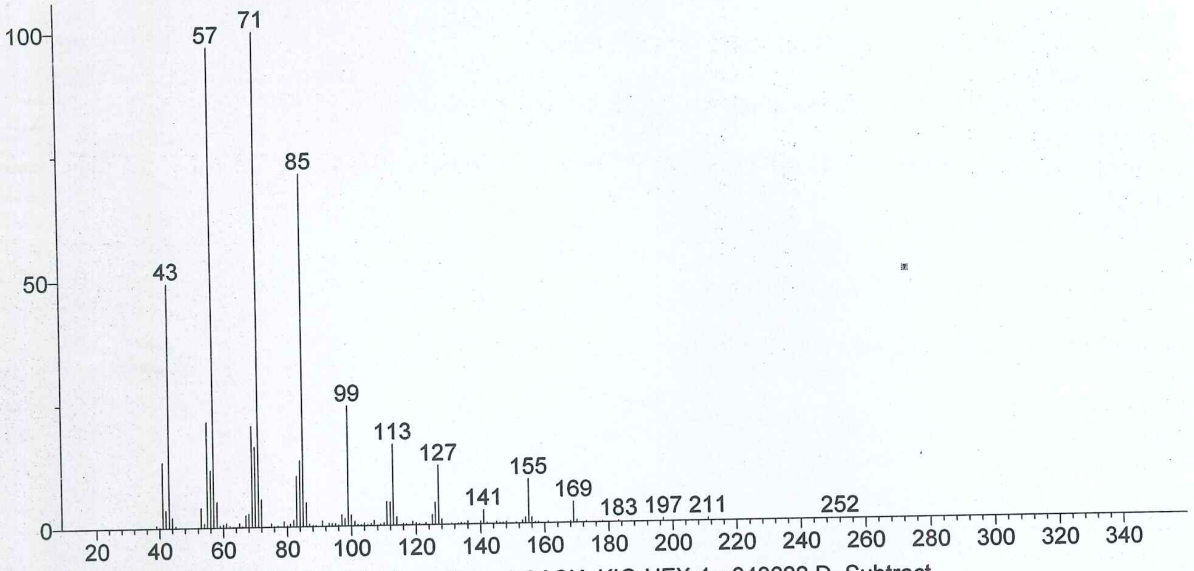

(Text File) +EI Scan (13.450-13.465 min, 5 Scans) AASIA-KIG-HEX-1a-040622.D Subtract

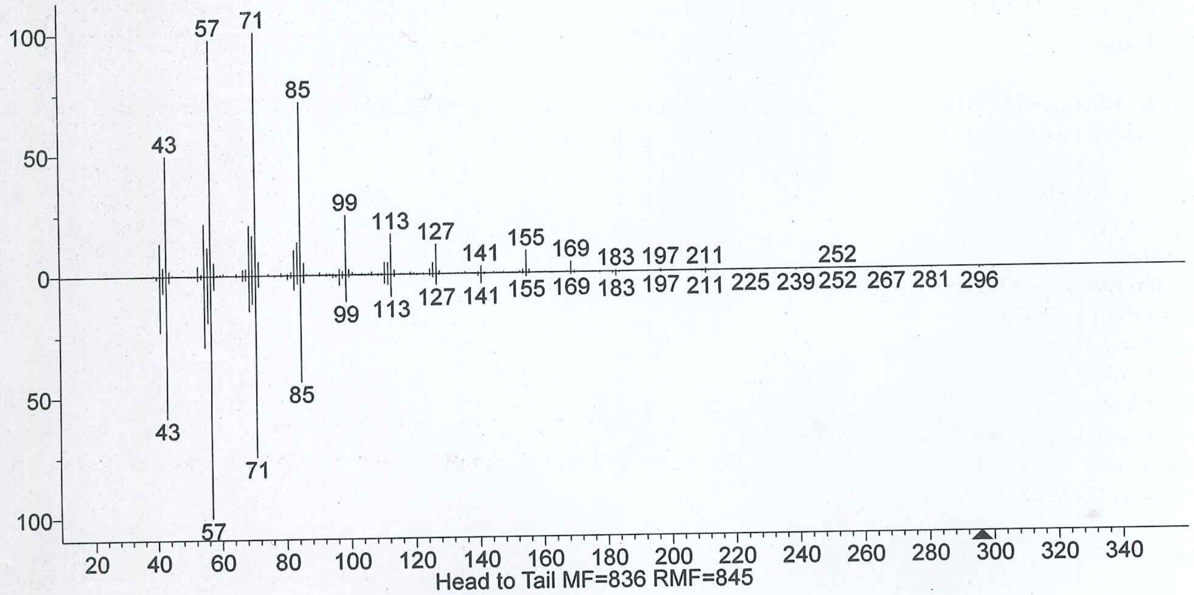

Head to Tail MF=836 RMF=845

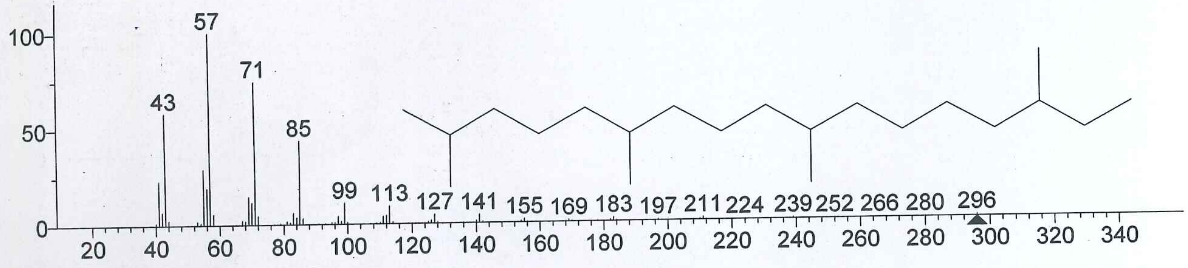

(mainlib) Heptadecane, 2,6,10,15-tetramethyl-

Name: Heptadecane, 2,6,10,15-tetramethyl-
Formula: $C_{21}H_{44}$
MW: 296 CAS#: 54833-48-6 NIST#: 14103 ID#: 22779 DB: mainlib
Other DBs: None
Contributor: W. VAN HOEVEN, UNIV. OF CALIFORNIA, BERKELEY, USA
10 largest peaks:
57 999 | 71 745 | 43 582 | 85 436 | 55 291 | 41 228 | 56 191 | 69 145 | 70 114 | 99 109 |
Synonyms:
1.2,6,10,15-Tetramethylheptadecane #

Estimated non-polar retention index (n-alkane scale):
Value: 1852 iu
Confidence interval (Hydrocarbons): 39(50%) 167(95%) iu

Retention index.
1. Value: 1914 iu
Column Type: Capillary
Column Class: Standard non-polar
Active Phase: Cross-Linked
Methylsilicone
Data Type: Linear RI
Program Type: Ramp
Start T: 40 C
End T: 300 C
Heat Rate: 5
K/min
Source: Khorasheh, F.; Gray, M.R.; Selucky, M.L., Correlation for Kováts retention index of C9-C26 monoalkyl and polymethyl alkanes and alkenes, J. Chromatogr., 481, 1989, 1-16.

2. Value: 1893 iu
Column Type:
Capillary
Column Class: Semi-standard non-polar
Active Phase: Apiezon L
Data Type: Kovats RI
Program
Type: Isothermal
Source: Shlyakhov, A.F., Gas chromatography in organic geochemistry, Nedra, Moscow, 1984, 221.

<...>

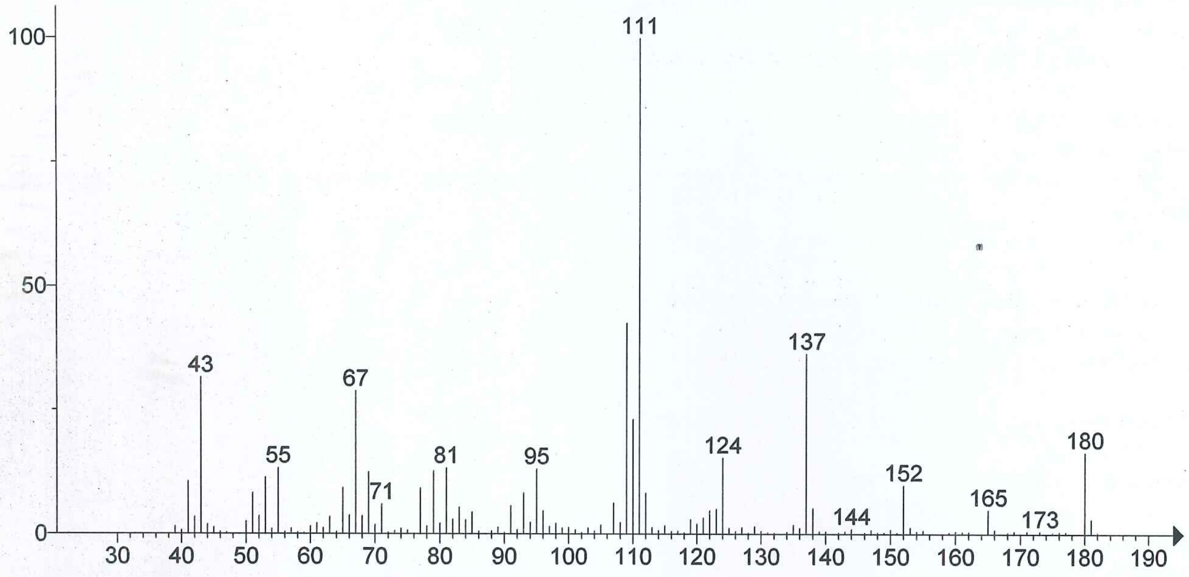

(Text File) +EI Scan (13.982 min) AASIA-KIG-HEX-1a-040622.D Subtract

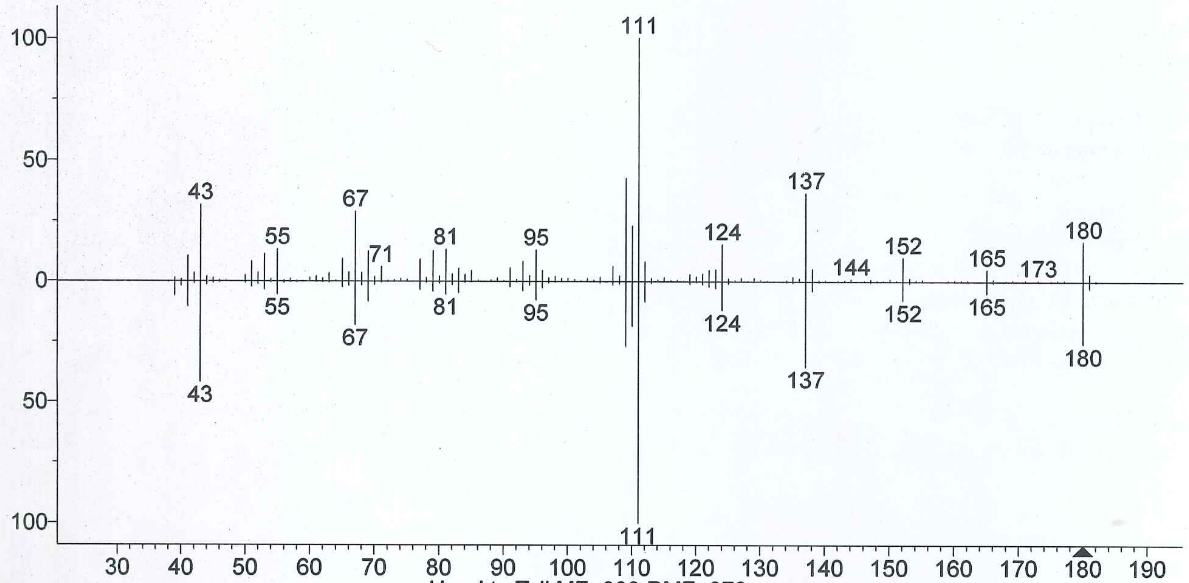

Head to Tail MF=839 RMF=872

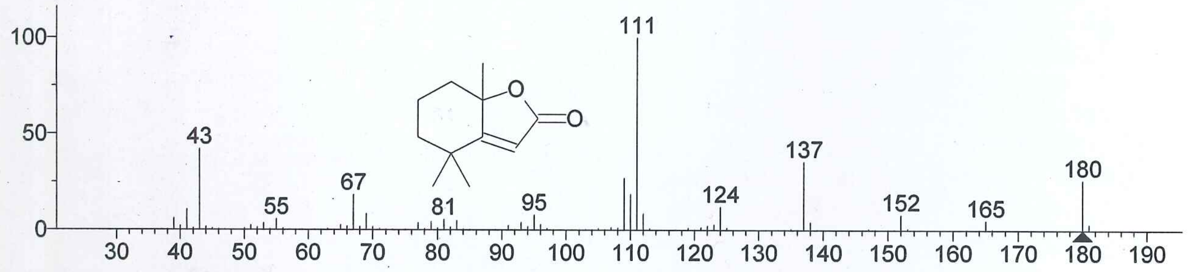

(mainlib) 2(4H)-Benzofuranone, 5,6,7,7a-tetrahydro-4,4,7a-trimethyl-, (R)-

Name: 2(4H)-Benzofuranone, 5,6,7,7a-tetrahydro-4,4,7a-trimethyl-, (R)-
Formula: $C_{11}H_{16}O_2$
MW: 180 CAS#: 17092-92-1 NIST#: 108912 ID#: 74534 DB: mainlib
Other DBs: NIH
Contributor: Philip Morris R&D
10 largest peaks:
111 999 | 43 418 | 137 353 | 109 268 | 180 259 | 110 184 | 67 181 | 124 119 | 41 106 | 112 85 |
Synonyms:
1.2(4H)-Benzofuranone, 5,6,7,7a-tetrahydro-4,4,7a-trimethyl-
2.Actinidiolide, dihydro-
3.2(4H)-Benzofuranone, 5,6,7,7a-tetrahydro-4,4,7a-trimethyl-, (S)-
4.Dihydroactinidiolide
5.4,4,7a-Trimethyl-5,6,7,7a-tetrahydro-1-benzofuran-2(4H)-one #

Estimated non-polar retention index (n-alkane scale):
Value: 1426 iu
Confidence interval (Esters): 47(50%) 201(95%) iu

Retention index.
1. Value: 1532 iu
Column Type: Capillary
Column Class: Standard non-polar
Active Phase: HP-1
Column
Length: 50 m
Carrier Gas: He
Column Diameter: 0.32 mm
Phase Thickness: 1.05 um
Data Type: Linear
RI
Program Type: Complex
Description: 20C(0.5min) =>60C=>4C/min=>250C
Source: Sing, A.S.C.; Smadja, J.;
Brevard, H.; Maignial, L.; Chaintreau, A.; Marion, J.-P., Volatile constituents of faham (Jumellea fragrans (Thou.) Schltr.), J. Agric. Food Chem., 40, 1992, 642-646.

2. Value: 1471 iu
Column Type: Capillary
Column Class:
Standard non-polar
Active Phase: SPB-1
Column Length: 30 m
Carrier Gas: He
Column Diameter: 0.32
mm
Phase Thickness: 0.25 um
Data Type: Kovats RI
Program Type: Complex
Description: 40C(3min)
=>2C/min =>100C=>4C/min =>220C (7min)
Source: Borse, B.B.; Jagan Mohan Rao, L.; Nagalakshmi, S.;
Krishnamurthy, N., Fingerprint of black teas from India: identification of the regio-specific characteristics, Food Chem., 79, 2002, 419-424.

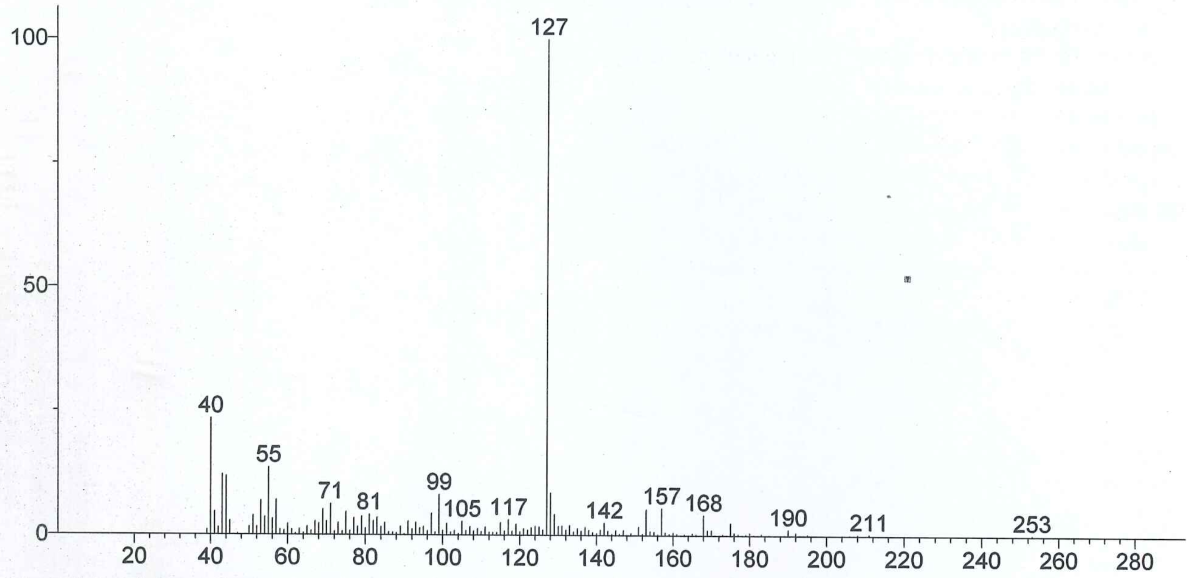

(Text File) +EI Scan (14.370-14.395 min, 8 Scans) AASIA-KIG-HEX-1a-040622.D

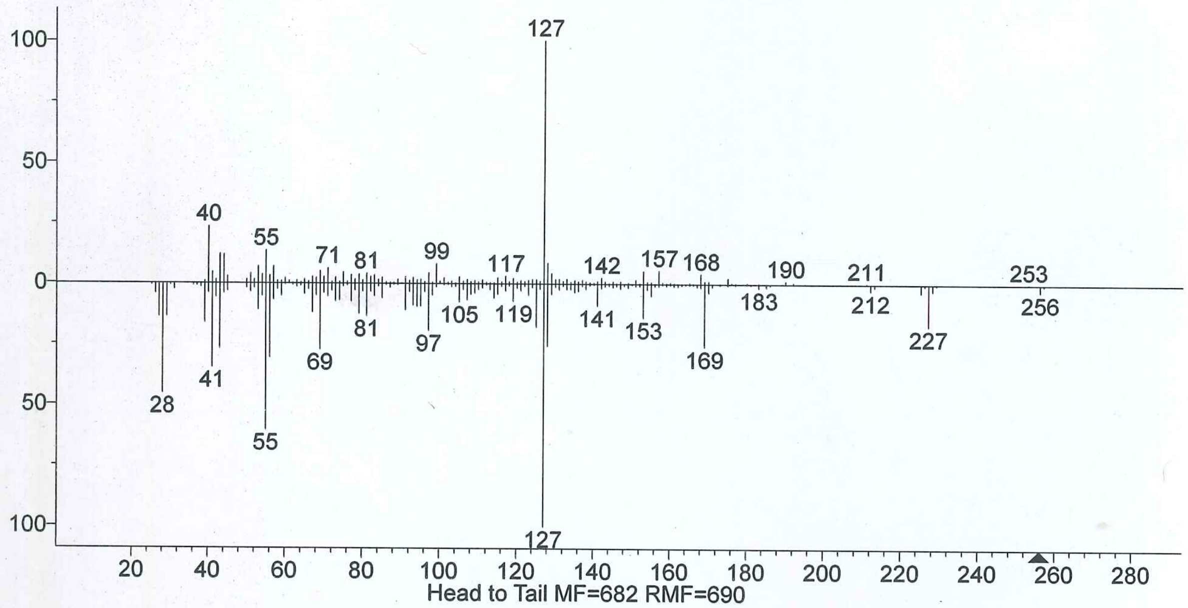

Head to Tail MF=682 RMF=690

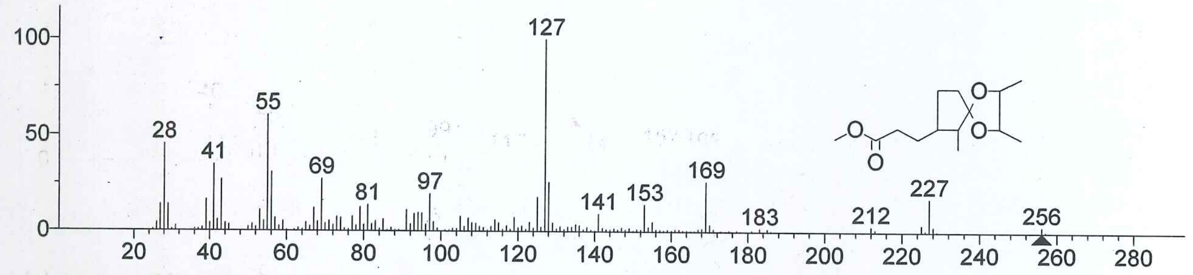

(mainlib) Propanoic acid, 3-(2,3,6-trimethyl-1,4-dioxaspiro[4.4]non-7-yl)-, methyl ester

Name: Propanoic acid, 3-(2,3,6-trimethyl-1,4-dioxaspiro[4.4]non-7-yl)-, methyl ester
Formula: $C_{14}H_{24}O_4$
MW: 256 NIST#: 197888 ID#: 89727 DB: mainlib
Contributor: Chemical Concepts
10 largest peaks:
127 999 | 55 603 | 28 452 | 41 346 | 56 306 | 69 272 | 43 268 | 169 260 | 128 257 | 97 194 |
Synonyms:
no synonyms.

Estimated non-polar retention index (n-alkane scale):
Value: 1682 iu
Confidence interval (Diverse functional groups): 89(50%) 382(95%) iu

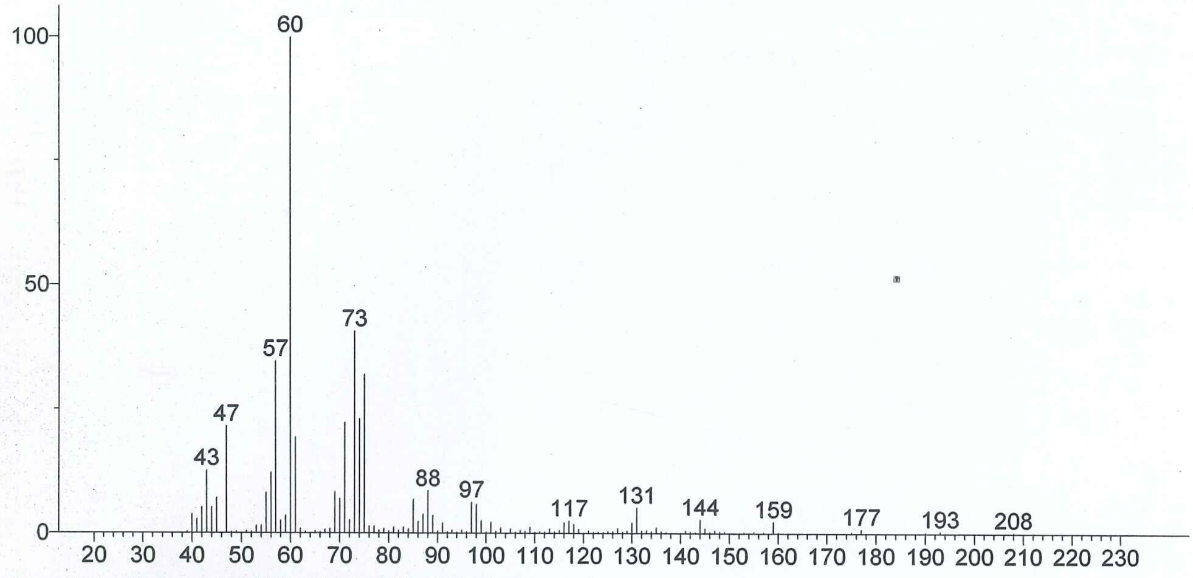

(Text File) +EI Scan (15.059 min) AASIA-KIG-HEX-1a-040622.D

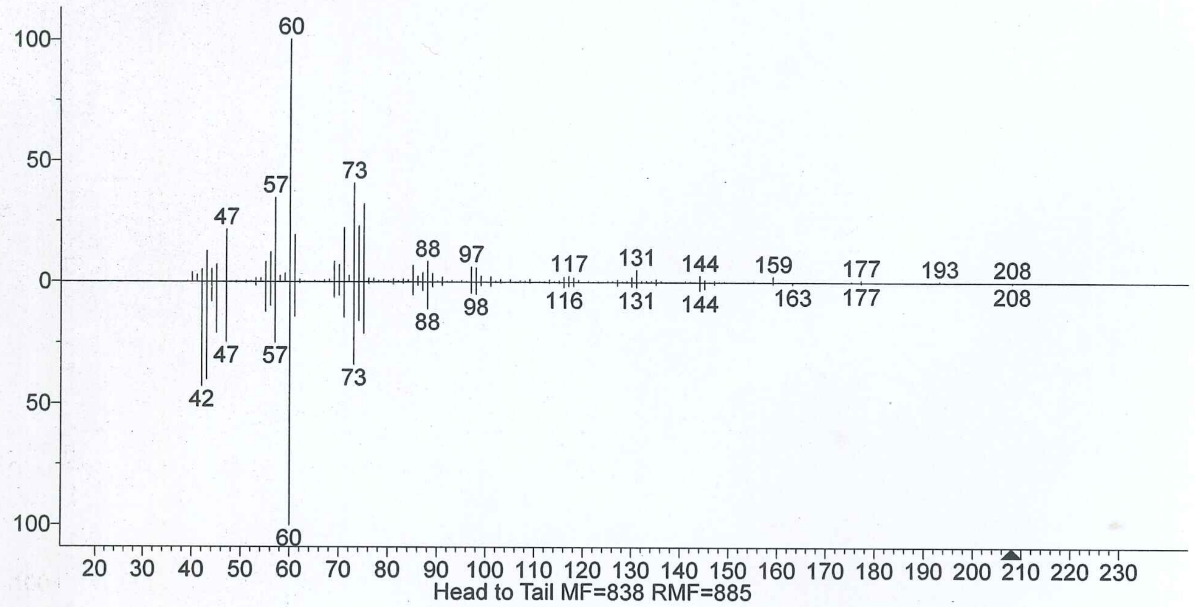

Head to Tail MF=838 RMF=885

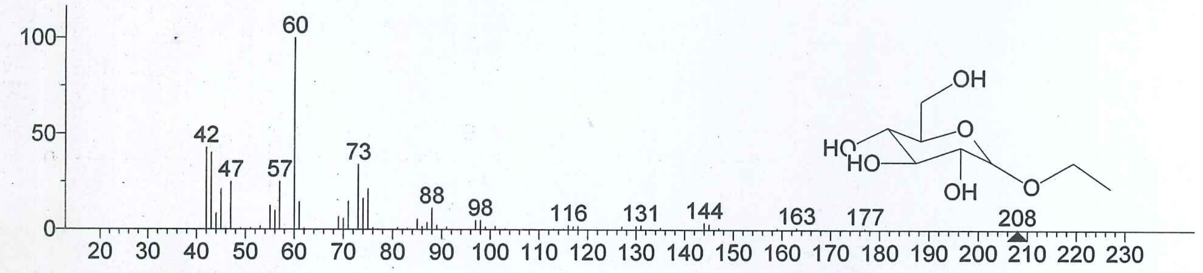

(mainlib) Ethyl α-d-glucopyranoside

Name: Ethyl α-d-glucopyranoside
Formula: $C_8H_{16}O_6$
MW: 208 NIST#: 127294 ID#: 27054 DB: mainlib
Contributor: LAC, NIDDK, NIH, Bethesda, MD 20892
10 largest peaks:
60 999 | 42 427 | 43 400 | 73 339 | 57 249 | 47 247 | 75 211 | 45 210 | 74 160 | 71 146 |
Synonyms:
no synonyms.

Estimated non-polar retention index (n-alkane scale):
Value: 1813 iu
Confidence interval (Diverse functional groups): 89(50%) 382(95%) iu

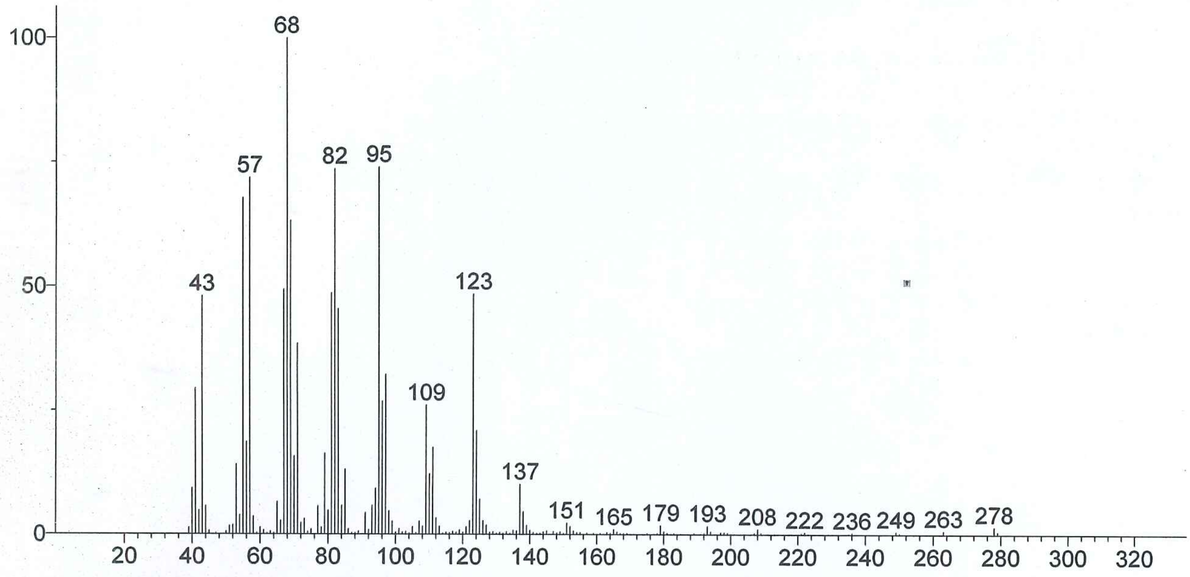

(Text File) +EI Scan (17.307 min) AASIA-KIG-HEX-1a-040622.D

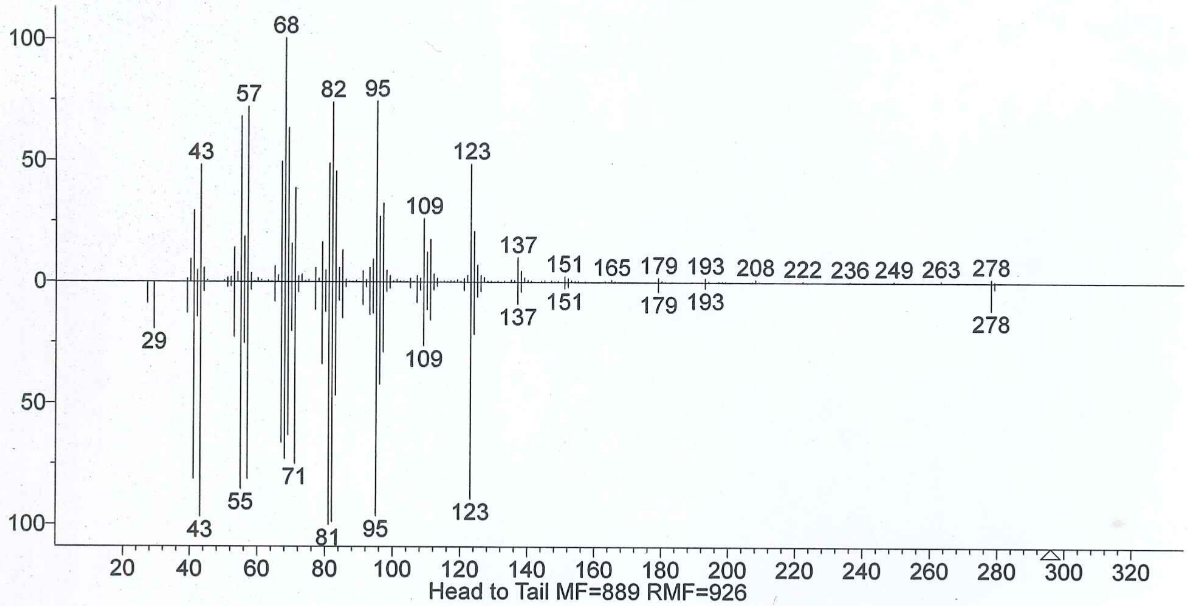

Head to Tail MF=889 RMF=926

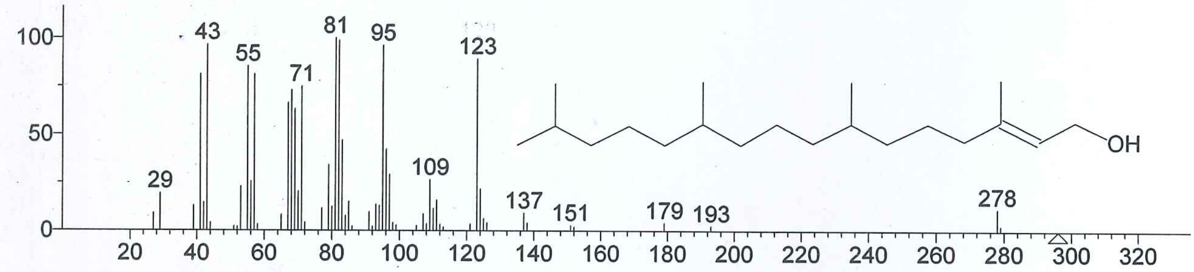

(mainlib) 3,7,11,15-Tetramethyl-2-hexadecen-1-ol

Name: 3,7,11,15-Tetramethyl-2-hexadecen-1-ol
Formula: $C_{20}H_{40}O$
MW: 296 CAS#: 102608-53-7 NIST#: 114703 ID#: 43206 DB: mainlib
Other DBs: IRDB
Contributor: NIST Mass Spectrometry Data Center, 1990.
10 largest peaks:
81 999 | 82 986 | 43 965 | 95 962 | 123 892 | 55 852 | 41 811 | 57 811 | 71 748 | 68 728 |
Synonyms:
1.(2E)-3,7,11,15-Tetramethyl-2-hexadecen-1-ol #

Estimated non-polar retention index (n-alkane scale):
Value: 2045 iu
Confidence interval (Alcohols): 41(50%) 176(95%) iu

Retention index.
1. Value: 2119.33 iu
Column Type: Capillary
Column Class: Semi-standard non-polar
Active Phase: SE
-54
Column Length: 25 m
Column Diameter: 0.31 mm
Data Type: Linear RI
Program Type: Ramp
Start T: 35
C
End T: 230 C
Heat Rate: 4 K/min
Start Time: 3 min
End Time: 10 min
Source: Yin, W.; Xiu, Z.; Aijin, H.,
Analysis of the volatile components in trogopterorum feces by capillary gas chromatography and gas chromatography/mass spectrometry, Fenxi Huaxue, 29(2), 2001, 195-198.

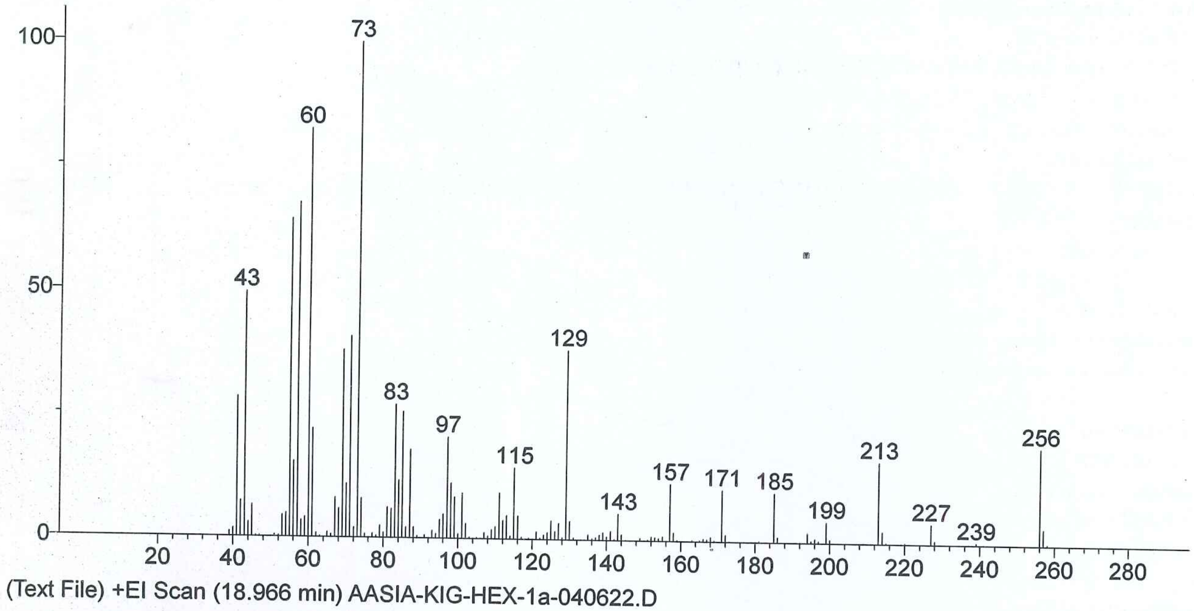

(Text File) +EI Scan (18.966 min) AASIA-KIG-HEX-1a-040622.D

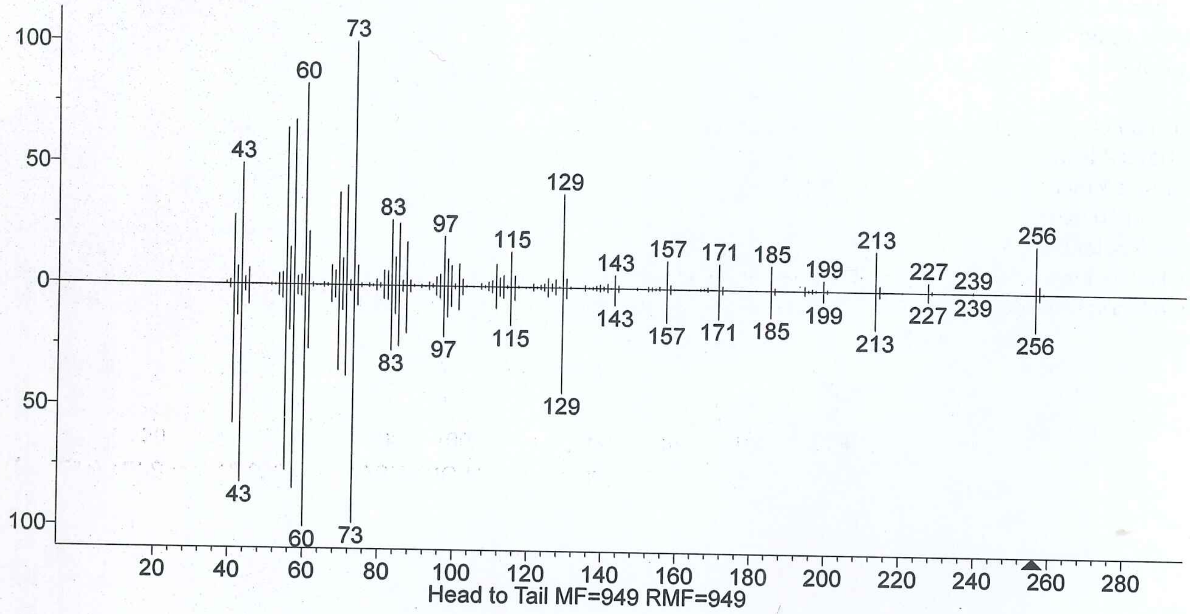

Head to Tail MF=949 RMF=949

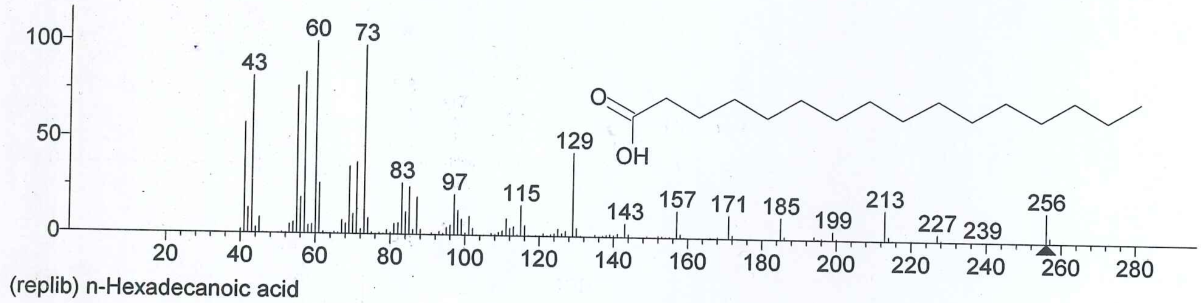

(replib) n-Hexadecanoic acid

Name: n-Hexadecanoic acid
Formula: $C_{16}H_{32}O_2$
MW: 256 CAS#: 57-10-3 NIST#: 335494 ID#: 6723 DB: replib
Other DBs: Fine, TSCA, RTECS, EPA, HODOC, NIH, EINECS, IRDB
Contributor: Drug Lab
10 largest peaks:
60 999 | 73 980 | 57 840 | 43 817 | 55 767 | 41 574 | 129 435 | 71 373 | 69 351 | 83 267 |
Synonyms:
1.Hexadecanoic acid
2.n-Hexadecoic acid
3.Palmitic acid
4.Pentadecanecarboxylic acid
5.1-Pentadecanecarboxylic acid
6.Cetylic acid
7.Emersol 140
8.Emersol 143
9.Hexadecylic acid
10.Hydrofol
11.Hystrene 8016
12.Hystrene 9016
13.Industrene 4516
14.Prifrac 2960
15.Glycon P-45
16.Prifac 2960
17.Univol U332

Estimated non-polar retention index (n-alkane scale):
Value: 1968 iu
Confidence interval (Carboxylic acids): 51(50%) 220(95%) iu

Retention index.
1. Value: 1942 iu
Column Type: Capillary
Column Class: Standard non-polar
Active Phase: RTX-1
Column
Length: 60 m
Carrier Gas: He
Column Diameter: 0.22 mm
Phase Thickness: 0.25 um
Data Type: Linear
RI
Program Type: Ramp
Start T: 60 C
End T: 230 C
Heat Rate: 2 K/min
End Time: 35 min
Source: Paolini, J.;
Muselli, A.; Bernardini, A.-F.; Bighelli, A.; Casanova, J.; Costa, J., Thymol derivatives from essential oil of Doronicum corsicum L., Flavour Fragr. J., 22, 2007, 479-487.

2. Value: 1972 iu
Column Type: Capillary
Column

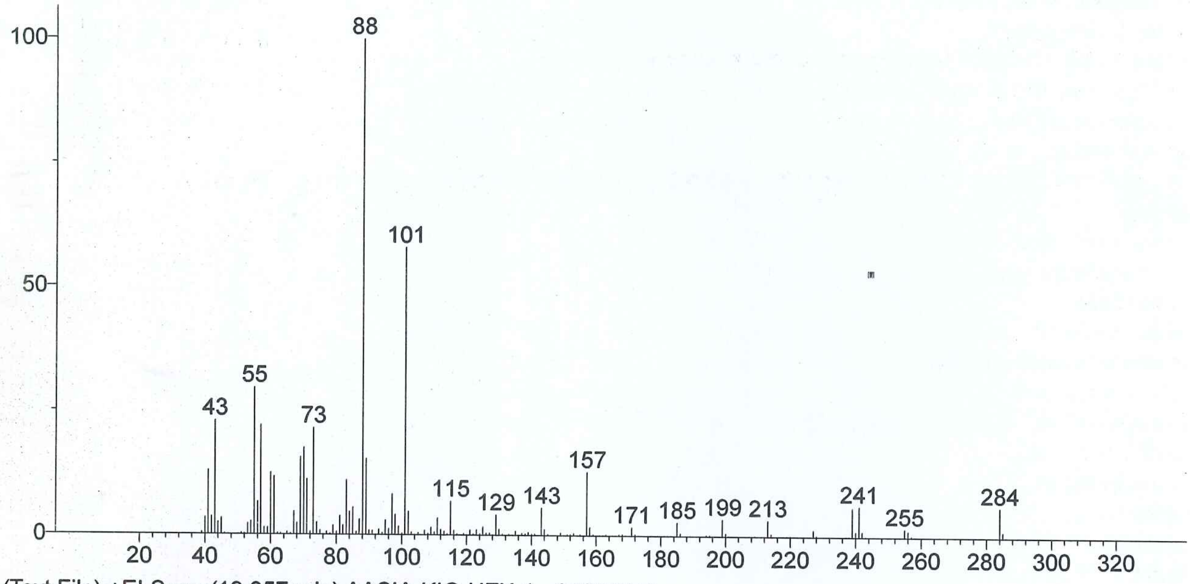

(Text File) +EI Scan (19.357 min) AASIA-KIG-HEX-1a-040622.D

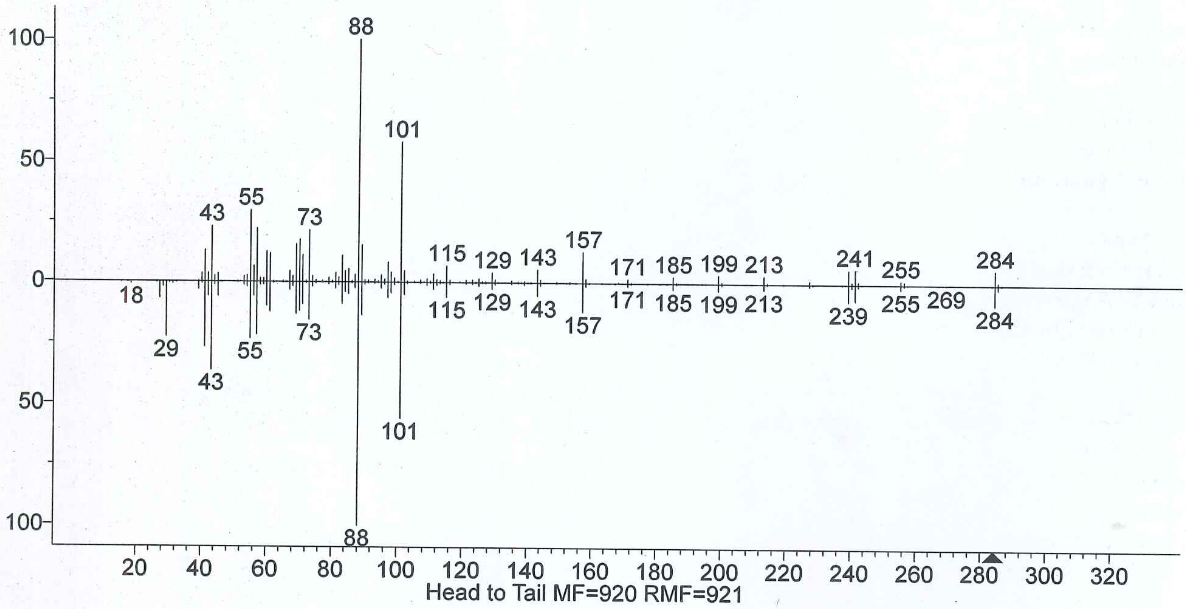

Head to Tail MF=920 RMF=921

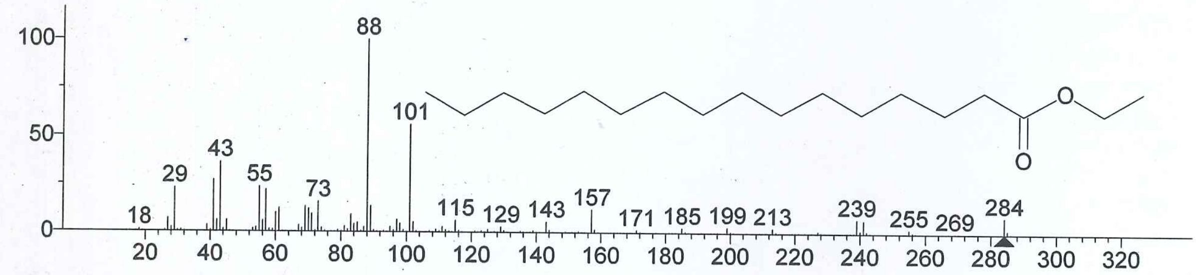

(mainlib) Hexadecanoic acid, ethyl ester

Name: Hexadecanoic acid, ethyl ester
Formula: $C_{18}H_{36}O_2$
MW: 284 CAS#: 628-97-7 NIST#: 233204 ID#: 49485 DB: mainlib
Other DBs: Fine, TSCA, EPA, HODOC, NIH, EINECS, IRDB
Contributor: Japan AIST/NIMC Database- Spectrum MS-NW-5396
10 largest peaks:
88 999 | 101 559 | 43 362 | 41 268 | 55 233 | 29 227 | 57 217 | 73 156 | 89 136 | 69 132 |
Synonyms:
1.Palmitic acid, ethyl ester
2.Ethyl hexadecanoate
3.Ethyl palmitate

Estimated non-polar retention index (n-alkane scale):
Value: 1978 iu
Confidence interval (Esters): 47(50%) 201(95%) iu

Retention index.
1. Value: 1968 iu
Column Type: Capillary
Column Class: Standard non-polar
Active Phase: SPB-1
Column
Length: 30 m
Carrier Gas: He
Column Diameter: 0.25 mm
Phase Thickness: 0.25 um
Data Type: Linear
RI
Program Type: Ramp
Start T: 50 C
End T: 250 C
Heat Rate: 5 K/min
Start Time: 3 min
End Time: 15
min
Source: Blagojevic, P.; Radulovic, N.; Palic, R.; Stojanovic, G., Chemical composition of the essential oils of Serbian wild-growing Srtemisia absinthium and Artemisia vulgaris, J. Agric. Food Chem., 54, 2006, 4780-4789.

2.
Value: 1966 iu
Column Type: Capillary
Column Class: Standard non-polar
Active Phase: HP-1
Column Length:
50 m
Carrier Gas: He
Column Diameter: 0.2 mm
Phase Thickness: 0.33 um
Data Type: Linear RI
Program
Type: Ramp
Start T: 60 C
End T: 250 C
Heat Rate: 2 K/min

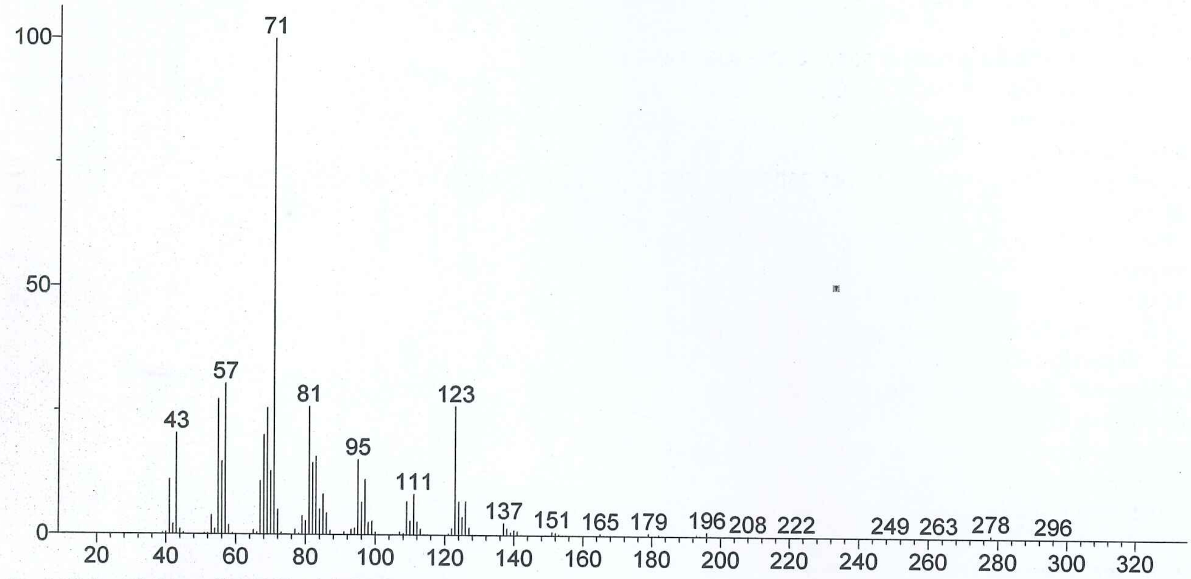

(Text File) +EI Scan (21.745 min) AASIA-KIG-HEX-1a-040622.D

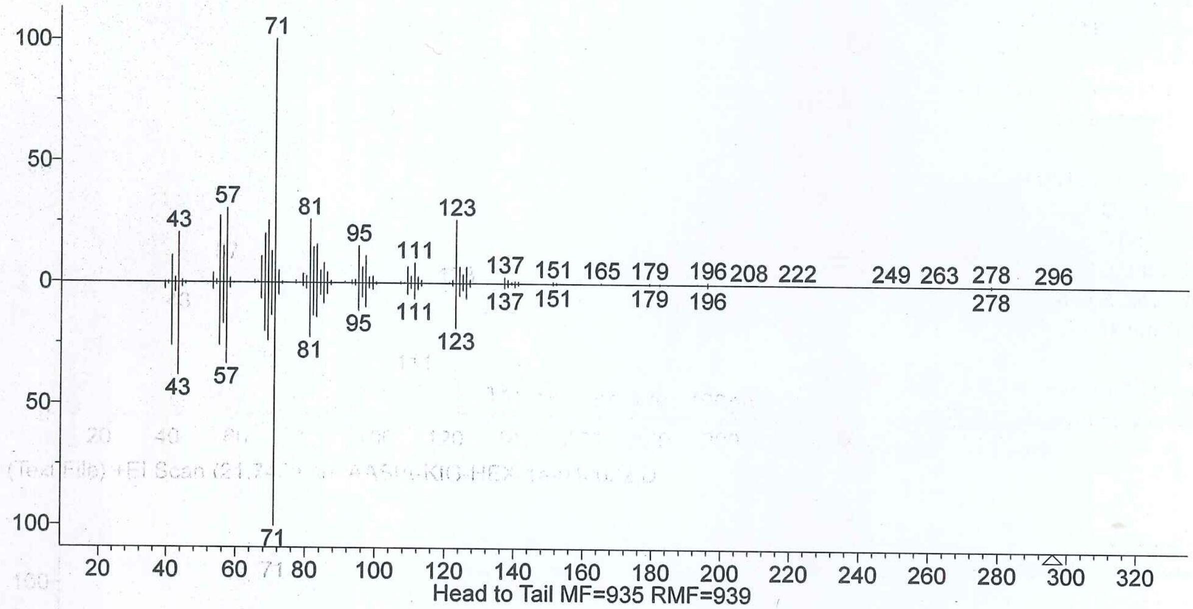

Head to Tail MF=935 RMF=939

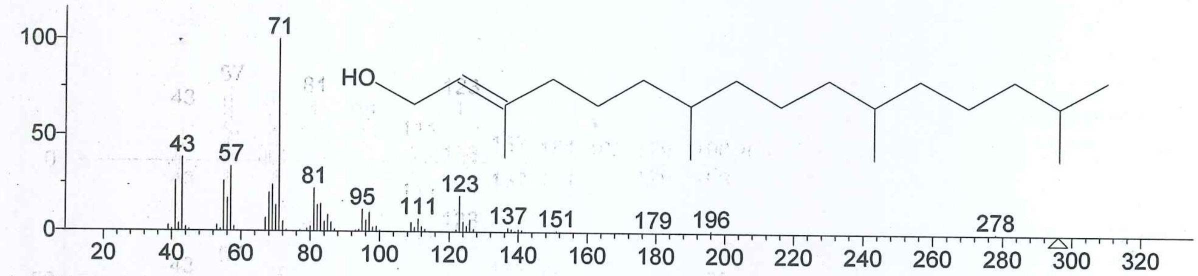

(replib) Phytol

Name: Phytol
Formula: $C_{20}H_{40}O$
MW: 296 CAS#: 150-86-7 NIST#: 108727 ID#: 8051 DB: replib
Other DBs: Fine, TSCA, RTECS, HODOC, EINECS
Contributor: Philip Morris R&D
10 largest peaks:
71 999 | 43 381 | 57 334 | 41 260 | 55 259 | 69 239 | 81 223 | 68 199 | 123 184 | 56 169 |
Synonyms:
1.2-Hexadecen-1-ol, 3,7,11,15-tetramethyl-, [R-[R*,R*-(E)]]-
2.trans-Phytol
3.3,7,11,15-Tetramethyl-2-hexadecen-1-ol
4.(2E)-3,7,11,15-Tetramethyl-2-hexadecen-1-ol #

Estimated non-polar retention index (n-alkane scale):
Value: 2045 iu
Confidence interval (Alcohols): 41(50%) 176(95%) iu

Retention index.
1. Value: 2104 iu
Column Type: Capillary
Column Class: Standard non-polar
Active Phase: SPB-1
Column
Length: 30 m
Carrier Gas: He
Column Diameter: 0.25 mm
Phase Thickness: 0.25 um
Data Type: Linear
RI
Program Type: Ramp
Start T: 50 C
End T: 250 C
Heat Rate: 5 K/min
Start Time: 3 min
End Time: 15
min
Source: Radulovic, N.; Lazarevic, J.; Stojanovic, G.; Palic, R., Chemotaxonomically significant 2-ethyl substituted fatty acids from Stachys milanii Petrovic (Lamiaceae), Biochem. Syst. Ecol., 34, 2006, 341-344.

2.
Value: 2099 iu
Column Type: Capillary
Column Class: Standard non-polar
Active Phase: RTX-1
Column Length:
60 m
Carrier Gas: He
Column Diameter: 0.22 mm
Phase Thickness: 0.25 um
Data Type: Linear RI
Program
Type: Ramp
Start T: 60 C
End T: 230 C

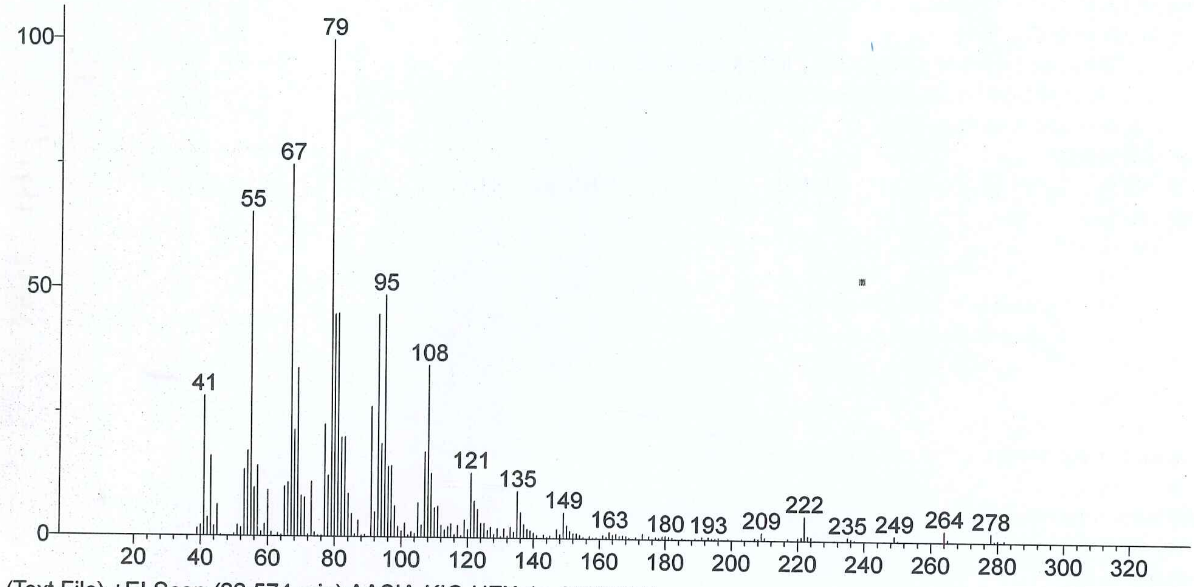

(Text File) +EI Scan (22.574 min) AASIA-KIG-HEX-1a-040622.D

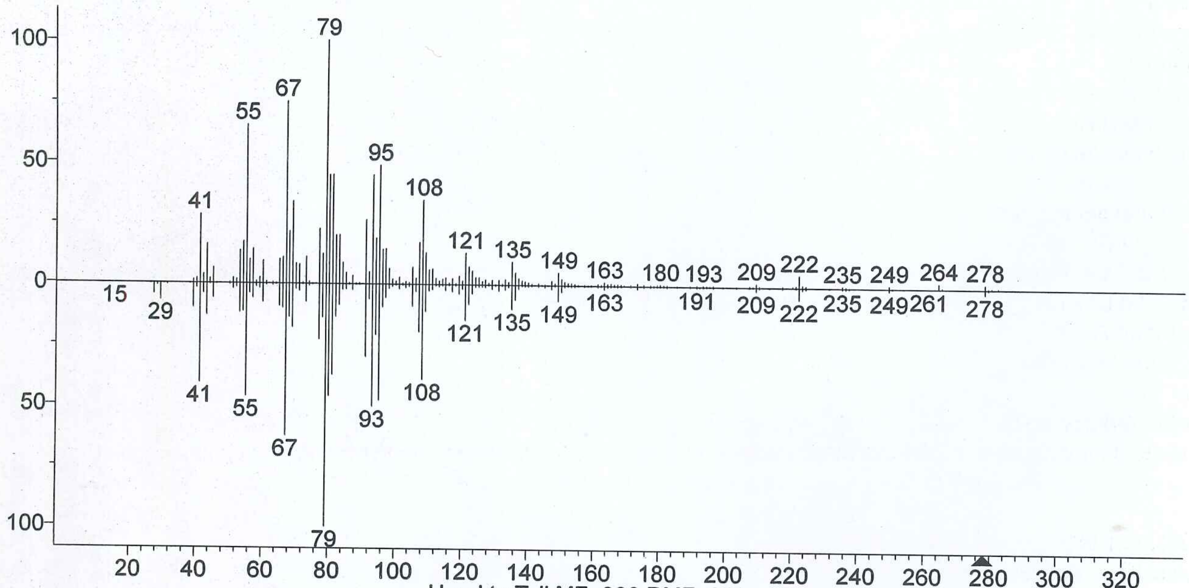

Head to Tail MF=906 RMF=911

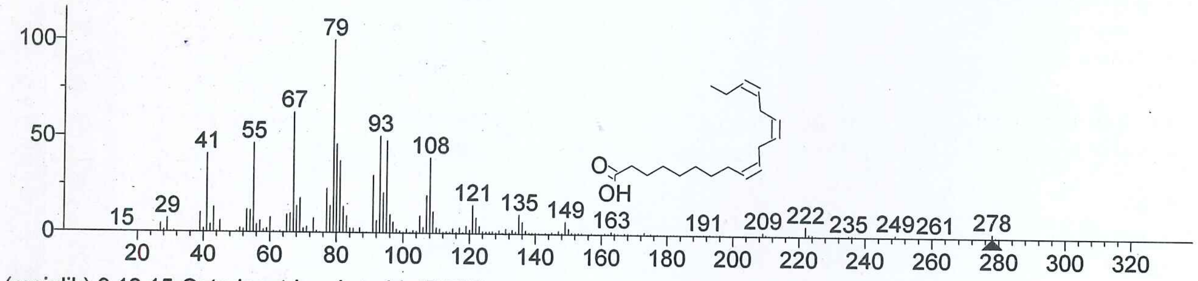

(mainlib) 9,12,15-Octadecatrienoic acid, (Z,Z,Z)-

Name: 9,12,15-Octadecatrienoic acid, (Z,Z,Z)-
Formula: $C_{18}H_{30}O_2$
MW: 278 CAS#: 463-40-1 NIST#: 333201 ID#: 41695 DB: mainlib
Other DBs: Fine, TSCA, HODOC, NIH, EINECS
Contributor: NIST Mass Spectrometry Data Center
10 largest peaks:
79 999 | 67 624 | 93 503 | 95 478 | 55 463 | 80 460 | 41 408 | 108 391 | 81 373 | 91 299 |
Synonyms:
1.Linolenic acid
2.α-Linolenic acid
3.All-cis-9,12,15-Octadecatrienoic acid
4.cis,cis,cis-9,12,15-Octadecatrienoic acid
5.(Z,Z,Z)-9,12,15-Octadecatrienoic acid
6.Industrene 120

Estimated non-polar retention index (n-alkane scale):
Value: 2191 iu
Confidence interval (Carboxylic acids): 51(50%) 220(95%) iu

Retention index.
1. Value: 2102 iu
Column Type: Capillary
Column Class: Standard non-polar
Active Phase: HP-101
Column
Length: 25 m
Carrier Gas: He
Column Diameter: 0.2 mm
Phase Thickness: 0.2 um
Data Type: Normal alkane
RI
Program Type: Ramp
Start T: 70 C
End T: 200 C
Heat Rate: 3 K/min
Start Time: 2 min
Source: Jerkovic, I.;
Mastelic, J.; Marijanovic, Z., Bound volatile compounds and essential oil from the fruit of Maclura pomifera (Raf.) Schneid. (osage orange), Flavour Fragr. J., 22, 2007, 84-88.

2. Value: 2178 iu
Column Type: Other
Column
Class: Standard non-polar
Active Phase: Methyl Silicone
Data Type: Normal alkane RI
Program Type:
Ramp
Source: Ardrey, R.E.; Moffat, A.C., Gas-liquid chromatographic retention indices of 1318 substances of toxicological interest on SE-30 or OV-1 stationary phase, J. Chromatogr., 220, 1981, 195-252.

<...>

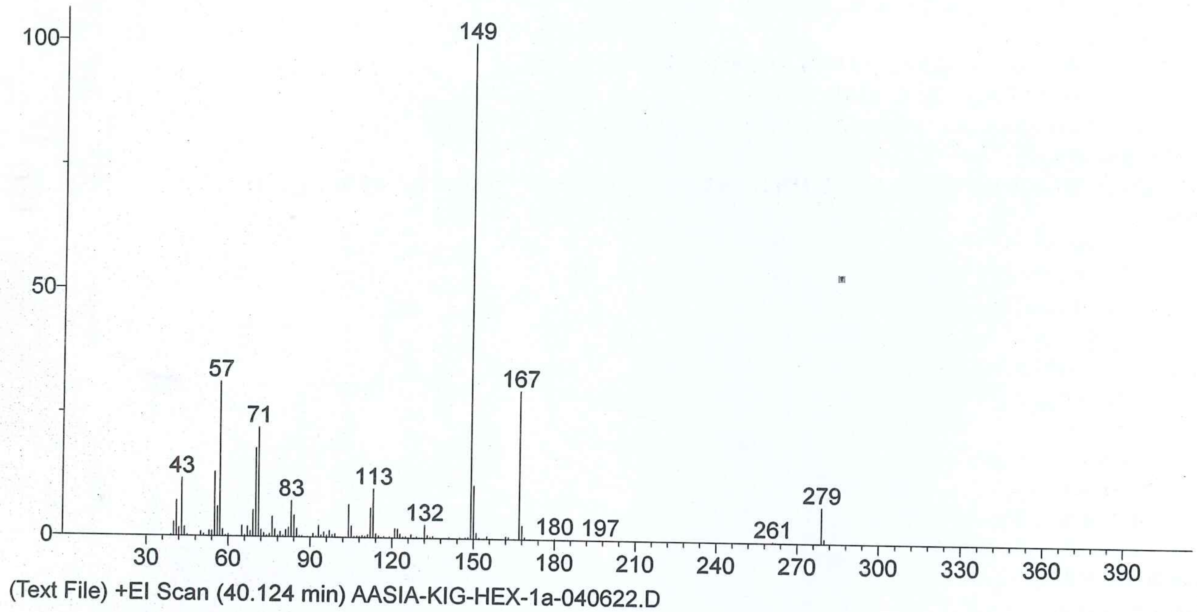

(Text File) +EI Scan (40.124 min) AASIA-KIG-HEX-1a-040622.D

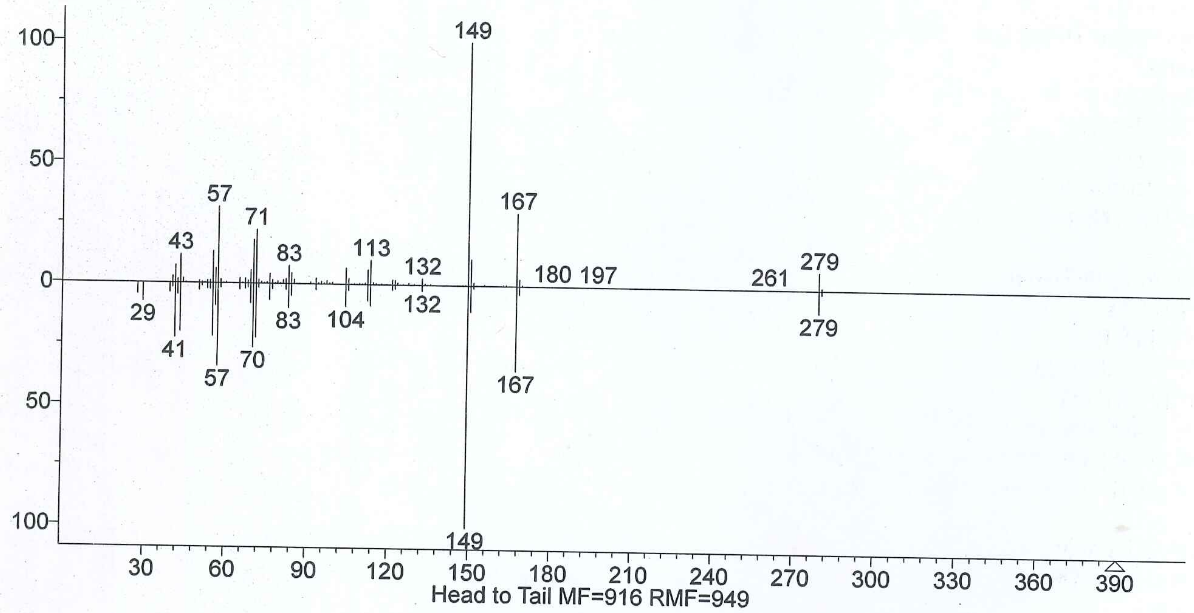

Head to Tail MF=916 RMF=949

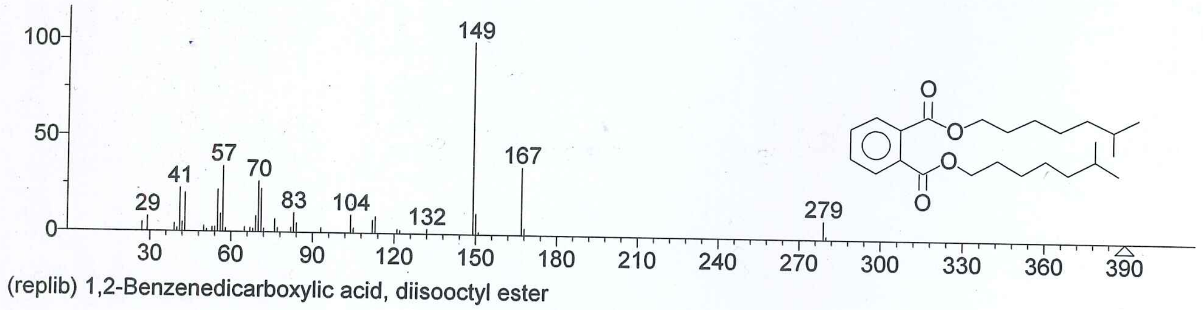

(replib) 1,2-Benzenedicarboxylic acid, diisooctyl ester

Name: 1,2-Benzenedicarboxylic acid, diisooctyl ester
Formula: $C_{24}H_{38}O_4$
MW: 390 CAS#: 27554-26-3 NIST#: 113206 ID#: 20061 DB: replib
Other DBs: Fine, TSCA, RTECS, EINECS, IRDB
Contributor: NIST Mass Spectrometry Data Center, 1990.
10 largest peaks:
149 999 | 167 350 | 57 341 | 70 264 | 41 225 | 71 224 | 55 218 | 43 200 | 150 107 | 83 100 |
Synonyms:
1.Diisooctyl phthalate
2.Hexaplas M/O
3.Isooctyl phthalate
4.Corflex 880
5.DIOP
6.Flexol plasticizer diop
7.Morflex 100
8.Palatinol D10
9.Phthalic acid, bis(6-methylheptyl) ester
10.Phthalic acid, diisooctyl ester
11.Witcizer 313
12.Bis(6-methylheptyl) phthalate #

Estimated non-polar retention index (n-alkane scale):
Value: 2704 iu
Confidence interval (Esters): 47(50%) 201(95%) iu

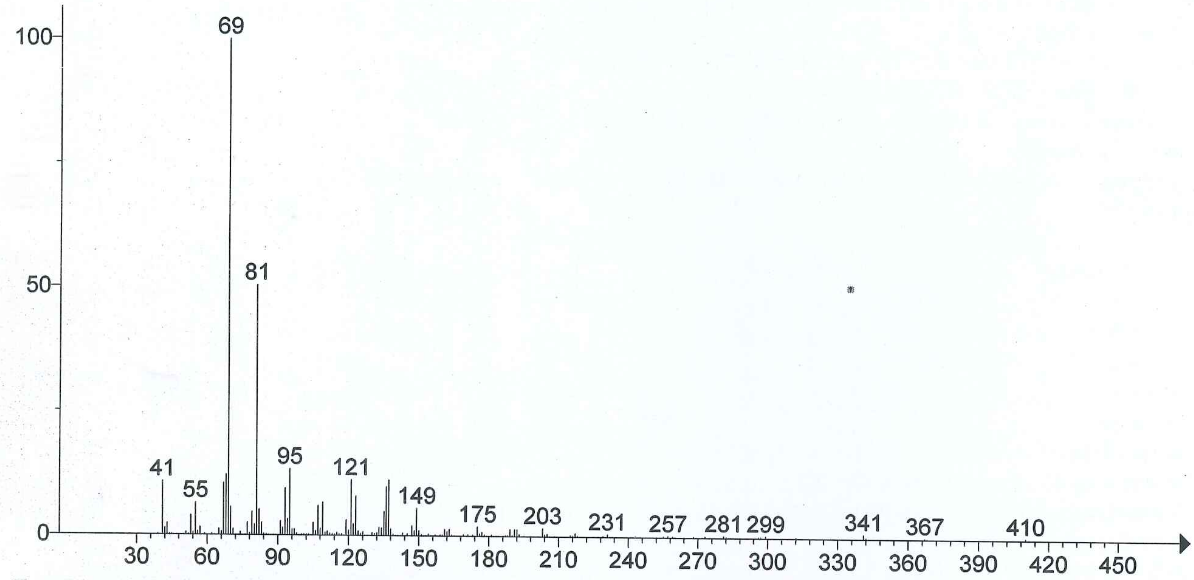

(Text File) +EI Scan (44.573-44.598 min, 8 Scans) AASIA-KIG-HEX-1a-040622.D Subtract

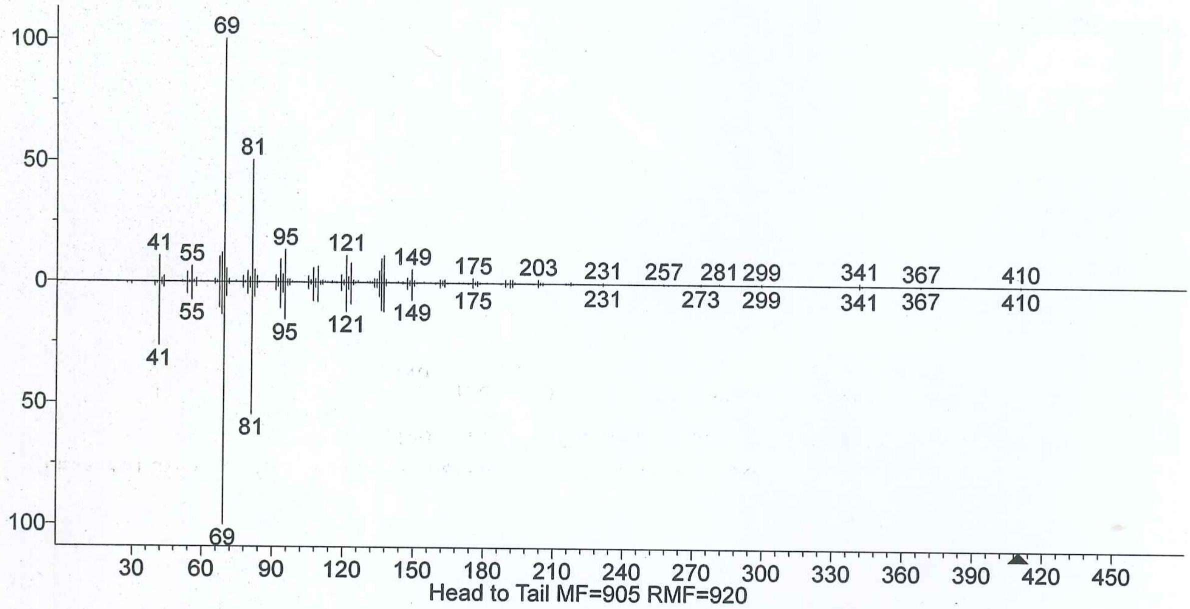

Head to Tail MF=905 RMF=920

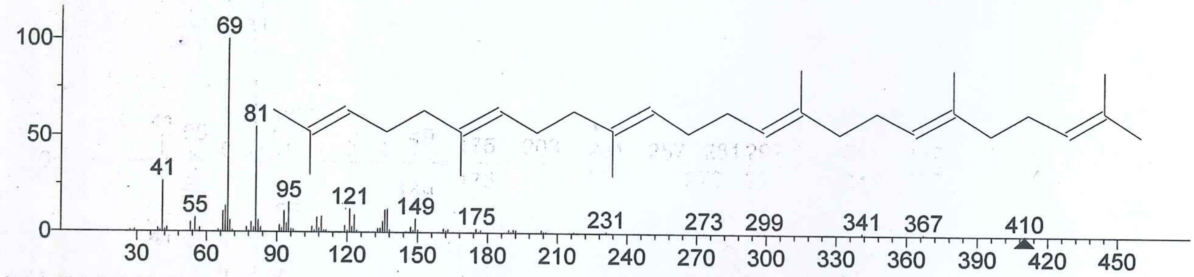

(mainlib) 2,6,10,14,18,22-Tetracosahexaene, 2,6,10,15,19,23-hexamethyl-, (all-E)-

Name: 2,6,10,14,18,22-Tetracosahexaene, 2,6,10,15,19,23-hexamethyl-, (all-E)-
Formula: $C_{30}H_{50}$
MW: 410 CAS#: 111-02-4 NIST#: 290792 ID#: 30950 DB: mainlib
Other DBs: Fine, TSCA, RTECS, HODOC, EINECS
Contributor: NIST Mass Spectrometry Data Center, 1998.
10 largest peaks:
69 999 | 81 545 | 41 264 | 95 155 | 68 134 | 121 121 | 137 121 | 136 116 | 93 108 | 67 107 |
Synonyms:
1.All-trans-Squalene
2.trans-Squalene
3.Spinacen
4.Spinacene
5.Squalen
6.Squalene
7.Supraene
8.2,6,10,15,19,23-Hexamethyl-2,6,10,14,18,22-Tetracosahexaene
9.2,6,10,15,19,23-Hexamethyltetracosa-2,6,10,14,18,22-hexaene
10.(All-E)-2,6,10,15,19,23-hexamethyl-2,6,10,14,18,22-tetracosahexaene
11.trans-Spinacene
12.(6E,10E,14E,18E)-2,6,10,15,19,23-Hexamethyl-2,6,10,14,18,22-tetracosahexaene #

Estimated non-polar retention index (n-alkane scale):
Value: 2914 iu
Confidence interval (Hydrocarbons): 39(50%) 167(95%) iu

Retention index.
1. Value: 2847.1 iu
Column Type: Capillary
Column Class: Semi-standard non-polar
Active Phase: HP
-5MS
Column Length: 30 m
Carrier Gas: He
Column Diameter: 0.25 mm
Phase Thickness: 0.25 um
Data Type:
Linear RI
Program Type: Ramp
Start T: 80 C
End T: 300 C
Heat Rate: 4 K/min
Source: Zhao C.X.; Li, X.N.;
Liang Y.Z.; Fang H.Z.; Huang L.F.; Guo F.Q., Comparative analysis of chemical components of essential oils from different samples of Rhododendron with the help of chemometrics methods, Chemom. Intell. Lab. Syst., 82, 2006, 218-228.

2. Value: 2809.1 iu
Column Type: Capillary
Column Class: Semi-standard non-polar
Active Phase:
DB-5
Column Length: 30 m
Carrier Gas: He
Column Diameter: 0.32 mm

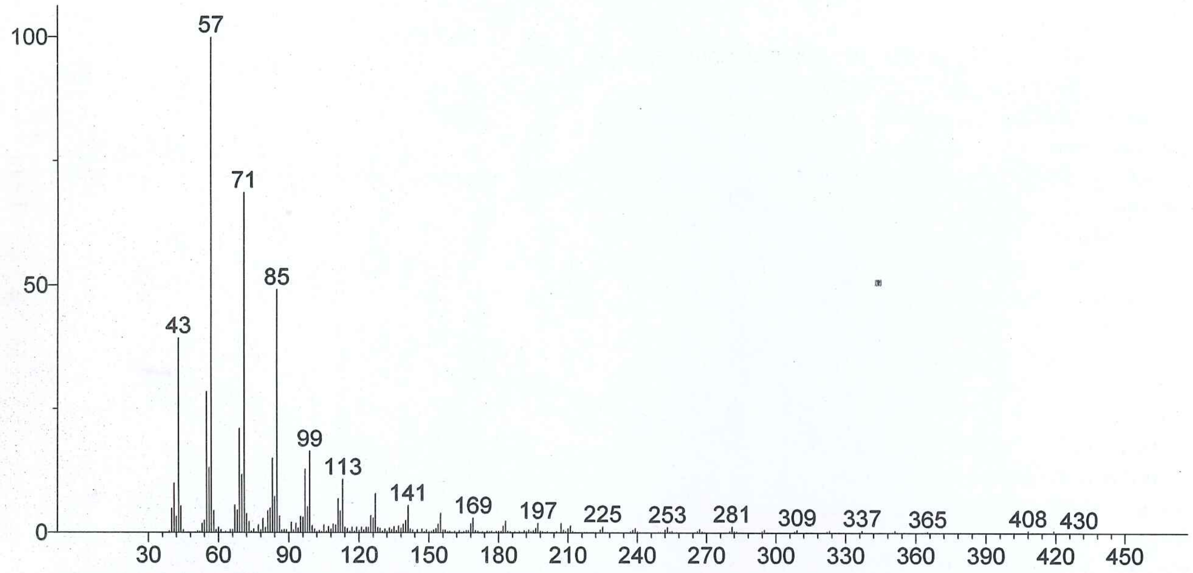

(Text File) +EI Scan (45.323 min) AASIA-KIG-HEX-1a-040622.D

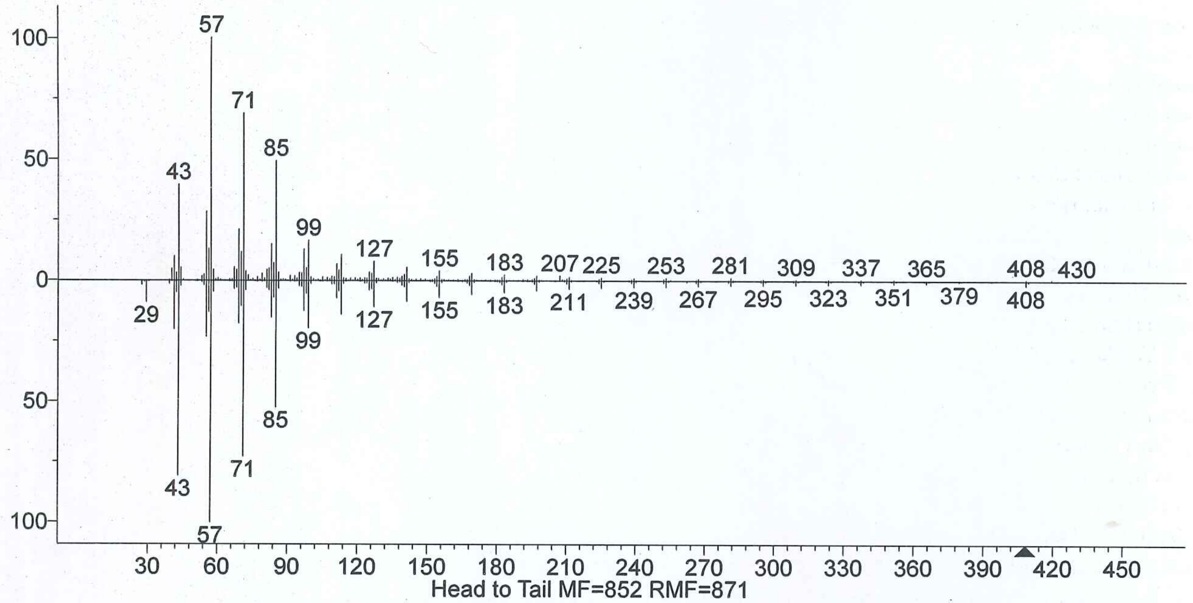

Head to Tail MF=852 RMF=871

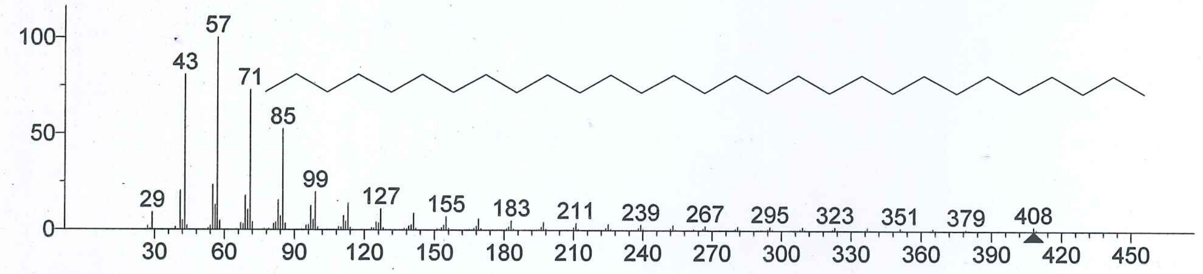

(replib) Nonacosane

Name: Nonacosane
Formula: $C_{29}H_{60}$
MW: 408 CAS#: 630-03-5 NIST#: 197624 ID#: 5478 DB: replib
Other DBs: Fine, TSCA, HODOC, EINECS
Contributor: Chemical Concepts
10 largest peaks:
57 999 | 43 806 | 71 727 | 85 523 | 55 232 | 41 201 | 99 196 | 69 177 | 83 152 | 113 139 |
Synonyms:
1.n-Nonacosane

Estimated non-polar retention index (n-alkane scale):
Value: 2904 iu
Confidence interval (Hydrocarbons): 39(50%) 167(95%) iu

Retention index.
1. Value: 2900 iu
Column Class: All column types
Data Type: Normal alkane RI value specified by scale definition
Source: von Kováts, E., 206. Gas-chromatographische Charakterisierung organischer Verbindungen. Teil 1: Retentionsindices aliphatischer Halogenide, Alkohole, Aldehyde und Ketone, Helv. Chim. Acta, 41(7), 1958, 1915 -1932.

2. Value: 475.3 iu
Column Type: Capillary
Column Class: Standard non-polar
Active Phase: DB
-1
Column Length: 30 m
Column Diameter: 0.2 mm
Phase Thickness: 0.25 um
Data Type: Lee RI
Program
Type: Ramp
Start T: 50 C
End T: 300 C
Heat Rate: 5 K/min
Start Time: 2 min
End Time: 5 min
Source:
Johnson, C.I.; Urso, A.; Geleta, L., Broad spectrum analysis of municipal and industrial effluents discharged into the Peace, Athabasca and Slave river basins: characterization of effluent samples, 1994 - Volume 1 of 2, Northern River Basins Study Project Report No. 121, Norther River Basins Study, Edmonton, Alberta, 1997, 27.

<...>

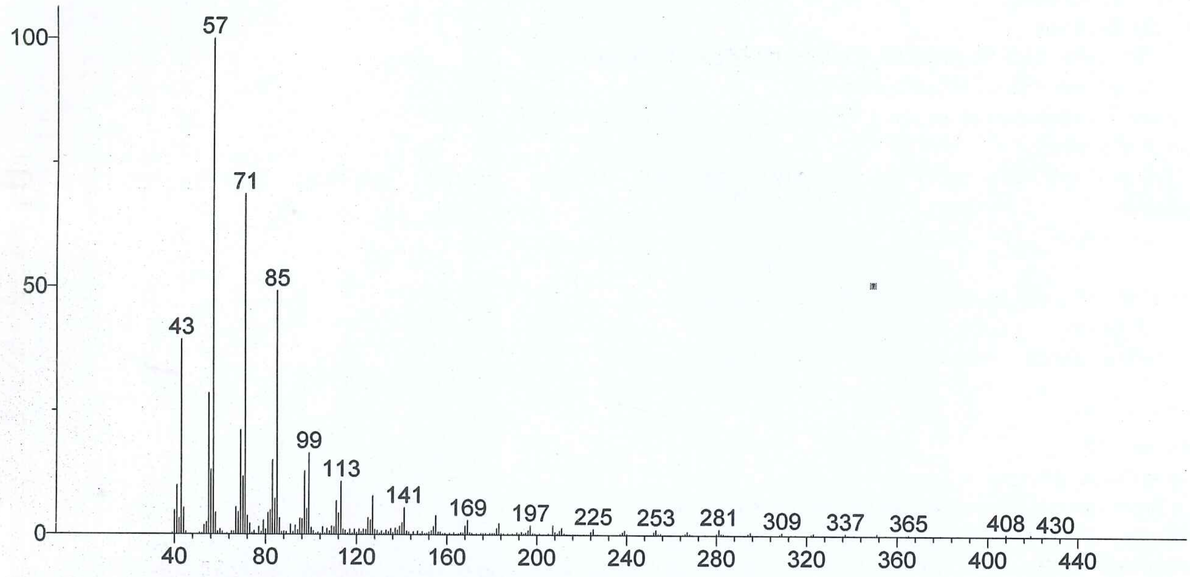

(Text File) +EI Scan (45.323 min) AASIA-KIG-HEX-1a-040622.D

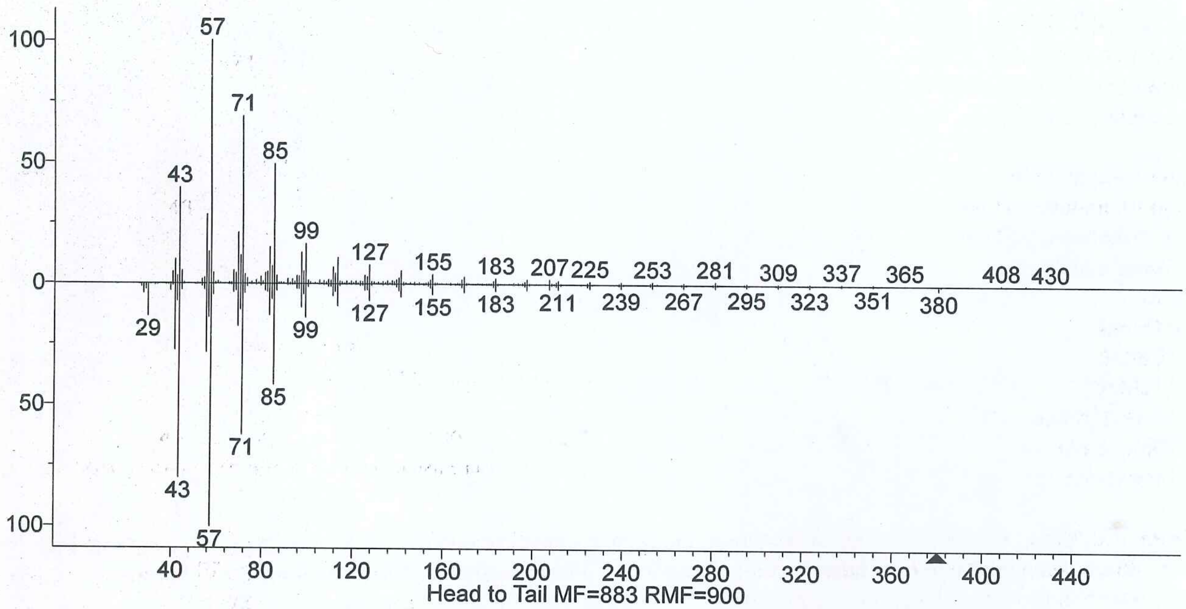

Head to Tail MF=883 RMF=900

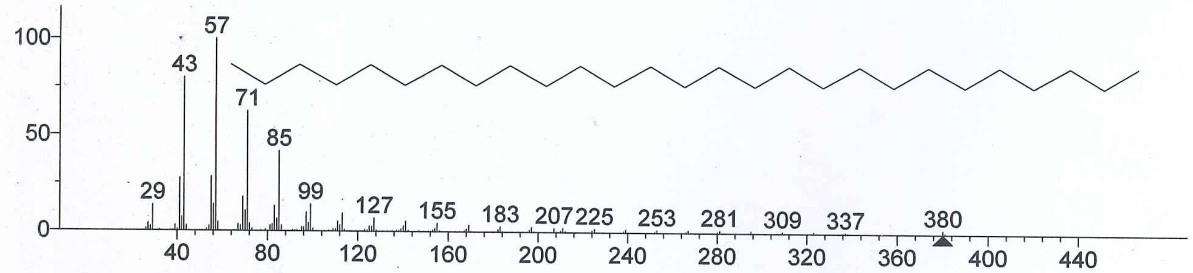

(replib) Heptacosane

Name: Heptacosane
Formula: $C_{27}H_{56}$
MW: 380 CAS#: 593-49-7 NIST#: 79427 ID#: 5508 DB: replib
Other DBs: Fine, EPA, HODOC, EINECS
Contributor: O A MAMER, MCGILL UNIVERSITY, MONTREAL, CANA
10 largest peaks:
57 999 | 43 798 | 71 622 | 85 416 | 55 283 | 41 275 | 69 176 | 99 140 | 56 139 | 29 135 |
Synonyms:
1.n-Heptacosane

Estimated non-polar retention index (n-alkane scale):
Value: 2705 iu
Confidence interval (Hydrocarbons): 39(50%) 167(95%) iu

Retention index.
1. Value: 2700 iu
Column Class: All column types
Data Type: Normal alkane RI value specified by scale definition
Source: von Kováts, E., 206. Gas-chromatographische Charakterisierung organischer Verbindungen. Teil 1: Retentionsindices aliphatischer Halogenide, Alkohole, Aldehyde und Ketone, Helv. Chim. Acta, 41(7), 1958, 1915 -1932.

2. Value: 447.8 iu
Column Type: Capillary
Column Class: Standard non-polar
Active Phase: DB
-1
Column Length: 30 m
Column Diameter: 0.2 mm
Phase Thickness: 0.25 um
Data Type: Lee RI
Program
Type: Ramp
Start T: 50 C
End T: 300 C
Heat Rate: 5 K/min
Start Time: 2 min
End Time: 5 min
Source:
Johnson, C.I.; Urso, A.; Geleta, L., Broad spectrum analysis of municipal and industrial effluents discharged into the Peace, Athabasca and Slave river basins: characterization of effluent samples, 1994 - Volume 1 of 2, Northern River Basins Study Project Report No. 121, Norther River Basins Study, Edmonton, Alberta, 1997, 27.

<...>

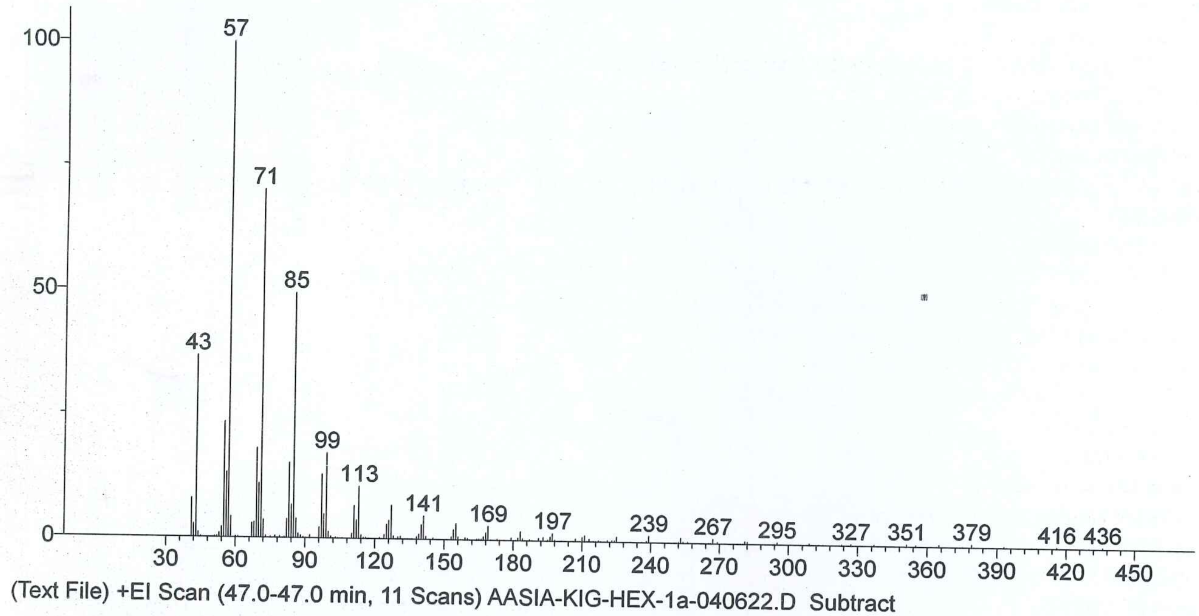

(Text File) +EI Scan (47.0-47.0 min, 11 Scans) AASIA-KIG-HEX-1a-040622.D Subtract

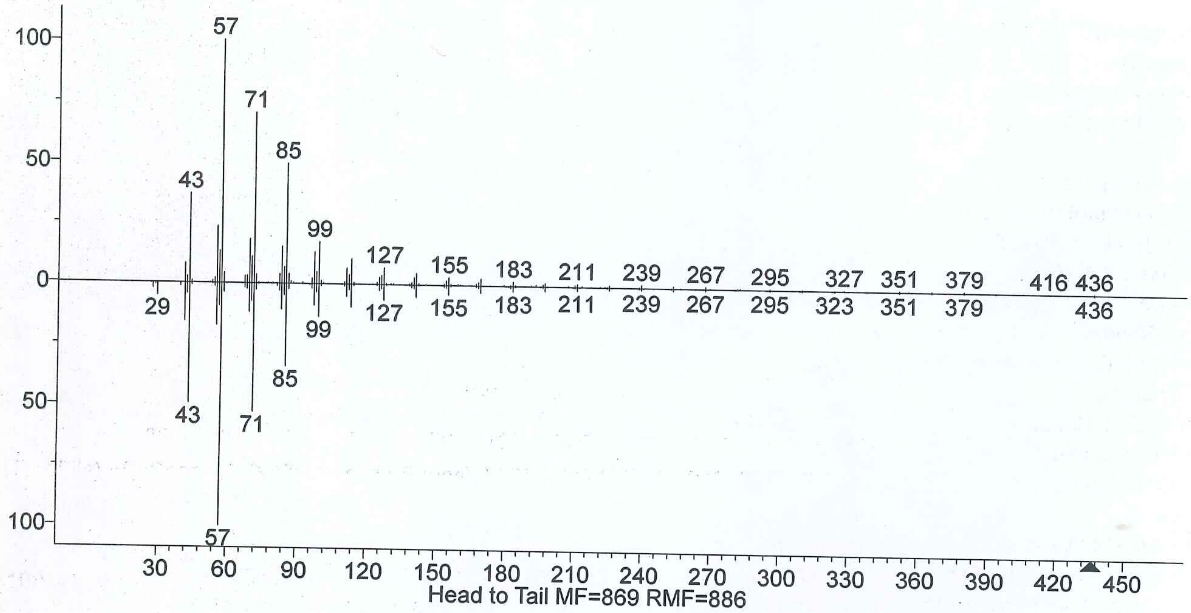

Head to Tail MF=869 RMF=886

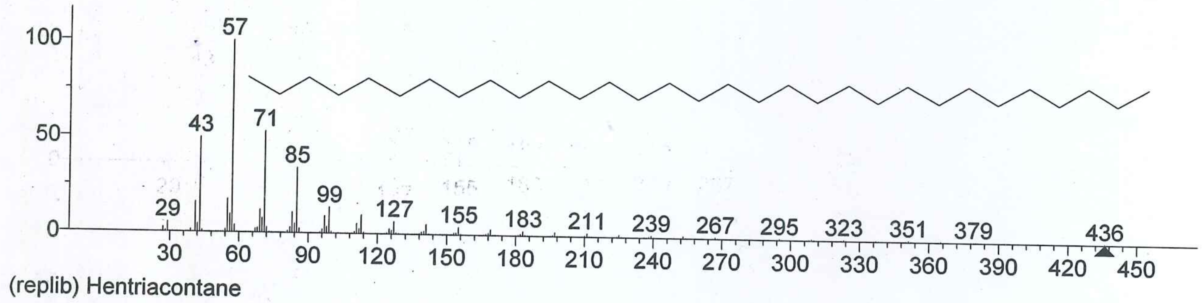

(replib) Hentriacontane

Name: Hentriacontane
Formula: $C_{31}H_{64}$
MW: 436 CAS#: 630-04-6 NIST#: 150572 ID#: 5728 DB: replib
Other DBs: None
Contributor: Chemical Concepts
10 largest peaks:
57 999 | 71 528 | 43 494 | 85 340 | 55 173 | 41 157 | 99 136 | 69 120 | 83 107 | 56 95 |
Synonyms:
1.n-Hentriacontane
2.Untriacontane

Estimated non-polar retention index (n-alkane scale):
Value: 3103 iu
Confidence interval (Hydrocarbons): 39(50%) 167(95%) iu

Retention index.
1. Value: 3100 iu
Column Class: All column types
Data Type: Normal alkane RI value specified by scale definition
Source: von Kováts, E., 206. Gas-chromatographische Charakterisierung organischer Verbindungen. Teil 1: Retentionsindices aliphatischer Halogenide, Alkohole, Aldehyde und Ketone, Helv. Chim. Acta, 41(7), 1958, 1915 -1932.

2. Value: 472.7 iu
Column Type: Capillary
Column Class: Semi-standard non-polar
Active Phase: DB
-5
Column Length: 60 m
Data Type: Lee RI
Program Type: Ramp
Source: Fuentes, M.J.; Font, R.; Gomez-Rico, M.F.; Martin-Gullon, I., Pyrolysis and combustion of waste lubricant oil from diesel cars: Decomposition and pollutants, J. Anal. Appl. Pyrolysis, 79, 2007, 215-226.

<...>

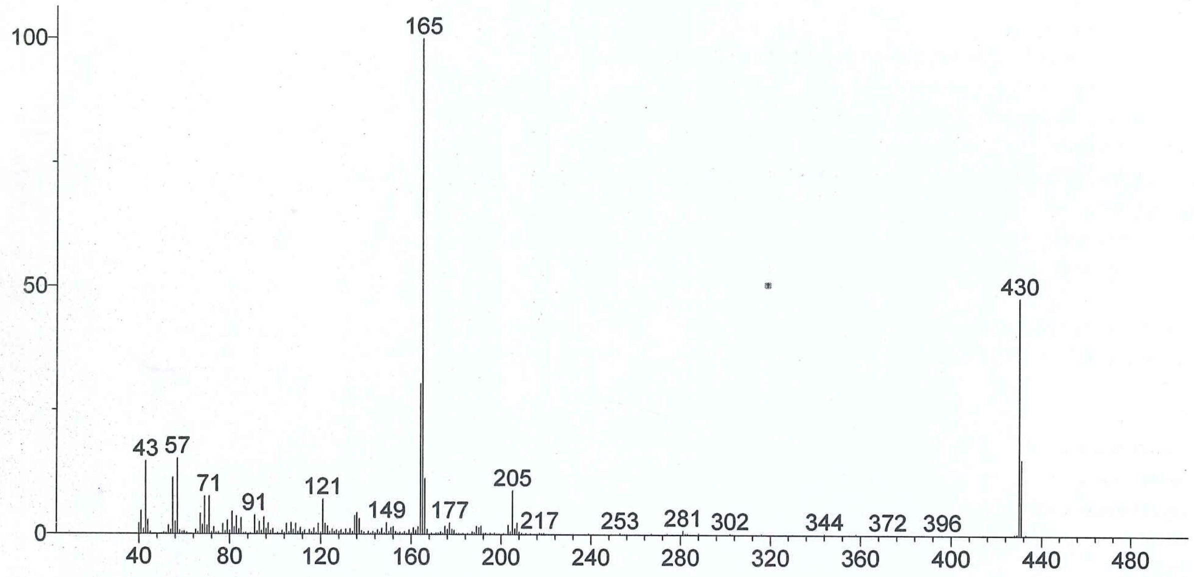

(Text File) +EI Scan (47.3 min) AASIA-KIG-HEX-1a-040622.D

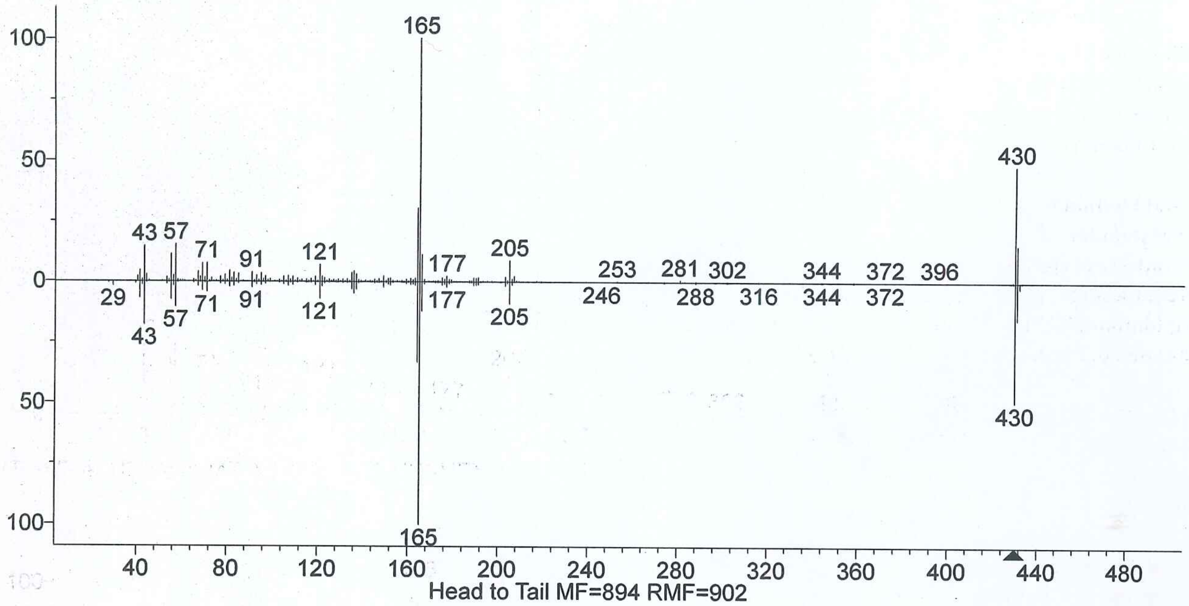

Head to Tail MF=894 RMF=902

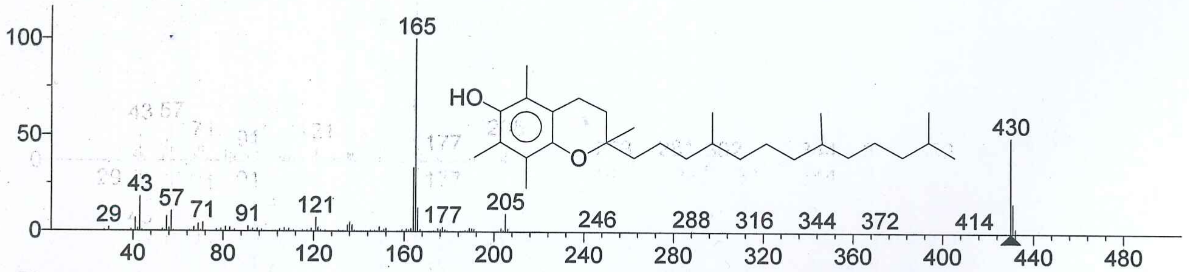

(replib) Vitamin E

Name: Vitamin E
Formula: $C_{29}H_{50}O_2$
MW: 430 CAS#: 59-02-9 NIST#: 290780 ID#: 21723 DB: replib
Other DBs: TSCA, RTECS, HODOC, NIH, EINECS
Contributor: NIST Mass Spectrometry Data Center, 1998.
10 largest peaks:
165 999 | 430 494 | 164 332 | 43 178 | 431 155 | 166 122 | 57 104 | 205 91 | 55 75 | 121 71 |
Synonyms:
1.2H-1-Benzopyran-6-ol, 3,4-dihydro-2,5,7,8-tetramethyl-2-(4,8,12-trimethyltridecyl)-, [2R-[2R*(4R*,8R*)]]-
2.α-Tocopherol
3.α-Tokoferol
4.(All-R)-α-Tocopherol
5.(2R,4'R,8'R)-α-Tocopherol
6.D-α-tocopherol
7.Almefrol
8.Antisterility vitamin
9.Denamone
10.Emipherol
11.Endo E
12.Ephynal
13.Eprolin
14.Eprolin S
15.Epsilan
16.Esorb
17.Etamican
18.Etavit
19.Evion
20.Evitaminum
21.Ilitia
22.Phytogermine
23.Profecundin
24.Spavit E
25.Syntopherol
26.Tokopharm
27.Vascuals
28.Verrol
29.Vi-E
30.Vitaplex E
31.Vitayonon
32.Viteolin
33.5,7,8-Trimethyltocol
34.component of E and C-Level
35.component of Estopherol
36.Aquasol E
37.Lan-E
38.Med-E
39.Vita E
40.Covi-ox
41.Spavit
42.(R,R,R)-α-Tocopherol
43.(+)-α-Tocopherol
44.(+)-α-Tocopherolα-tocopherolantisterility vitamin
45.[2R-2R*(4R*,8R*)]-3,4-Dihydro-2,5,7,8-tetramethyl-2-(4,8,12-trimethyltridecyl)-2H-1-benzopyran-6-ol
46.2,5,7,8-Tetramethyl-2-(4',8',12'-trimethyltridecyl)-6-chromanol

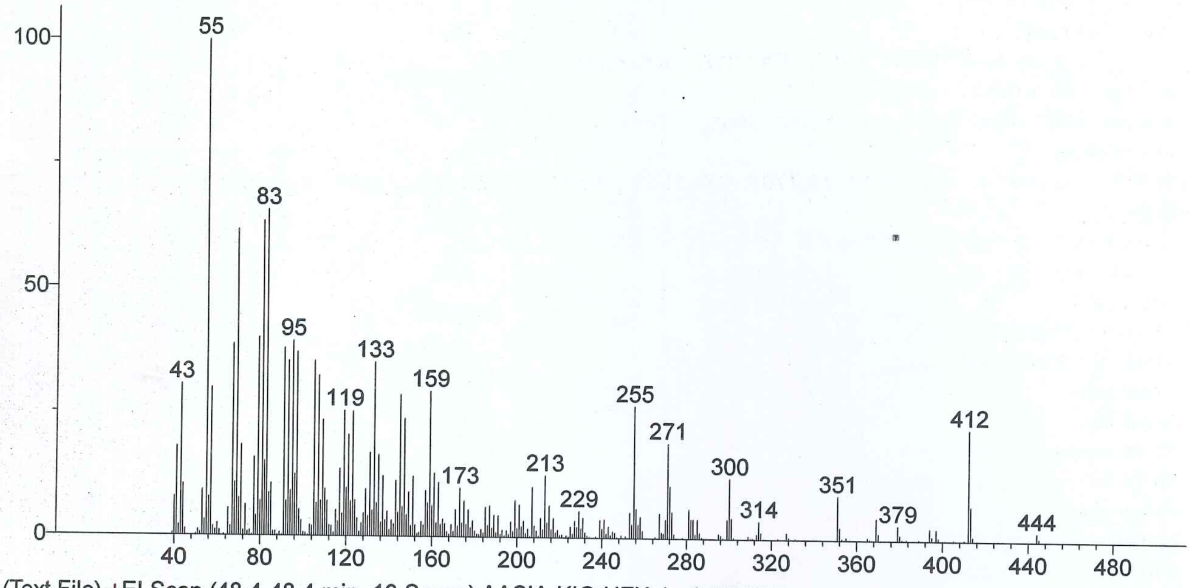

(Text File) +EI Scan (48.4-48.4 min, 13 Scans) AASIA-KIG-HEX-1a-040622.D

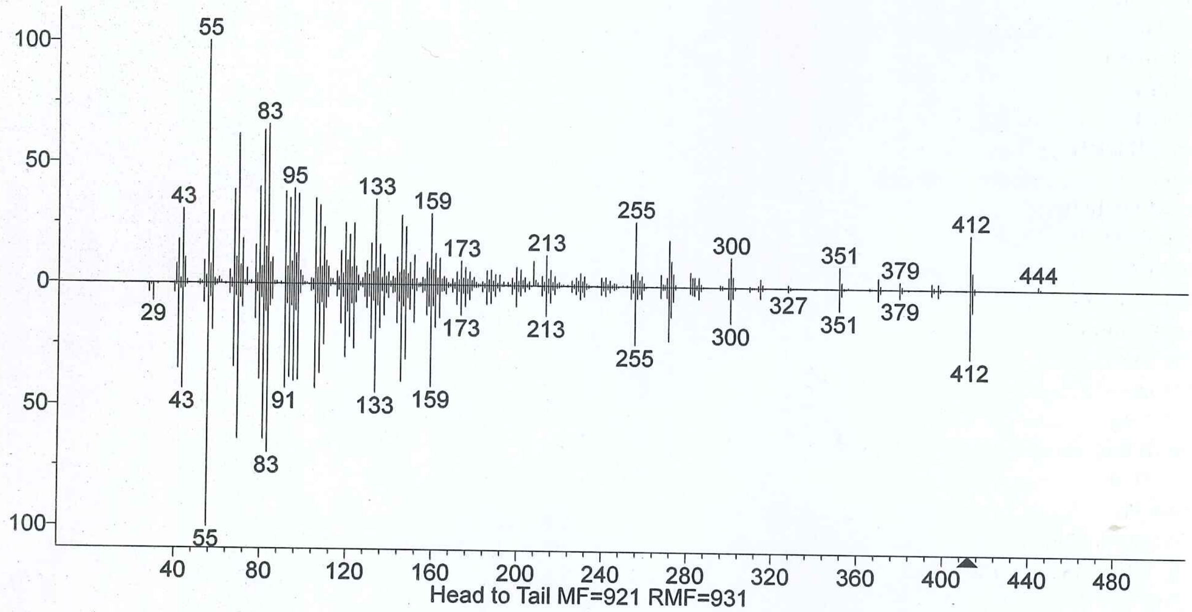

Head to Tail MF=921 RMF=931

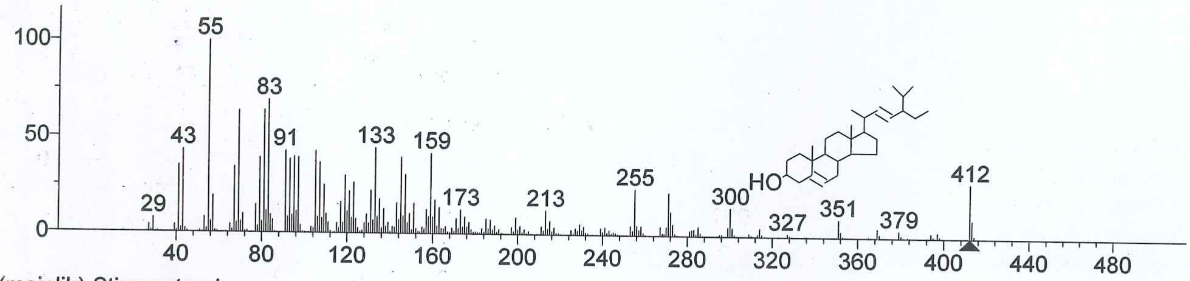

(mainlib) Stigmasterol

Name: Stigmasterol
Formula: $C_{29}H_{48}O$
MW: 412 CAS#: 83-48-7 NIST#: 352610 ID#: 18876 DB: mainlib
Other DBs: Fine, HODOC, NIH, EINECS
Contributor: NIST Mass Spectrometry Data Center
10 largest peaks:
55 999 | 83 692 | 81 638 | 69 636 | 133 444 | 43 431 | 91 427 | 105 427 | 159 418 | 95 398 |
Synonyms:
1.Stigmasta-5,22-dien-3-ol, (3β,22E)-
2.Stigmasta-5,22-dien-3β-ol
3.β-Stigmasterol
4.(24S)-5,22-Stigmastadien-3β-ol
5.Stigmasta-5,22-dien-3-ol, (3β)-
6.Stigmasterin
7.Phytosterol
8.5,22-Cholestadien-24-ethyl-3β-ol
9..DELTA.5,22-Stigmastadien-3β-ol
10.I-Stigmasterol
11.Stigmasta-5,22-dien-3-ol
12.(22E)-Stigmasta-5,22-dien-3-ol #

Estimated non-polar retention index (n-alkane scale):
Value: 2739 iu
Confidence interval (Low reliability): 174(50%) 752(95%) iu

Retention index.
1. Value: 3221.93 iu
Column Type: Capillary
Column Class: Standard non-polar
Active Phase: DB-1
Column
Length: 30 m
Carrier Gas: H2
Column Diameter: 0.25 mm
Phase Thickness: 0.25 um
Data Type: Kovats
RI
Program Type: Isothermal
Start T: 270 C
Source: Stránský, K.; Valterová, I.; Fiedler, P., Nonsaponifiable lipid
components of the pollen of elder (Sambucus nigra L.), J. Chromatogr. A, 936, 2001, 173-181.

2. Value: 3222.15
iu
Column Type: Capillary
Column Class: Standard non-polar
Active Phase: DB-1
Column Length: 30
m
Carrier Gas: H2
Column Diameter: 0.25 mm
Phase Thickness: 0.25 um
Data Type: Kovats RI
Program

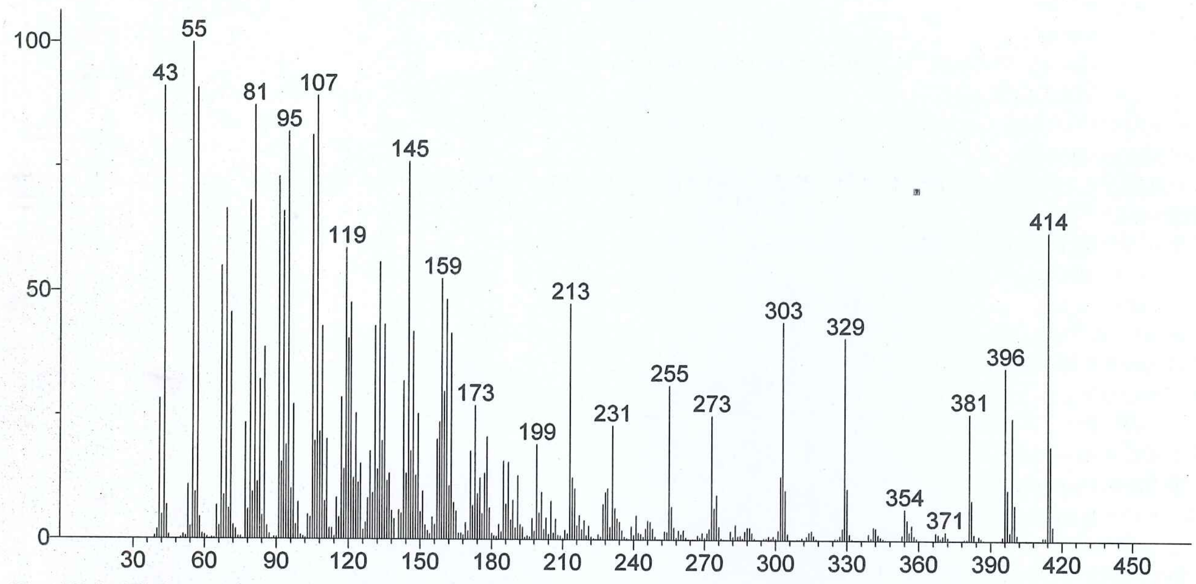

(Text File) +EI Scan (49.0 min) AASIA-KIG-HEX-1a-040622.D

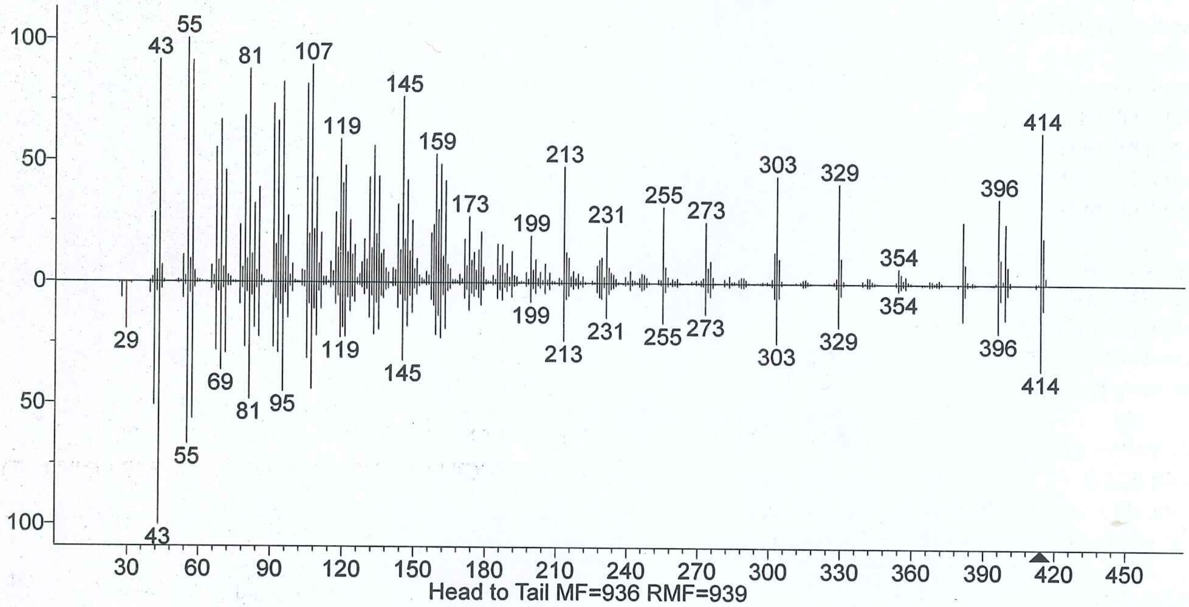

Head to Tail MF=936 RMF=939

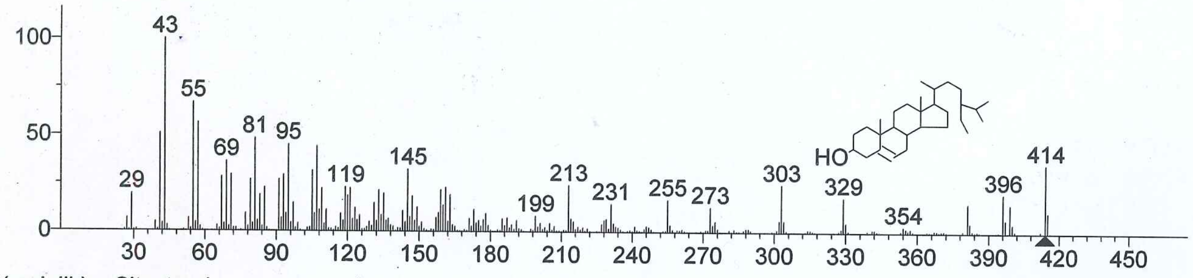

(mainlib) γ-Sitosterol

Name: γ-Sitosterol
Formula: $C_{29}H_{50}O$
MW: 414 CAS#: 83-47-6 NIST#: 151558 ID#: 6743 DB: mainlib
Other DBs: HODOC, EINECS
Contributor: Chemical Concepts
10 largest peaks:
43 999 | 55 667 | 57 563 | 41 507 | 81 481 | 95 449 | 107 440 | 69 362 | 414 354 | 145 321 |
Synonyms:
1.Stigmast-5-en-3-ol, (3β,24S)-
2.Stigmast-5-en-3β-ol, (24S)-
3.Clionasterol
4.Fucosterol, β-dihydro-
5.24β-Ethyl-5-cholesten-3β-ol
6.β-Dihydrofucosterol
7.22,23-Dihydroporiferasterol
8.24S-Ethylcholest-5-en-3β-ol
9.24β-Ethylcholesterol
10.Stigmast-5-en-3-ol #

Estimated non-polar retention index (n-alkane scale):
Value: 2731 iu
Confidence interval (Low reliability): 174(50%) 752(95%) iu

Retention index.
1. Value: 3066 iu
Column Type: Capillary
Column Class: Semi-standard non-polar
Active Phase: HP-5
Column
Length: 30 m
Carrier Gas: He
Column Diameter: 0.25 mm
Phase Thickness: 0.25 um
Data Type: Normal
alkane RI
Program Type: Complex
Description: 60C(5min) =>3C/min =>120C (2min) =>2C/min =>200C (2min)
=>3C/min =>320C
Source: Yasar, A.; Üçüncü, O.; Güleç, C.; Inceer, H.; Ayaz, S.; Yayh, N., GC-MS analysis of chloroform extracts in flowers, stems, and roots of Tripleurospermum callosum, Pharm. Biol., 43(2), 2005, 108-112.

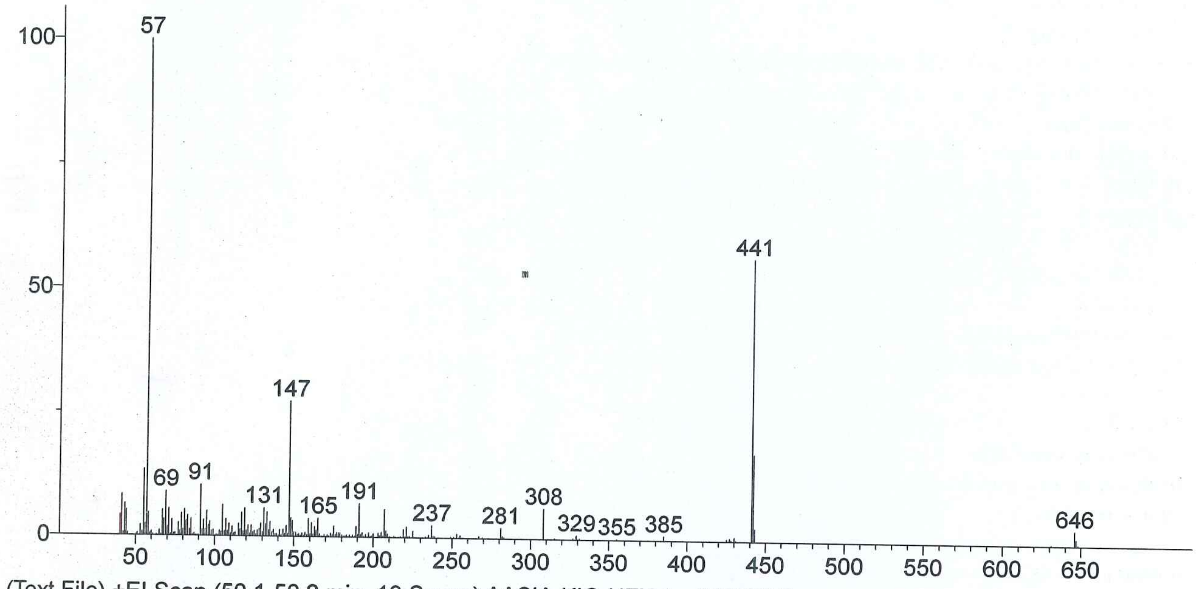

(Text File) +EI Scan (50.1-50.2 min, 19 Scans) AASIA-KIG-HEX-1a-040622.D

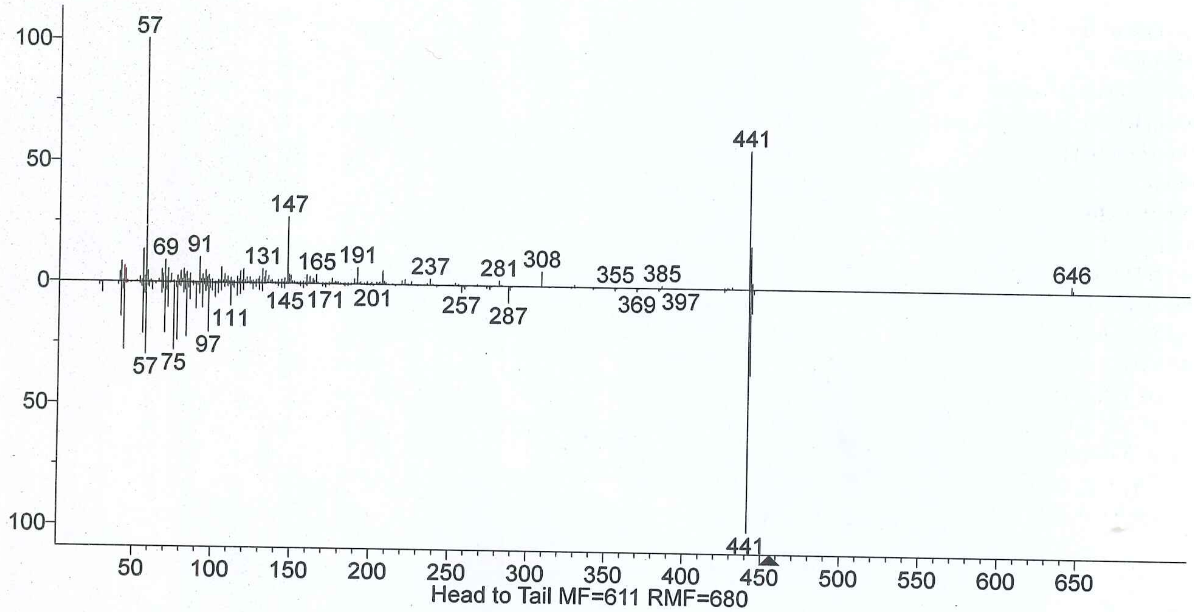

Head to Tail MF=611 RMF=680

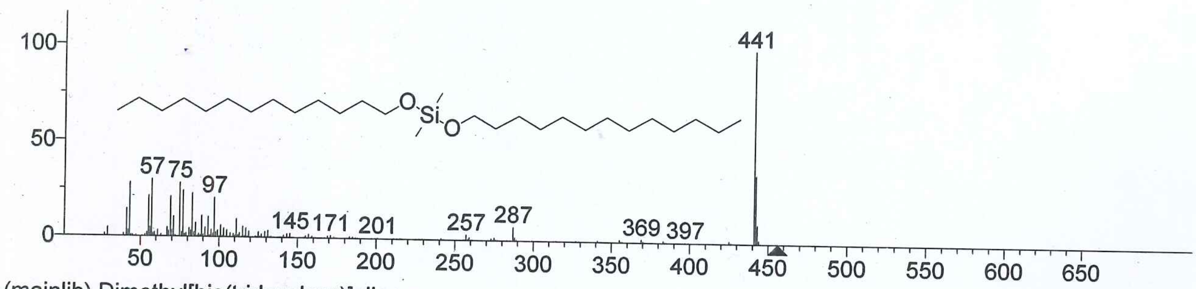

(mainlib) Dimethyl[bis(tridecyloxy)]silane

Name: Dimethyl[bis(tridecyloxy)]silane
Formula: $C_{28}H_{60}O_2Si$
MW: 456 NIST#: 334076 ID#: 189572 DB: mainlib
Contributor: NIST Mass Spectrometry Data Center
10 largest peaks:
441 999 | 442 354 | 57 296 | 43 279 | 75 279 | 77 239 | 83 225 | 55 212 | 69 208 | 97 203 |
Synonyms:
no synonyms.

Estimated non-polar retention index (n-alkane scale):
Value: 2866 iu
Confidence interval (Diverse functional groups): 89(50%) 382(95%) iu

Retention index.
1. Value: 2771 iu
Column Type: Capillary
Column Class: Semi-standard non-polar
Active Phase: VF
-5MS
Column Length: 30 m
Carrier Gas: He
Column Diameter: 0.25 mm
Phase Thickness: 0.25 um
Data Type:
Linear RI
Program Type: Ramp
Start T: 60 C
End T: 270 C
Source: Tret'yakov, K.V., Retention Data. NIST
Mass Spectrometry Data Center., 2007.

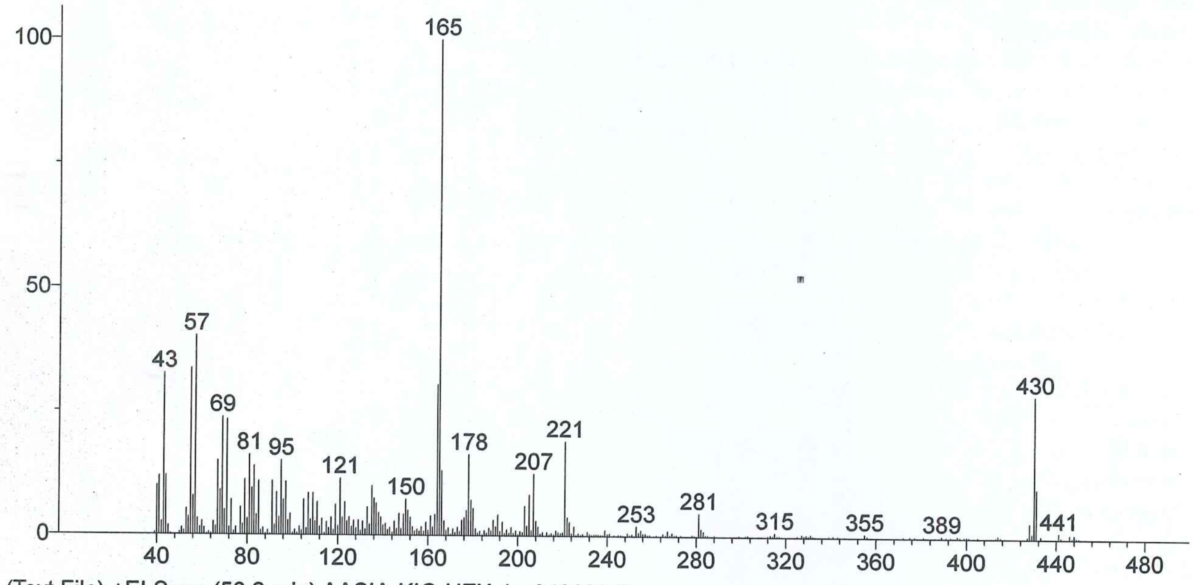

(Text File) +EI Scan (50.2 min) AASIA-KIG-HEX-1a-040622.D

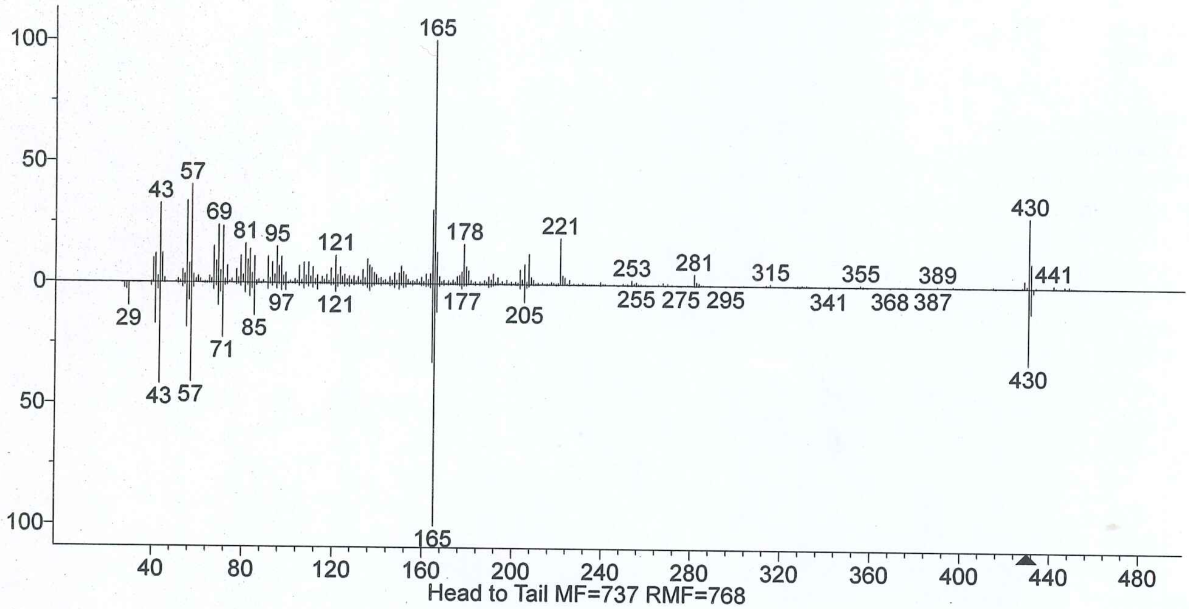

Head to Tail MF=737 RMF=768

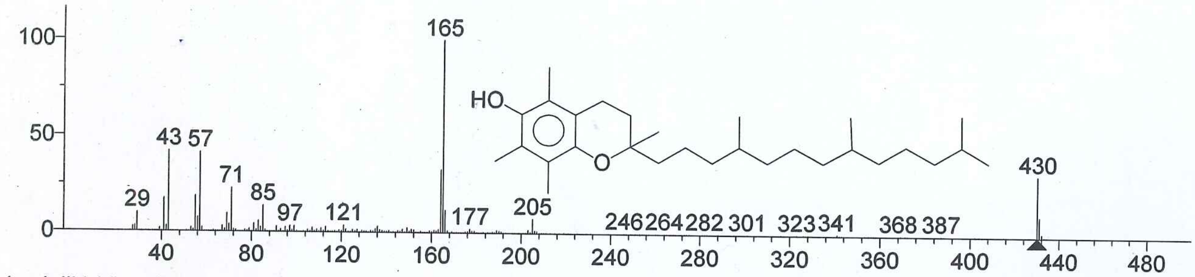

(mainlib) Vitamin E

Name: Vitamin E
Formula: $C_{29}H_{50}O_2$
MW: 430 CAS#: 59-02-9 NIST#: 151382 ID#: 122577 DB: mainlib
Other DBs: TSCA, RTECS, HODOC, NIH, EINECS
Contributor: Chemical Concepts
10 largest peaks:
165 999 | 43 419 | 57 410 | 164 326 | 430 318 | 71 226 | 55 185 | 41 171 | 85 135 | 166 117 |
Synonyms:
1.2H-1-Benzopyran-6-ol, 3,4-dihydro-2,5,7,8-tetramethyl-2-(4,8,12-trimethyltridecyl)-, [2R-[2R*(4R*,8R*)]]-
2.α-Tocopherol
3.α-Tokoferol
4.(All-R)-α-Tocopherol
5.(2R,4'R,8'R)-α-Tocopherol
6.D-α-tocopherol
7.Almefrol
8.Antisterility vitamin
9.Denamone
10.Emipherol
11.Endo E
12.Ephynal
13.Eprolin
14.Eprolin S
15.Epsilan
16.Esorb
17.Etamican
18.Etavit
19.Evion
20.Evitaminum
21.Ilitia
22.Phytogermine
23.Profecundin
24.Spavit E
25.Syntopherol
26.Tokopharm
27.Vascuals
28.Verrol
29.Vi-E
30.Vitaplex E
31.Vitayonon
32.Viteolin
33.5,7,8-Trimethyltocol
34.component of E and C-Level
35.component of Estopherol
36.Aquasol E
37.Lan-E
38.Med-E
39.Vita E
40.Covi-ox
41.Spavit
42.(R,R,R)-α-Tocopherol
43.(+)-α-Tocopherol
44.(+)-α-Tocopherolα-tocopherolantisterility vitamin
45.[2R-2R*(4R*,8R*)]-3,4-Dihydro-2,5,7,8-tetramethyl-2-(4,8,12-trimethyltridecyl)-2H-1-benzopyran-6-ol
46.2,5,7,8-Tetramethyl-2-(4',8',12'-trimethyltridecyl)-6-chromanol

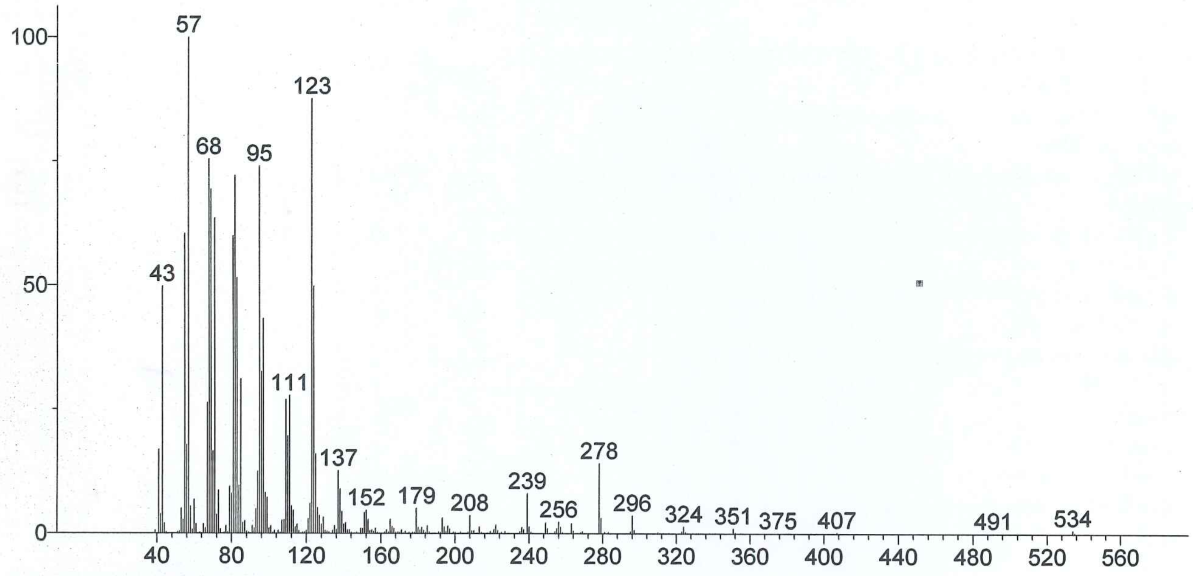

(Text File) +EI Scan (51.7 min) AASIA-KIG-HEX-1a-040622.D Subtract

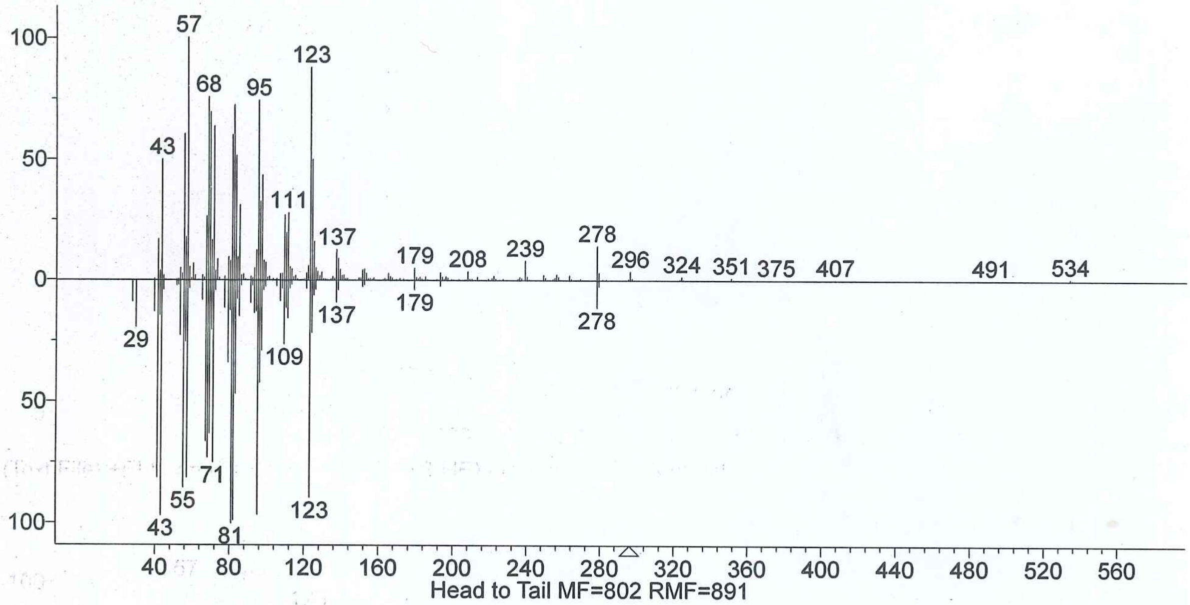

Head to Tail MF=802 RMF=891

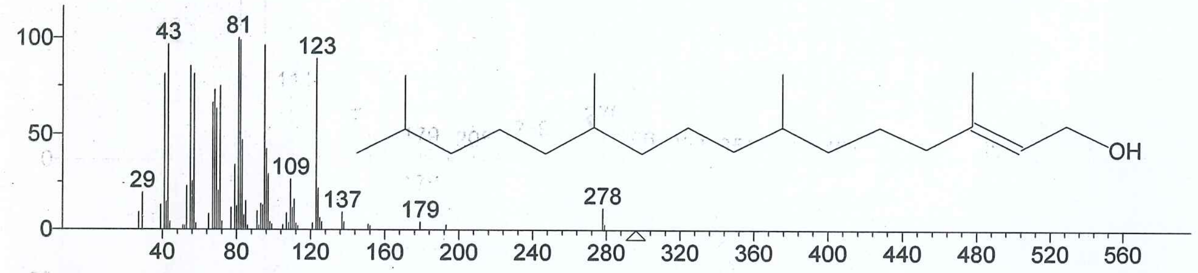

(mainlib) 3,7,11,15-Tetramethyl-2-hexadecen-1-ol

Name: 3,7,11,15-Tetramethyl-2-hexadecen-1-ol
Formula: $C_{20}H_{40}O$
MW: 296 CAS#: 102608-53-7 NIST#: 114703 ID#: 43206 DB: mainlib
Other DBs: IRDB
Contributor: NIST Mass Spectrometry Data Center, 1990.
10 largest peaks:
81 999 | 82 986 | 43 965 | 95 962 | 123 892 | 55 852 | 41 811 | 57 811 | 71 748 | 68 728 |
Synonyms:
1.(2E)-3,7,11,15-Tetramethyl-2-hexadecen-1-ol #

Estimated non-polar retention index (n-alkane scale):
Value: 2045 iu
Confidence interval (Alcohols): 41(50%) 176(95%) iu

Retention index.
1. Value: 2119.33 iu
Column Type: Capillary
Column Class: Semi-standard non-polar
Active Phase: SE
-54
Column Length: 25 m
Column Diameter: 0.31 mm
Data Type: Linear RI
Program Type: Ramp
Start T: 35
C
End T: 230 C
Heat Rate: 4 K/min
Start Time: 3 min
End Time: 10 min
Source: Yin, W.; Xiu, Z.; Aijin, H.,
Analysis of the volatile components in trogopterorum feces by capillary gas chromatography and gas chromatography/mass spectrometry, Fenxi Huaxue, 29(2), 2001, 195-198.

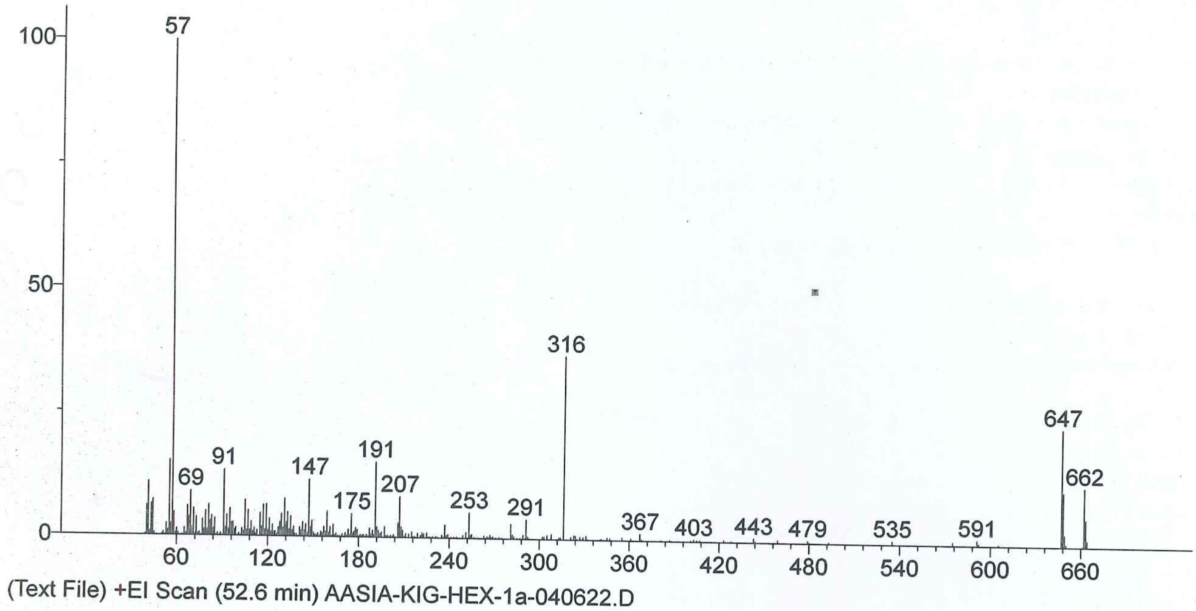

(Text File) +EI Scan (52.6 min) AASIA-KIG-HEX-1a-040622.D

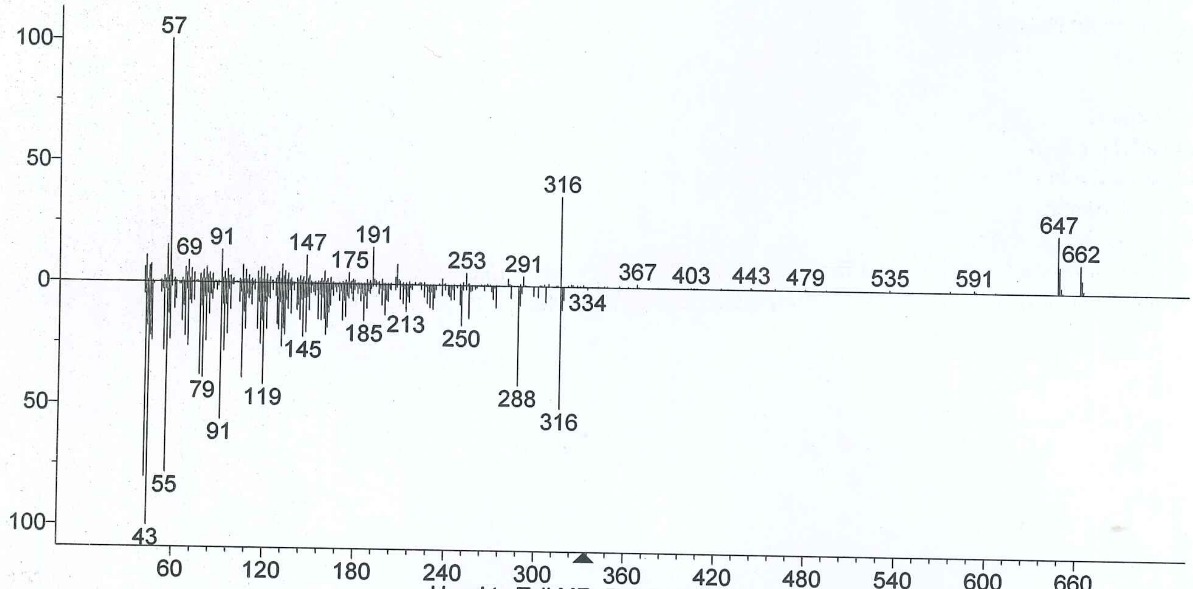

Head to Tail MF=543 RMF=702

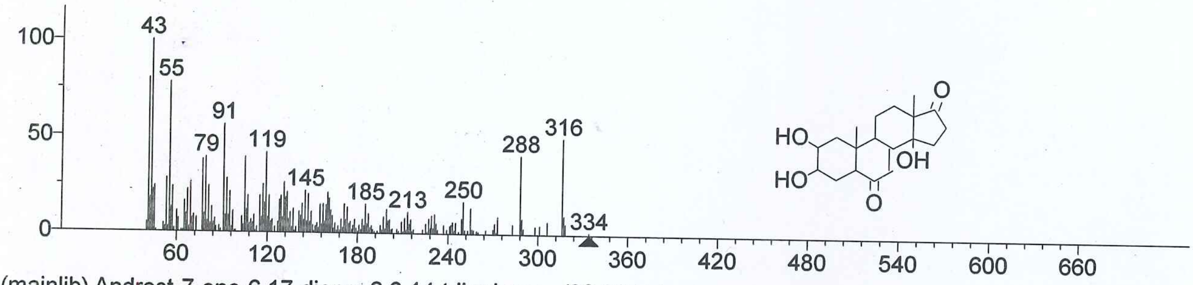

(mainlib) Androst-7-ene-6,17-dione, 2,3,14-trihydroxy-, (2β,3β,5α)-

Name: Androst-7-ene-6,17-dione, 2,3,14-trihydroxy-, (2β,3β,5α)-
Formula: $C_{19}H_{26}O_5$
MW: 334 CAS#: 55191-58-7 NIST#: 15527 ID#: 5584 DB: mainlib
Other DBs: None
Contributor: G.SCHULZ SCHERING AG, BERLIN, E.GERMANY.
10 largest peaks:
43 999 | 41 800 | 55 780 | 91 560 | 316 500 | 119 415 | 288 408 | 79 390 | 105 390 | 77 378 |
Synonyms:
1.2,3,14-Trihydroxyandrost-7-ene-6,17-dione #

Estimated non-polar retention index (n-alkane scale):
Value: 2649 iu
Confidence interval (Low reliability): 174(50%) 752(95%) iu

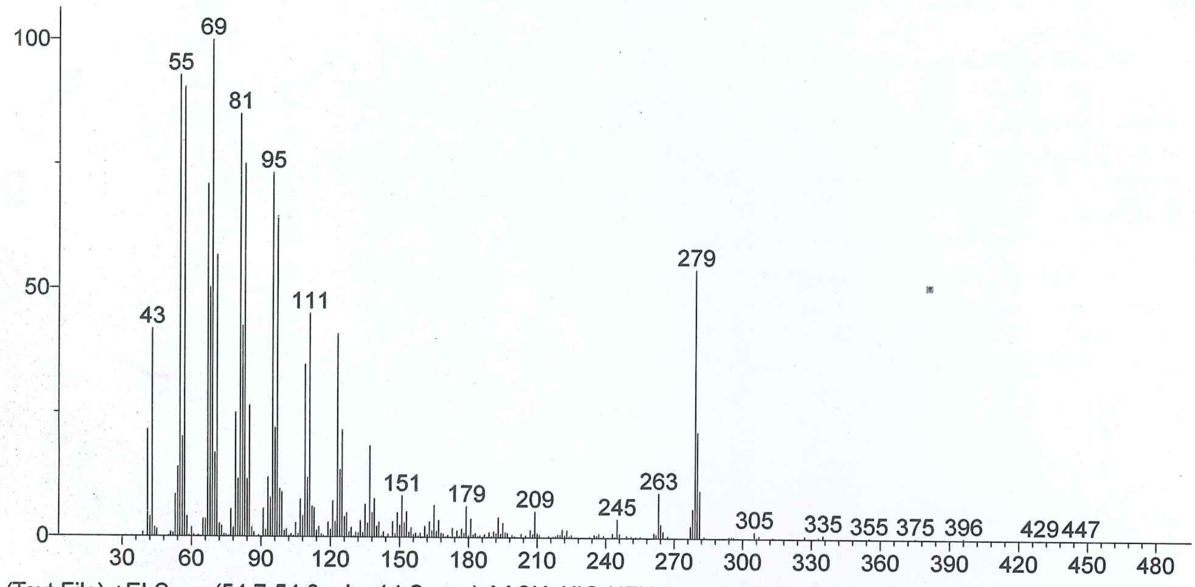

(Text File) +EI Scan (54.7-54.8 min, 14 Scans) AASIA-KIG-HEX-1a-040622.D Subtract

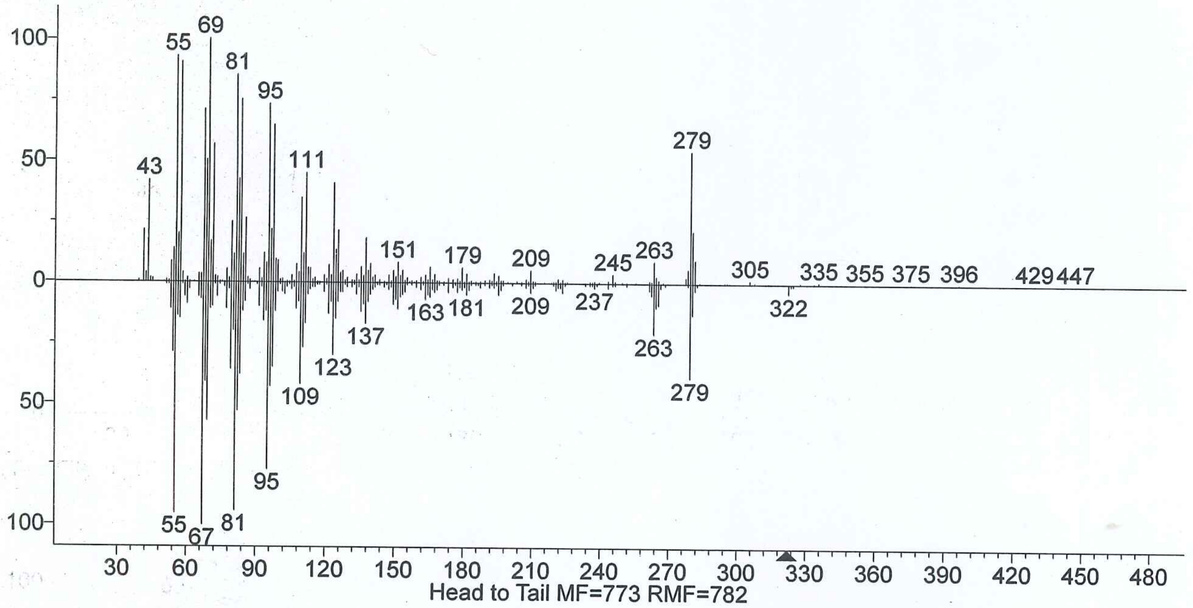

Head to Tail MF=773 RMF=782

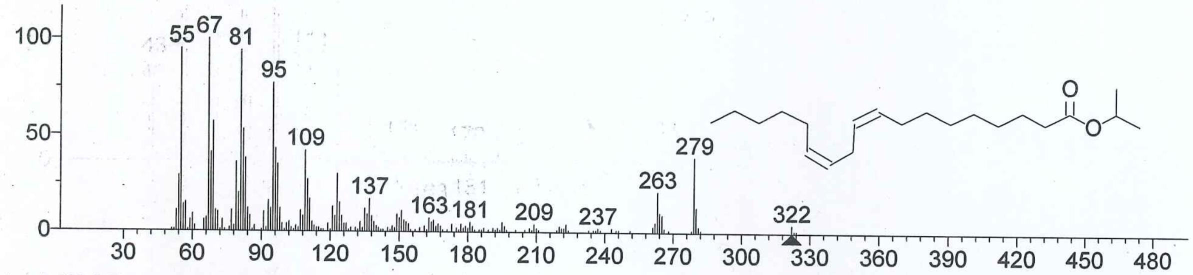

(mainlib) i-Propyl 9,12-octadecenadienoate

Name: i-Propyl 9,12-octadecenadienoate
Formula: $C_{21}H_{38}O_2$
MW: 322 NIST#: 336798 ID#: 28653 DB: mainlib
Contributor: William W. Christie, Mylnefield Lipid Analysis, Invergowrie, Dundee, Scotland, UK
10 largest peaks:
67 999 | 55 949 | 81 939 | 95 769 | 69 569 | 82 529 | 96 429 | 109 419 | 68 409 | 279 389 |
Synonyms:
no synonyms.

Estimated non-polar retention index (n-alkane scale):
Value: 2228 iu
Confidence interval (Esters): 47(50%) 201(95%) iu

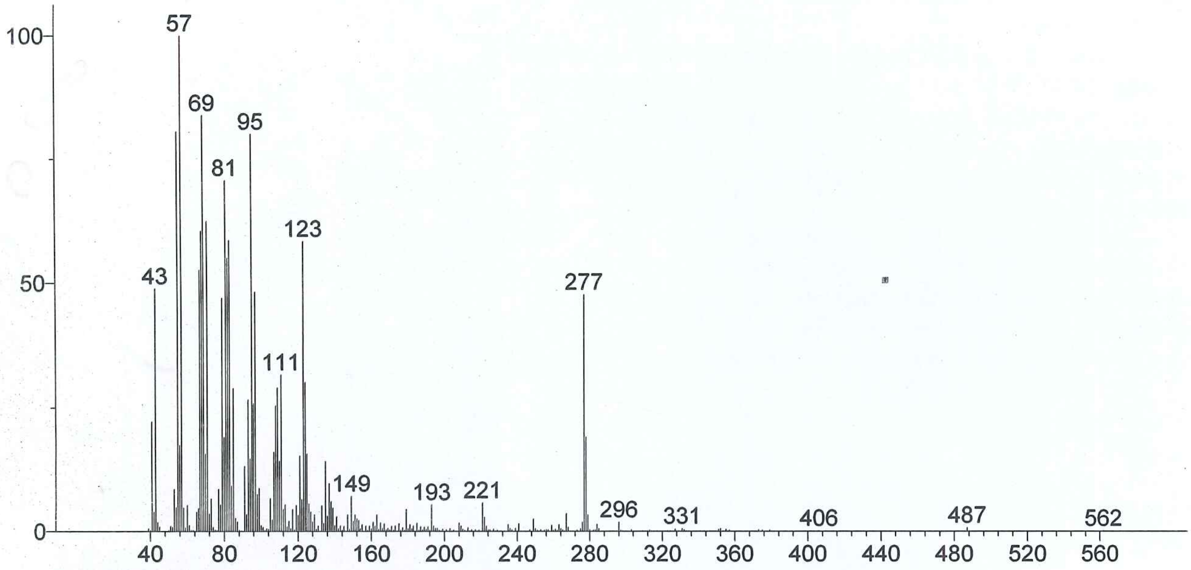

(Text File) +EI Scan (55.0-55.1 min, 28 Scans) AASIA-KIG-HEX-1a-040622.D Subtract

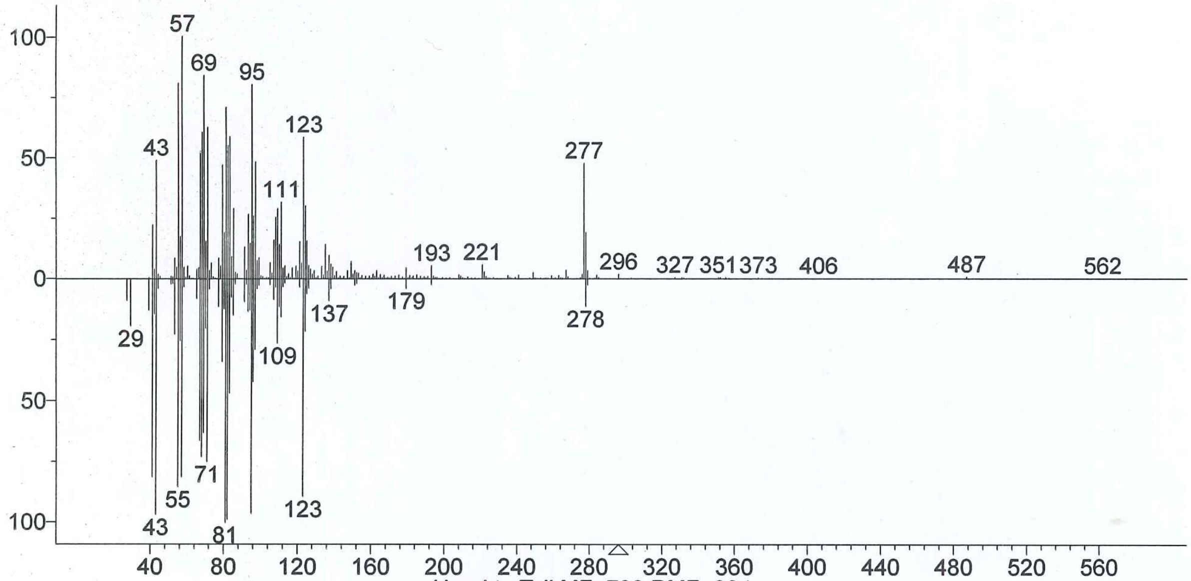

Head to Tail MF=739 RMF=884

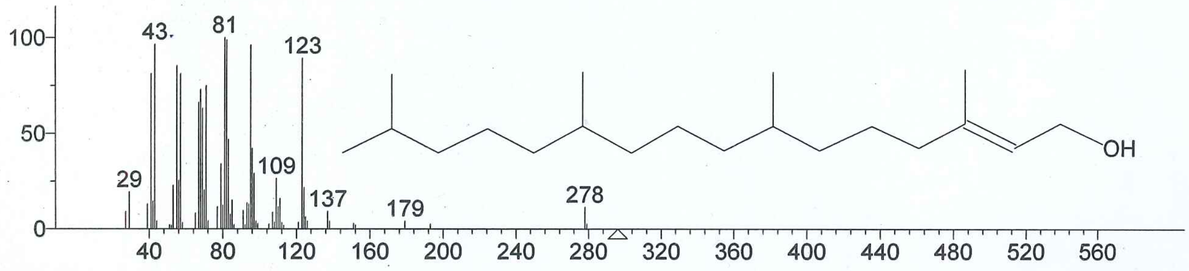

(mainlib) 3,7,11,15-Tetramethyl-2-hexadecen-1-ol

Name: 3,7,11,15-Tetramethyl-2-hexadecen-1-ol
Formula: $C_{20}H_{40}O$
MW: 296 CAS#: 102608-53-7 NIST#: 114703 ID#: 43206 DB: mainlib
Other DBs: IRDB
Contributor: NIST Mass Spectrometry Data Center, 1990.
10 largest peaks:
81 999 | 82 986 | 43 965 | 95 962 | 123 892 | 55 852 | 41 811 | 57 811 | 71 748 | 68 728 |
Synonyms:
1.(2E)-3,7,11,15-Tetramethyl-2-hexadecen-1-ol #

Estimated non-polar retention index (n-alkane scale):
Value: 2045 iu
Confidence interval (Alcohols): 41(50%) 176(95%) iu

Retention index.
1. Value: 2119.33 iu
Column Type: Capillary
Column Class: Semi-standard non-polar
Active Phase: SE
-54
Column Length: 25 m
Column Diameter: 0.31 mm
Data Type: Linear RI
Program Type: Ramp
Start T: 35
C
End T: 230 C
Heat Rate: 4 K/min
Start Time: 3 min
End Time: 10 min
Source: Yin, W.; Xiu, Z.; Aijin, H.,
Analysis of the volatile components in trogopterorum feces by capillary gas chromatography and gas chromatography/mass spectrometry, Fenxi Huaxue, 29(2), 2001, 195-198.
