# Supplementary material for: Gene expression and anticancer evaluation of Kigelia africana (Lam.) Benth. Extracts using MDA-MB-231 and MCF-7 cell lines
Source: PLoS One. 2024 Jun 5;19(6):e0303134. doi: 10.1371/journal.pone.0303134 (PMC11152317; doi:10.1371/journal.pone.0303134)
Supplement: S5 Fig — (DOCX) [file pone.0303134.s005.docx]

| **Sr No.** | **Compounds name** | **MF** | **Ethanol extract**  **(EKA)** | | | **Hexane extract**  **(HKA)** | | |
| --- | --- | --- | --- | --- | --- | --- | --- | --- |
|  |  |  | **RT**  **(min)** | **MW**  **(g/mol)** | **Area %** | **RT**  **(min)** | **MW**  **(g/mol)** | **Area %** |
| 1. | 4-Ethoxy-4-oxobutanoic acid | C_6_H_10_O_4_ | 9 | 146 | 0.16 | - | - | - |
| 2. | 2,6,10-Trimethyltetradecane | C_17_H_36_ | 10.6 | 240 | 0.08 | - | - | - |
| 3. | 2,6,10,15-Tetramethylheptadecane | C_21_H_44_ | 13.5 | 296 | 0.15 | - | - | - |
| 4. | Dihydroactinidiolide | C_11_H_16_O_2_ | 14 | 180 | 0.07 | - | - | - |
| 5. | Propanoic acid, 3-(2,3,6-tri methyl-1,4-dioxaspiro[4.4]non-7-yl)-, methyl ester | C_14_H_24_O_4_ | 14.4 | 256 | 0.14 | - | - | - |
| 6. | Ethyl alpha-d-glucopyranoside | C_8_H_16_O_6_ | 15.1 | 208 | 4.67 | - | - | - |
| 7. | 3,7,11,15-Tetramethyl-2-hexadecen-1-OL | C_20_H_40_O | 17.3 | 296 | 0.41 | - | - | - |
| 8. | Palmitic acid | C_16_H_32_O_2_ | 19 | 256 | 11.33 | 18.9 | 256 | 4.17 |
| 9. | Ethyl palmitate | C_18_H_36_O_2_ | 19.4 | 284 | 1.65 | - | - | - |
| 10 | Phytol | C_20_H_40_O | 21.8 | 296 | 40.37 | 21.7 | 296 | 2.09 |
| 11. | Linolenic acid | C_18_H_30_O_2_ | 22.6 | 278 | 17.11 | 22.4 | 278 | 2.74 |
| 12. | Diisooctyl phthalate | C_24_H_38_O_4_ | 40.1 | 390 | 3.61 | 40.1 | 390 | 0.45 |
| 13. | Squalene | C_30_H_50_ | 44.6 | 410 | 0.7 | 44.6 | 410 | 7.56 |
| 14. | Nonacosane | C_29_H_60_ | 45.3 | 408 | 1.03 | - | - | - |
| 15. | Hentriacontane | C_31_H_64_ | 47 | 436 | 0.94 | 47 | 436 | 21.12 |
| 16. | D-α-Tocopherol | C_29_H_50_O_2_ | 47.3 | 430 | 2.66 | 47.3 | 430 | 5.33 |
| 17. | Stigmasterol | C_29_H_48_O_2_ | 48.4 | 412 | 1.09 | 48.4 | 412 | 1.14 |
| 18. | Clionasterol | C_29_H_50_O | 49 | 414 | 4.56 | 49 | 414 | 4.98 |
| 19. | Dimethyl[bis(tridecyloxy)]silane | C_28_H_60_O_2_Si | 50.1 | 456 | 2.4 | - | - | - |
| 20. | Vitamin E | C_29_H_50_O_2_ | 50.2 | 430 | 0.45 |  |  |  |
| 21. | 3,7,11,15-Tetramethyl-2-hexadecen-1-OL | C_20_H_40_O | 51.7 | 296 | 2.2 | 60 | 296 | 0.6 |
| 22. | Androst-7-ene-6,17-dione, 2,3,14-trihydroxy-, (2beta,3beta,5alpha)- | C_19_H_26_O_5_ | 52.6 | 334 | 1.78 | - | - | - |
| 23. | Isopropyl linolenate | C_21_H_38_O_2_ | 54.7 | 322 | 1.06 | 54.7 | 322 | 0.83 |
| 24. | 3,7,11,15-Tetramethyl-2-hexadecen-1-OL | C_20_H_40_O | 55.1 | 296 | 1.38 | 60 | 296 | 0.6 |
| 25 | Thymol | C_10_H_14_O | - | - | - | 10.8 | 150 | 5.25 |
| 26 | Dihydroactinidiolide | C_11_H_16_O_2_ | - | - | - | 14 | 180 | 0.17 |
| 27 | Diisobutyl phthalate | C_16_H_22_O_~~4~~_ | - | - | - | 17.7 | 278 | 0.14 |
| 28 | Methyl palmitate | C_17_H_34_O_2_ | - | - | - | 18.4 | 270 | 2.55 |
| 29 | Methyl linolenate | C_19_H_34_O_2_ | - | - | - | 21.3 | 294 | 1.66 |
| 30 | Linoleoyl chloride | C_18_H_31_CIO | - | - | - | 21.4 | 298 | 2.05 |
| 31 | Methyl stearate | C_19_H_38_O_2_ | - | - | - | 22 | 298 | 0.52 |
| 32 | Linoleic acid | C_18_H_32_O_2_ | - | - | - | 22.3 | 280 | 1.99 |
| 33 | Dodecanoic acid, 2-butoxyethyl ester | C_18_H_36_O_3_ | - | - | - | 35.7 | 300 | 1.99 |
| 34 | 2-Linoleoylglycerol | C_21_H_38_O_4_ | - | - | - | 41.8 | 354 | 2.5 |
| 35 | Butyl (9Z,12Z,15Z)-9,12,15 octadecatrienoate | C_22_H_38_O_2_ | - | - | - | 41.9 | 334 | 3.53 |
| 36 | Dodecanoic acid, 2-butoxyethyl ester | C_18_H_36_O_3_ | - | - | - | 42.3 | 300 | 0.78 |
| 37 | Heptacosane | C_27_H_56_ | - | - | - | 43.1 | 380 | 0.62 |
| 38 | Octacosane | C_28_H_58_ | - | - | - | 44.3 | 394 | 0.51 |
| 39 | Octacosane | C_28_H_58_ | - | - | - | 45.3 | 394 | 9.54 |
| 40 | ,2,4-trimethyl-3-[(3E,7E,11E)-3,8,12,16-tetramethylheptadeca-3,7,11,15-tetraenyl]cyclohexan-1-ol | C_30_H_52_O | - | - | - | 45.6 | 428 | 0.21 |
| 41 | 2,2,4-trimethyl-3-[(3E,7E,11E)-3,8,12,16-tetramethylheptadeca-3,7,11,15-tetraenyl]cyclohexan-1-ol | C_30_H_52_O | - | - | - | 45.7 | 428 | 0.28 |
| 42 | Heptacosane | C_27_H_56_ | - | - | - | 46.2 | 380 | 1.56 |
| 43 | 13'-Hydroxy-alpha-tocopherol | C_29_H_50_O_3_ | - | - | - | 47.4 | 446 | 1.76 |
| 44 | Tetratriacontane | C34H70 | - | - | - | 47.8 | 478 | 1.08 |
| 45 | Campesterol | C_28_H_48_O | - | - | - | 48.1 | 400 | 0.25 |
| 46 | Triacontane-1,30-diol | C_30_H_62_O_2_ | - | - | - | 48.2 | 454 | 0.31 |
| 47 | Hexatriacontane | C_36_H_74_ | - | - | - | 48.7 | 506 | 6.01 |
| 48 | Cycloartenol | C_30_H_50_O | - | - | - | 49.7 | 426 | 0.24 |
| 49 | Stigmast-4-en-3-one/Sitostenone | C_29_H_48_O | - | - | - | 50.4 | 412 | 1.71 |
| 50 | Hentriacontane | C_31_H_64_ | - | - | - | 50.9 | 436 | 0.73 |
| 51 | Ethanol,2-(9-octadecenyloxy)-, (Z)- | C_20_H_40_O_2_ | - | - | - | 51.7 | 312 | 0.82 |
| 52 | Ethyl iso-allocholate | C_26_H_44_O_5_ | - | - | - | 52.6 | 436 | 0.26 |
